# The evidence for cognitive behavioural therapy in any condition, population or context: a meta-review of systematic reviews and panoramic meta-analysis

**DOI:** 10.1017/S0033291720005292

**Published:** 2021-01

**Authors:** Beth Fordham, Thavapriya Sugavanam, Katherine Edwards, Paul Stallard, Robert Howard, Roshan das Nair, Bethan Copsey, Hopin Lee, Jeremy Howick, Karla Hemming, Sarah E. Lamb

**Affiliations:** 1 Nuffield Department of Orthopaedic, Rheumatology and Musculoskeletal Science (NDORMS) University of Oxford, Oxford, UK; 2 Nuffield Department of Population Health, University of Oxford, Oxford, UK; 3 Reviews and Implementation Group, University of Liverpool, Liverpool, UK; 4 Department for Health, University of Bath, Bath, UK; 5 Institute of Mental Health, University College London, London, UK; 6 Faculty of Medicine & Health Sciences, University of Nottingham, Nottingham, UK; 7 School of Medicine, University of Leeds, Leeds, UK; 8 Department of Philosophy, University of Oxford, Oxford, UK; 9 Public Health, Epidemiology and Biostatistics, University of Birmingham, Birmingham, UK; 10 College of Medicine and Health, University of Exeter, Exeter, UK

**Keywords:** Cognitive Behavioural Therapy, meta-review, overview, panoramic meta-analysis, systematic reviews

## Abstract

The majority of psychological treatment research is dedicated to investigating the effectiveness of cognitive behavioural therapy (CBT) across different conditions, population and contexts. We aimed to summarise the current systematic review evidence and evaluate the consistency of CBT's effect across different conditions. We included reviews of CBT randomised controlled trials in any: population, condition, format, context, with any type of comparator and published in English. We searched DARE, Cochrane, MEDLINE, EMBASE, PsycINFO, CINAHL, CDAS, and OpenGrey between 1992 and January 2019. Reviews were quality assessed, their data extracted and summarised. The effects upon health-related quality of life (HRQoL) were pooled, within-condition groups. If the across-condition heterogeneity was *I*^2^ < 75%, we pooled effects using a random-effect panoramic meta-analysis. We summarised 494 reviews (221 128 participants), representing 14/20 physical and 13/20 mental conditions (World Health Organisation's International Classification of Diseases). Most reviews were lower-quality (351/494), investigated face-to-face CBT (397/494), and in adults (378/494). Few reviews included trials conducted in Asia, South America or Africa (45/494). CBT produced a modest benefit across-conditions on HRQoL (standardised mean difference 0.23; 95% confidence intervals 0.14–0.33, *I*^2^ = 32%). The effect's associated prediction interval −0.05 to 0.50 suggested CBT will remain effective in conditions for which we do not currently have available evidence. While there remain some gaps in the completeness of the evidence base, we need to recognise the consistent evidence for the general benefit which CBT offers.

## Introduction

Cognitive behavioural therapy (CBT) has more evidence supporting it than any other psychological therapy (David, Cristea, & Hofmann, 2018). It aims to improve the quality of life by changing patients' thoughts or thinking patterns considered to maintain problematic symptoms. Randomised controlled trials (RCTs) and systematic reviews of CBT have been conducted across physical and mental conditions in different populations and contexts. An overview of CBT systematic reviews, conducted in 2012, reported it was effective across most conditions (Hofmann, Asnaani, Vonk, Sawyer, & Fang, 2012). However, only 11 of the reviews were based on RCT evidence and no attempt was made to examine the consistency of the effect estimates across different conditions. Since 2012, hundreds of reviews have been published and guidelines have improved the reporting quality of both trials and reviews (Higgins et al., 2019; Moher, Liberati, Tetzlaff, & Altman, 2009; Shea et al., 2017). New methods have been introduced, including panoramic meta-analyses, which synthesise evidence by examining the consistency of effects and when appropriate pooling effect estimates across multiple systematic reviews (Hemming et al., 2013). The aims of this meta-review were to (i) map all reviews of CBT RCTs and (ii) examine whether CBT produces a general effect upon health-related quality of life (HRQoL).

## Methods

We undertook a systematic review of systematic reviews of RCTs evaluating the effect of CBT across all conditions represented in the International Classification of Diseases version 11 (ICD-11, (World Health Organisation, 2018)). The review protocol was prospectively registered (PROSPERO: CRD42017078690) and published (Fordham et al., 2018). Our primary outcome was HRQoL, as this is pertinent to patients across conditions. Our secondary outcomes were depression, anxiety, and pain.

We discussed our design, methods, results and interpretation of our results with an expert consultation group. The group met in person three times during the study and remained in e-mail contact throughout. The group included four patient representatives, six clinicians and eight research academics.

### Eligibility criteria

We included systematic reviews of RCTs (henceforth referred to as reviews): in participants with any condition listed in the ICD-11 (World Health Organisation, 2018) (henceforth referred to as the 20 major physical and 20 major mental conditions); testing an intervention explicitly reported as CBT or including at least one cognitive and one behavioural element; comparing CBT to an active or a non-active comparator; which reported HRQoL, depression, anxiety or pain outcomes; and, fulfilled a minimum of four quality criteria defined by the Centre for Reviews and Dissemination (CRD, 2009): inclusion/exclusion criteria reported, adequate search strategy; RCTs synthesised or only one trial of CBT, trial quality assessed, sufficient trial details reported.

Reviews were excluded if: CBT trials were combined with another intervention and did not provide a separate analysis of CBT; the intervention was third-wave CBT (Hayes, 2004); or, we could not extract separate RCT evidence.

### Information sources

The Database of Abstracts of Reviews of Effects (DARE), the Cochrane Library of Systematic Reviews, MEDLINE, EMBASE, PsycINFO, CINAHL, Child Development and Adolescent Studies, and OpenGrey were searched on 30 January 2019. We restricted inclusion to English language and publication since 1992 [first published systematic review of CBT (Grossman & Hughes, 1992)]. The MEDLINE search strategy is presented in online Supplementary Materials 1.

### Study selection

Results were de-duplicated, entered into Endnote for manual checks and transferred to Covidence (Covidence, Downloaded 2018). The abstract and full-text screen was completed independently and in duplicate (TS, BF) using Covidence.

### Data collection and review quality assessment

As with the full-text selection process, two reviewers (KE and TS or BC), independently performed data extraction (data extraction form available in online Supplementary Materials 2) and quality assessment, using the ‘Assessing the Methodological Quality of Systematic Review, version 2’ (AMSTAR-2) (Shea et al., 2017) tool. Where there was disagreement, a third reviewer (BF) adjudicated and made the final decision.

### Data synthesis

As our extraction and analysis were conducted at the review level, we could not account for the risk of bias in the RCTs included within the reviews. CBT delivery format was classified as high-intensity (face-to-face with a specialist) or low-intensity (face-to-face delivery from a para-professional or self-help techniques) (Roth & Pilling, 2007). Comparator groups were active (e.g. pharmacotherapy) or non-active (e.g. waitlist). Reviews were categorised into ‘higher-quality’ reviews (‘moderate’ or ‘high’ on AMSTAR-2) or lower-quality reviews (‘low’ or ‘critically low’ on AMSTAR-2). Physical conditions were coded using the primary ICD-11 codes and mental conditions were coded with the secondary codes under the primary code of ‘Mental disorders’. The major conditions represented by the reviews were presented in a Bubble chart using Spotfire software (‘Tibco Spotfire’, 2020); frequency information regarding their individual populations and contexts were presented in tables.

### Selection criteria for the panoramic meta-analysis

From the reviews identified, we selected those which reported an effect estimated from a single RCT or a synthesis of RCTs. We included higher-quality reviews in our primary analyses and all-quality reviews in our sensitivity analyses. We selected one meta-analysis per outcome per review based on the following criteria, the meta-analysis with: longest follow-up; largest number of RCTs; the review's primary outcome; continuous outcomes; active prioritised over not-active comparator; and random prioritised over fixed effects meta-analysis.

Next, we listed all the included RCTs. Then we checked if any review included an RCT which was also included in another review. We did not include two reviews which drew upon data from the same trial as this would duplicate the primary data. Therefore, if two reviews included the same RCT, we selected the review with the: highest AMSTAR-2 rating; longest follow-up; conducted most recently; or, contained the largest number of RCTs.

### Panoramic meta-analysis

Data were analysed with a two-step frequentist, random effects panoramic meta-analysis (PMA) [DerSimonian and Laird method (DerSimonian & Laird, 1986)] using the *metan* command in STATA v.13. If a meta-analysis reported a mean difference, we converted the pooled estimate into a standardised mean difference (SMD) using the standard deviation reported (Higgins et al., 2019). Fixed effect and random effect meta-analyses were combined into our random effects model. We did not include meta-analyses of change scores, because of concern that these may be biased due to regression to the mean (Higgins et al., 2019). Our analysis produced a within-condition pooled estimate of the effect with a measure of heterogeneity (*I*^2^). It also generated an across-condition measure of heterogeneity. If this measure was less than *I*^2^ = 75%, which is the cut off for acceptable heterogeneity in the meta-analysis (Higgins et al., 2019), then we pooled an across-condition estimate (henceforth referred to as ‘general effect’). We calculated prediction intervals for our primary analyses. These give an expected range for which the effect estimate for a condition not included in our review will fall. This is subtly different to the confidence intervals of the standardised mean difference, which reflects the certainty which the pooled effect estimate has been estimated across reviews. We multiplied the general effect SMD by the standard deviation of the most commonly used outcome measure, to produce an unstandardized estimate of the mean difference (Higgins et al., 2019). We identified the standard deviation from the lowest risk of bias trial within a higher-quality review.

### Additional panoramic meta-analyses

Sub-group analyses were agreed on a priori to test effects of follow-up time (<12 months and ⩾12 months), age group (<18, 18–65 and >65 years old), CBT delivery format (high- or low-intensity) and comparator type (active or inactive control group). In a sensitivity analysis, we included the lower-quality reviews to examine how review quality affected the results. We performed interaction tests by using the meta-regression *metareg* STATA command. Funnel plots and Egger's test assessed publication bias and small-study effects.

### Changes made to our protocol

Due to resource constraints, we only included English language papers. We restricted our primary analysis to higher-quality reviews as we identified a large volume of reviews suitable for inclusion in the PMA and we found higher-quality reviews reduced the variation of results. We originally planned to analyse a psychosis outcome. However, we revised this decision and chose, instead, to analyse the most frequently reported ‘condition specific’ outcome, which was pain. We did not intend to include prediction intervals however reviewers suggested this would aid our discussion regarding the generalisability of the effect across to conditions not specifically included in our panoramic meta-analyses.

## Results

**Fig. 1.**
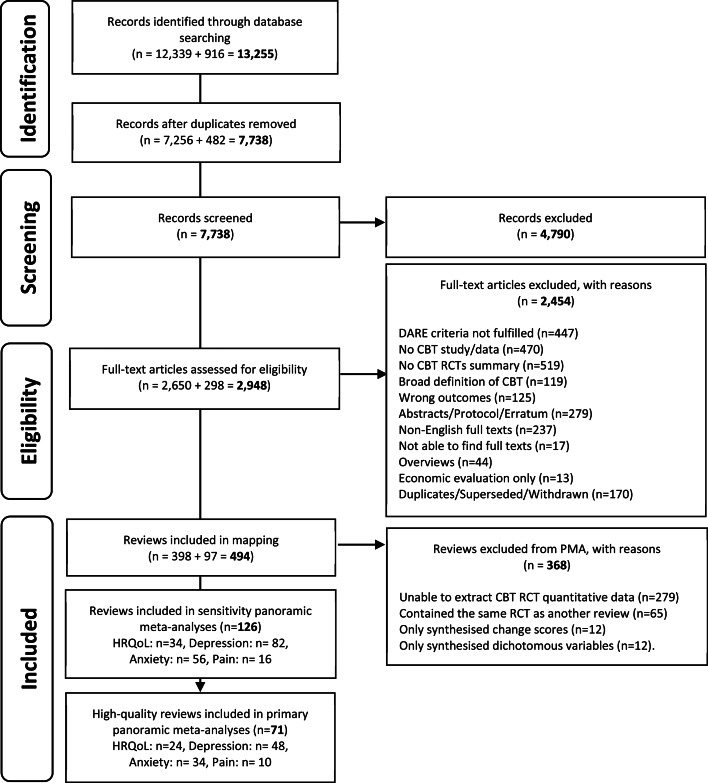
Study selection.

We screened 7738 records and included 498 reviews in our meta-review map. Figure 1 represents the review identification and selection process through the mapping and PMA stages. Reviews were most commonly excluded because we could not extract an isolated synthesis of CBT RCTs (519 reviews excluded). We excluded 237 non-English full texts and could not access 17 full texts. The full list of excluded reviews with reasons is available in online Supplementary Materials 2.

### Review selection

The 494 reviews represented 221 128 participants. The reviews were conducted in 14/20 major physical and 13/20 major mental conditions. The volume of the review evidence is represented across two bubble maps which are available in online Supplementary Material eFigs 1 and 2.

### Review characteristics

The demographic and context characteristics of the trial samples included in the mapped reviews (*n* = 494) are presented in online Supplementary Materials eTables 1 and 2.

The quality of the reviews we identified was poor (351/494 were low or critically-low). Most reviews considered the effects of high-intensity CBT (397/494), delivered as an intervention rather than a preventative programme (463/494), in the short-term (<12 months) (402/494), and in the adult population (378/494). Research with older adults was limited (30/494). Reporting on condition severity (247/494) and the recruitment setting was poor (283/494). Nearly half of the included reviews did not report details on sex (218/494), ethnicity (459/494) or the country where the trials were conducted (218/494). Only 45/494 reviews reported including trials conducted in the Asian, South American and African continents.

### Panoramic meta-analyses

From the reviews identified in the mapping exercise, most (368/494) were not included in our evaluation of the consistency of effects across conditions because we could not extract suitable data. From the 126 reviews with suitable data, 71 (20 862 participants) reviews were higher-quality and included in our primary PMAs.

### Primary outcome: HRQoL

**Fig. 2.**
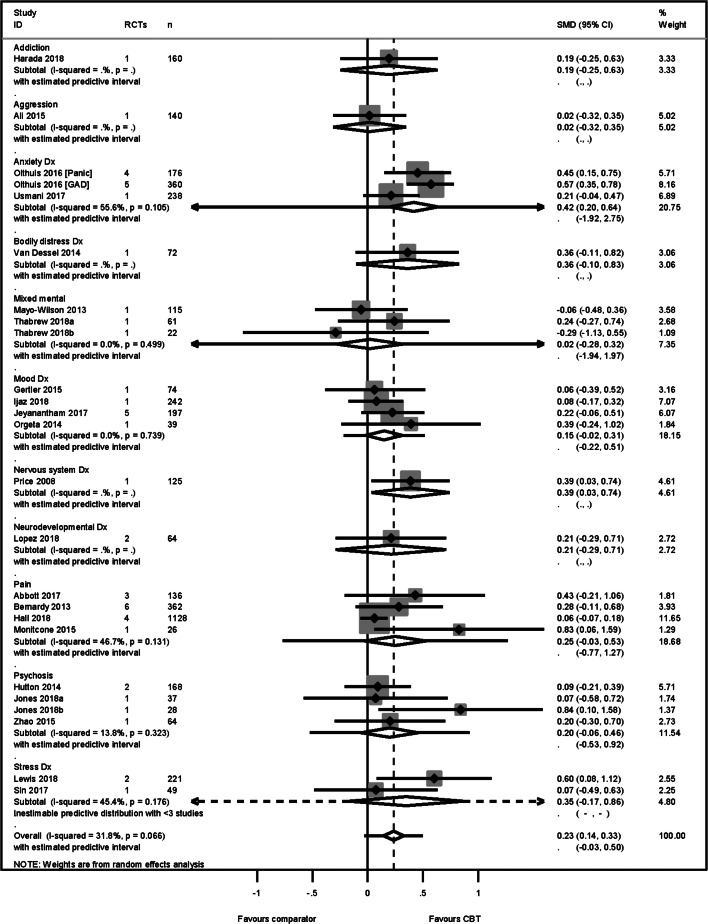
HRQoL primary PMA.

The HRQoL analysis included data from 24 higher-quality reviews (4304 participants), representing 10 different conditions. Each of these conditions produced an effect in favour of CBT. Heterogeneity across the conditions was low (Higgins et al., 2019) (*I*^2^ = 32%), and therefore we pooled across conditions. We found a modest effect in favour of CBT for HRQoL, SMD 0.23 [95% confidence intervals (95% CI) 0.14–0.33] (Fig. 2). Variation in effects was observed across conditions, for example, in aggression, the estimate mean effect was almost zero although estimated with considerable uncertainty (SMD: −0.02, 95% CI −0.28 to 0.32); whereas in anxiety disorders the estimated effect was positive and estimated with much greater certainty (SMD: 0.42, 95% CI 0.20–0.64). This heterogeneity is reflected in the resulting prediction intervals, which indicated for the overall effect (within any given condition) was between −0.03 and 0.50, indicating at worst (and with little support in the prediction interval) a small negative effect of CBT for some conditions and at best a large positive effect for other conditions.

The SMD was transformed into a mean difference of 3 points (95% CI 2–4) on the mental component score of the SF-36 (Ware, Davies, & Donald, 1978), using the standard deviation = 10.93 identified from a low risk-of-bias RCT (Kunik et al., 2008) included in a higher-quality review (Usmani et al., 2017). No publication nor small study biases were detected [Egger's bias = 0.18 (95% CI −0.34 to 1.76) *p* = 0.18].

### Secondary outcomes: depression

We found considerable heterogeneity (*I*^2^ = 81%) between estimates of CBT's effectiveness on depression outcomes across the 14 conditions (48 reviews and 14 073 participants). The majority of the conditions reported effect estimates in favour of CBT however estimates from reviews of aggressive behaviour and mixed mental health reviews i.e. reviews combining trials in anxiety, depression, post-traumatic stress disorder, and obsessive-compulsive disorders, reported negative estimates. Therefore, we did not pool across the conditions. The depression analysis is presented in online Supplementary Material eFig. 3. No publication or small-study bias was detected [Egger's bias = 0.17 (95% CI −1.21 to 1.44) *p* = 0.87].

### Anxiety

**Fig. 3.**
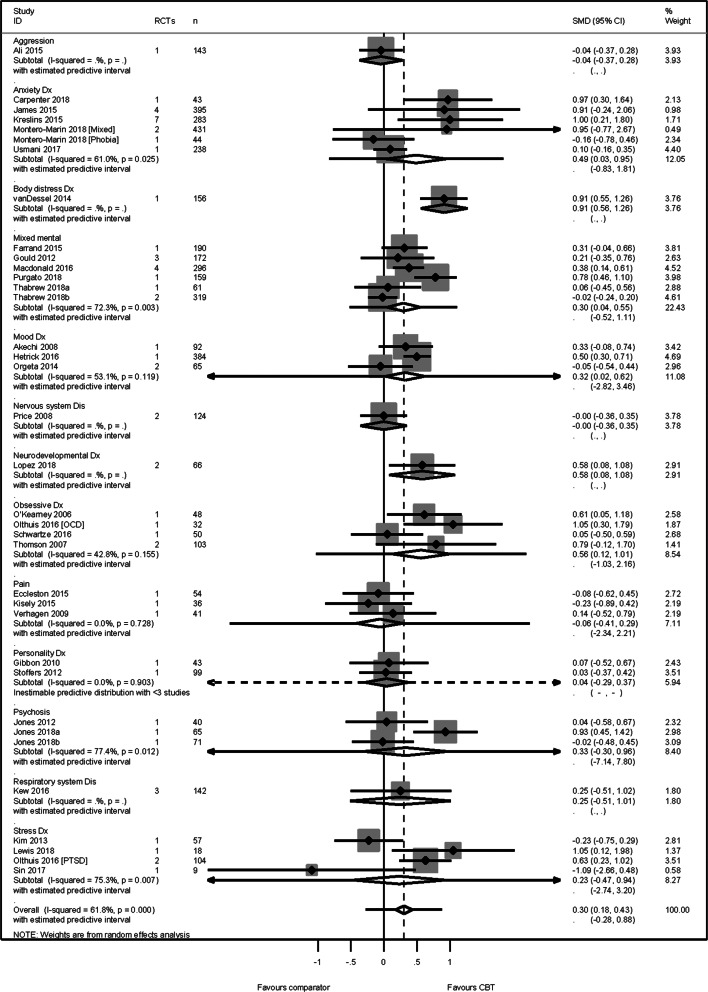
Anxiety primary PMA.

The anxiety analysis included data from 34 reviews (4673 participants), representing 12 conditions. Heterogeneity across the conditions was substantial yet acceptable (*I*^2^ = 62%) and therefore we pooled across conditions. We found a modest effect in favour of CBT SMD 0.30 (95% CI 0.18–0.43) and prediction intervals −0.28 to 0.88 (Fig. 3). This translated to a mean improvement of 4 points on the Beck Anxiety Inventory (Beck & Steer, 1993) [s.d. = 13.46 identified from (Kunik et al., 2008; Usmani et al., 2017)]. No publication or small sample bias was detected [Egger's bias = 0.39 (95% CI −1.03, 1.52) *p* = 0.70].

### Pain outcome

**Fig. 4.**
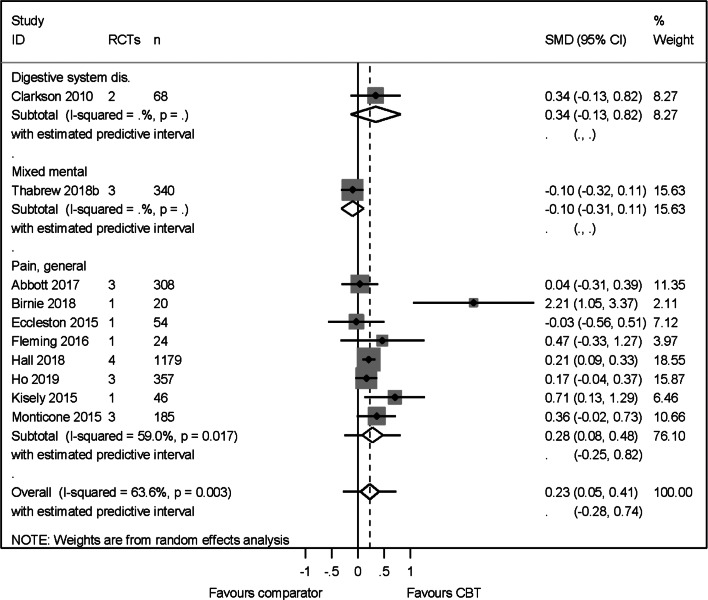
Pain primary PMA.

We pooled results from ten higher-quality reviews (2581 participants), across abdominal, leukaemia-related, non-specific chest, osteoarthritis, spinal, back and neck pain. The across condition heterogeneity was substantial, yet acceptable (*I*^2^ = 64%) and the across condition pooled effect was modest in favour of CBT SMD 0.23 (95% CI 0.05–0.41) and prediction interval of −0.28 to 0.74 (Fig. 4). The effect translated to a change of 6 mm (95% CI 1–11 mm) on the visual analogue scale [VAS, (Huskisson, 1974)] for pain. No publication or small sample bias was detected [Egger's bias = 1.44 (95% CI −0.74 to 3.25) *p* = 0.19].

### Additional panoramic meta-analyses

Results from the sub-group analyses are presented in Table 1. In the HRQoL analyses, we found a statistically significant interaction between reviews which compared CBT to an active as opposed to a non-active comparator (*p* = 0.04). The effect size was larger in the non-active comparator subgroup SMD 0.31 (95% CI 0.18–0.45) than the effect comparing CBT to an active comparator SMD 0.09 (95% CI −0.01 to 0.19). The active comparator interventions were psychoeducation, relaxation, psychotherapy, counselling and physical exercise. None of the remaining interaction tests between the age groups, CBT delivery format or length of follow-up subgroups was statistically significant.

**Table 1.** Sub-group analyses for HRQoL, anxiety and pain outcomes

	HRQoL SMD (95% CI), *I*^2^ across conditions [No. meta-analyses (MAs)]	Anxiety SMD (95% CI), *I*^2^ across conditions [No. meta-analyses (MAs)]	Pain SMD (95% CI), *I*^2^ across conditions [No. meta-analyses (MAs)]
Follow-up			
Long (≥12 months)	0.11 (0.02–0.20) *I*^2^ = 0% (9 MAs)	0.38 (0.15–0.60), *I*^2^ = 65.9% (10 MAs)	0.19 (0.08–0.31), *I*^2^ = 0% (2 MAs)
Short (<12 months)	0.29 (0.17–0.42) *I*^2^ = 29.5% (15 MAs)	0.27 (0.12–0.43), *I*^2^ = 59.4% (26 MAs)	0.32 (0.04–0.59), *I*^2^ = 70.5% (8 MAs)
Interaction effect	*p* = 0.06	*p* = 0.48	*p* = 0.62
Age			
<18 years	0.20 (−0.15 to 0.56) *I*^2^ = 0% (3 MAs)	0.37 (0.12–0.62) *I*^2^ = 67.1% (7 MAs)	Too heterogenous *I*^2^ = 86.5% (3 MAs)
18–65 years	0.23 (0.14–0.33) *I*^2^ = 39.4% (21 MAs)	0.32 (0.15–0.48) *I*^2^ = 63.6% (26 MAs)	0.21 (0.12–0.31) *I*^2^ = 0% (6 MAs)
>65 years	0.39 (−0.24 to 1.02) *I*^2^ = NA (1 MA)	0.06 (−0.30 to 0.43) *I*^2^ = 0% (2 MAs)	No data available
Interaction effect	*p* = 0.86	*p* = 0.69	*p* = 0.68
CBT intensity			
High	0.21 (0.11–0.32) *I*^2^ = 0% (14 MAs)	0.28 (0.15–0.42) *I*^2^ = 54.3% (28 MAs)	0.19 (0.01–0.37) *I*^2^ = 17.5% (5 MAs)
Low	0.23 (0.03–0.42) *I*^2^ = 68.1% (9 MAs)	Too heterogenous *I*^2^ = 78.4% (8 MAs)	Too heterogenous *I*^2^ = 84.1% (4 MAs)
Interaction effect	*p* = 0.99	*p* = 0.62	*p* = 0.87
Control group			
Active	0.09 (−0.01 to 0.19) *I*^2^ = 0% (8 MAs)	0.19 (−0.00 to 0.37) *I*^2^ = 48.6% (14 MAs)	0.14 (−0.11 to 0.38) *I*^2^ = 72.8% (3 MAs)
Not active	0.31 (0.18–0.45) *I*^2^ = 40.3% (14 MAs)	0.37 (0.19–0.55) *I*^2^ = 64.3% (20 MAs)	0.59 (0.07–1.11) *I*^2^ = 68.8% (5 MAs)
Interaction effect	*p* = 0.04*	*p* = 0.24	*p* = 0.86

*Statistically significant interaction effect at *p* = 0.05.

### Sensitivity analyses

The HRQoL sensitivity analysis included additional 10 lower-quality reviews. In comparison to the primary analysis, the effect size did not change SMD 0.28 (95% CI 0.17–0.38) but the heterogeneity increased (*I*^2^ = 71%). The heterogeneity in the anxiety sensitivity analysis became too high for us to pool across conditions (*I*^2^ = 76%). The pain sensitivity analysis included six additional lower quality reviews. In comparison to the primary analysis, the effect size remained similar SMD 0.21 (95% CI 0.11–0.31) but the heterogeneity increased (*I*^2^ = 51%).

### Consistency of effect across conditions

We have evidence from higher-quality reviews that CBT improves HRQoL, anxiety and pain outcomes across 10 mental and four physical conditions. Evidence from lower-quality reviews, (sensitivity analyses) remained consistent, albeit with higher heterogeneity levels, with our higher-quality review evidence and introduced one more condition (cancer). The reviews included in the primary and sensitivity analyses tested the effectiveness of CBT in populations who had reported the following co-morbid conditions: intellectual disabilities, brain injury, dementia, migraines, epilepsy, circulatory diseases, chronic obstructive pulmonary disease, irritable bowel syndrome, arthritis, tinnitus and fatigue. Consequently, we have identified evidence that CBT improves HRQoL, anxiety and pain outcomes in participants living with 22 of the 40 major conditions. The 10 physical conditions which were not represented in any PMAs were disorders of the blood, infectious, immune, sleep, ear, skin, sexual health, pregnancy, during the puerperium, developmental abnormalities. The eight mental conditions not represented were catatonia, dissociative, eating, elimination, impulse control, disruptive behaviour, paraphilic, and factitious disorders.

## Discussion

We have mapped reviews of CBT's effectiveness across 27 of the 40 major physical and mental conditions. We were able to synthesise evidence from the highest quality reviews, published in English, to determine the consistency of effects across 14 of these conditions (plus eight co-morbidities). From this apex of current evidence, we found a consistency in effect estimates, which suggests that CBT improves the quality of life by a modest amount, irrespective of the underlying condition(s). Our prediction intervals suggest this effect will remain in favour of CBT for meta-analyses conducted in other conditions from these ICD-11 categories.

The improvements were maintained for more than 12 months after receiving CBT and were evident if CBT was delivered through high- or low-intensity formats. CBT appears to help children and adults but there is little evidence available for older adults. Our sensitivity analyses suggest that higher- and lower-quality reviews both estimate similar effects of CBT upon HRQOL, anxiety and pain outcomes but lower-quality reviews increase the degree of variability in the estimates.

Reviews have shown CBT is effective in reducing depression outcomes for people with clinical depression (López-López et al., 2019; NICE, 2009). However, when we examined the heterogeneity of effects upon depression outcomes across reviews of CBT for clinical depression, we found the review effects varied greatly and we could not pool across these reviews. Our mapping exercise showed that clinical depression was, by far, the most common condition represented by our included reviews. Perhaps CBT for clinical depression has been tested in some populations or contexts where CBT is less effective thus generating greater variation of effects. This breadth of research may explain the high levels of within- and across-condition heterogeneity. However, we only found one review which reported an effect estimate which was statistically significant and in favour of the comparator to CBT (Akechi, Okuyama, Onishi, Morita, & Furukawa, 2008). This review was higher-quality but included data from just one RCT with 92 participants and therefore does not demonstrate a robust model of inconsistency.

Our meta-review identified some gaps in the CBT review evidence base. For example, data from the very young (<6 years), older adults (65–80 years), and the oldest old (>80 years); and whether ethnicity or country of residence moderates CBT effectiveness. Considering each category of reviews, we interrogated for HRQoL, anxiety and pain produced a result consistent with the general effect, these may generalise to the under-represented categories quite readily. It may be more important to examine issues of access and acceptability for these populations.

The major strength of this meta-review is that by pooling data from many reviews across conditions we become more certain of the effect estimates. Our HRQoL and anxiety outcome estimates include more than 4000 participants, which guidance suggests indicates a certain effect (Schünemann, Brożek, Guyatt, & Oxman, 2013).

A limitation of our methodology was the extraction of data only at the review level. This meant that we excluded many reviews which included relevant RCTs, but had combined these with trials of other interventions. If we had extended our methods to extract data from individual RCTs which had been identified by reviews this may have been a more comprehensive picture of the CBT evidence base. Another weakness was the exclusion of reviews published in languages other than English (237 reviews). These reviews may not have met all the inclusion criteria but if they did, they might have addressed the evidence gap we identified of few trials having been conducted in Africa, Asia or South America.

Our CBT intensity subgroup analyses suggested no difference in effectiveness between using high- and low-intensity CBT. This is not to say that low-intensity is equally effective as high-intensity CBT, only that, where it has been deemed appropriate to use and has been empirically tested, low-intensity CBT has been effective. Reviews of high-intensity CBT produced broadly similar estimates of CBT's effectiveness, whereas, the estimates from low-intensity reviews were highly varied. The large variation may be due to our definition of low-intensity CBT (Roth & Pilling, 2007). We combined face-to-face delivery of CBT by paraprofessionals with self-help CBT and therefore we do not know if these two methods of delivery moderate the effectiveness of CBT.

This meta-review suggests CBT works, it improves the quality of life for people living with many different mental and physical conditions. For some conditions, we do not currently know precisely by how much it works only that it will provide small to moderate effects. The clinical implications of CBT producing consistent benefits which are not specific to any one condition will influence future clinical commissioning and implementation decisions. The research implications of this meta-review suggest shifting the focus away from broad CBT effectiveness research and instead focussing on how to increase the modest effect sizes seen with CBT. For example, identifying alternative delivery formats to increase adherence and reduce dropout, and pursuing novel methods to assess intervention fidelity and quality. The biggest area of uncertainty is around whether factors such as ethnicity, religion, culture, country or language could moderate the effectiveness of CBT or whether it will be equally effective across these constructs.

## Supplementary material

For supplementary material accompanying this paper visit https://doi.org/10.1017/S0033291720005292. click here to view supplementary material
