# Supplementary material for: The evidence for cognitive behavioural therapy in any condition, population or context: a meta-review of systematic reviews and panoramic meta-analysis
Source: Psychol Med. 2021 Jan 18;51(1):21–9. doi: 10.1017/S0033291720005292 (PMC7856415; doi:10.1017/S0033291720005292)
Supplement: Supplementary file 1 [file S0033291720005292sup001.pdf]

<u>Online-only supplements</u>

<u>1: Search strategy example (MEDLINE)</u>

MEDLINE
Database & platform:
Ovid MEDLINE(R) Epub Ahead of Print, In-Process & Other Non-Indexed Citations, Ovid MEDLINE(R) Daily and Ovid MEDLINE(R) 1946 to Present
Search filter:
SIGN Systematic Review Search Filter for MEDLINE (OVID) (http://www.sign.ac.uk/search-filters.html)
Date search conducted:
Original: 25 April 2018 (for publication years 1992 to Present) [2967 references]
Update: 30 January 2019 (for publication years 2018 to 2019) [359 references]

1. (cognitive adj2 behavio?r adj3 (therap$ or theor$ or intervention$ or train$ or treatment$ or psychotherap$ or programme$ or program$ or method$ or approach$)).ti,ab,kw.
2. (cognitive adj2 behavio?ral adj3 (therap$ or theor$ or intervention$ or train$ or treatment$ or psychotherap$ or programme$ or program$ or method$ or approach$)).ti,ab,kw.
3. CBT.ti,ab,kw.
4. Cognitive Therapy/
5. or/1-4
6. Meta-Analysis as Topic/
7. meta analy$.tw.
8. metaanaly$.tw.
9. Meta-Analysis/
10. (systematic adj (review$1 or overview$1)).tw.
11. exp Review Literature as Topic/
12 or/6-11
13. cochrane.ab.
14. embase.ab.
15. (psychlit or psyclit).ab.
16. (psychinfo or psycinfo).ab.
17. (cinahl or cinhal).ab.
18. science citation index.ab.
19. bids.ab.
20. cancerlit.ab.
21. or/13-20
22. reference list$.ab.
23. bibliograph$.ab.
24. hand-search$.ab.
25. relevant journals.ab.
26. manual search$.ab.
27. or/22-26
28. selection criteria.ab.
29. data extraction.ab.
30. 28 or 29
31. Review/
32. 30 and 31
33. Comment/
34. Letter/
35. Editorial/
36. animal/
37. human/
38. 36 and 37
39. 36 not 38
40. or/33-35,39
41. 12 or 21 or 27 or 32
42. 41 not 40
43. 5 and 42
44. limit 43 to yr="1992-2018"

2. Data extraction form

	Data Extraction	Notes
**Review ID (Surname Year)**		
**Reviewer completing form**		
**Date completed**		
**Reference citation (first author, title, journal, volume, issue)**		
**Published within last 5 years (Y/N)**		
**Aim of the review**		
**Design of included studies [RCT n= [participants n=]]**		
**Any risk of bias tool employed (Y/N) [Detail]**		RoB not just quality assessment tool. Report the actual tool used in 'details'
**Primary health problem**		Report ICD-11 for every health problem reported
**Secondary health problem**		
**Severity (mild, moderate, Severe, NR)**		Select one or many or 'unclear'
**Age categories [No.RCTs]**		Select one or many of children, adolescents, adults, older adults or 'not reported' or 'unclear'
**Other characteristics reported: (1) Gender, (2) ethnicity (3) other specific/unique information**		Report top level information on (1) Gender (2) Ethnicity (3) if available other information (but do not need to search)
**Where recruited [No.RCTs]**		Report as the review has reported: Clinic, University, internet, etc
**When delivered [No.RCTs]**		Dropdown list [(1) preventative (2) preventative for relapse (3) early intervention (4) standard treatment (5)mixed (6)not reported (7)other (standard treatment is the norm, the others are if review specifies a specific target)
**Countries included [No.RCTs]**		
**CBT high/low/combined: description of CBT**		**High intensity**: Formal psychotherapy delivered by relatively specialist psychological therapist<br>**Low intensity**: Guided self-help: books, internet, structured exercise, brief interventions can be with relevantly trained individual.
**CBT overall no.session, duration and frequency**		As much as is available within the review. Can synthesise ourselves but only at this top level.
**CBT 1 content description [No.RCTs/Total RCTs]**		Report high intensity intervention first then low intensity
**CBT description 2 [No.RCTs/Total]**		Complete for every type of CBT category the review includes
**CBT description 3 [No.RCTs/Total]**		
**CBT description 4 [No.RCTs/Total]**		
**CBT description 5 [No.RCTs/Total]**		
**Control description 1 [No.RCTs]**		If review synthesises all no-active/active together then extract as such, if reported as separate control groups then we can extract as such then later we will combine.
**Control description 2 [No.RCTs]**		
**Control description 3 [No.RCTs]**		
**Control description 4 [No.RCTs]**		
**Control description 5 [No.RCTs]**		
**Other details:**		Only most pertinent information if required
**HRQoL category**		Chose (1) category (2)category but no data available (3) not measured. If HRQL emerges at individual RCT level then we extract (but only for HRQL)
**How measured [name(s) of instruments/Method]**		Specific name of outcome
**When measured [pegged timepoint?]**		Short (majority is <12 months) Long (majority >= 12 months) or 'unclear' [if the review reports where the follow-up timepoints are pegged to i.e. post-randomisation, post intervention. Then report]
**No. RCTs [no. participants]**		Number of RCTs [number of participants]
**Meta-analysis [Y/N]**		If no meta-analysis can we just report direction of results

**Depression category**		Chose (1) category (2)category but no data available (3) not measured.
**How measured [name(s) of instruments/Method]**		Specific name of outcome
**When measured [pegged timepoint?]**		Short (majority is <12 months) Long (majority >= 12 months) or 'unclear' [if the review reports where the follow-up timepoints are pegged to i.e. post-randomisation, post intervention. Then report]
**No. RCTs [no. participants]**		Number of RCTs [number of participants]
**Meta-analysis [Y/N]**		If no meta-analysis can we just report direction of results
**Anxiety category**		Chose (1) category (2)category but no data available (3) not measured.
**How measured [name(s) of instruments/Method]**		Specific name of outcome
**When measured [pegged timepoint?]**		Short (majority is <12 months) Long (majority >= 12 months) or 'unclear' [if the review reports where the follow-up timepoints are pegged to i.e. post-randomisation, post intervention. Then report]
**No. RCTs [no. participants]**		Number of RCTs [number of participants]
**Meta-analysis [Y/N]**		If no meta-analysis can we just report direction of results
**Physical/Physiological category**		Chose (1) category (2)category but no data available (3) not measured.
**How measured [name(s) of instruments/Method]**		Specific name of outcome
**When measured [pegged timepoint?]**		Short (majority is <12 months) Long (majority >= 12 months) or 'unclear' [if the review reports where the follow-up timepoints are pegged to i.e. post-randomisation, post intervention. Then report]
**No. RCTs [no. participants]**		Number of RCTs [number of participants]
**Meta-analysis [Y/N]**		If no meta-analysis can we just report direction of results
**Psychosis category**		Chose (1) category (2)category but no data available (3) not measured.
**How measured [name(s) of instruments/Method]**		Specific name of outcome
**When measured [pegged timepoint?]**		Short (majority is <12 months) Long (majority >= 12 months) or 'unclear' [if the review reports where the follow-up timepoints are pegged to i.e. post-randomisation, post intervention. Then report]
**No. RCTs [no. participants]**		Number of RCTs [number of participants]
**Meta-analysis [Y/N]**		If no meta-analysis can we just report direction of results
**All other outcomes reported in review**		List format e.g. (1) PANSS psychosis
**Overall**		See AMSTAR 2 pdf
**Mechanism data**		Extraction of entire section 'How the intervention might work' (for Cochrane Reviews), or similar (other reviews), and/or Direct data: i.e. changes to beliefs such as self-efficacy or hypotheses for mechanisms such as presented in the discussions
**Acceptability**		
**Satisfaction**		
**Adverse Effects**		
**Economic analyses**		

3. Full list of excluded reviews with reasons.

a. References of studies excluded due to no English full text or translation available (237)

Abdel-Baki A, Nicole L. [Schizophrenia and cognitive-behavioural therapy (CBT)] *Canadian Journal of Psychiatry* 2001;**46**:511-21.

Aghaie E, Abedi A, Paghale SJ. [Meta-analysis of the effectiveness of cognitive-behavior interventions in the reduction 3 of test anxiety in Iran]. *Iranian Journal of Psychiatry and Clinical Psychology* 2012;**18**:3-12.

Ahmadnia E, Haseli A, Karamat A. [Therapeutic interventions conducted on improving women's sexual satisfaction and function during reproductive ages in Iran: A systematic review]. *Journal of Mazandaran University of Medical Sciences* 2017;**27**:146-62.

Ahonen S, Kivela SL. [Effects of cognitive and behavioral treatments on primary insomnia in old age]. *Duodecim* 2010;**126**:794-802.

Albert U, Barbaro F, Aguglia A, Maina G, Bogetto F. [Combined treatments in obsessive-compulsive disorder: Current knowledge and future prospects]. *Rivista di Psichiatria* 2012;**47**:255-68.

Algar MJM, Garcia PB. [Approach to anxiety in patients diagnosed with cancer]. *Psicooncologia* 2016;**13**:227-48.

Almeida Lima Junior N, Lopes Paes DG, Belchior Pontes GC, Gomes Sancho A, da Silva Rosa JL, Dias Faria ÁC. Possíveis impactos do transtorno de ansiedade social no processo de envelhecimento. *Fisioterapia Brasil* 2018;**19**:577-81.

Almeida AM, Lotufo Neto F. [Cognitive-behavioral therapy in prevention of depression relapses and recurrences: a review]. *Revista Brasileira de Psiquiatria* 2003;**25**:239-44.

Ambresin G, De Roten Y, Despland JN. [Psychotherapy of depression in primary care] *Schweizer Archiv fur Neurologie und Psychiatrie* 2016;**167**:147-54.

Andanson J, Pourre F, Maffre T, Raynaud JP. [Social skills training groups for children and adolescents with Asperger syndrome: A review] Les groupes d'entrainement aux habiletes sociales pour enfants et adolescents avec syndrome d'Asperger: revue de la litterature. *Archives de Pediatrie* 2011;**18**:589-96.

Arteriole N. *[Cognitive-behavioral therapy in bipolar disorder] Thérapies cognitivo-comportementales du trouble bipolaire*: Université de Caen Normandie & Université de Caen UFR de médecine; 2013.

Auclair V, Harvey PO, Lepage M. [Cognitive Behavioral Therapy and the Treatment of ADHD in Adults]. *Sante Mentale au Quebec* 2016;**41**:291-311.

Bacaltchuk J, Hay P. [Treatment of bulimia nervosa: A synthesis of evidence]. *Revista Brasileira de Psiquiatria* 1999;**21**:184-7.

Banti S, Borri C, Montagnani MS, Cargioli C, Belli S, Cotugno B*, et al.* [Premenstrual disphoric disorder: An update]. Sindrome disforica premestruale: Un aggiornamento. *Journal of Psychopathology* 2012;**18**:261-72.

Barreto EMDP, Elkis H. [Evidences from the efficacy of the cognitive behavior therapy on schizophrenia] Evidencias de eficacia da terapia cognitiva comportamental na esquizofrenia. *Revista de Psiquiatria Clinica* 2007;**34**:204-7.

Baum E, Maisel P, Dorr C, Donner-Banzhoff N. [Update of the DEGAM-guideline fatigue: New developments and recommendations] Aktualisierung der DEGAM-leitlinie mudigkeit: Neue entwicklungen und empfehlungen. *Zeitschrift fur Allgemeinmedizin* 2012;**88**:133-7.

Beelmann A, Schneider N. [The effects of psychotherapy with children and adolescents. A review and meta-analysis of German-language research] Wirksamkeit von psychotherapie bei kindern und jugendlichen. Eine

ubersicht und meta-analyse zum bestand und zu ergebnissen der deutschsprachigen effektivitatsforschung. *Zeitschrift fur klinische Psychologie und Psychotherapie* 2003;**32**:129-43.

Belanger L, Vallieres A, Morin CM. [Insomnia and the increased use of sleeping pills among the elderly: Problems and alternative therapy] Insomnie et utilisation accrue de somniferes chez les aines: Problematique et alternative de traitement. *Canadian Family Physician* 2006;**52**:968-73.

Bellino S, Zizza M, Di Lorenzo R, Paradiso E, Falakfarsa R, Fulcheri M*, et al.* [Combined therapy of major depressive disorder: A critical review] Terapia combinata del disturbo depressivo maggiore: Revisione critica della letteratura. *Italian Journal of Psychopathology* 2002;**8**:401-16.

Bellver Perez A, Moreno P. [Psychosocial risks and psychological intervention in the transplanted bone marrow patients] Riesgos psicosociales e intervencion psicologica en los pacientes trasplantados de medula osea. *Psicooncologia* 2009;**6**:65-81.

Blanca-Gutierrez JJ, Jimenez-Diaz MDC, Escalera-Franco LF. [Effective interventions to reduce absenteeism among hospital nurses] Intervenciones eficaces para reducir el absentismo del personal de enfermeria hospitalario. *Gaceta Sanitaria* 2013;**27**:545-51.

Block I, Loeber S. [Evidence-based psychotherapy of addictive disorders] Evidenzbasierte Psychotherapie bei Abhangigkeitserkrankungen. *Nervenarzt* 2018;**89**:283-9.

Bomba J. [Research on psychotherapy effectiveness in treatment of mental and behavioural disorders in children and adolescents] Badania nad efektywnoscia psychoterapii w leczeniu zaburzen psychicznych u dzieci i mlodziezy. *Psychoterapia* 2010:37-47.

Bornas X, Rodrigo T, Barcelo F, Toledo M. [New technologies in cognitive-behavioral therapy: A review]. *International Journal of Clinical and Health Psychology* 2002;**2**:533-41.

Boschi M, Santandrea S, Vanti C. [Efficacy of cognitive behavioural therapy in non-specific neck pain: a systematic review]. *Scienza Riabilitativa* 2010;**12**:5-15.

Bottlender M, Kohler J, Soyka M. [The effectiveness of psychosocial treatment approaches for alcohol dependence - A review] Effektivitat psychosozialer behandlungsmethoden zur medizinischen rehabilitation alkoholabhangiger patienten. *Fortschritte der Neurologie Psychiatrie* 2006;**74**:19-31.

Boudebesse C, Henry C. [Emotional hyper-reactivity and sleep disturbances in remitted patients with bipolar disorders] Perturbations de la reactivite emotionnelle et du sommeil dans les troubles bipolaires en periode intercritique. *Encephale* 2012;**38**:S173-S8.

Brandao T, Mena Matos P. [Efficacy of psychological group interventions for women with breast cancer: A systematic review] Eficacia das intervencoes psicologicas em grupo dirigidas a mulheres com cancro da mama: uma revisao sistematica. *Revista Portuguesa de Saude Publica* 2015;**33**:98-106.

Brown RC. [Psychotherapeutic interventions for suicidal adolescents - A systematic review of the current literature]. *Zeitschrift fur Kinder- und Jugendpsychiatrie und Psychotherapie* 2017;**45**:499-508.

Bruun Wyller V, Bjorneklett A, Brubakk O, Festvag L, Follestad I, Malt U*, et al. [Diagnosis and Treatment of Chronic Fatigue Syndrome/Myalgic Encephalopathy (CFS/ME)]*. Report from Knowledge Centre No. 9: Oslo: National Knowledge Centre for Health Services at the Norwegian Institute of Public Health (NIPH); 2006.

Calzolari L, Fioravanti G. [A comparison between Acceptance and Commitment Therapy and Cognitive Behavioural Therapy: A review of literature]. *Psicoterapia Cognitiva e Comportamentale* 2016;**22**:103-17.

Caselli G, Manfredi C, Ruggiero GM, Sassaroli S. [Cognitive Behavioural Therapy for anxiety disorders: A review of efficacy studies]. *Psicoterapia Cognitiva e Comportamentale* 2016;**22**:81-101.

Castelein S, Knegtering H, Van Meijel B, Van Der Gaag M. [Dutch guideline on Schizophrenia 2012: Basic care within the areas of psychosocial interventions and nursing care] Richtlijn Schizofrenie 2012: Basiszorg

op het gebied van psychosociale interventies en verpleegkundige zorg. *Tijdschrift voor Psychiatrie* 2013;**55**:707-14.

Cavadas LF, Ribeiro L. [Management of adult secondary insomnia in Primary Health Care] Abordagem da insonia secundaria do adulto nos Cuidados de Saude Primarios. *Acta Medica Portuguesa* 2011;**24**:135-44.

Copanitsanou P, Sourtzi P. [The Effect of Educational Interventions for the Reduction of Nursing Staff's Occupational Stress: Systematic Review]. *Nosileftiki* 2016;**55**:250-62.

Cuijpers P, Dekker J. [Psychological treatment of depression; a systematic review of meta-analyses] Psychologische behandeling van depressie; een systematisch overzicht van meta-analysen. *Nederlands Tijdschrift voor Geneeskunde* 2005;**149**:1892-7.

Daga G, Quaranta M, Notaro G, Urani C, Amianto F, Fassino S. [Family therapy and eating disorders in young female patients: State of the art]. *Giornale Italiano di Psicopatologia / Italian Journal of Psychopathology* 2011;**17**:40-7.

Dahm KT, Landmark B, Kirkehei I, Reinar LM. *[The Effects of School Health Services for Children and Young People's Health and Growing Up Conditions]*. Report from Knowledge Centre No. 17: Oslo: National Knowledge Centre for Health Services at the Norwegian Institute of Public Health (NIPH); 2010.

Dahm KT, Smedslund G, Havelsrud K, Hafstad E, Reinar LM. *[Psychological Interventions for Children and Youth with Serious Somatic Illness in Primary Care]*. Report from Knowledge Centre No.10: Oslo: National Knowledge Centre for Health Services at the Norwegian Institute of Public Health (NIPH); 2014.

De Almeida AM, Lotufo Neto F. [Cognitive-behavioral therapy in prevention of depression relapses and recurrences: A review] Revisao sobre o uso da terapia cognitiva-comportamental na prevencao de recaidas e recorrencias depressivas. *Revista Brasileira de Psiquiatria* 2003;**25**:239-44.

De Carvalho MR, Nardi AE, Range B. [Comparison between cognitive, behavioral and cognitive-behavioral approaches in the treatment of panic disorder] Comparacao entre os enfoques cognitivo, comportamental e cognitivo-comportamental no tratamento do transtorno de panico. *Revista de Psiquiatria Clinica* 2008;**35**:66-73.

de Cerqueira ACR, Nardi AE. [Depression and multiple sclerosis: On overview]. *Revista Brasileira de Neurologia* 2011;**47**:11-6.

De Haan E, Huyser C, Boer F. [Obsessive-compulsive disorder in children and adolescents] De dwangstoornis bij kinderen en adolescenten. *Tijdschrift voor Psychiatrie* 2005;**47**:229-38.

De Haan L, Bakker JM. [Effectivity of individual psychotherapy in schizophrenia. A review of recent studies] Effectiviteit van individuele psychotherapie bij schizofrenie: Een overzicht van recent onderzoek. *Tijdschrift voor Psychiatrie* 2000;**42**:751-8.

Deffieux X, Billecocq S, Demoulin G, Rivain AL, Trichot C, Thubert T. [Pelvic floor rehabilitation for female urinary incontinence: Mechanisms of action] Mecanismes d'action de la reeducation perineale pour l'incontinence urinaire de la femme. *Progres en Urologie* 2013;**23**:491-501.

Ding Y. [A review of the psychological characteristics and intervention of patients with malignant tumor before and atter treatment]. *Journal of Clinical Rehabilitative Tissue Engineering Research* 2007;**11**:7951-4.

Dingemans AE, Bruna MJ, Van Furth EF. [Binge eating disorder. A review] Vreetbuistoornis: Een overzicht. *Tijdschrift voor Psychiatrie* 2001;**43**:321-31.

Dingemans A, Bruna M, van Furth E. [Binge eating disorder: A review]. *Tijdschrift voor Psychiatrie* 2001;**43**:321-31.

Doerr JP, Hajak G, Riemann D. [Pharmacotherapy of primary insomnia - Efficacy, effectiveness and safety of various therapeutic options] Pharmakotherapie der primaren insomnie - Uber die wirksamkeit, sicherheit und effektivitat verschiedener therapieoptionen. *PsychoNeuro* 2008;**34**:86-93.

dos Reis E, Camargo Novelli MMP, Fernandes Guerra RL. Intervenções realizadas com grupos de cuidadores de idosos com síndrome demencial: revisão sistemática. *Brazilian Journal of Occupational Therapy / Cadernos Brasileiros de Terapia Ocupacional* 2018;**26**:646-57.

Driessen E, Cuijpers P, Hollon SD, Van HL, Dekker JJM. [The efficacy of psychological treatments for depression: A review of recent research findings] De effectiviteit van psychologische behandelingen voor depressie: Een overzicht van nieuwe onderzoeksbevindingen. *Tijdschrift voor Psychiatrie* 2014;**56**:455-62.

Ducasse D, Denis H. [Pathological nighttime fears in children: Clinical specificities and effective therapeutics] Peurs nocturnes pathologiques de l'enfant: Particularites cliniques et therapeutiques efficaces. *Encephale* 2015;**41**:323-31.

Duchesne M, Appolinario JC, Range BP, Freitas S, Papelbaum M, Coutinho W. [Evidence of cognitive-behavioral therapy in the treatment of obese patients with binge eating disorder] Evidencias sobre a terapia cognitivo-comportamental no tratamento de obesos com transtorno da compulsao alimentar periodica. *Revista de Psiquiatria do Rio Grande do Sul* 2007;**29**:80-92.

박 수 인, 김 연 지, 오 의. 만성적인 신체질환을 가진 환자의 우울 감소를 위한 전화기반 인지행동치료의 효과: 메타분석. *Journal of Korean Academy of Psychiatric & Mental Health Nursing* 2018;**27**:227-39.

김지현, 오복자. 수면장애가 있는 중장년 환자에게 적용한 비약물적 중재의 효과: 메타분석. *Korean Journal of Adult Nursing* 2016;**28**:13-29.

Elisha D, Karny N, Styr BB. [Psychotherapy--outcome studies and guidelines for evidence-based care policy in Israel]. *Harefuah* 2011;**150**:269-74, 302.

Espanol Armengol N, Mijan De La Torre A. [Eating disorders in obesity] Trastornos de la conducta alimentaria en la obesidad. *Revista Espanola de Obesidad* 2006;**4**:317-27.

Fagiolo D, Berardelli I. [Use of cognitive-behavioural techniques in the treatment of headaches: A systematic review of the last 10-years' literature]. *Medicina Psicosomatica* 2007;**52**:117-24.

Faller H, Herschbach P. [Psychooncological interventions - How successful are they?] Psychoonkologische Interventionen - Wie erfolgreich sind sie? *Nervenheilkunde* 2011;**30**:133-7.

Fan RM, Yang L. [Progress in clinical treatment of insomnia]. *Chinese Journal of Clinical Rehabilitation* 2006;**10**:149-51.

Fernandes PA, de Carvalho MR. [Neurobiological changes after cognitive-behavioral therapy of Obsessive-Compulsive Disorder]. *Psicologia: Teoria e Pesquisa* 2016;**32**:1-9.

Fischer-Terworth C, Probst P, Glanzmann PG, Knorr CC. [Psychological interventions in dementia: An evaluative review] Psychologische interventionen bei demenziellen erkrankungen: Eine evaluative literaturstudie. *Zeitschrift fur Psychiatrie, Psychologie und Psychotherapie* 2009;**57**:195-206.

Flores-Valdez IH, Leon-Santos MP, Vera-Hernandez E, del Rocio Hernandez Pozo M. [Psychological interventions on stress management and reduction for hypertensive patients: A review of their effectiveness]. *Psychologia: Avances de la Disciplina* 2013;**7**:25-44.

Fodor KE, Bitter I. [Psychological interventions following trauma to prevent posttraumatic stress disorder: A systematic review of the literature] Pszichologiai intervenciok traumatikus esemenyek utan a poszttraumas stressz zavar megelozesere: Szisztematikus irodalmi attekintes. *Orvosi Hetilap* 2015;**156**:1321-4.

Foldes-Busque G, Marchand A, Landry P. [Early detection and treatment of panic disorder with or without agoraphobia] L'identification et traitement du trouble panique avec ou sans agoraphobie. Mise a jour. *Canadian Family Physician* 2007;**53**:1686-93.

Fond G, Franc N. [Treating specific childhood phobia in a single session? A systematic review of the literature] Traiter la phobie specifique de l'enfant en une seance ? Une revue systematique de la litterature. *Encephale* 2013;**39**:109-14.

Fritsche G, Kroner-Herwig B, Kropp P, Niederberger U, Haag G. [Psychological therapy of migraine: Systematic review] Psychologische Therapie der Migrane: Systematische Ubersicht. *Schmerz* 2013;**27**:263-74.

Fu L, Wu MB, Hu Y. [Influence of cognitive behavioural therapy on depression, medication adherence and quality of life in people living with HIV/AIDS (PLHIV): A systematic review]. *Chinese Journal of Evidence-Based Medicine* 2014;**14**:734-42.

Gärtner-Tschacher N. [The effectiveness of combined active physiotherapy and cognitive, behavioural or cognitive-behavioural therapy approaches in patients with musculoskeletal pain]. *Manuelle Therapie* 2005;**9**:11-34.

Gai X-S, Lan G-R, Liu X-P. [A meta-analytic review on treatment effects of attention deficit/hyperactivity disorder children in China]. *Acta Psychologica Sinica*;**40**:1190-6.

Gajdos P, Rigo A. [Irritable bowel syndrome: comorbid psychiatric disorders and psychological treatment options]. *Orvosi Hetilap* 2018;**159**:2115-21.

Galindo-Vazquez O, Perez-Barrientos H, Alvarado-Aguilar S, Rojas-Castillo E, Alvarez-Avitia MA, Aguilar-Ponce JL. [Cognitive behavioral therapy effects in cancer patients: A review] Efectosde la terapia cognitivo conductual en el paciente oncologico: Una revision. *Gaceta Mexicana de Oncologia* 2013;**12**:108-15.

Garcia-Perez L, Valdivia-Salas S. [Acceptance and commitment therapy for social anxiety disorder: A systematic review]. *Behavioral Psychology* 2018;**26**:379-92.

Garcia-Torres F, Alos FJ, Perez-Duenas C. [Posttraumatic stress disorder in cancer survivors: A review of the psychological treatments available] El trastorno por estrés postraumático en los supervivientes al cáncer: Una revisión de los tratamientos psicológicos disponibles. *Psicooncologia* 2015;**12**:293-301.

Garcia-Vera MP, Moreno N, Sanz J, Gutierrez S, Gesteira C, Zapardiel A*, et al.* [Efficacy and clinical utility (effectiveness) of treatments for adult victims of terrorist attacks: A systematic review]. *Behavioral Psychology / Psicologia Conductual: Revista Internacional Clinica y de la Salud* 2015;**23**:215-44.

Garcia-Vera MP, Sanz J. [Analysis of the situation of treatments for smoking cessation based on cognitive-behavioral therapy and nicotine patches]. *Psicooncologia* 2006;**3**:269-89.

Gellato C. *[Eye movement desensitization and reprocessing and cognitive-behavioral therapy in the treatment of adult's post traumatic syndrome disorder EMDR] et thérapie cognitive et comportementale dans le traitement du syndrome de stress post-traumatique chez l'adulte*: Université d'Aix-Marseille II 1969-2011; 2009.

Gomes JB, Matte BC, Vivan A, Viana A, Bortoncello CF, Salum GA*, et al.* [Cognitive behavioral therapy with family intervention for children and adolescents with obsessivecompulsive disorder: A systematic review] Terapia cognitivo-comportamental com intervencao familiar para criancas e adolescentes com transtorno o b s essivo-compulsivo: Uma revisao sistematica. *Revista de Psiquiatria do Rio Grande do Sul* 2011;**33**:121-7.

Gomez Puente JM, Martinez-Marcos M. [Overweight and obesity: effectiveness of interventions in adults]. *Enfermeria Clinica* 2018;**28**:65-74.

Gonzalez Larrabe I, Torre Mollinedo F, Telletxea Benguria S, Arizaga Maguregi A. [Update in the multidisciplinary treatment of fibromyalgia] Actualizacion en el tratamiento multidisciplinar de la fibromialgia. *Dolor* 2008;**23**:194-206.

Groschwitz RC, Plener PL. [Psychotherapeutic interventions for non-suicidal self-injury] Psychotherapie von nicht suizidalem selbstverletzendem verhalten. *Nervenheilkunde* 2013;**32**:30-6.

Gunthner A, Batra A. [Prevention of burnout by stress management] Stressmanagement als Burn-out-Prophylaxe. *Bundesgesundheitsblatt - Gesundheitsforschung - Gesundheitsschutz* 2012;**55**:183-9.

Guzman GAR, Lemus CAD, Garcia RR, Agraz FP. [Cognitive behavioral therapy for binge eating disorder: A review]. *Psiquiatria* 2005;**21**.

Haidl TK, Rosen M, Ruhrmann S, Klosterkotter J. [Social Anxiety in individuals with clinical high-risk state for psychosis] Soziale Angste im klinischen Hochrisikostadium fur Psychosen. *Fortschritte der Neurologie-Psychiatrie* 2018;**87**:284-97.

Hauser W, Bernardy K. [Psychotherapeutic procedures for fibromyalgia syndrome] Psychotherapeutische Verfahren beim Fibromyalgiesyndrom. *Zeitschrift fur Rheumatologie* 2015;**74**:584-90.

Hautzinger M, Meyer TD. [Psychotherapy for bipolar disorder: A systematic review of controlled studies] Psychotherapie bei bipolaren affektiven storungen: Ein systematischer uberblick kontrollierter interventionsstudien. *Nervenarzt* 2007;**78**:1248-60.

Hautzinger M, Wetzel H, Scheurich A, Mainz Univ, Saarland Univ, Rostock Univ*, et al. [Serotonin 1A-agonist and cognitive behavior therapy in relapse prevention of alcoholics. Evaluation of efficacy of different treatment modalities and their combination] Serotonin_1_A-Agonisten und Verhaltenstherapie in der Alkoholrueckfallprophylaxe - Vergleichende Evaluation der Wirksamkeit unterschiedlicher Therapiemodalitaeten und ihrer Kombination Schlussbericht*; 2001.

Hautzinger M. [Behavioural therapy with affective and neurological disorders in the elderly]. *Verhaltenstherapie & Verhaltensmedizin* 2002;**23**:195-212.

Heckrath VC, Dohmen P. [On the empirical foundation of the highly significant superiority of the cognitive-behavior compared with the psychoanalytically oriented psychotherapies] Zu der empirischen basis der hochsignifikanten uberlegenheit' der kognitiv-behavioralen gegenuber den psychoanalytischen psychotherapieverfahren. *Zeitschrift fur Psychosomatische Medizin und Psychoanalyse* 1997;**43**:179-201.

Hendriks GJ, Keijsers GPJ, Kampman M, Verbraak MJPM, Broekman TG, Hoogduin CAL*, et al.* [Treatment of anxiety disorders in the elderly] Behandeling van angststoornissen bij ouderen. *Tijdschrift voor Psychiatrie* 2011;**53**:589-95.

Herr L, Mingebach T, Becker K, Christiansen H, Kamp-Becker I. [A systematic review of the effectiveness of parent-based interventions for children aged two to twelve years]. *Kindheit und Entwicklung: Zeitschrift fur Klinische Kinderpsychologie* 2015;**24**:6-19.

Hess M, Wirtz S, Allroggen M, Scheithauer H. [Intervention and Therapy for Perpetrators and Victims of Bullying: A Systematic Review]. *Praxis der Kinderpsychologie und Kinderpsychiatrie* 2017;**66**:740-55.

Hilbert A, Brahler E. [Interpersonal psychotherapy for eating disorders: A systematic and practical review]. *Verhaltenstherapie* 2012;**22**:149-57.

Hirjak D, Bechdolf A, Thomann PA, Thiemann U, Wolf RC. [Prevention of psychosis] Indizierte Pravention psychotischer Storungen: Behandlung von Personen mit erhohtem Psychoserisiko. *Nervenheilkunde* 2012;**31**:923-32.

Hua Y, Dai J. [Studies on occupational stress intervention in workplaces abroad: a systematic review]. *Zhonghua Lao Dong Wei Sheng Zhi Ye Bing Za Zhi* 2015;**33**:759-64.

Huang F-F, Li Z-J, Han H-Y, Xiong H-F, Ma Y. [Cognitive behavioral therapy combined with pharmacotherapy for obsessive compulsive disorder: A meta-analysis]. *Chinese Mental Health Journal* 2013;**27**:643-9.

한수연, 황지혜, 김초희, 장혜영, 방경숙. 소아암 환자의 형제자매 중재에 관한 연구논문의 체계적 문헌고찰. *Child Health Nursing Research* 2017;**23**:394-404.

Isolan L, Pheula G, Manfro GG. [Treatment of social anxiety disorder in children and adolescents] Tratamento do transtorno de ansiedade social em criancas e adolescentes. *Revista de Psiquiatria Clinica* 2007;**34**:125-32.

Jakle C, Basler HD. [Change of cognition in psychological pain therapy: A meta-analysis of the cognitive-behavioral mode] Veranderung von kognitionen in der psychologischen schmerztherapie - Eine metaanalyse zum kognitiv-behavioralen modell. *Zeitschrift fur klinische Psychologie und Psychotherapie* 2000;**29**:127-39.

Jank R, Pieh C. [Efficacy and evidence base of group psychotherapy for depressive disorders]. *Psychotherapie Forum* 2016;**21**:62-71.

Jelonkiewicz I. [Psychotherapy of gambling - How to effectively treat gamblers? The effectivenes of applied therapeutic methods] Psychoterapia hazardu - Jak skutecznie leczyc hazardzistow? Efektywnosc stosowanych metod terapeutycznych. *Psychoterapia* 2014:31-41.

Juge C, Tubert-Jeannin S. [Effects of hypnosis in dental care] Effets de l'hypnose lors des soins dentaires. *Presse Medicale* 2013;**42**:e114-e24.

Kamaradova D, Prasko J, Grambal A, Diveky T, Latalova K, Silhan P. [Pharmacoresistance in patients with panic disorder] Farmakorezistence u pacientu s panickou poruchou. *Psychiatrie* 2012;**16**:150-6.

Kapfhammer H-P. [The relationship between depression, anxiety and heart disease-A psychosomatic challenge]. *Psychiatria Danubina* 2011;**23**:412-25.

Kapusta ND, Fegert JM, Haring C, Plener PL. [Psychotherapeutic interventions for suicidal adolescents]. *Psychotherapeut* 2014;**59**:16-23.

Karjalainen P, Santalahti P, Sihvo S. [Are programs supporting parenthood skills effective in the prevention and reduction of conduct disorders and problems of childhood?]. *Duodecim* 2016;**132**:967-74.

Kim JH. [A meta-analysis of effects of job stress management interventions (SMIs)]. *Taehan Kanho Hakhoe chi* 2007;**37**:529-39.

Kim Y, Park I, Park JS. [Meta-analysis of effects on adolescent smoking cessation programs in Korea]. *Taehan Kanho Hakhoe chi* 2008;**38**:204-16.

Kirk I, Leiknes KA, Laru L, Hammerstrom KT, Bramness JG, Grawe RW*, et al. [Dual Diagnoses - Severe Mental Illness and Substance Use Disorder. Part 2 - Effect of Psychosocial Interventions]*. Report from Knowledge Centre No. 25: Oslo: National Knowledge Centre for Health Services at the Norwegian Institute of Public Health (NIPH); 2008.

Kirste U, Haugstad GK, Leganger S, Blomhoff S, Malt UF. [Chronic pelvic pain in women] Kroniske bekkensmerter hos kvinner. *Tidsskrift for den Norske Laegeforening* 2002;**122**:1223-7.

Kollner V, Bernardy K, Greiner W, Krumbein L, Lucius H, Offenbacher M*, et al.* [Psychotherapy and psychological procedures for fibromyalgia syndrome: Updated guidelines 2017 and overview of systematic review articles] Psychotherapie und psychologische Verfahren beim Fibromyalgiesyndrom: Aktualisierte Leitlinie 2017 und Ubersicht von systematischen Ubersichtsarbeiten. *Schmerz* 2017;**31**:266-73.

Kollner V, Hauser W, Klimczyk K, Kuhn-Becker H, Settan M, Weigl M*, et al.* [Psychotherapy for patients with fibromyalgia syndrome. Systematic review, meta-analysis and guideline] Psychotherapie von patienten mit fibromyalgiesyndrom: Systematische ubersicht, metaanalyse und leitlinie. *Schmerz* 2012;**26**:291-6.

Kornor H, Winje D, Ekeberg O, Johansen K, Weisaeth L, Ormstad SS*, et al. [Psychosocial Interventions After Crises and Accidents]*. Report from Knowledge Centre No. 14: Oslo: National Knowledge Centre for Health Services at the Norwegian Institute of Public Health (NIPH); 2007.

Kraft S, Schepker R, Goldbeck L, Fegert J. [Treatment of posttraumatic stress disorder in children and adolescents--A review of treatment outcome studies]. *Nervenheilkunde: Zeitschrift fur interdisziplinaere Fortbildung* 2006;**25**:709-16.

Krause K, Gurtler D, Bischof G, Rumpf H-J, Lucht M, John U*, et al.* [Computer-based interventions to reduce depressive symptoms-An overview of available and evidence-based programs]. *Zeitschrift fur Psychiatrie, Psychologie und Psychotherapie* 2016;**64**:121-31.

Kreissl S, Burkle C, Ruffer U, Mehnert A, Borchmann P. [Cancer-related fatigue in Hodgkin's lymphoma] Tumorassoziierte Fatigue beim Hodgkin-Lymphom. *Onkologe* 2018;**24**:329-34.

Kremberg E, Mitte K. [Cognitive-behavioral treatment of social phobia in children and adolescents: A review] Kognitiv-behaviorale und behaviorale interventionen der sozialen phobie im kindes- und jugendalter: Ein uberblick zur wirksamkeit. *Zeitschrift fur klinische Psychologie und Psychotherapie* 2005;**34**:196-204.

Kroese MEA, de Vet HCW, Scholten RJP. [Review of research on the effectiveness of regular physical therapy for chronic benign pain]. *Nederlands Tijdschrift Voor Fysiotherapie* 2002;**112**:42-9.

López RNA, Girona FG. [Cognitive-behavioral therapy in the treatment of generalized anxiety]. *Metas de Enfermería* 2011;**14**:70-3.

Laakmann M, Petermann U, Petermann F. [Parental participation in the context of anxiety treatment for children: A systematic review]. *Kindheit und Entwicklung: Zeitschrift fur Klinische Kinderpsychologie* 2017;**26**:77-92.

Lagrue G, Le Foll B, Melihan-Cheinin P, Rostoker G, Ades J, de Beaurepaire R*, et al.* [Drug and Non-Drug Treatment Strategies to Assist Smoking Cessation] Les Strategies Therapeutiques Medicamenteuses et Non Medicamenteuses de l'Aide a l'Arret du Tabac. *Therapie* 2003;**58**:479-97.

Landuyt G, Dierckx B, De Nus PFA, Dieleman GC. [Treatment options for paediatric trichotillomania] Behandelopties bij pediatrische trichotillomanie. *Tijdschrift voor Psychiatrie* 2016;**58**:463-70.

Lang T, Helbig-Lang S, Petermann F. [Which interventions are crucial in CBT for panic disorder and agoraphobia? - A systematic review] Was wirkt in der kognitiven verhaltenstherapie der panikstorung mit agoraphobie? - Ein systematisches review. *Zeitschrift fur Psychiatrie, Psychologie und Psychotherapie* 2009;**57**:161-75.

Larsson B. [Cognitive outcome of childhood depression using cognitive behavior therapy] Kognitiv beteendeterapi ger goda resultat vid depression hos barn. *Lakartidningen* 2002;**99**:1810-2, 5-9.

Larun L, Dalsbo TK, Hafstad E, Reinar LM. *[Effects of Interventions for Prevention of Sick Leave and Disability for Health Personnel]*. Raport from Knowledge Centre No. 2: Oslo: Knowledge Centre for Health Services at the Norwegian Institute of Public Health (NIPH); 2014.

Lefio LA, Villarroel SR, Rebolledo C, Zamorano P, Rivas K. [Effective interventions in the problematic use of alcohol and other drugs]. *Pan American Journal of Public Health* 2013;**34**:257-66.

Leibetseder M, Laireiter AR, Vierhauser M, Hittenberger B. [Efficacy and effectiveness of psychological and psycho-pharmacological treatments in pathological gambling - A meta-analysis] Die wirksamkeit psychologischer und psychopharmakologischer interventionen bei pathologischem gluckspiel - Eine metaanalyse. *Sucht* 2011;**57**:275-85.

Leichsenring F, Leibing E. [How effective are psychoanalytic-oriented therapy and behavioral therapy by personality disorders?] Wie wirksam sind psychoanalytisch orientierte therapie und verhaltenstherapie bei personlichkeitsstorungen? *Forum der Psychoanalyse* 2003;**19**:378-85.

Leite CEP, Vicentini HC, dos Santos Neves J, Torres AR. [Emetophobia: A critical review about an understudied disorder]. *Jornal Brasileiro de Psiquiatria* 2011;**60**:123-30.

Levitan MN, Chagas MH, Crippa JA, Manfro GG, Hetem LA, Andrada NC, *et al.* [Guidelines of the Brazilian Medical Association for the treatment of social anxiety disorder]. *Revista Brasileira de Psiquiatria* 2011;**33**:292-302.

Li JY, Ge LJ. [Epidemiologic characteristics and therapy of social anxiety]. *Journal of Clinical Rehabilitative Tissue Engineering Research* 2007;**11**:10644-7.

Li J, Liu L, Li MQ, Zhang WW, Si Y. [Evidence-based evaluation of therapeutic measures for sleep disorders]. *Chinese Journal of Contemporary Neurology and Neurosurgery* 2013;**13**:398-404.

Lin CW, Haas M, Maher CG, MacHado LA, Van Tulder MW, Joos S. [Cost-effectiveness of guideline-endorsed treatments for low back pain: A systematic review]. *Deutsche Zeitschrift fur Akupunktur* 2011;**54**:26-7.

Lin W-C. [Cognitive behavioral treatment for insomnia on cancer patients: A systematic review]. *Chinese Journal of Psychology*;**52**:173-88.

Lincoln TM, Suttner C, Nestoriuc Y. [Effects of cognitive interventions for schizophrenia: A meta-analysis]. *Psychologische Rundschau* 2008;**59**:217-32.

Liu Q, Wang H, Wang W, Zhang F, Li J, Song WF, *et al.* [Interventions on preventing and treating mental health problems of involuntary migrants: A systematic review]. *Chinese Journal of Evidence-Based Medicine* 2009;**9**:929-37.

Liu Y-C, Lan Y-L, Liu C-J, Chou Y-J. [The 10-year meta-analysis of cognitive-behavioral group therapy for depressive symptoms]. *Chinese Journal of Psychology*;**50**:383-402.

Loeber S, Dinter C, Mann K. [Psychotherapeutic treatment for addiction and comorbid depression] Psychotherapie von patienten mit depression und abha ngigkeitserkrankung. *Sucht* 2011;**57**:373-81.

Lopes LO, Cachioni M. [Psychoeducational intervention for caregivers of elderly with dementia: A systematic review] Intervencoes psicoeducacionais para cuidadores de idosos com demencia: Uma revisao sistematica. *Jornal Brasileiro de Psiquiatria* 2012;**61**:252-61.

Mao Z-H, Zhao X-D. [Comprehensive analysis of articles on counseling and psychotherapy researches (2000-2009) in Chinese Mental Health Journal]. *Chinese Mental Health Journal* 2011;**25**:254-8.

Marquez S, de la Vega R. [Exercise addiction: an emergent behavioral disorder]. *Nutricion Hospitalaria* 2015;**31**:2384-91.

Martin A, Gaab J. [Chronic fatigue syndrome. Evidenced-based psychotherapy for chronic medically unexplained fatigue]. *Psychotherapeut* 2011;**56**:231-8.

Martinez M, Miro E, Sanchez AI. [Global clinical benefits of cognitive behavioral therapy for insomnia and mindfulness-based therapy applied to fibromyalgia: Systematic review and meta-analysis]. *Behavioral Psychology / Psicologia Conductual: Revista Internacional Clinica y de la Salud* 2016;**24**:459-80.

Martinez M, Sanchez AI, Martinez MP, Miro E. [Psychological treatment in patients with systemic lupus erythematosus: A systematic review] Tratamiento psicologico en pacientes lupus eritematoso sistemico: Una revision sistematica. *Terapia Psicologica* 2016;**34**:167-81.

Mate O, Somogyi K, Miklosi M. [Cognitive conceptualization of adult attention deficit hyperactivity disorder: a systematic review]. *Psychiatria Hungarica* 2015;**30**:68-77.

Mazoni CG, Fernandes S, Pierozan PS, Moreira T, Freese L, Ferigolo M, *et al.* [Efficacy of pharmacological and no-pharmacological treatments for smoking cessation]. *Estudos de Psicologia* 2008;**13**:133-40.

Mehlum L, Dieserud G, Ekeberg O, Groholt B, Mellesdal L, Walby F, *et al.* *[Effects of Interventions for Prevention of Sick Leave and Disability for Health Personnel]*. Report from Knowledge Centre No. 2: Oslo: National Knowledge Centre for Health Services at the Norwegian Institute of Public Health (NIPH); 2006.

Meister R, Jansen A, Berger M, Baumeister H, Bschor T, Harfst T*, et al.* [Psychotherapy of depressive disorders. Procedures, evidence and perspectives]. *Der Nervenarzt* 2018;**89**:241-51.

Mendez Carrillo FX, Moreno PJ, Sanchez-Meca J, Olivares J, Espada JP. [Effectiveness of psychological treatment for child and adolescent depression: A qualitative review of two decades of research] Eficacia del tratamiento psicologico de la depresion infantil y adolescente: Una revision cualitativa y cuantitativa de dos decadas de investigacion. *Psicologia Conductual* 2000;**8**:487-510.

Mendez X, Rosa AI, Montoya M, Espada JP, Olivares J, Sanchez-Meca J. [The psychological treatment of childhood and adolescent depression: Evidence or promise?] Tratamiento psicologico de la depresion infantil y adolescente: Evidencia o promesa? *Psicologia Conductual* 2002;**10**:563-80.

Meyer TD, Hautzinger M. [Cognitive behavioral therapy in addition to pharmacotherapy for manic depressive disorders. Empirical results] Kognitive verhaltenstherapie als erganzung der pharmakotherapie manisch-depressiver storungen. Wie sieht die empirie aus? *Nervenarzt* 2002;**73**:620-8.

Mikulska J, Brynska A. [Obsessive-compulsive disorder - Methods of treatment and the factors influencing their efficacy] Zaburzenie obsesyjno-kompulsyjne - Metody leczenia i czynniki determinujace ich skutecznosc. *Psychoterapia* 2004:55-67.

Minelli A, Zambello F, Vaona A. [Effectiveness of cognitive-associated with behavioral therapy psychopharmacological depression. Literature review meta-analyses] Efficacia della psicoterapia cognitivo-comportamentale associata a terapia psicofarmacologica nella depressione. Revisione della letteratura metanalitica. *Rivista di Psichiatria* 2011;**46**:18-23.

Mora Moscoso R, Guzman Ruiz M, Soriano Perez AM, De Alba-Moreno R. [Treatment of central neuropathic pain; Future analgesic therapies: Systematic review] Tratamiento del dolor neuropatico central; futuras terapias analgesicas. Revision sistematica. *Revista de la Sociedad Espanola del Dolor* 2014;**21**:270-80.

Moreno Gil PJ, Carrillo F, Sanchez Meca J. [Effectiveness of cognitive-behavioural treatment in social phobia: A meta-analytic review]. *Psicothema* 2000;**12**:346-52.

Mortan Sevi O, Tekinsav Sutcu S. [Cognitive-behavioral group treatment for schizophrenia and other psychotic disorders- a systematic review] Sizofreni ve diger psikotik bozukluklarda bilissel-davranisci grup terapisi-sistematik bir gozden gecirme. *Turk Psikiyatri Dergisi* 2012;**23**:206-18.

Muller A, De Zwaan M. [Compulsive buying: A review of the current literature] Pathologisches kaufen - Ein uberblick uber den stand der literatur. *Sucht* 2008;**54**:271-9.

Muller H, Wiessmann T, Bechdolf A. [Interventions in People at Risk of Developing First Episode Psychosis: A Survey of Current Randomised Controlled Studies] Interventionen bei Personen mit erhohtem Psychoserisiko: Eine aktuelle Ubersicht uber randomisiert kontrollierte Studien. *Fortschritte der Neurologie* 2012;**Psychiatrie. 80**:570-9.

Mululo SCC, De Menezes GB, Fontenelle L, Versiani M. [Cognitive behavioral-therapies, cognitive therapies and behavioral strategies for the treatment of social anxiety disorder] Terapias cognitivo-comportamentais, terapias cognitivas e tecnicas comportamentais para o transtorno de ansiedade social. *Revista de Psiquiatria Clinica* 2009;**36**:221-8.

Myrhaug HT, Strom V, Hafstad E, Kirkehei I, Reinar LM. *[The Effect of Hydrotherapy for Persons with Musculoskeletal Disorders]*. Report from Knowledge Centre No. 11: Oslo: National Knowledge Centre for Health Services at the Norwegian Institute of Public Health (NIPH); 2015.

Napierala M. [Cognitive-behavioural therapy in the treatment of bipolar affective disorders] Terapia poznawczo-behawioralna w leczeniu zaburzen afektywnych dwubiegunowych. *Neuropsychiatria i Neuropsychologia* 2017;**12**:118-25.

Nardi B, Laurenzi S, Nicolo M, Bellantuono C. [Is the cognitive-behavioural therapy an effective strategy also in the prevention of post partum depression? A critical review]. *Riv Psichiatr* 2012;**47**:205-13.

Nauta KJ, Batelaan NM, Van Balkom AJLM. [Obsessive-compulsive disorder from a family perspective; implications for treatment and research] De obsessieve-compulsieve stoornis vanuit gezinsperspectief; implicaties voor behandeling en onderzoek. *Tijdschrift voor Psychiatrie* 2012;**54**:439-48.

Navarro-Mateu F, Garriga-Puerto A, Sanchez-Sanchez JA. [Tree decision analysis of the therapeutic alternatives for panic disorders in primary care] Analisis de las alternativas terapeuticas del trastorno de panico en atencion primaria mediante un arbol de decision. *Atencion Primaria* 2010;**42**:86-94.

Nedate K, Ichii M, Sekiguchi Y, Miyamae Y, et al. [Is cognitive behavior therapy effective? From the viewpoint of meta-analysis and individual differences]. *Japanese Journal of Counseling Science* 1995;**28**:87-103.

Nilges P. [Systematic review and meta-analysis of randomized controlled trials of cognitive behaviour therapy and behaviour therapy for chronic pain adults, excluding headache] Effektivitat (kognitiver) verhaltenstherapie bei chronischem schmerz: Eine systematische ubersicht und metaanalyse ohne berucksichtigung von kopfschmerz. *Schmerz* 2001;**15**:69-70.

Oh PJ, Han SJ. [Meta-analysis of psychosocial interventions to reduce pain in patients with cancer]. *Journal of Korean Academy of Nursing* 2013;**43**:658-68.

Oliva VHS, Vianna A, Lotufo Neto F. [Cinematherapy as psychotherapeutic intervention: Characteristics, applications and identification of cognitive-behavior techniques] Cinematerapia como intervencao psicoterapica: Caracteristicas, aplicacoes e identificacao de tecnicas cognitivo-comportamentais. *Revista de Psiquiatria Clinica* 2010;**37**:138-44.

Olivares J, Rosa AI, Sanchez Meca J. [Meta-analysis of the effectiveness of coping skills in clinical and health problems in Spain]. *Anuario de Psicologia* 2000;**31**:43-61.

Park WJ, Park SJ, Hwang SD. [Effects of Cognitive Behavioral Therapy on Attention Deficit Hyperactivity Disorder among School-aged Children in Korea: A Meta-Analysis]. *Journal of Korean Academy of Nursing* 2015;**45**:169-82.

Pelissolo A. [Hypnosis for anxiety and phobic disorders: A review of clinical studies] L'hypnose dans les troubles anxieux et phobiques : Revue des etudes cliniques. *Presse Medicale* 2016;**45**:284-90.

Pitschel-Walz G, Bauml J. [Efficacy of psychotherapy for patients with schizophrenia - Results of meta-analyses] Psychotherapie bei schizophrenien - Ergebnisse von metaanalysen zur wirksamkeit. *Psychiatrische Praxis, Supplement* 2007;**34**:S28-S32.

Pok Ja O, Eun Ai L. [Cognitive Behavioral Therapy for Psychological Distress, Self Care and Quality of Life in Patients with Cancer: A Meta-analysis]. *Korean Journal of Adult Nursing* 2013;**25**:377-88.

Prados G, Miro E. [Fibromyalgia and sleep: A review] Fibromialgia y sueno: Una revision. *Revista de Neurologia* 2012;**54**:227-40.

Prasko JP, Ociskova M, Kamaradova D, Latalova K, Vrbova K, Sedlackova Z, *et al.* [Cognitive-behavioral therapy and dialectical-behavioral therapy in suicidal patients]. 2014;**18**:8-17.

Prazeres AM, De Souza WF, Fontenelle LF. [Cognitive-behavior therapy for obsessive-compulsive disorder: A systematic review of the last decade] Terapias de base cognitivo-comportamental do transtorno obsessivo-compulsivo: Revisao sistematica da ultima decada. *Revista Brasileira de Psiquiatria* 2007;**29**:262-70.

Puschber B, Born A, Giesler A, Helm H, Becker T, Angermeyer MC. [Effects of Interventions to Improve Compliance with Antipsychotic Medication in People Suffering from Schizophrenia Results of Recent Reviews]. *Psychiatrische Praxis* 2005;**32**:62-7.

Puschner B, Vauth R, Jacobi F, Becker T. [Evidence basis of psychotherapy for schizophrenia patients in Germany]. *Nervenarzt* 2006;**77**:1301-2, 4.

Quintero MF, Finck C. [Effective psychological intervention for breast cancer patients in latin america and spain: A systematic review] Intervenciones psicologicas eficaces en pacientes con cancer de mama en latinoamerica y espana: Una revision sistematica. *Psicooncologia* 2018;**15**:49-64.

Raffin AL, Ferrao YA, De Souza FP, Cordioli AV. [Outcome predictor factors in the treatment of obsessive-compulsive disorder using behavior and cognitive-behavior therapies: A systematic review] Fatores preditores de resultados no tratamento do transtorno obsessivo-compulsivo com as terapias comportamental e cognitivo-comportamental: Uma revisao sistematica. *Revista de Psiquiatria do Rio Grande do Sul* 2008;**30**.

Rafihi-Ferreira RE, Soares MRZ. [Insomnia in patients with breast cancer]. *Estudos de Psicologia* 2012;**29**:597-607.

Ramos SAT, del Carmen Lara Munoz M. [Non-pharmacological interventions in primary insomnia: Controlled clinical trial findings (1998-2008)]. *Revista Colombiana de Psiquiatria* 2011;**40**:310-35.

Reinecke H, Sorgatz H, German Society for the Study of P. [S3 guideline LONTS. Long-term administration of opioids for non-tumor pain]. *Der Schmerz* 2009;**23**:440-7.

Riehle M, Pillny M, Lincoln TM. [Are the Negative Symptoms of Schizophrenia Treatable at All? A Systematic Review on Efficacy Studies for Targeted Psychological Interventions for Negative Symptoms] Ist Negativsymptomatik bei Schizophrenie uberhaupt behandelbar? Ein systematisches Literaturreview zur Wirksamkeit psychotherapeutischer Interventionen fur Negativsymptomatik. *Verhaltenstherapie* 2017;**27**:199-208.

Rodrigues MGA, Krauss-Silva L, Martins ACM. [Meta-analysis of clinical trials on family intervention in schizophrenia] Meta-analise de ensaios clinicos de intervencao familiar na condicao esquizofrenia. *Cadernos de Saude Publica* 2008;**24**:2203-18.

Rojas E, Real T, Garcia-Silberman S, Medina-Mora ME. [Systematic review of addiction treatment in Mexico]. *Salud Mental* 2011;**34**:351-65.

Ruhmland M, Margraf J. [Efficacy of psychological treatments for generalized anxiety disorder and social phobia] Effektivitat psychologischer therapien von generalisierter angststorung und sozialer phobie: Meta-analysen auf storungsebene. *Verhaltenstherapie* 2001;**11**:27-40.

Ruhmland M, Margraf J. [Efficacy of psychological treatments for panic and agoraphobia] Effektivitat psychologischer therapien von panik und agoraphobie: Meta-analysen auf storungsebene. *Verhaltenstherapie* 2001;**11**:41-53.

Ruhmland M, Margraf J. [Efficacy of psychological treatments for specific phobia and obsessive compulsive disorder] Effektivitat psychologischer therapien von spezifischer phobie und zwangsstorung: Meta-analysen auf storungsebene. *Verhaltenstherapie* 2001;**11**:14-26.

Sánchez Ocón MT, Pérez Morente MÁ, Mingorance Ruiz MV, Pérez Robles MA, Muñoz de la Fuente JM. Revisión de las intervenciones farmacológicas y complementarias en el manejo del Síndrome de Fibromialgia. *Enfermería Comunitaria* 2013;**9**:1-9.

Santacruz I, Orgiles M, Rosa AI, Sanchez-Meca J, Mendez X, Olivares J. [Generalized anxiety, separation anxiety and school phobia: the predominance of cognitive-behavioural therapy]. *Psicologia Conductual* 2002;**10**:503-21.

Santandrea S, Boschi M, Vanti C. [Effectiveness of cognitive behavioural therapy in spinal pain: a systematic review]. *Scienza Riabilitativa* 2011;**13**:5-23.

Schmid G, Henningsen P, Dieterich M, Sattel H, Lahmann C. [Psychotherapy in dizziness-a systematic review]. *PDP Psychodynamische Psychotherapie: Forum der tiefenpsychologisch fundierten Psychotherapie* 2011;**10**:25-40.

Sikorski C, Luppa M, Kersting A, Konig HH, Riedel-Heller SG. [Computer-aided cognitive behavioral therapy for depression: A systematic review of the literature] Effektivitat computer- und internetgestutzter

kognitiver Verhaltenstherapie bei Depression: Ein systematischer Literaturuberblick. *Psychiatrische Praxis* 2011;**38**:61-8.

Siles J, Tarquinio C. [Psychosexual consequences and their treatments in the field of cancer: A systematic review of psychotherapeutics interventions] Les consequences psychosexuelles et leurs traitements dans le champ du cancer : une revue systematique d'interventions psychotherapeutiques. *Sexologies* 2017;**26**:87-95.

Silva L, Morgado P. [Koro syndrome associated with obsessive-compulsive disorder: Clinical case and brief review]. *Jornal Brasileiro de Psiquiatria* 2018;**67**:135-9.

Slotema C, Blom J, Sommer I. [Treatment strategies for auditory verbal hallucinations]. *Tijdschrift voor Psychiatrie* 2014;**56**:247-55.

Smits CT, Van Der Gaag M. [Cognitive behavioural therapy for schizophrenia] Cognitieve gedragstherapie bij schizofrenie. *Tijdschrift voor Psychiatrie* 2010;**52**:99-109.

Soulia V, Giannakopoulou M. [Non-invasive and Non-pharmacological Methods for the Alleviation of Neuropathic Pain]. *Nosileftiki* 2011;**50**:147-62.

Soussana M, Sunyer B, Pry R, Baghdadli A. [Anxiety in children and adolescents with pervasive developmental disorder without mental retardation: review of literature]. *Encephale* 2012;**38**:16-24.

Spijker A, van Zaane J, Koenders M, Hoekstra R, Kupka R. [Bipolar disorder and alcohol use disorder: Practical recommendations for treatment, based on a literature review]. *Tijdschrift voor Psychiatrie* 2018;**60**:87-95.

Staccini L. [Psychological treatment of female sexual dysfunction: a critical review of the literature]. *Rivista di Psichiatria* 2015;**50**:265-73.

Stolarska-Werynska U, Biedron A, Kacinski M. [The links between neuropsychology and neurophysiology]. *Przeglad Lekarski* 2016;**73**:187-90.

Svec J, Svec P, Bencova V, Krcmery V. [Anxio-depressive syndrome - Biopsychosocial model of Supportive Care] Anxio-depresivny syndrom v onkologii - Biopsychosocialny model suportivnej terapie. *Klinicka Onkologie* 2015;**28**:177-82.

SBU. *[Treatment for Binge Eating Disorder]*. SBU report no 248: Stockholm: Swedish Agency for Health Technology Assessment and Assessment of Social Services; 2016.

Takano A, Miyamoto Y, Matsumoto T. [A review about new approaches using the Internet and computer technology for people with drug use disorder]. *Nihon Arukoru Yakubutsu Igakkai Zasshi* 2015;**50**:19-34.

Tian L, Cao XY. [Effectiveness of cognitive behavioral therapy treating insomnia in patients with breast cancer: A systematic review]. *Chinese Journal of Evidence-Based Medicine* 2013;**13**:70-7.

Tomayo JM, Rosales-Barrera JI, Villasenor-Bayardon SJ, Rojas-Malpica C. [Literature review on management of treatment-resistant depression]. *Salud Mental* 2011;**34**:257-66.

Toorn SL, Ferdinand RF. [Anxiety disorders in children: which type of psychosocial treatment has proved effective] Angststoornissen bij kinderen: welke psychosociale behandeling is bewezen effectief *Tijdschrift voor Psychiatrie* 2004;**46**:167-77.

Turkcapar A, Turkcapar M. [Diagnosis and treatment of premenstrual syndrome and premenstrual dysphoric disorder: A review]. *Klinik Psikiyatri Dergisi: The Journal of Clinical Psychiatry* 2011;**14**:241-53.

Tutus D, Plener PL, Niemitz M. [Quality criteria of internet-based cognitive-behavioral interventions for children and adolescents and their parents - A systematic review]. *Zeitschrift fur Kinder-und Jugendpsychiatrie und Psychotherapie* 2018:1-17.

Valiune D. [Effectiveness of the cognitive behaviour therapy based interventions to children's and adolescents' anger and aggressive behaviour. Systematic analysis]. *International Journal of Psychology: A Biopsychosocial Approach / Tarptautinis psichilogijos zurnalas: Biopsichosocialinis poziuris* 2014;**15**:111-31.

van Alphen SP. [Prevalence, diagnosis and treatment of personality disorders in older adults] Prevalentie, diagnostiek en behandeling van persoonlijkheidsstoornissen bij ouderen. Een literatuuroverzicht. *Tijdschrift voor gerontologie en geriatrie* 2010;**41**:79-86.

van den Berg B, Knoppert-Van der Klein E, van Zaane J. [Psychotherapeutic treatment options for bipolar disorders. A review of randomized controlled studies]. *Tijdschrift voor Psychiatrie* 2006;**48**:905-31.

van der Veen W, Renes J, Kupka R, Regeer E. [The effects of pharmacological and psychotherapeutic treatment of comorbid anxiety disorders in patients with bipolar disorder]. *Tijdschrift voor Psychiatrie* 2018;**60**:388-96.

Vancampfort D, Vanderlinden J, Pieters G, De Herdt A, Schueremans A, Adriaens A*, et al.* [The importance of movement-directed interventions in the multidisciplinary treatment of binge eating disorder: An overview] Bewegingsgerichte interventies in de multidisciplinaire behandeling van eetbuistoornissen; een literatuuronderzoek. *Tijdschrift voor Psychiatrie* 2012;**54**:719-30.

Vazquez FL. [Psychological and pharmacological smoking cessation approaches for smokers with depression disorders]. *Clinica y Salud* 2005;**16**:269-89.

Verhagen AP, Damen L, Berger MY, Lenssinck ML, Passchier J, Kroes BW. [Treatment of tension type headache: paracetamol and NSAIDs work: a systematic review] Behandeling van spanningshoofdpijn: paracetamol en NSAID's werken: een systematische review. *Nederlands Tijdschrift voor Geneeskunde* 2010;**154**:A1924.

Verhagen A, Damen L, Bruijn J, Berger M, Passchier J, Koes B. [Effectiveness of interventions in children with migraine] Effectiviteit van interventies bij kinderen met migraine. *Huisarts en Wetenschap* 2006;**49**:123-9.

Vetere G, Rodriguez Biglieri R. [Empirical validation of theoretical models and cognitive behavioral treatments for Generalized Anxiety Disorder] Validacion empirica de modelos teoricos y tratamientos cognitivo - conductuales para el Trastorno de Ansiedad Generalizada. *Vertex (Buenos Aires, Argentina)* 2005;**16**:170-5.

Vist GE, Reinar LM, Straumann GH, Wisting L. *[Treatment of Persons who Suffer from Both an Eating Disorder and Diabetes]*. Report from Knowledge Centre No. 18: Oslo: National Knowledge Centre for Health Services at the Norwegian Institute of Public Health (NIPH); 2015.

Voderholzer U, Barton B. [Long-term effects of psychotherapy for non-chronic depressive disorder: A systematic review of studies in comparison with pharmacotherapy] Langfristige Wirkung von Psychotherapie bei nichtchronischen Depressionen: Ein systematisches Review von Studien im Vergleich mit Pharmakotherapie. *Verhaltenstherapie* 2016;**26**:108-15.

Vogel-Mergaerts SML, Liessens D. [Panic disorder during pregnancy] Paniekstoornis tijdens zwangerschap. *Tijdschrift voor Psychiatrie* 2002;**44**:687-92.

von Hofe I, Latza U, Lonnfors S, Muckelbauer R. [Online Health Services for the Prevention of Stress-associated Psychological Impairments at the Workplace]. *Gesundheitswesen* 2017;**79**:144-52.
Vulink NCC, Denys D. [Body dysmorphic disorder: An overview] Body dysmorphic disorder (stoornis in de lichaamsbeleving): Een overzicht. *Tijdschrift voor Psychiatrie* 2005;**47**:21-7.

Walter M, Dursteler KM, Petitjean SA, Wiesbeck GA, Euler S, Sollberger D*, et al.* [Psychosocial Treatment of Addictive Disorders--An Overview of Psychotherapeutic Options and their Efficacy]. *Fortschritte der Neurologie-Psychiatrie* 2015;**83**:201-10.

Wang F, Wu HM, Huang CQ, Lun ZC, Dong BR. [Psychotherapy for depression in older patients: A systematic review]. *Chinese Journal of Evidence-Based Medicine* 2008;**8**:1079-85.

Willemse Y, Trijsburg R. [Cognitive behavior therapy and interpersonal psychotherapy. An analysis of factors leading to a successful treatment-outcome]. *Tijdschrift voor Psychiatrie* 2005;**47**:593-602.

Yablonsky PK, Sukhovskaya OA. [Smoking influence on the outcomes and complications of coronary bypass surgery]. *Russian Journal of Cardiology* 2018;**153**:66-71.

Yang ZS, Chen JB. [Psychotheraphy for tumor patients]. *Chinese Journal of Clinical Rehabilitation* 2005;**9**:77-9.

Ye YY, Jiang XJ, Liu J, Li XJ, Liu YZ, Lang Y*, et al.* [Efficacy of telephone-delivered cognitive behavioral therapy for insomnia: A meta-analysis]. *Chinese Journal of Evidence-Based Medicine* 2016;**16**:334-40.

Yu C, Liu XJ, Huang J, Zhou YT. [Effectiveness of psychological intervention on post-stoke depression: a systematic review]. *Chinese Journal of Evidence-Based Medicine* 2011;**11**:670-80.

Zabalegui Yarnoz A, Navarro Diez M, Cabrera Torres E, Fernandez-Puebla AG, Bardallo Porras D, Rodriguez Higueras E*, et al.* [Efficacy of interventions aimed at the main carers of dependent individuals aged more than 65 years old. A systematic review] Eficacia de las intervenciones dirigidas a cuidadores principales de personas dependientes mayores de 65 anos. Una revision sistematica. *Revista Espanola de Geriatria y Gerontologia* 2008;**43**:157-66.

Zenner HP, Delb W, Kroner-Herwig B, Jager B, Peroz I, Hesse G*, et al.* [On the interdisciplinary S3 guidelines for the treatment of chronic idiopathic tinnitus]. *HNO* 2015;**63**:419-27.

Zhang YL, Meng B, Zhao GF. [Application of cognitive behavioral therapy in the rehabilitative treatment of mental diseases]. *Chinese Journal of Clinical Rehabilitation* 2005;**9**:223-5.

Zimmermann T, Heinrichs N. [Psychosocial interventions for women with genital cancers]. *Verhaltenstherapie & Verhaltensmedizin* 2006;**27**:125-41.

Zonnenberg C, Niemantsverdriet M, Blom J, Slotema C. [Auditory verbal hallucinations in patients with borderline personality disorder]. *Tijdschrift voor Psychiatrie* 2016;**58**:122-9.

b. References of studies excluded due to DARE criteria not fulfilled (447)

Aboujaoude E. Three decades of telemedicine in obsessive-compulsive disorder: A review across platforms. *Journal of Obsessive-Compulsive and Related Disorders* 2017;**14**:65-70.

Aboujaoude E, Salame W. Technology at the service of pediatric mental health: Review and assessment. *The Journal of Pediatrics* 2016;**171**:20-4.

Abramowitz JS. Does cognitive-behavioral therapy cure obsessive-compulsive disorder? A meta-analytic evaluation of clinical significance. *Behavior Therapy* 1998;**29**:339-55.

Abramowitz JS, Franklin ME, Foa EB. Empirical status of cognitive-behavioral therapy for obsessive-compulsive disorder: A meta-analytic review. *Romanian Journal of Cognitive & Behavioral Psychotherapies* 2002;**2**:89-104.

Abramowitz JS, Whiteside SP, Deacon BJ. The effectiveness of treatment for pediatric obsessive-compulsive disorder: A meta-analysis. *Behavior Therapy* 2005;**36**:55-63.

Adams C, Wilson P, Bagnall A. Psychosocial interventions for schizophrenia. *Quality in Health Care* 2000;**9**:251-6.

Adams N, Sim J. Rehabilitation approaches in fibromyalgia. *Disability and rehabilitation* 2005;**27**:711-23.

Adams TG, Brady RE, Lohr JM, Jacobs W. A meta-analysis of CBT components for anxiety disorders. *the Behavior Therapist* 2015;**38**:87-97.

Adili F, Larijani B, Haghighatpanah M. Diabetic patients: Psychological aspects. *Annals of the New York Academy of Sciences* 2006;**1084**:329-49.

Agosti V, Nunes EV, O'Shea D. Do manualized psychosocial interventions help reduce relapse among alcohol-dependent adults treated with naltrexone or placebo? A meta-analysis *American Journal on Addictions* 2012;**21**:501-7.

Albazaz R, Wong YT, Homer-Vanniasinkam S. Complex Regional Pain Syndrome: A Review. *Annals of Vascular Surgery* 2008;**22**:297-306.

Albert U, Aguglia A, Bramante S, Bogetto F, Maina G. Treatment-resistant obsessive-compulsive disorder (OCD): Current knowledge and open questions. *Clinical Neuropsychiatry* 2013;**10**:19-30.

Ale CM, McCarthy DM, Rothschild LM, Whiteside SP. Components of Cognitive Behavioral Therapy Related to Outcome in Childhood Anxiety Disorders. *Clinical Child and Family Psychology Review* 2015;**18**:240-51.

Allison DB, Faith MS. Hypnosis as an adjunct to cognitive-behavioral psychotherapy for obesity: a meta-analytic reappraisal. *Journal of Consulting and Clinical Psychology* 1996;**64**:513-6.

Aman MM, Jason Yong R, Kaye AD, Urman RD. Evidence-Based Non-Pharmacological Therapies for Fibromyalgia. *Current Pain and Headache Reports* 2018;**22(5)**.

Andersson G, Hedman E. Effectiveness of guided Internet-based cognitive behavior therapy in regular clinical settings. *Verhaltenstherapie* 2013;**23**:140-8.

Andersson G, Lyttkens L. A meta-analytic review of psychological treatments for tinnitus. *British Journal of Audiology* 1999;**33**:201-10.

Andersson G, Topooco N, Havik O, Nordgreen T. Internet-supported versus face-to-face cognitive behavior therapy for depression. *Expert Review of Neurotherapeutics* 2016;**16**:55-60.

Andrews G, Corry J, Oakley-Browne M, Shepherd L. Australian and New Zealand clinical practice guidelines for the treatment of panic disorder and agoraphobia. *Australian and New Zealand Journal of Psychiatry* 2003;**37**:641-56.

Anthony MT, Farella M. Body dysmorphic disorder and orthodontics--an overview for clinicians. *Australian orthodontic journal* 2014;**30**:208-13.

Armenti NA, Babcock JC. Conjoint treatment for intimate partner violence: A systematic review and implications. *Couple and Family Psychology: Research and Practice* 2016;**5**:109-23.

Arnberg FK, Alaie I, Parling T, Jonsson U. Recent randomized controlled trials of psychological interventions in healthcare: A review of their quantity, scope, and characteristics. *Journal of Psychosomatic Research* 2013;**75**:401-8.

Arumugham SS, Reddy JY. Augmentation strategies in obsessive-compulsive disorder. *Expert Review of Neurotherapeutics* 2013;**13**:187-203.

Astin JA. Mind-Body Therapies for the Management of Pain. *Clinical Journal of Pain* 2004;**20**:27-32.

Bailey AP, Parker AG, Colautti LA, Hart LM, Liu P, Hetrick SE. Mapping the evidence for the prevention and treatment of eating disorders in young people. *Journal of Eating Disorders* 2014;**2**.

Bain KT. Management of chronic insomnia in elderly persons. *American Journal Geriatric Pharmacotherapy* 2006;**4**:168-92.

Baker AL, Hides L, Lubman DI. Treatment of cannabis use among people with psychotic or depressive disorders: A systemic review. *Journal of Clinical Psychiatry* 2010;**71**:247-54.

Bakker A, Van Balkom AJLM, Van Dyck R. Selective serotonin reuptake inhibitors in the treatment of panic disorder and agoraphobia. *International Clinical Psychopharmacology* 2000;**15**:S25-S30.

Ball K, Carver A, Downing K, Jackson M, O'Rourke K. Addressing the social determinants of inequities in physical activity and sedentary behaviours. *Health Promotion International* 2015;**30**:ii8-ii19.

Barkham M, Moller NP, Pybis J. How should we evaluate research on counselling and the treatment of depression? A case study on how the National Institute for Health and Care Excellence's draft 2018 guideline for depression considered what counts as best evidence. *Counselling & Psychotherapy Research* 2017;**17**:253-68.

Barrera M, Jr. Reaffirmation of behavioral approaches to depression treatment. *Clinical Psychology: Science and Practice* 2009;**16**:416-9.

Barron P, Hassiotis A, Banes J. Offenders with intellectual disability: The size of the problem and therapeutic outcomes. *Journal of Intellectual Disability Research* 2002;**46**:454-63.

Barry TJ, Yeung SP, Lau JYF. Meta-analysis of the influence of age on symptom change following cognitive-behavioural treatment for anxiety disorders. *Journal of Adolescence* 2018;**68**:232-41.

Bauer I, Wilansky-Traynor P, Rector NA. Cognitive-behavioral therapy for anxiety disorders with comorbid depression: A review. *International Journal of Cognitive Therapy* 2012;**5**:118-56.

Beelmann A, Losel F. Child social skills training in developmental crime prevention: Effects on antisocial behavior and social competence. *Psicothema* 2006;**18**:603-10.

Beintner I, Jacobi C, Taylor CB. Effects of an Internet-based prevention programme for eating disorders in the USA and Germany--a meta-analytic review. *European Eating Disorders Review* 2012;**20**:1-8.

Belli H, Belli S, Oktay MF, Ural C. Psychopathological dimensions of tinnitus and psychopharmacologic approaches in its treatment. *General Hospital Psychiatry* 2012;**34**:282-9.

Bergfeld IO, Mantione M, Figee M, Schuurman PR, Lok A, Denys D. Treatment-resistant depression and suicidality. *Journal of Affective Disorders* 2018;**235**:362-7.

Best L, Stevens A. *Cognitive behavioural therapy in the treatment of chronic fatigue syndrome*. Southampton: Development and Evaluation Committee, Wessex Institute of Public Health Medicine; 1996.

Bhar SS, Beck AT. Treatment integrity of studies that compare short-term psychodynamic psychotherapy with cognitive-behavior therapy. *Clinical Psychology: Science and Practice* 2009;**16**:370-8.

Birmaher B, Ryan ND, Williamson DE, Brent DA. Childhood and adolescent depression: A review of the past 10 years, Part II. *Journal of the American Academy of Child & Adolescent Psychiatry* 1996;**35**:1575-83.

Bisson JI. Post-traumatic stress disorder. *BMJ* 2007;**14**:789-93.

Blake CS, Hamrin V. Current approaches to the assessment and management of anger and aggression in youth: a review. *Journal of Child & Adolescent Psychiatric Nursing* 2007;**20**:209-21.

Blanco C, Bragdon LB, Schneier FR, Liebowitz MR. The evidence-based pharmacotherapy of social anxiety disorder. *International Journal of Neuropsychopharmacology* 2013;**16**:235-49.

Blanco C, Raza MS, Schneier FR, Liebowitz MR. The evidence-based pharmacological treatment of social anxiety disorder. *International Journal of Neuropsychopharmacology* 2003;**6**:427-42.

Bloch MH, Storch EA. Assessment and Management of Treatment-Refractory Obsessive-Compulsive Disorder in Children. *Journal of the American Academy of Child & Adolescent Psychiatry* 2015;**54**:251-62.

Bluett EJ, Homan KJ, Morrison KL, Levin ME, Twohig MP. Acceptance and commitment therapy for anxiety and OCD spectrum disorders: An empirical review. *Journal of Anxiety Disorders* 2014;**28**:612-24.

Boccia M, Piccardi L, Cordellieri P, Guariglia C, Giannini AM. EMDR therapy for PTSD after motor vehicle accidents: Meta-analytic evidence for specific treatment. 2015;**9**:1-9.

Bolognesi F, Baldwin D, Ruini C. Psychological interventions in the treatment of generalized anxiety disorder: A structured review. *Journal of Psychopathology / Giornale di Psicopatologia* 2014;**20**:111-26.

Bosanac P, Castle D. How should we manage anxiety in patients with schizophrenia? *Australasian Psychiatry* 2015;**23**:374-7.

Brewer M, Melnyk BM. Evidence-based practice. Effective coping/mental health interventions for critically ill adolescents: an evidence review. *Pediatric Nursing* 2007;**33**:361-73.

Bridle D, McCabe R, Priebe S. Incorporating psychotherapeutic methods in routine community treatment for patients with psychotic disorders. *Psychosis: Psychological, Social and Integrative Approaches* 2013;**5**:154-65.

Broomfield NM, Laidlaw K, Hickabottom E, Murray MF, Pendrey R, Whittick JE*, et al.* Post-stroke depression: The case for augmented, individually tailored cognitive behavioural therapy. *Clinical psychology & psychotherapy* 2011;**18**:202-17.

Brown WJ, Dewey D, Bunnell BE, Boyd SJ, Wilkerson AK, Mitchell MA*, et al.* A Critical Review of Negative Affect and the Application of CBT for PTSD. *Trauma, Violence & Abuse* 2018;**19**:176-94.

Buijs PCM, Bassett AS, Boot E. Non-pharmacological treatment of psychiatric disorders in individuals with 22q11.2 deletion syndrome; a systematic review. *American Journal of Medical Genetics, Part A* 2018.

Burgener SC, Twigg P. Interventions for persons with irreversible dementia. *Annual Review of Nursing Research* 2002;**20**:89-124.

Burke BL, Dunn CW, Atkins DC, Phelps JS. The emerging evidence base for motivational interviewing: A meta-analytic and qualitative inquiry. *Journal of Cognitive Psychotherapy* 2004;**18**:309-22.

Burton C. Beyond somatisation: A review of the understanding and treatment of medically unexplained physical symptoms (MUPS). *British Journal of General Practice* 2003;**53**:231-9.

Buse DC, Andrasik F. Behavioral medicine for migraine. *Neurologic Clinics* 2009;**27**:445-65.

Bussotti M, Sommaruga M. Anxiety and depression in patients with pulmonary hypertension: impact and management challenges. *Vascular Health & Risk Management* 2018;**14**:349-60.

Bustillo JR, Lauriello J, Horan WP, Keith SJ. The psychosocial treatment of schizophrenia: An update. *The American journal of psychiatry* 2001;**158**:163-75.

Butts K. *Innovative strategies for students with ADHD: How to employ best practices without losing student interest*: Capella University; 2013.

Calati R, Courtet P, Lopez-Castroman J. Refining Suicide Prevention: a Narrative Review on Advances in Psychotherapeutic Tools. *Current Psychiatry Reports* 2018;**20**.

Canadian Agency for D, Technologies in H. Self-directed cognitive behavioural therapy for adults with diagnosis of depression: systematic review of clinical effectiveness, cost-effectiveness, and guidelines. *CADTH Technology Overviews* 2010;**1**:e0125.

Carpenter J, Gass MLS, Maki PM, Newton KM, Pinkerton JV, Taylor M*, et al.* Nonhormonal management of menopause-Associated vasomotor symptoms: 2015 position statement of the North American Menopause Society. *Menopause* 2015;**22**:1155-74.

Carr A. Depression in young people: Description, assessment and evidence-based treatment. *Developmental Neurorehabilitation* 2008;**11**:3-15.

Carroll KM. Relapse prevention as a psychosocial treatment: A review of controlled clinical trials. *Experimental and Clinical Psychopharmacology* 1996;**4**:46-54.

Chambless DL, Gillis MM. Cognitive therapy of anxiety disorders. *Journal of Consulting and Clinical Psychology* 1993;**61**:248-60.

Chard KM. *A meta-analysis of posttraumatic stress disorder treatment outcome studies of sexually victimized women*: Indiana University; 1995.

Charyton C, Elliott JO, Moore JL, Klatte ET. Is it time to consider cognitive behavioral therapy for persons with epilepsy? Clues from pathophysiology, treatment and functional neuroimaging. *Expert Review of Neurotherapeutics* 2010;**10**:1911-27.

Chen E, Cole SW, Kato PM, Chen E. A review of empirically supported psychosocial interventions for pain and adherence outcomes in sickle cell disease. *Journal of Pediatric Psychology* 2004;**29**:197-209.

Chien WT, Leung SF, Yeung FKK, Wong WK. Current approaches to treatments for schizophrenia spectrum disorders, part II: Psychosocial interventions and patient-focused perspectives in psychiatric care. *Neuropsychiatric Disease and Treatment* 2013;**9**:1463-81.

Christensen H, Batterham P, Calear A. Online interventions for anxiety disorders. *Current Opinion in Psychiatry* 2014;**27**:7-13.

Clauw DJ. Fibromyalgia: a clinical review. *JAMA: Journal of the American Medical Association* 2014;**311**:1547-55.

Cochrane G. Role for a sense of self-worth in weight-loss treatments: Helping patients develop self-efficacy. *Canadian Family Physician* 2008;**54**:543-7.

Colom F, Lam D. Psychoeducation: Improving outcomes in bipolar disorder. *European Psychiatry* 2005;**20**:359-64.

Colom F, Vieta E. A perspective on the use of psychoeducation, cognitive-behavioral therapy and interpersonal therapy for bipolar patients. *Bipolar Disorders* 2004;**6**:480-6.

Compton SN, Kratochvil CJ, March JS. Pharmacotherapy for anxiety disorders in children and adolescents: An evidence-based medicine review. *Psychiatric Annals* 2007;**37**:504-17.

Comtois KA, Linehan MM. Psychosocial treatments of suicidal behaviors: A practice-friendly review. *Journal of Clinical Psychology* 2006;**62**:161-70.

Conn DK, Seitz DP. Advances in the treatment of psychiatric disorders in long-term care homes. *Current Opinion in Psychiatry* 2010;**23**:516-21.

Copeland J, Gates P, Pokorski I. A narrative review of psychological cannabis use treatments with and without pharmaceutical adjunct. *Current Pharmaceutical Design* 2016;**22**:6397-408.

Cordioli AV. Cognitive-behavioral therapy in obsessive-compulsive disorder. *Revista Brasileira de Psiquiatria* 2008;**30**:S65-S72.

Cottraux J. Nonpharmacological treatments for anxiety disorders. *Dialogues in Clinical Neuroscience* 2002;**4**:305-19.

Crabb RM, Cavanagh K, Proudfoot J, Learmonth D, Rafie S, Weingardt KR. Is computerized cognitive-behavioural therapy a treatment option for depression in late-life? A systematic review. *British Journal of Clinical Psychology* 2012;**51**:459-64.

Craft LL, Perna FM. The Benefits of Exercise for the Clinically Depressed. *Primary Care Companion to the Journal of Clinical Psychiatry* 2004;**6**:104-11.

Craig LA, Browne KD, Stringer I. Treatment and sexual offence recidivism. *Trauma, Violence & Abuse* 2003;**4**:70-89.

Crider A, Glaros AG, Gevirtz RN. Efficacy of biofeedback-based treatments for temporomandibular disorders. *Applied Psychophysiology Biofeedback* 2005;**30**:333-45.

Crino RD. Psychological treatment of obsessive compulsive disorder: An update. *Australasian Psychiatry* 2015;**23**:347-9.

Crowe M, Porter R. Inpatient treatment for mania: A review and rationale for adjunctive interventions. *Australian and New Zealand Journal of Psychiatry* 2014;**48**:716-21.

Cuijpers P. Are all psychotherapies equally effective in the treatment of adult depression? The lack of statistical power of comparative outcome studies. *Evidence Based Mental Health* 2016;**19**:39-42.

Cuijpers P, Gentili C. Psychological treatments are as effective as pharmacotherapies in the treatment of adult depression: A summary from Randomized Clinical Trials and neuroscience evidence. *Research in Psychotherapy: Psychopathology, Process and Outcome* 2017;**20**:147-52.

Cunha LM. *The efficacy of therapeutic interventions for adolescent maltreatment victims: A meta-analysis*: Alliant International University; 2008.

Currier JM. *Psychotherapeutic interventions for grief: A comprehensive review of controlled outcome research*: The University of Memphis; 2009.

Currier JM, Holland JM, Neimeyer RA. Do CBT-based interventions alleviate distress following bereavement? A review of the current evidence. *International Journal of Cognitive Therapy* 2010;**3**:77-93.

Cwikel J, Behar L, Rabson-Hare J. A comparison of a vote count and a meta-analysis review of intervention research with adult cancer patients. *Research on Social Work Practice* 2000;**10**:139-58.

Dautovich ND, McNamara J, Williams JM, Cross NJ, McCrae CS. Tackling sleeplessness: Psychological treatment options for insomnia. *Nature & Science of Sleep* 2010;**2**:23-37.

Davidson MA. ADHD in adults: A review of the literature. *Journal of Attention Disorders* 2008;**11**:628-41.

de Carvalho MR, Rozenthal M, Nardi AE. The fear circuitry in panic disorder and its modulation by cognitive-behaviour therapy interventions. *World Journal of Biological Psychiatry* 2010;**11**:188-98.

De Silva S, Parker A, Purcell R, Callahan P, Liu P, Hetrick S. Mapping the evidence of prevention and intervention studies for suicidal and self-harming behaviors in young people. *Crisis* 2013;**34**:223-32.

Deary V, Chalder T, Sharpe M. The cognitive behavioural model of medically unexplained symptoms: A theoretical and empirical review. *Clinical Psychology Review* 2007;**27**:781-97.

Decker SE, Kiluk BD, Frankforter T, Babuscio T, Nich C, Carroll KM. Just showing up is not enough: Homework adherence and outcome in cognitive-behavioral therapy for cocaine dependence. *Journal of Consulting & Clinical Psychology* 2016;**84**:907-12.

Diseth TH, Christie HJ. Trauma-related dissociative (conversion) disorders in children and adolescents - An overview of assessment tools and treatment principles. *Nordic Journal of Psychiatry* 2005;**59**:278-92.

do Amaral JMX, Spadaro PTM, Pereira VM, e Silva ACO, Nardi AE. The carbon dioxide challenge test in panic disorder: A systematic review of preclinical and clinical research. *Revista Brasileira de Psiquiatria* 2013;**35**:318-31.

Domhardt M, Baumeister H. Psychotherapy of adjustment disorders: Current state and future directions. *World Journal of Biological Psychiatry* 2018;**19**:S21-S35.

Draper ML, Velligan DI, Tai S. Cognitive behavioral therapy for schizophrenia: A review of recent literature and meta-analyses. *Minerva Psichiatrica* 2010;**51**:85-94.

Dubicka B, Brent D. Combined therapy in adolescent depression. *International Journal of Cognitive Therapy* 2014;**7**:136-48.

Duff W, Haskey N, Potter G, Alcorn J, Hunter P, Fowler S. Non-pharmacologic therapies for inflammatory bowel disease: Recommendations for self-care and physician guidance. *American Journal of Gastroenterology* 2017;**112**:S612-S3.

Duff W, Haskey N, Potter G, Alcorn J, Hunter P, Fowler S. Non-pharmacological therapies for inflammatory bowel disease: Recommendations for self-care and physician guidance. *World Journal of Gastroenterology* 2018;**24**:3055-70.

Dunn RL, Schwebel AI. Meta-Analytic Review of Marital Therapy Outcome Research. *Journal of Family Psychology* 1995;**9**:58-68.

Dutcher TD. *A meta-analytic study of marital therapy modalities and clients' presenting problems*: The Union Institute; 2000.

Dy SM, Apostol CC. Evidence-based approaches to other symptoms in advanced cancer. *Cancer Journal* 2010;**16**:507-13.

Eckhardt CI, Murphy CM, Whitaker DJ, Sprunger J, Dykstra R, Woodard K. The effectiveness of intervention programs for perpetrators and victims of intimate partner violence *Partner Abuse* 2013;**4**:196-231.

Eddy KT, Dutra L, Bradley R, Westen D. A multidimensional meta-analysis of psychotherapy and pharmacotherapy for obsessive-compulsive disorder. *Clinical Psychology Review* 2004;**24**:1011-30.

Ehlers A, Clark D. Early psychological interventions for adult survivors of trauma: A review. *Biological Psychiatry* 2003;**53**:817-26.

Ehrenzeller MF, Mayer DK, Goldstein A. Smoking Prevalence and Management Among Cancer Survivors. *Oncology Nursing Forum* 2018;**45**:55-68.

Elliott R. The effectiveness of humanistic therapies: A meta-analysis. In: Cain DJ, editor. *Humanistic psychotherapies: Handbook of research and practice* Washington, DC: American Psychological Association; 2002:57-81.

Elliott R. Person-centered/experiential psychotherapy for anxiety difficulties: Theory, research and practice. *Person-Centered and Experiential Psychotherapies* 2013;**12**:16-32.

Ellis P, Hickie I, Bushnell J, Hirini P, Stevens S, Smith DAR. Australian and New Zealand clinical practice guidelines for the treatment of depression. *Australian and New Zealand Journal of Psychiatry* 2004;**38**:389-407.

El-Mallakh P, Findlay J. Strategies to improve medication adherence in patients with schizophrenia: The role of support services. *Neuropsychiatric Disease and Treatment* 2015;**11**:1077-90.

Emmerik AA, Reijntjes A, Kamphuis JH. Writing therapy for posttraumatic stress: a meta-analysis. *Psychotherapy and Psychosomatics* 2013;**82**:82-8.

Fedoroff IC, Taylor S. Psychological and pharmacological treatments of social phobia: A meta-analysis. *Journal of Clinical Psychopharmacology* 2001;**21**:311-24.

Fernandez E, Salem D, Swift JK, Ramtahal N. Meta-Analysis of Dropout From Cognitive Behavioral Therapy: Magnitude, Timing, and Moderators. *Journal of Consulting and Clinical Psychology* 2015;**24**.

Field T. Postnatal anxiety prevalence, predictors and effects on development: A narrative review. *Infant Behavior and Development* 2018;**51**:24-32.

Fingeret MC, Teo I, Epner DE. Managing body image difficulties of adult cancer patients: lessons from available research. *Cancer* 2014;**120**:633-41.

Fisher EB, Thorpe CT, DeVellis BM, DeVellis RF. Healthy coping, negative emotions, and diabetes management: a systematic review and appraisal. *Diabetes Educator* 2007;**33**:1080-103.

Flannery V. Increasing Breastfeeding Rates: Evidence-Based Strategies. *International Journal of Childbirth Education* 2014;**29**:59-62.

Fluckiger C, Del Re AC, Wampold BE, Symonds D, Horvath AO. How central is the alliance in psychotherapy? A multilevel longitudinal meta-analysis. *Journal of counseling psychology* 2012;**59**:10-7.

Foa EB. Psychosocial treatment of posttraumatic stress disorder. *Journal of Clinical Psychiatry* 2000;**61(S5)**:43-8.

Fontenelle LF, Nascimento AL, Mendlowicz MV, Shavitt RG, Versiani M. An update on the pharmacological treatment of obsessive-compulsive disorder. *Expert Opinion on Pharmacotherapy* 2007;**8**:563-83.

Foral P, Dewan N, Malesker M. Insomnia: A therapeutic review for pharmacists. *Consultant Pharmacist* 2011;**26**:332-41.

Galante E, Gazzi L, Caffarra S. Psychological activities in neurorehabilitation: From research to clinical practice. *Giornale Italiano di Medicina del Lavoro ed Ergonomia* 2011;**33**:A19-A28.

Gale CK, Millichamp J. Generalised anxiety disorder. *BMJ clinical evidence* 2007.

Ganasen K, Ipser J, Stein D. Augmentation of cognitive behavioral therapy with pharmacotherapy. *Psychiatric Clinics of North America* 2010;**33**:687-99.

Gasparini S, Beghi E, Ferlazzo E, Beghi M, Belcastro V, Biermann KP*, et al.* Management of psychogenic non-epileptic seizures: a multidisciplinary approach. *European Journal of Neurology* 2019;**26**:205-e15.

Gaudiano BA. Cognitive behavior therapies for psychotic disorders: Current empirical status and future directions. *Clinical Psychology: Science and Practice* 2005;**12**:33-50.

Gaudin D, Krafcik BM, Mansour TR, Alnemari A. Considerations in Spinal Fusion Surgery for Chronic Lumbar Pain: Psychosocial Factors, Rating Scales, and Perioperative Patient Education-A Review of the Literature. *World Neurosurgery* 2017;**98**:21-7.

Gellatly J, Bower P, Hennessy S, Richards D, Gilbody S, Lovell K. What makes self-help interventions effective in the management of depressive symptoms? Meta-analysis and meta-regression. *Psychological Medicine* 2007;**37**:1217-28.

Genoves VG, Morales LA, Sanchez-Meca J. What works for serious juvenile offenders? A systematic review. *Psicothema* 2006;**18**:611-9.

Gewirtz A, Minen M. Adherence to Behavioral Therapy for Migraine: Knowledge to Date, Mechanisms for Assessing Adherence, and Methods for Improving Adherence. *Current pain and headache reports* 2019;**23**:3.

Ghafoori B. *Effectiveness of cognitive-behavioral therapy in reducing classroom disruptive behaviors: A meta-analysis*. (ERIC Document Reproduction Service No. ED457182); 2001.

Gil PJ, Carrillo F, Meca JS. Effectiveness of cognitive-behavioural treatment in social phobia: A meta-analytic review. *Psychology in Spain* 2001;**5**:17-25.

Giles TR, Prial EM, Neims DM. Evaluating psychotherapies: A comparison of effectiveness. *International Journal of Mental Health* 1993;**22**:43-65.

Goldenberg DL, Burckhardt C, Crofford L, Goldenberg DL, Burckhardt C, Crofford L. Management of fibromyalgia syndrome. *JAMA: Journal of the American Medical Association* 2004;**292**:2388-95.

Gomez-de-Regil L, Alvarez-Nemegyei J. Open access scientific evidence of Cognitive Behavioral Therapy for patients with fibromyalgia. *Actualidades en Psicologia* 2016;**30**:91-102.

Gontard A, Niemczyk J, Wagner C, Equit M. Voiding postponement in children-a systematic review. *European Child & Adolescent Psychiatry* 2016;**25**:809-20.

Gonzales AH, Bergstrom L. Adolescent Non-Suicidal Self-Injury (NSSI) Interventions. *Journal of Child & Adolescent Psychiatric Nursing* 2013;**26**:124-30.

Goodwin PJ. Psychosocial support for women with advanced breast cancer. *Breast Cancer Research and Treatment* 2003;**81**:S103-S10.

Gould MS, Greenberg T, Velting DM, Shaffer D. Youth suicide risk and preventive interventions: A review of the past 10 years. *Journal of the American Academy of Child and Adolescent Psychiatry* 2003;**42**:386-405.

Gould RA, Johnson MW. Comparative effectiveness of cognitive-behavioral treatment and pharmacotherapy for social phobia: Meta-analytic outcome. 2001:379-90.

Graham J. Cognitive behavioural therapy for occupational trauma: A systematic literature review exploring the effects of occupational trauma and the existing CBT support pathways and interventions for staff working within mental healthcare including allied professions. *the Cognitive Behaviour Therapist* 2012;**5**:24-45.

Grambal A, Prasko J, Ociskova M, Slepecky M, Kotianova A, Sedlackova Z*, et al.* Borderline personality disorder and unmet needs. *Neuroendocrinology Letters* 2017;**38**:275-89.

Grant JE, Odlaug BL, Donahue C. Gambling. In: Hofmann SG, editor. *The Wiley Handbook of Cognitive Behavioural Therapy.* Chichester: Wiley-Blackwell; 2014.

Grech E. Psychological interventions for psychosis: A critical review of the current evidence. *Internet Journal of Mental Health* 2004;**1**:1-9.

Gregory VL, Jr. Cognitive-behavioral therapy for depression in bipolar disorder: a meta-analysis. *Journal of Evidence-Based Social Work* 2010;**7**:269-79.

Gremeaux V, Coudeyre E. The Internet and the therapeutic education of patients: A systematic review of the literature. [French] Internet et education therapeutique des patients: revue systematique de la litterature. *Annals of Physical and Rehabilitation Medicine* 2010;**53**:669-92.

Grossman LS, Martis B, Fichtner CG. Are sex offenders treatable? A research overview. *Psychiatric Services* 1999;**50**:349-61.

Guay DRP. Drug treatment of paraphilic and nonparaphilic sexual disorders. *Clinical Therapeutics* 2009;**31**:1-31.

Guidi J, Tomba E, Cosci F, Park SK, Fava GA. The role of staging in planning psychotherapeutic interventions in depression. *Journal of Clinical Psychiatry* 2017;**78**:456-63.

Gutierrez M, Sanchez M, Trujillo A, Sanchez L. Cognitive-behavioral therapy for chronic psychosis. *Actas Espanolas de Psiquiatria* 2009;**37**:106-14.

Hallberg SC, Lisboa CS, de Souza DB, Mester A, Braga AZ, Strey AM, *et al.* Systematic review of research investigating psychotherapy and information and communication technologies. *Trends in Psychiatry & Psychotherapy* 2015;**37**:118-25.

Halvorsen JG, Metz ME. Sexual dysfunction, Part II: Diagnosis, management, and prognosis. *Journal of the American Board of Family Practice* 1992;**5**:177-92.

Ham P, Waters DB, Oliver MN. Treatment of panic disorder. *American Family Physician* 2005;**71**:733-810.

Hammond A. Rehabilitation in rheumatoid arthritis: a critical review. *Musculoskeletal care* 2004;**2**:135-51.

Harrington R, Whittaker J, Shoebridge P. Psychological treatment of depression in children and adolescents. A review of treatment research. *British Journal of Psychiatry* 1998;**173**:291-8.

Hauser W, Arnold B, Eich W, Felde E, Flugge C, Henningsen P*, et al.* Management of fibromyalgia syndrome--an interdisciplinary evidence-based guideline. *German Medical Science* 2008;**6**:Doc14.

Hayee B, Forgacs I. Psychological approach to managing irritable bowel syndrome. *British Medical Journal* 2007;**334**:1105-9.

Heimberg RG. Current status of psychotherapeutic interventions for social phobia. *Journal of Clinical Psychiatry* 2001;**62**:36-42.

Heimberg RG. Cognitive-behavioral therapy for social anxiety disorder: Current status and future directions. *Biological Psychiatry* 2002;**51**:101-8.

Herpertz S, Hagenah U, Vocks S, Von Wietersheim J, Cuntz U, Zeeck A. The diagnosis and treatment of eating disorders. *Deutsches Arzteblatt* 2011;**108**:678-85

Hetrick SE, Cox GR, Fisher CA, Bhar SS, Rice SM, Davey CG*, et al.* Back to basics: Could behavioural therapy be a good treatment option for youth depression? A critical review. *Early Intervention in Psychiatry* 2015;**9**:93-9.

Hewitt J, Coffey M. Therapeutic working relationships with people with schizophrenia: literature review. *Journal of Advanced Nursing* 2005;**52**:561-70.

Hitsman B, Papandonatos GD, McChargue DE, Demott A, Herrera MJ, Spring B*, et al.* Past major depression and smoking cessation outcome: a systematic review and meta-analysis update. *Addiction* 2013;**108**:294-306.

Ho BP, Stephenson J, Carter M. Cognitive-behavioral approaches for children with autism spectrum disorder: A trend analysis. *Research in Autism Spectrum Disorders* 2018;**45**:27-41.

Hoch E, Bonnetn U, Thomasius R, Ganzer F, Havemann-Reinecke U, Preuss UW. Risks associated with the non-medicinal use of cannabis. *Deutsches Arzteblatt International* 2015;**112**:271-8.

Hollon SD, Jarrett RB, Nierenberg AA, Thase ME, Trivedi M, Rush AJ. Psychotherapy and medication in the treatment of adult and geriatric depression: Which monotherapy or combined treatment? *Journal of Clinical Psychiatry* 2005;**66**:455-68.

Howell D, Oliver TK, Keller-Olaman S, Davidson J, Garland S, Samuels C*, et al.* A Pan-Canadian practice guideline: prevention, screening, assessment, and treatment of sleep disturbances in adults with cancer. *Supportive Care in Cancer* 2013;**21**:2695-706.

Hruschak V, Cochran G, Wasan AD. Psychosocial interventions for chronic pain and comorbid prescription opioid use disorders: A narrative review of the literature. *Journal of Opioid Management* 2018;**14**:345-58.

Hutchinson AD, Wilson C. Improving nutrition and physical activity in the workplace: a meta-analysis of intervention studies. *Health Promotion International* 2012;**27**:238-49.

Huynh ME, Vandvik IH, Diseth TH. Hypnotherapy in Child Psychiatry: The State of the Art. *Clinical Child Psychology & Psychiatry* 2008;**13**:378-94.

Hwang MS, Yeagley KL, Petosa R. A meta-analysis of adolescent psychosocial smoking prevention programs published between 1978 and 1997 in the United States. *Health Education & Behavior* 2004;**31**:702-19.

Janicke DM, Finney JW. Empirically supported treatments in pediatric psychology: Recurrent abdominal pain. *Journal of Pediatric Psychology* 1999;**24**:115-27.

Jarrell JF, Vilos GA, Allaire C, Burgess S, Fortin C, Gerwin R*, et al.* No. 164-Consensus Guidelines for the Management of Chronic Pelvic Pain. *Journal of Obstetrics & Gynaecology Canada: JOGC* 2018;**40**:e747-e87.

Jassogne C, Zdanowicz N. Management of adult patients with anorexia nervosa: a literature review. *Psychiatria Danubina* 2018;**30**:533-6.

Jayasekara R, Procter N, Harrison J, Skelton K, Hampel S, Draper R*, et al.* Cognitive behavioural therapy for older adults with depression: a review. *Journal of Mental Health* 2015;**24**:168-71.

Jeffries FW, Davis P. What is the role of eye movements in eye movement desensitization and reprocessing (EMDR) for post-traumatic stress disorder (PTSD)? A review. *Behavioural and Cognitive Psychotherapy* 2013;**41**:290-300.

Jhanjee S. Evidence based psychosocial interventions in substance use. *Indian Journal of Psychological Medicine* 2014;**36**:112-8.

Julius RJ, Novitsky MA, Jr., Dubin WR. Medication adherence: A review of the literature and implications for clinical practice. *Journal of Psychiatric Practice* 2009;**15**:34-44.

Kaplan MS, Krueger RB. Cognitive-behavioral treatment of the paraphilias. *The Israel journal of psychiatry and related sciences* 2012;**49**:291-6.

Kaptein AA, van Dijk S, Broadbent E, Falzon L, Thong M, Dekker FW. Behavioural research in patients with end-stage renal disease: A review and research agenda. *Patient Education and Counseling* 2010;**81**:23-9.

Kazantzis N, Deane FP, Ronan KR. Homework assignments in cognitive and behavioral therapy: A meta-analysis. *Clinical Psychology: Science and Practice* 2000;**7**:189-202.

Killgore WD. Academic and research interest in several approaches to psychotherapy: a computerized search of literature in the past 16 years. *Psychological Reports* 2000;**87**:717-20.

Kirsch I. Hypnotic enhancement of cognitive-behavioral weight loss treatments--another meta-reanalysis. *Journal of Consulting and Clinical Psychology* 1996;**64**:517-9.

Kirsch I, Montgomery G, Sapirstein G. Hypnosis as an adjunct to cognitive-behavioral psychotherapy: A meta- analysis. *Journal of Consulting and Clinical Psychology* 1995;**63**:214-20.

Knouse LE, Teller J, Brooks MA. Meta-analysis of cognitive-behavioral treatments for adult ADHD. *Journal of Consulting and Clinical Psychology* 2017;**85**:737-50.

Koder DA, Brodaty H, Anstey KJ. Cognitive therapy for depression in the elderly. *International Journal of Geriatric Psychiatry* 1996;**11**:97-107.

Korczak D, Wastian M, Schneider M. Therapy of the burnout syndrome. *GMS Health Technology Assessment* 2012;**8**:Doc05.

Kovacs M, Lopez-Duran N. Prodromal symptoms and atypical affectivity as predictors of major depression in juveniles: implications for prevention. *Journal of Child Psychology & Psychiatry* 2010;**51**:472-96.

Kroon Van Diest AM, Powers SW. Cognitive Behavioral Therapy for Pediatric Headache and Migraine: Why to Prescribe and What New Research Is Critical for Advancing Integrated Biobehavioral Care. *Headache* 2018;**59**:289-97.

Krueger SJ, Glass CR. Integrative psychotherapy for children and adolescents: A practice-oriented literature review. *Journal of Psychotherapy Integration* 2013;**23**:331-44.

Kunkle B, Bae S, Singh KP, Roy D. Increased Risk of Childhood Brain Tumors among Children Whose Parents Had Farm-Related Pesticide Exposures during Pregnancy. *Jp Journal of Biostatistics* 2014;**11**:89-101.

Kwekkeboom KL. Cancer Symptom Cluster Management. *Seminars in Oncology Nursing* 2016;**32**:373-82.
Lam S, MacIna LO. Therapy update for insomnia in the elderly. *Consultant Pharmacist* 2017;**32**:610-22.

Lamarche LJ, De Koninck J. Sleep disturbance in adults with posttraumatic stress disorder: A review. *Journal of Clinical Psychiatry* 2007;**68**:1257-70.

Lampe L, Coulston C, Berk L. Psychological management of unipolar depression. *Acta Psychiatrica Scandinavica* 2013;**127**:24-37.

Lampe LA. Social anxiety disorder: recent developments in psychological approaches to conceptualization and treatment. *Australian & New Zealand Journal of Psychiatry* 2009;**43**:887-98.

Landenberger NA, Lipsey MW. The positive effects of cognitive-behavioral programs for offenders: A meta-analysis of factors associated with effective treatment. *Journal of Experimental Criminology* 2005;**1**:451-76.

Lazar SG, Offenkrantz W. Psychotherapy in the treatment of posttraumatic stress disorder. In: Lazar SG, editor. *Psychotherapy Is Worth It: A Comprehensive Review of Its Cost-Effectiveness.* Washington DC, USA, London, England: American Psychiatric Publishing, Inc; 2010:87-102.

Le Grange D, Schmidt U. The treatment of adolescents with bulimia nervosa. *Journal of Mental Health* 2005;**14**:587-97.

Le HN, Boyd RC. Prevention of major depression: Early detection and early intervention in the general population. *Clinical Neuropsychiatry* 2006;**3**:6-22.

Leddy JJ, Sandhu H, Sodhi V, Baker JG, Willer B. Rehabilitation of Concussion and Post-concussion Syndrome. *Sports & Health* 2012;**4**:147-54.

Leichsenring F, Hiller W, Weissberg M, Leibing E. Cognitive-behavioral therapy and psychodynamic psychotherapy: Techniques, efficacy, and indications. *American Journal of Psychotherapy* 2006;**60**:233-59.

Leichsenring F, Leibing E. Psychodynamic psychotherapy: A systematic review of techniques, indications and empirical evidence. *Psychology and Psychotherapy: Theory, Research and Practice* 2007;**80**:217-28.

Lejoyeux M, Weinstein A. Compulsive buying. *The American Journal of Drug and Alcohol Abuse* 2010;**36**:248-53.

Lener MS, Iosifescu DV. In pursuit of neuroimaging biomarkers to guide treatment selection in major depressive disorder: a review of the literature. *Annals of the New York Academy of Sciences* 2015;**1344**:50-65.

Lenz A, Haktanir A, Callender K. Meta-analysis of trauma-focused therapies for treating the symptoms of posttraumatic stress disorder. *Journal of Counseling & Development* 2017;**95**:339-53.

Lett HS, Davidson J, Blumenthal JA. Nonpharmacologic treatments for depression in patients with coronary heart disease. *Psychosomatic Medicine* 2005;**67**:S58-S62.

Leung KS, Cottler LB. Treatment of pathological gambling. *Current Opinion in Psychiatry* 2009;**22**:69-74.

Levenstein S. The evidence for treatments for somatoform disorders: A view from the trenches. In: Gresser M, editor. *Somatic presentations of mental disorders: Refining the research agenda for DSM-V.* Arlington, VA: American Psychological Association; 2009:165-9.

Linden W, Chambers L. Clinical effectiveness of non-drug treatment for hypertension: A meta- analysis. *Annals of Behavioral Medicine* 1994;**16**:35-45.

Linton SJ, Bergbom S. Understanding the link between depression and pain. *Scandinavian Journal of Pain* 2011;**2**:47-54.

Liu P, Parker AG, Hetrick SE, Callahan P, de Silva S, Purcell R. An evidence map of interventions across premorbid, ultra-high risk and first episode phases of psychosis. *Schizophrenia Research* 2010;**123**:37-44.

Ljotsson B, Hedman E, Mattsson S, Andersson E. The effects of cognitive-behavioral therapy for depression are not falling: A re-analysis of Johnsen and Friborg (2015). *Psychological Bulletin* 2017;**143**:321-5.

Lock J. An Update on Evidence-Based Psychosocial Treatments for Eating Disorders in Children and Adolescents. *Journal of clinical child and adolescent psychology : the official journal for the Society of Clinical Child and Adolescent Psychology, American Psychological Association, Division 53* 2015;**44**:707-21.

Loerinc AG, Meuret AE, Twohig MP, Rosenfield D, Bluett EJ, Craske MG. Response rates for CBT for anxiety disorders: Need for standardized criteria. *Clinical Psychology Review* 2015;**42**:72-82.

Losel F, Beelmann A. Effects of child skills training in preventing antisocial behavior: A systematic review of randomized evaluations. *Annals of the American Academy of Political and Social Science* 2003;**587**:84-109.

Lourenco Leite P, Pereira VM, Nardi AE, Silva AC. Psychotherapy for compulsive buying disorder: A systematic review. *Psychiatry Research* 2014;**219**:411-9.

Luby JL. Treatment of anxiety and depression in the preschool period. *Journal of the American Academy of Child & Adolescent Psychiatry* 2013;**52**:346-58.

Luhmann D, Stoll S, Burkhardt-Hammer T, Raspe H. Prevention of relapsing backache. *GMS Health Technology Assessment* 2006;**2**:Doc12.

Lundgren JD, Danoff-Burg S, Anderson DA. Cognitive-behavioral therapy for bulimia nervosa: an empirical analysis of clinical significance. *International Journal of Eating Disorders* 2004;**35**:262-74.

Luskin FM, Newell KA, Griffith M, Holmes M, Telles S, DiNucci E*, et al.* A review of mind/body therapies in the treatment of musculoskeletal disorders with implications for the elderly. *Alternative Therapies in Health and Medicine* 2000;**6**:46-56.

Luskin FM, Newell KA, Griffith M, Holmes M, Telles S, Marvasti FF*, et al.* A review of mind-body therapies in the treatment of cardiovascular disease. Part 1: Implications for the elderly. *Alternative Therapies in Health and Medicine* 1998;**4**:46-61.

Lustman PJ, Clouse RE. Depression in diabetic patients: The relationship between mood and glycemic control. *Journal of Diabetes and its Complications* 2005;**19**:113-22.

Lustyk MKB, Gerrish WG, Shaver S, Keys SL. Cognitive-behavioral therapy for premenstrual syndrome and premenstrual dysphoric disorder: A systematic review. *Archives of Women's Mental Health* 2009;**12**:85-96.

MacLeod S, Musich S, Kraemer S, Wicker E. Practical non-pharmacological intervention approaches for sleep problems among older adults. *Geriatric Nursing* 2018;**39**:506-12.

Mai F. Somatization disorder: A practical review. *Canadian Journal of Psychiatry* 2004;**49**:652-62.

Maia AC, Braga AA, Soares-Filho G, Pereira V, Nardi AE, Silva AC. Efficacy of cognitive behavioral therapy in reducing psychiatric symptoms in patients with implantable cardioverter defibrillator: an integrative review. *Brazilian Journal of Medical & Biological Research* 2014;**47**:265-72.

Makris UE, Abrams RC, Gurland B, Reid MC. Management of persistent pain in the older patient: a clinical review. *JAMA: Journal of the American Medical Association* 2014;**312**:825-36.

Mallick S. Palliative care in Parkinson's disease: role of cognitive behavior therapy. *Indian Journal of Palliative Care* 2009;**15**:51-6.

Manfro GG, Heldt E, Cordioli AV, Otto MW. Cognitive-behavioral therapy in panic disorder. . *Revista Brasileira de Psiquiatria* 2008;**30**:S81-S7.

Maquet D, Demoulin C, Croisier JL, Crielaard JM. Benefits of physical training in fibromyalgia and related syndromes. *Annales de Readaptation et de Medecine Physique* 2007;**50**:363-8.

Marazziti D, Consoli G. Treatment strategies for obsessive-compulsive disorder. *Expert Opinion on Pharmacotherapy* 2010;**11**:331-43.

Marcotte D. Treating depression in adolescence: a review of the effectiveness of cognitive-behavioral treatments. *Journal of Youth and Adolescence* 1997;**26**:273-83.

Marcus DK, O'Connell D, Norris AL, Sawaqdeh A. Is the Dodo bird endangered in the 21st century? A meta-analysis of treatment comparison studies. *Clinical Psychology Review* 2014;**34**:519-30.

Maricutoiu LP, Sava FA, Butta O. The effectiveness of controlled interventions on employees' burnout: A meta-analysis. *Journal of Occupational and Organizational Psychology* 2016;**89**:1-27.

Marsch LA, Hegel MT, Greene MA. Leveraging digital technology to intervene on personality processes to promote healthy aging. *Personality Disorders: Theory, Research, & Treatment* 2019;**10**:33-45.

Martin GW, Rehm J, Martin GW, Rehm J. The effectiveness of psychosocial modalities in the treatment of alcohol problems in adults: a review of the evidence. *Canadian Journal of Psychiatry* 2012;**57**:350-8.

Math SB, Janardhan Reddy YC. Issues in the pharmacological treatment of obsessive-compulsive disorder. *International Journal of Clinical Practice* 2007;**61**:1188-97.

Maxwell N, Scourfield J, Featherstone B, Holland S, Tolman R. Engaging fathers in child welfare services: a narrative review of recent research evidence. *Child & Family Social Work* 2012;**17**:160-9.

McClellan JM, Werry JS. Evidence-Based Treatments in Child and Adolescent Psychiatry: An Inventory. *Journal of the American Academy of Child & Adolescent Psychiatry* 2003;**42**:1388-400.

McCormack M, Tierney K, Brennan D, Lawlor E, Clarke M. Lack of insight in psychosis: theoretical concepts and clinical aspects. *Behavioural and Cognitive Psychotherapy* 2014;**42**:327-38.

McCracken LM, Thompson M. Psychological advances in chronic pain: a concise selective review of research from 2010. *Current opinion in supportive and palliative care* 2011;**5**:122-6.

McCracken LM, Turk DC. Behavioral and cognitive-behavioral treatment for chronic pain: Outcome, predictors of outcome, and treatment process. *Spine* 2002;**27**:2564-73.

McCrady BS, Owens MD, Borders AZ, Brovko JM. Psychosocial approaches to alcohol use disorders since 1940: a review. *Journal of Studies on Alcohol and Drugs* 2014;**75 Suppl 17**:68-78.

McGuire AW, Ahearn E, Doering LV. Psychological distress and cardiovascular disease. *Journal of Clinical Outcomes Management* 2015;**22**:421-32.

McHugh R, Hearon BA, Otto MW. Cognitive behavioral therapy for substance use disorders. *Psychiatric Clinics of North America* 2010;**33**:511-25.

McLntyre RS, Alsuwaidan M, Goldstein BI, Taylor VH, Beaulieu S, Schaffer A*, et al.* The Canadian Network for Mood and Anxiety Treatments (CANMAT) task force recommendations for the management of patients with mood disorders and comorbid metabolic disorders. *Annals of Clinical Psychiatry* 2012;**24**:69-81.

McMain S, Pos AE. Advances in psychotherapy of personality disorders: A research update. *Current Psychiatry Reports* 2007;**9**:46-52.

McManus F, Shafran R, Cooper Z. What does a 'transdiagnostic' approach have to offer the treatment of anxiety disorders? *British Journal of Clinical Psychology* 2010;**49**:491-505.

McPherson S, Evans C, Richardson P. The NICE Depression Guidelines and the recovery model: is there an evidence base for IAPT? *Journal of Mental Health* 2009;**18**:405-14.

Mehler-Wex C, Kolch M. Depression in children and adolescents. . *Deutsches Arzteblatt* 2008;**105**:149-55.

Melendez JC, McCrank E. Anxiety-related reactions associated with magnetic resonance imaging examinations. *Journal of the American Medical Association* 1993;**270**:745-7.

Menon V, Rajan TM, Kuppili PP, Sarkar S. Cognitive Behavior Therapy for Medically Unexplained Symptoms: A Systematic Review and Meta-analysis of Published Controlled Trials. *Indian Journal of Psychological Medicine* 2017;**39**:399-406.

Middaugh SJ, Pawlick K. Biofeedback and behavioral treatment of persistent pain in the older adult: A review and a study. *Applied Psychophysiology Biofeedback* 2002;**27**:185-202.

Milin R, Walker S, Chow J. Major Depressive Disorder in Adolescence: A Brief Review of the Recent Treatment Literature. *Canadian Journal of Psychiatry* 2003;**48**:600-6.

Miller LJ, Mittenberg W. Brief cognitive behavioral interventions in mild traumatic brain injury. *Applied Neuropsychology* 1998;**5**:172-83.

Mitchell JE, Raymond NC. Cognitive-behavioral therapy in treatment of bulimia nervosa. In: Halmi K, editor. *Psychobiology and treatment of anorexia nervosa and bulimia nervosa.* Arlington, VA: American Psychiatric Association; US; 1992:307-27.

Mitchell JE, Roerig J, Steffen K. An update on treatment strategies for bulimia nervosa. In: Latzer DSaY, editor. *Treatment and recovery of eating disorders*Hauppauge, NY: Nova Science Publishers; US; 2012:27-39.

Mitte K. Meta-analysis of cognitive-behavioral treatments for generalized anxiety disorder: A comparison with pharmacotherapy. *Psychological Bulletin* 2005;**131**:785-95.

Mitte K. A meta-analysis of the efficacy of psycho- and pharmacotherapy in panic disorder with and without agoraphobia. *Journal of Affective Disorders* 2005;**88**:27-45.

Miziou S, Tsitsipa E, Moysidou S, Karavelas V, Dimelis D, Polyzoidou V*, et al.* Psychosocial treatment and interventions for bipolar disorder: A systematic review. *Annals of General Psychiatry* 2015;**14**.

Mobini S, Grant A. Clinical implications of attentional bias in anxiety disorders: An integrative literature review. *Psychotherapy: Theory, Research, Practice, Training* 2007;**44**:450-62.

Molinuevo B, Batista-Miranda JE. Under the tip of the iceberg: Psychological factors in incontinence. *Neurourology and Urodynamics* 2012;**31**:669-71.

Mulcahy RA, Blue RS, Vardiman JL, Castleberry TL, Vanderploeg JM. Screening and mitigation of layperson anxiety in aerospace environments. *Aerospace Medicine and Human Performance* 2016;**87**:882-9.

Muller A, Mitchell JE, De Zwaan M. Compulsive buying. *American Journal on Addictions* 2015;**24**:132-7.

Musiat P, Tarrier N. Collateral outcomes in e-mental health: a systematic review of the evidence for added benefits of computerized cognitive behavior therapy interventions for mental health. *Psychological Medicine* 2014;**44**:3137-50.

Mychailyszyn MP. *School-based interventions for anxious and depressed youth: A meta-analysis of outcomes*: Temple University; 2012.

Mychailyszyn MP, Brodman DM, Read KL, Kendall PC. Cognitive-behavioral school-based interventions for anxious and depressed youth: A meta-analysis of outcomes. *Clinical Psychology: Science and Practice* 2012;**19**:129-53.

Nalajala N, Walls K, Hili E. Insomnia in chronic lower back pain: Non-pharmacological physiotherapy interventions. *International Journal of Therapy & Rehabilitation* 2013;**20**:510-6.

Nardi B, Francesconi G, Catena-Dell'Osso M, Bellantuono C. Adolescent depression: Clinical features and therapeutic strategies. *European Review for Medical and Pharmacological Sciences* 2013;**17**:1546-51.

Nardi B, Massei M, Arimatea E, Moltedo-Perfetti A. Effectiveness of group CBT in treating adolescents with depression symptoms: a critical review. *International Journal of Adolescent Medicine and Health* 2017;**29**.

Neuderth S, Jabs B, Schmidtke A. Strategies for reducing test anxiety and optimizing exam preparation in German university students: A prevention-oriented pilot project of the University of Wurzburg. *Journal of Neural Transmission* 2009;**116**:785-90.

Nicholas MK, Molloy AR, Brooker C. Using opioids with persisting noncancer pain: A biopsychosocial perspective. *Clinical Journal of Pain* 2006;**22**:137-46.

Nicholl C, Thompson A. The psychological treatment of post traumatic stress disorder (PTSD) in adult refugees: a review of the current state of psychological therapies. *Journal of Mental Health* 2004;**13**:351-62.

Nordin M, Balague F, Cedraschi C. Nonspecific lower-back pain: Surgical versus nonsurgical treatment. *Clinical Orthopaedics and Related Research* 2006;**443()**:156-67.

Norman RMG, Townsend LA. Cognitive-behavioural therapy for psychosis: A status report. *Canadian Journal of Psychiatry* 1999;**44**:245-52.

Norris AL, Marcus DK. Cognition in health anxiety and hypochondriasis: Recent advances. *Current Psychiatry Reviews* 2014;**10**:44-9.

Norton PJ, Price EC. A meta-analytic review of adult cognitive-behavioral treatment outcome across the anxiety disorders. *Journal of Nervous & Mental Disease* 2007;**195**:521-31.

O'Connor K. Research in young people at ultra-high risk for psychosis: A review of the current evidence. *Irish Journal of Psychological Medicine* 2013;**30**:77-89.

Oei TP, Free ML. Do cognitive behaviour therapies validate cognitive models of mood disorders? A review of the empirical evidence. *International Journal of Psychology* 1995;**30**:145-80.

Olatunji BO, Cisler JM, Deacon BJ. Efficacy of cognitive behavioral therapy for anxiety disorders: a review of meta-analytic findings. *Psychiatric Clinics of North America* 2010;**33**:557-77.

Olatunji BO, Cisler JM, Tolin DF. A meta-analysis of the influence of comorbidity on treatment outcome in the anxiety disorders. *Clinical Psychology Review* 2010;**30**:642-54.

Olivares JM, Sermon J, Hemels M, Schreiner A. Definitions and drivers of relapse in patients with schizophrenia: A systematic literature review. *Annals of General Psychiatry Vol 12 2013, ArtID 32* 2013;**12**.

O'Neal P, Jackson A, McDermott F. A Review of the Efficacy and Effectiveness of Cognitive-Behaviour Therapy and Short-Term Psychodynamic Therapy in the Treatment of Major Depression: Implications for Mental Health Social Work Practice. *Australian Social Work* 2014;**67**:197-213.

O'Reilly R, Wilkes L, Luck L, Jackson D. The efficacy of family support and family preservation services on reducing child abuse and neglect: what the literature reveals. *Journal of Child Health Care* 2010;**14**:82-94.

Ost LG. Cognitive behavior therapy for anxiety disorders: 40 years of progress. *Nordic Journal of Psychiatry* 2008;**62**:5-10.

Otte C. Cognitive behavioral therapy in anxiety disorders: Current state of the evidence. *Dialogues in Clinical Neuroscience* 2011;**13**:413-21.

Parikh SV, Kusumakar V, Haslam DR, Matte R, Sharma V, Yatham LN. Psychosocial interventions as an adjunct to pharmacotherapy in bipolar disorder. *Canadian Journal of Psychiatry - Revue Canadienne de Psychiatrie* 1997;**42 Suppl 2**:74S-8S.

Parikh SV, Quilty LC, Ravitz P, Rosenbluth M, Pavlova B, Grigoriadis S*, et al.* Canadian Network for Mood and Anxiety Treatments (CANMAT) 2016 Clinical Guidelines for the Management of Adults with Major Depressive Disorder: Section 2. Psychological Treatments. *Canadian Journal of Psychiatry - Revue Canadienne de Psychiatrie* 2016;**61**:524-39.

Parikh SV, Segal ZV, Grigoriadis S, Ravindran AV, Kennedy SH, Lam RW*, et al.* Canadian Network for Mood and Anxiety Treatments (CANMAT) Clinical guidelines for the management of major depressive disorder in adults. II. Psychotherapy alone or in combination with antidepressant medication. *Journal of Affective Disorders* 2009;**117**:S15-S25.

Parker G, Fletcher K. Treating depression with the evidence-based psychotherapies: A critique of the evidence. *Acta Psychiatrica Scandinavica* 2007;**115**:352-9.

Parker G, Parker I, Brotchie H, Stuart S. Interpersonal psychotherapy for depression? The need to define its ecological niche. *Journal of Affective Disorders* 2006;**95**:1-11.

Parker G, Roy K, Eyers K. Cognitive behavior therapy for depression? Choose horses for courses. *American Journal of Psychiatry* 2003;**160**:825-34.

Parker GB, Crawford J, Hadzi-Pavlovic D. Quantified superiority of cognitive behaviour therapy to antidepressant drugs: A challenge to an earlier meta-analysis. *Acta Psychiatrica Scandinavica* 2008;**118**:91-7.

Patel N, A CdCW, Kellezi B. Reviewing outcomes of psychological interventions with torture survivors: Conceptual, methodological and ethical Issues. *Torture : quarterly journal on rehabilitation of torture victims and prevention of torture* 2016;**26**:2-16.

Patterson E, Wan YWT, Sidani S. Nonpharmacological nursing interventions for the management of patient fatigue: a literature review. *Journal of Clinical Nursing* 2013;**22**:2668-78.

Pattison S, Harris B. Counselling children and young people: a review of the evidence for its effectiveness. *Counselling & Psychotherapy Research* 2006;**6**:233-7.

Pavan C, Simonato P, Marini M, Mazzoleni F, Pavan L, Vindigni V. Psychopathologic aspects of body dysmorphic disorder: A literature review. *Aesthetic Plastic Surgery* 2008;**32**:473-84.

Pearsall DF. Psychotherapy outcome research in child psychiatric disorders. *Canadian Journal of Psychiatry - Revue Canadienne de Psychiatrie* 1997;**42**:595-601.

Pearson FS, Lipton DS, Cleland CM, Yee DS. The effects of behavioral/cognitive-behavioral programs on recidivism. *Crime & Delinquency* 2002;**48**:476-96.

Penckofer S, Doyle T, Byrn M, Lustman PJ. State of the Science: Depression and Type 2 Diabetes. *Western Journal of Nursing Research* 2014;**36**:1158-82.

Peng XD, Huang CQ, Chen LJ, Lu ZC. Cognitive behavioural therapy and reminiscence techniques for the treatment of depression in the elderly: a systematic review. *The Journal of international medical research* 2009;**37**:975-82.

Perry JC, Banon E, Ianni F. Effectiveness of psychotherapy for personality disorders. *American Journal of Psychiatry* 1999;**156**:1312-21.

Petrocelli JV. Effectiveness of group cognitive-behavioral therapy for general symptomatology: A meta-analysis. *Journal for Specialists in Group Work* 2002;**27**:92-115.

Pfammatter M, Junghan UM, Brenner HD. Efficacy of psychological therapy in schizophrenia: conclusions from meta-analyses. *Schizophrenia Bulletin* 2006;**32 Suppl 1**:S64-80.

Pheng TL, Hassan S. A review of psychotherapy as add-on treatment to pharmacotherapy for bipolar disorder. *Research Journal of Medical Sciences* 2014;**8**:99-108.

Pinquart M, Sorensen S. How effective are psychotherapeutic and other psychosocial interventions with older adults? A meta-analysis. *Journal of Mental Health and Aging* 2001;**7**:207-43.

Pinquart M, Sorensen S. Helping caregivers of persons with dementia: which interventions work and how large are their effects? . *International Psychogeriatrics* 2006;**18**:577-95.

Pinto TF, Da Silva FGC, De Bruin VMS, De Bruin PFC. Night eating syndrome: How to treat it? *Revista da Associacao Medica Brasileira* 2016;**62**:701-7.

Piotrowski C. Chronic Pain in the Elderly: Mapping the Mental Health Literature. *Journal of Instructional Psychology* 2014;**41**:16-8.

Podea D, Suciu R, Suciu C, Ardelean M. An update on the cognitive behavior therapy of obsessive compulsive disorder in adults. *Journal of Cognitive and Behavioral Psychotherapies* 2009;**9**:221-33.

Poiraudeau S, Rannou F, Revel M. Functional restoration programs for low back pain: a systematic review. *Annales de Readaptation et de Medecine Physique* 2007;**50**:425-9.

Polackwich AS, Shoskes DA. Chronic prostatitis/chronic pelvic pain syndrome: a review of evaluation and therapy. *Prostate Cancer & Prostatic Diseases* 2016;**19**:132-8.

Polaschek DL, Collie RM. Rehabilitating serious violent adult offenders: An empirical and theoretical stocktake. *Psychology, Crime & Law* 2004;**10**:321-34.

Ponniah K, Hollon SD. Empirically supported psychological interventions for social phobia in adults: A qualitative review of randomized controlled trials. *Psychological Medicine* 2008;**38**:3-14.

Portzky G, van Heeringen K. Deliberate self-harm in adolescents. *Current Opinion in Psychiatry* 2007;**20**:337-42.

Pospos S, Young IT, Downs N, Iglewicz A, Depp C, Chen JY*, et al.* Web-Based Tools and Mobile Applications To Mitigate Burnout, Depression, and Suicidality Among Healthcare Students and Professionals: a Systematic Review. *Academic Psychiatry* 2018;**42**:109-20.

Pospos S, Young IT, Downs N, Iglewicz A, Depp C, Chen JY*, et al.* Web-Based Tools and Mobile Applications To Mitigate Burnout, Depression, and Suicidality Among Healthcare Students and Professionals: a Systematic Review. *Academic psychiatry : the journal of the American Association of Directors of Psychiatric Residency Training and the Association for Academic Psychiatry* 2018;**42**:109-20.

Post MWM, van Leeuwen CMC. Psychosocial issues in spinal cord injury: a review. *Spinal Cord* 2012;**50**:382-9.

Poynter BA, Hunter JJ, Coverdale JH, Kempinsky CA. Hard to swallow: A systematic review of deliberate foreign body ingestion. *General Hospital Psychiatry* 2011;**33**:518-24.

Prajapati AR. Pharmacology vs. psychotherapy in the management of depression and anxiety disorders. *International Journal of Psychotherapy* 2014;**18**:50-61.

Pratchett LC, Daly K, Bierer LM, Yehuda R. New approaches to combining pharmacotherapy and psychotherapy for posttraumatic stress disorder. *Expert Opinion on Pharmacotherapy* 2011;**12**:2339-54.

Price A, Hotopf M. The treatment of depression in patients with advanced cancer undergoing palliative care. *Current opinion in supportive and palliative care* 2009;**3**:61-6.

Prout SM, Prout H. A meta-analysis of school-based studies of counseling and psychotherapy: An update. *Journal of School Psychology* 1998;**36**:121-36.

Pull CB. Combined pharmacotherapy and cognitive-behavioural therapy for anxiety disorders. *Current Opinion in Psychiatry* 2007;**20**:30-5.

Pull CB. Current empirical status of acceptance and commitment therapy. *Current Opinion in Psychiatry* 2009;**22**:55-60.

Pull CB, Damsa C. Pharmacotherapy of panic disorder. *Neuropsychiatric Disease and Treatment* 2008;**4**:779-95.

Putnam FW. Ten-year research update review: Child sexual abuse. *Journal of the American Academy of Child and Adolescent Psychiatry* 2003;**42**:269-78.
Rachbeiscl J, Scott J, Dixon L. Co-occurring severe mental illness and substance use disorders: A review of recent research. *Psychiatric Services* 1999;**50**:1427-34.

Radziwon CD, Lackner JM. Cognitive Behavioral Therapy for IBS: How Useful, How Often, and How Does It Work? *Current Gastroenterology Reports* 2017;**19**.

Ramacciotti CE, Coli E, Marazziti D, Segura-Garcia C, Brambilla F, Piccinni A*, et al.* Therapeutic options for binge eating disorder. *Eating and Weight Disorders* 2013;**18**:3-9.

Rathod S, Hansen L, Kingdon D. Insight in Psychosis and Cognitive Behaviour Therapy. In: Abelian M, editor. *Focus on Psychotherapy Research.* Hauppauge, NY: Nova Science Publishers; US; 2005:51-67.

Rathod S, Kingdon D, Weiden P, Turkington D. Cognitive-behavioral therapy for medication-resistant schizophrenia: a review. *Journal of Psychiatric Practice* 2008;**14**:22-33.

Reinecke MA, Ryan NE, DuBois DL. Cognitive-behavioral therapy of depression and depressive symptoms during adolescence: a review and meta-analysis. *Journal of the American Academy of Child and Adolescent Psychiatry* 1998;**37**:26-34.

Richmond J, Berman BM, Docherty JP, Goldstein LB, Kaplan G, Keil JE*, et al.* Integration of behavioral and relaxation approaches into the treatment of chronic pain and insomnia. *Journal of the American Medical Association* 1996;**276**:313-8.

Ridings LE, Moreland AD, Petty KH. Implementing trauma-focused CBT for children of veterans in the VA: Providing comprehensive services to veterans and their families. *Psychological services* 2018;**30**:30.

Riemann D, Perlis ML. The treatments of chronic insomnia: A review of benzodiazepine receptor agonists and psychological and behavioral therapies. *Sleep Medicine Reviews* 2009;**13**:205-14.

Rimbaut S, Van Gutte C, Van Brabander L, Vanden Bossche L. Chronic fatigue syndrome - an update. *Acta Clinica Belgica: International Journal of Clinical and Laboratory Medicine* 2016;**71**:273-80.

Rimes KA, Chalder T. Treatments for chronic fatigue syndrome. *Occupational Medicine* 2005;**55**:32-9.

Ritz T, Meuret AE, Trueba AF, Fritzsche A, von Leupoldt A. Psychosocial factors and behavioral medicine interventions in asthma. *Journal of Consulting and Clinical Psychology* 2013;**81**:231-50.

Rodrigo C, Rajapakse S, Jayananda G. The 'antisocial' person: An insight in to biology, classification and current evidence on treatment. *Annals of General Psychiatry Vol 9 2010, ArtID 31* 2010;**9**.

Roepke AM, Seligman ME. Depression and prospection. *The British journal of clinical psychology / the British Psychological Society* 2016;**55**:23-48.

Roffman JL, Marci CD, Glick DM, Dougherty DD, Rauch SL. Neuroimaging and the functional neuroanatomy of psychotherapy. *Psychological Medicine* 2005;**35**:1385-98.

Rogers-Nicastro J. *A meta-analytic review of play therapy outcomes and the role of age: Implications for school psychologists*: St John's University (New York); 2006.

Ronconi JM, Shiner B, Watts BV. Inclusion and Exclusion Criteria in Randomized Controlled Trials of Psychotherapy for PTSD. *Journal of Psychiatric Practice* 2014;**20**:25-37.

Roshanaei-Moghaddam B, Pauly MC, Atkins DC, Baldwin SA, Stein MB, Roy-Byrne P. Relative effects of CBT and pharmacotherapy in depression versus anxiety: Is medication somewhat better for depression, and CBT somewhat better for anxiety? *Depression and Anxiety* 2011;**28**:560-7.

Rostain AL. Attention-deficit/hyperactivity disorder in adults: evidence-based recommendations for management. *Postgraduate Medicine* 2008;**120**:27-38.

Roth AD, Pilling S, Turner J. Therapist training and supervision in clinical trials: Implications for clinical practice. *Behavioural and Cognitive Psychotherapy* 2010;**38**:291-302.

Rotheram-Fuller E, MacMullen L. Cognitive-behavioral therapy for children with autism spectrum disorders. *Psychology in the Schools* 2011;**48**:263-71.

Rowa K, Antony MM. Psychological treatments for social phobia. *Canadian Journal of Psychiatry* 2005;**50**:308-16.

Rozental A, Magnusson K, Boettcher J, Andersson G, Carlbring P. For Better or Worse: An Individual Patient Data Meta-Analysis of Deterioration Among Participants Receiving Internet-Based Cognitive Behavior Therapy. *Journal of Consulting and Clinical Psychology* 2017;**85**:160-77.

Rubin A, Miao Y. Within-Group Effect Size Benchmarks for Cognitive--Behavioral Therapy in the Treatment of Adult Depression. *Social Work Research* 2017;**41**:135-44.

Rubinchik SM, Kablinger AS, Gardner JS. Medications for panic disorder and generalized anxiety disorder during pregnancy. *Primary Care Companion to the Journal of Clinical Psychiatry* 2005;**7**:100-5.

Rustad JK, Stern TA, Hebert KA, Musselman DL. Diagnosis and treatment of depression in patients with congestive heart failure: A review of the literature. *Primary Care Companion to the Journal of Clinical Psychiatry* 2013;**15**.

Rutherford L, Couturier J. A review of psychotherapeutic interventions for children and adolescents with eating disorders. *Journal of the Canadian Academy of Child and Adolescent Psychiatry* 2007;**16**:153-7.

Saini M. A meta-analysis of the psychological treatment of anger: developing guidelines for evidence-based practice. *The journal of the American Academy of Psychiatry and the Law* 2009;**37**:473-88.

Sakinofsky I. The aftermath of suicide: managing survivors' bereavement. *Canadian Journal of Psychiatry* 2007;**52**:129S-36S.

Salmoiraghi A, Sambhi R. Early termination of cognitive-behavioural interventions: Literature review. *Psychiatrist* 2010;**34**:529-32.

Sampaio FMC, Sequeira CAdC, Lluch Canut MT. Nursing psychotherapeutic interventions: a review of clinical studies. *Journal of Clinical Nursing* 2015;**24**:2096-105.

Samson JE, Tanner-Smith EE. Single-Session Alcohol Interventions for Heavy Drinking College Students: A Systematic Review and Meta-Analysis. *Journal of Studies on Alcohol and Drugs* 2015;**76**:530-43.

Schaffer A, McIntosh D, Goldstein BI, Rector NA, McLntyre RS, Beaulieu S*, et al.* The Canadian Network for Mood and Anxiety Treatments (CANMAT) task force recommendations for the management of patients with mood disorders and comorbid anxiety disorders. *Annals of Clinical Psychiatry* 2012;**24**:6-22.

Schoenwald SK, Garland AF. A review of treatment adherence measurement methods. *Psychological Assessment* 2013;**25**:146-56.

Schwalbe C, Gearing R. The moderating effect of adherence-promoting interventions with clients on evidence-based practices for children and adolescents with mental health problems. *American Journal of Orthopsychiatry* 2012;**82**:146-55.

Scogin F, Welsh D, Hanson A, Stump J, Coates A. Evidence-based psychotherapies for depression in older adults. *Clinical Psychology: Science and Practice* 2005;**12**:222-37.

Segal ZV, Whitney DK, Lam RW, Canmat Depression Work G. Clinical guidelines for the treatment of depressive disorders. III. Psychotherapy. *Canadian Journal of Psychiatry - Revue Canadienne de Psychiatrie* 2001;**46 Suppl 1**:29S-37S.

Sharma L. Clinical. *Osteoarthritis and Cartilage* 2015;**23**:A24.

Shen Y, Huang JY, Li J, Liu CF. Excessive Daytime Sleepiness in Parkinson's Disease: Clinical Implications and Management. *Chinese Medical Journal* 2018;**131**:974-81.

Shen YH, Nahas R. Complementary and alternative medicine for treatment of irritable bowel syndrome. *Canadian Family Physician* 2009;**55**:143-8.

Siegel P, Tencza M, Apodaca B, Poole JL. Effectiveness of Occupational Therapy Interventions for Adults With Rheumatoid Arthritis: A Systematic Review. *The American journal of occupational therapy : official publication of the American Occupational Therapy Association* 2017;**71**:7101180050p.

Silva JAMD, Siegmund G, Bredemeier J. Crisis interventions in online psychological counseling Intervencoes em crise nos atendimentos psicologicos online. *Trends in Psychiatry and Psychotherapy* 2015;**37**:171-82.

Sinclair D, Adams CE. Treatment resistant schizophrenia: A comprehensive survey of randomised controlled trials. *BMC Psychiatry* 2014;**14**.

Skeem JL, Steadman HJ, Manchak SM. Applicability of the Risk-Need-Responsivity Model to Persons With Mental Illness Involved in the Criminal Justice System. *Psychiatric Services* 2015;**66**:916-22.

Skriner LC, Chu BC, Kaplan M, Bodden DHM, Bogels SM, Kendall PC*, et al.* Trajectories and predictors of response in youth anxiety CBT: Integrative data analysis. *Journal of Consulting & Clinical Psychology* 2019;**87**:198-211.

Slater J, Townend M. Cognitive behaviour therapy for psychosis in high secure services: An exploratory hermeneutic review of the international literature. *Behavioural and Cognitive Psychotherapy* 2016;**44**:652-72.

Slobodin O, de Jong JTVM. Mental health interventions for traumatized asylum seekers and refugees: What do we know about their efficacy? *International Journal of Social Psychiatry* 2015;**61**:17-26.

Spattini L, Rioli G, Longo F, Ferrari S, Galeazzi GM. An update on current clinical management of eating disorders. *Minerva Psichiatrica* 2017;**58**:54-69.

Speisman BB, Storch EA, Abramowitz JS. Postpartum Obsessive-Compulsive Disorder. *JOGNN: Journal of Obstetric, Gynecologic & Neonatal Nursing* 2011;**40**:680-90.

Spielmans GI, Pasek LF, McFall JP. What are the active ingredients in cognitive and behavioral psychotherapy for anxious and depressed children? A meta-analytic review. *Clinical Psychology Review* 2007;**27**:642-54.

Stacciarini JR, O'Keeffe M, Mathews M. Group therapy as treatment for depressed Latino women: a review of the literature. *Issues in Mental Health Nursing* 2007;**28**:473-88.

Staub C. Concept of diverse sleep treatments in physiotherapy. *European Journal of Physiotherapy* 2018;**21**:177-84.

Stewart RE, Chambless DL. Cognitive-Behavioral Therapy for Adult Anxiety Disorders in Clinical Practice: A Meta-Analysis of Effectiveness Studies. *Journal of Consulting and Clinical Psychology* 2009;**77**:595-606.

Stuart S, Bowers WA. Cognitive therapy with inpatients: Review and meta-analysis. *Journal of Cognitive Psychotherapy: An International Quarterly* 1995;**9**:85-92.

Sturmey P. Behavioral activation is an evidence-based treatment for depression. *Behavior Modification* 2009;**33**:818-29.

Sturmey P. Treatment of psychopathology in people with intellectual and other disabilities. *Canadian Journal of Psychiatry* 2012;**57**:593-600.

Sukhodolsky DG. *Cognitive-behavioral treatment programs for anger-related problems in children and adolescents: A meta-analytic study*: Hofstra University; 1998.

Sukhodolsky DG, Kassinove H, Gorman BS. Cognitive-behavioral therapy for anger in children and adolescents: a meta-analysis. *Aggression & Violent Behavior* 2004;**9**:247-.

Sun M, Rith-Najarian LR, Williamson TJ, Chorpita BF. Treatment Features Associated with Youth Cognitive Behavioral Therapy Follow-Up Effects for Internalizing Disorders: A Meta-Analysis. *Journal of Clinical Child & Adolescent Psychology* 2018;**00**:1-15.

Sussman S, Sun P, Dent CW. A meta-analysis of teen cigarette smoking cessation. *Health Psychology* 2006;**25**:549-57.

Sveinsdottir V, Eriksen HR, Reme SE. Assessing the role of cognitive behavioral therapy in the management of chronic nonspecific back pain. *Journal of Pain Research* 2012;**5**:371-80.

Syka A. Depression in Pregnancy and Ways of Dealing. *International Journal of Caring Sciences* 2015;**8**:231-7.

Szentagotai A, David D. The efficacy of cognitive-behavioral therapy in bipolar disorder: A quantitative meta-analysis. *Journal of Clinical Psychiatry* 2010;**71**:66-72.

Taddio A, Appleton M, Bortolussi B, Chambers C, Dubey V, Halperin S*, et al.* Reducing the pain of childhood immunization - An evidence-based clinical practice guideline. *Canadian Journal of Infectious Diseases and Medical Microbiology* 2010;**21(4)**:179.

Tavares H, Lobo DSS, Fuentes D, Black DW. Compulsive buying disorder: A review and a Case Vignette. *Revista Brasileira de Psiquiatria* 2008;**30**:S16-S23.

Tavares H, Zilberman ML, El-Guebaly N. Are there cognitive and behavioural approaches specific to the treatment of pathological gambling? *Canadian Journal of Psychiatry* 2003;**48**:22-7.

Taylor S. Meta-analysis of cognitive-behavioral treatments for social phobia. *Journal of Behavior Therapy and Experimental Psychiatry* 1996;**27**:1-9.

Taylor S, Asmundson GJG, Coons MJ. Current directions in the treatment of hypochondriasis. *Journal of Cognitive Psychotherapy* 2005;**19**:285-304.

Thapar A, Collishaw S, Potter R, Thapar AK. Managing and preventing depression in adolescents. *BMJ (Online)* 2010;**340**:254-8.

Thorpe CT, Fahey LE, Johnson H, Deshpande M, Thorpe JM, Fisher EB. Facilitating Healthy Coping in Patients With Diabetes: A Systematic Review. *Diabetes Educator* 2013;**39**:33-52.

Tiffin PA, Welsh P. Practitioner Review: Schizophrenia spectrum disorders and the at-risk mental state for psychosis in children and adolescents - evidence-based management approaches. *Journal of Child Psychology & Psychiatry* 2013;**54**:1155-75.

Tolin DF. Beating a dead dodo bird: Looking at signal vs. noise in cognitive-behavioral therapy for anxiety disorders. *Clinical Psychology: Science and Practice* 2014;**21**:351-62.

Tsiros MD, Sinn N, Coates AM, Howe PRC, Buckley JD. Treatment of adolescent overweight and obesity. *European Journal of Pediatrics* 2008;**167**:9-16.

Turchiano TP. *A meta-analysis of behavioral and cognitive therapies for children and adolescents with attention deficit hyperactivity and/or impulsivity disorders*: Hofstra University; 1999.
Turner JA. Educational and behavioral interventions for back pain in primary care. *Spine* 1996;**21**:2851-9.

Twohig MP, Levin ME. Acceptance and Commitment Therapy as a Treatment for Anxiety and Depression: A Review. *Psychiatric Clinics of North America* 2017;**40**:751-70.

Ulloa-Flores RE, de la Pena-Olvera F, Nogaies-lmoca I. The multimodal treatment for children and adolescents with depression. *Salud Mental* 2011;**34**:403-7.

Usher AM, Stewart LA. Effectiveness of correctional programs with ethnically diverse offenders: a meta-analytic study. *International Journal of Offender Therapy and Comparative Criminology* 2014;**58**:209-30.

van der Feltz-Cornelis CM, Hoedeman R, Keuter EJ, Swinkels JA. Presentation of the Multidisciplinary Guideline Medically Unexplained Physical Symptoms (MUPS) and Somatoform Disorder in the Netherlands: disease management according to risk profiles. *Journal of Psychosomatic Research* 2012;**72**:168-9.

van der Klink JJL, Blonk RWB, Schene AH, van Dijk FJH. The benefits of interventions for work-related stress. *American Journal of Public Health* 2001;**91**:270-6.

van Emmerik AA, Reijntjes A, Kamphuis JH. Writing therapy for posttraumatic stress: a meta-analysis. *Psychotherapy & Psychosomatics* 2013;**82**:82-8.

van Ingen DJ, Freiheit SR, Vye CS. From the lab to the clinic: Effectiveness of cognitive-behavioral treatments for anxiety disorders. *Professional Psychology: Research and Practice* 2009;**40**:69-74.

Vanderploeg RD, Belanger HG, Curtiss G, Bowles AO, Cooper DB. Reconceptualizing rehabilitation of individuals with chronic symptoms following mild traumatic brain injury. *Rehabilitation Psychology* 2018;**20**:20.

Vasa RA. Assessment and management of anxiety in youth with autism spectrum disorder. *Journal of the American Academy of Child and Adolescent Psychiatry* 2016;**55(10)**:S98.

Vieta E, Colom F. Psychological interventions in bipolar disorder: From wishful thinking to an evidence-based approach. *Acta Psychiatrica Scandinavica* 2004;**110 (Suppl. 422)**:34-8.

Vieta E, Pacchiarotti I, Scott J, Sanchez-Moreno J, Di Marzo S, Colom F. Evidence-based research on the efficacy of psychologic interventions in bipolar disorders: A critical review. *Current Psychiatry Reports* 2005;**7**:449-55.

Vigil TM, Orellana AF, Garcia RR, Correa MD. Cognitive behavioral therapy and negative symptoms in schizophrenia. *Salud Mental* 2015;**38**:371-7.

Vitoula K, Venneri A, Varrassi G, Paladini A, Sykioti P, Adewusi J, *et al.* Behavioral Therapy Approaches for the Management of Low Back Pain: An Up-To-Date Systematic Review. *Pain and Therapy* 2018;**16**:16.

Vittengl JR, Jarrett RB, Weitz E, Hollon SD, Twisk J, Cristea I, *et al.* Divergent Outcomes in Cognitive-Behavioral Therapy and Pharmacotherapy for Adult Depression. *American Journal of Psychiatry* 2016;**173**:481-90.

Volavka J. Violence in schizophrenia and bipolar disorder. *Psychiatria Danubina* 2013;**25**:24-33.

Wagner JJ. *A meta-analysis/literature review comparing the effectiveness of SSRI antidepressants, cognitive behavioral therapy, and placebo for the treatment of depression*: Carlos Albizu University; 2005.

Waldron HB, Kaminer Y. On the learning curve: The emerging evidence supporting cognitive-behavioral therapies for adolescent substance abuse. *Addiction* 2004;**99**:93-105.

Walitza S, Melfsen S, Jans T, Zellmann H, Wewetzer C, Warnke A. Obsessive-compulsive disorder in children and adolescents. *Deutsches Arzteblatt International* 2011;**108**:173-9.

Walther L, Gantner A, Heinz A, Majic T. Evidence-based Treatment Options in Cannabis Dependency. *Deutsches Arzteblatt International* 2016;**113**:653-9.

Wang X, Huang S, Qi HB. Comparative efficacy and acceptability of seven augmentation agents for treatment-resistant depression: A multiple-treatments meta-analysis. *African Journal of Psychiatry (South Africa)* 2014;**20**:71-6.

Wellen DG. *A meta-analysis of single-subject studies of therapies for children and adolescents with aggression*: St John's University (New York); 1998.

Wellington J. Noninvasive and alternative management of chronic low back pain (efficacy and outcomes). *Neuromodulation* 2014;**17**:24-30.

Whitbread J, McGown A. The treatment of bulimia nervosa: What is effective? A meta-analysis. *Indian Journal of Clinical Psychology* 1994;**21**:32-44.

Wiers RW, Boffo M, Field M. What's in a Trial? On the Importance of Distinguishing Between Experimental Lab Studies and Randomized Controlled Trials: The Case of Cognitive Bias Modification and Alcohol Use Disorders. *Journal of Studies on Alcohol & Drugs* 2018;**79**:333-43.

Wijeratne C, Sachdev P. Treatment-resistant depression: critique of current approaches. *Australian & New Zealand Journal of Psychiatry* 2008;**42**:751-62.

Wild D, von Maltzahn R, Brohan E, Christensen T, Clauson P, Gonder-Frederick L. A critical review of the literature on fear of hypoglycemia in diabetes: Implications for diabetes management and patient education. *Patient Education and Counseling* 2007;**68**:10-5.

Wilks CR, Zieve GG, Lessing HK. Are Trials of Computerized Therapy Generalizable? A Multidimensional Meta-analysis. *Telemedicine journal and e-health : the official journal of the American Telemedicine Association* 2016;**22**:450-7.

Williams CR. *Long-term effectiveness of CBT for anxiety disorders: A meta-analytic review*: University of Hartford; 2016.

Williams NH. Optimising the psychological benefits of osteopathy. *International Journal of Osteopathic Medicine* 2007;**10**:36-41.

Wilson IR. Management of chronic pain through pain management programmes. *British Medical Bulletin* 2017;**124**:55-64.

Wurz A, Sungur MZ. Combining cognitive behavioural therapy and pharmacotherapy in the treatment of anxiety disorders: True gains or false hopes? *Klinik Psikofarmakoloji Bulteni* 2009;**19**:436-47.

Zaider T, Heimberg R. Non-pharmacologic treatments for social anxiety disorder. *Acta Psychiatrica Scandinavica* 2003;**108**:72-84.

Zaretsky AE, Rizvi S, Parikh SV. How well do psychosocial interventions work in bipolar disorder? *Canadian Journal of Psychiatry* 2007;**52**:14-21.

Zenner HP, Delb W, Kroner-Herwig B, Jager B, Peroz I, Hesse G*, et al.* A multidisciplinary systematic review of the treatment for chronic idiopathic tinnitus. *European Archives of Oto-Rhino-Laryngology* 2017;**274**:2079-91.

Zinzow HM, Jeffirs SM. Driving Aggression and Anxiety: Intersections, Assessment, and Interventions. *Journal of Clinical Psychology* 2018;**74**:43-82.

c. References of studies excluded due no CBT only data/comparison (470)

Aguilera M. Post-surgery support and the long-term success of bariatric surgery. *Practice Nursing* 2014;**25**:455-9

Alanazi MH, Parent EC, Dennett E. Effect of stabilization exercise on back pain, disability and quality of life in adult with scoliosis: a systematic review. *European journal of physical & rehabilitation medicine* 2017;**16**:16

Albert U, Marazziti D, Di Salvo G, Solia F, Rosso G, Maina G. A systematic review of evidence-based treatment strategies for obsessive-compulsive disorder resistant to first-line pharmacotherapy. *Current Medicinal Chemistry* 2017;**22**:22

Alcantara-Silva TR, Freitas-Junior R, Freitas NM, Machado GD. Fatigue related to radiotherapy for breast and/or gynaecological cancer: a systematic review. *Journal of Clinical Nursing* 2013;**22**:2679-86

Alessi C, Vitiello MV. Insomnia (primary) in older people: non-drug treatments. *Clinical Evidence* 2015;**13**:13

Alosaimi FD, Baker B. Clinical review of treatment options for major depressive disorder in patients with coronary heart disease. *Saudi Medical Journal* 2012;**33**:1159-68

Altena AM, Brilleslijper-Kater SN, Wolf JL. Effective interventions for homeless youth: a systematic review. *American Journal of Preventive Medicine* 2010;**38**:637-45

Alvarez-Jimenez M, Alcazar-Corcoles M, Gonzalez-Blanch C, Bendall S, McGorry P, Gleeson J. Online, social media and mobile technologies for psychosis treatment: A systematic review on novel user-led interventions. *Schizophrenia Research* 2014;**156**:96-106

Anderson RU, Wise D, Nathanson BH. Chronic Prostatitis and/or Chronic Pelvic Pain as a Psychoneuromuscular Disorder-A Meta-analysis. *Urology* 2018;**120**:23-9

Angel Garcia D, Martinez Nicolas I, Saturno Hernandez PJ. Clinical approach to fibromyalgia: Synthesis of Evidence-based recommendations, a systematic review. *Reumatologia Clinica* 2016;**12**:65-71

Arias E, Arce R, Vilarino M. Batterer intervention programmes: A meta-analytic review of effectiveness. *Psychosocial Intervention* 2013;**22**:153-60

Arico D, Raggi A, Ferri R. Cognitive Behavioral Therapy for Insomnia in Breast Cancer Survivors: A Review of the Literature. *Frontiers in Psychology* 2016;**7**:1162

Armelius B, Andreassen TH. Cognitive-behavioral treatment for antisocial behavior in youth in residential treatment. *Cochrane Database Syst Rev* 2007;**17(4)**:CD005650

A-Tjak JGL, Davis ML, Morina N, Powers MB, Smits JAJ, Emmelkamp PMG. A meta-analysis of the efficacy of acceptance and commitment therapy for clinically relevant mental and physical health problems. *Psychotherapy and Psychosomatics* 2015;**84**:30-6

Auty KM, Cope A, Liebling A. Psychoeducational programs for reducing prison violence: A systematic review. *Aggression and Violent Behavior* 2017;**33**:126-43

Badawy SM, Cronin RM, Hankins J, Crosby L, DeBaun M, Thompson AA, *et al.* Patient-Centered eHealth Interventions for Children, Adolescents, and Adults With Sickle Cell Disease: Systematic Review. *Journal of Medical Internet Research* 2018;**20**:e10940

Baez S, Hoch MC, Hoch JM. Evaluation of Cognitive Behavioral Interventions and Psychoeducation Implemented by Rehabilitation Specialists to Treat Fear-Avoidance Beliefs in Patients With Low Back Pain: A Systematic Review. *Archives of Physical Medicine & Rehabilitation* 2017;**14**:14

Baez S, Hoch MC, Hoch JM. Evaluation of Cognitive Behavioral Interventions and Psychoeducation Implemented by Rehabilitation Specialists to Treat Fear-Avoidance Beliefs in Patients With Low Back Pain: A Systematic Review. *Archives of Physical Medicine & Rehabilitation* 2018;**99**:2287-98

Bahrami HR, Hamedi S, Salari R, Noras M. Herbal Medicines for the Management of Irritable Bowel Syndrome: A Systematic Review. *Electronic Physician* 2016;**8**:2719-25

Baird E, Williams ACC, Hearn L, Amris K. Interventions for treating persistent pain in survivors of torture. *Cochrane Database Syst Rev* 2017;**18(8)**

Baker AL, Hiles SA, Thornton LK, Hides L, Lubman DI. A systematic review of psychological interventions for excessive alcohol consumption among people with psychotic disorders *Acta Psychiatrica Scandinavica* 2012;**126**:243-55

Baker GA, Brooks JL, Goodfellow L, Bodde N, Aldenkamp A. Treatments for non-epileptic attack disorder. *Cochrane Database Syst Rev* 2007;**(2)**:CD006370

Baliousis M, Rennoldson M, Snowden JA. Psychological interventions for distress in adults undergoing haematopoietic stem cell transplantation: a systematic review with meta-analysis. *Psycho-Oncology* 2016;**25**:400-11

Balkom AJ, Bakker A, Spinhoven P, Blaauw BM, Smeenk S, Ruesink B. A meta-analysis of the treatment of panic disorder with or without agoraphobia: a comparison of psychopharmacological, cognitive-behavioral, and combination treatments. *Journal of Nervous and Mental Disease* 1997;**185**:510-6

Barlow J, Bennett C, Midgley N, Larkin SK, Wei Y. Parent-infant psychotherapy for improving parental and infant mental health. *Cochrane Database Syst Rev* 2015;**8(1)**:CD010534

Barnason S, Zimmerman L, Young L. An integrative review of interventions promoting self-care of patients with heart failure *Journal of Clinical Nursing* 2012;**21**:448-75

Barnason S, Zimmerman L, Young L. An integrative review of interventions promoting self-care of patients with heart failure. *Journal of Clinical Nursing* 2012;**21**:448-75

Barnes TN, Smith SW, Miller MD. School-based cognitive-behavioral interventions in the treatment of aggression in the United States: A meta-analysis. *Aggression and Violent Behavior* 2014;**19**:311-21

Barrett S, Begg S, O'Halloran P, Kingsley M. Integrated motivational interviewing and cognitive behaviour therapy for lifestyle mediators of overweight and obesity in community-dwelling adults: a systematic review and meta-analyses. *BMC Public Health* 2018;**18**:1160

Belleville G, Cousineau H, Levrier K, St-Pierre-Delorme ME. Meta-analytic review of the impact of cognitive-behavior therapy for insomnia on concomitant anxiety *Clinical Psychology Review* 2011;**31**:638-52

Bennett C, Barlow J, Huband N, Smailagic N, Roloff V. Group-based parenting programs for improving parenting and psychosocial functioning: A systematic review. *Journal of the Society for Social Work and Research* 2013;**4**:300-32

Beynon S, Soares-Weiser K, Woolacott N, Duffy S, Geddes JR. Psychosocial interventions for the prevention of relapse in bipolar disorder: systematic review of controlled trials. *British Journal of Psychiatry* 2008;**192**:5-11

Binford MC, Kahana SY, Altice FL. A systematic review of antiretroviral adherence interventions for HIV-infected people who use drugs. *Current HIV/AIDS Reports* 2012;**9**:287-312

Binks CA, Fenton M, McCarthy L, Lee T, Adams CE, Duggan C. Psychological therapies for people with borderline personality disorder. *Cochrane Database Syst Rev* 2006;**25(1)**:CD005652

Bjornstad GJ, Montgomery P. Family therapy for attention-deficit disorder or attention-deficit/hyperactivity disorder in children and adolescents. *Cochrane Database Syst Rev* 2005;**18(2)**:CD005042

Blanchet C, Mathieu ME, St-Laurent A, Fecteau S, St-Amour N, Drapeau V. A Systematic Review of Physical Activity Interventions in Individuals with Binge Eating Disorders. *Current Obesity Reports* 2018;**7**:76-88

Bodryzlova Y, Audet JS, Bergeron K, O'Connor K. Group cognitive-behavioural therapy for hoarding disorder: Systematic review and meta-analysis. *Health & Social Care in the Community* 2018;**23**:23

Boniface S, Malet-Lambert I, Coleman R, Deluca P, Donoghue K, Drummond C*, et al.* The Effect of Brief Interventions for Alcohol Among People with Comorbid Mental Health Conditions: A Systematic Review of Randomized Trials and Narrative Synthesis. *Alcohol & Alcoholism* 2018;**53**:282-93

Borsay C. Anger management interventions for adults with learning disabilities living in the community: a review of recent (2000-2010) evidence. *British Journal of Learning Disabilities* 2013;**41**:38-44

Bougea A, Darviri C, Alexopoulos EC. A systematic review of randomized controlled interventions for parents' distress in pediatric leukemia. *Isrn Oncology Print* 2011;**2011**:959247

Bowker E, Dorstyn D. Hypnotherapy for disability-related pain: A meta-analysis. *Journal of Health Psychology* 2016;**21**:526-39

Bradley R, Greene J, Russ E, Dutra L, Westen D. A multidimensional meta-analysis of psychotherapy for PTSD. *American Journal of Psychiatry* 2005;**162**:214-27

Brazier J, Tumur I, Holmes M, Ferriter M, Parry G, Dent-Brown K*, et al.* Psychological therapies including dialectical behaviour therapy for borderline personality disorder: a systematic review and preliminary economic evaluation. *Health Technology Assessment* 2006;**10**:iii-131

Brettle A, Hill A, Jenkins P. Counselling in primary care: a systematic review of the evidence. *Counselling & Psychotherapy Research* 2008;**8**:207-14

Brewer M, Melnyk BM. Evidence-based practice. Effective coping/mental health interventions for critically ill adolescents: an evidence review. *Pediatric Nursing* 2007;**33**:361-73

Brok EC, Lok P, Oosterbaan DB, Schene AH, Tendolkar I, Van Eijndhoven PF. Infant-related intrusive thoughts of harm in the postpartum period: A critical review. *Journal of Clinical Psychiatry* 2017;**78**:e913-e23

Brooks JL, Baker GA, Goodfellow L, Bodde N, Aldenkamp A. Behavioural treatments for non-epileptic attack disorder. *Cochrane Database Syst Rev* 2007;**24(1)** CD006370

Brown JL, Vanable PA. Cognitive-behavioral stress management interventions for persons living with HIV: a review and critique of the literature. *Annals of Behavioral Medicine* 2008;**35**:26-40

Brox JI, Storheim K, Grotle M, Tveito TH, Indahl A, Eriksen HR. Systematic review of back schools, brief education, and fear-avoidance training for chronic low back pain. *Spine Journal* 2008;**8**:948-58

Brunner E, De Herdt A, Minguet P, Baldew SS, Probst M. Can cognitive behavioural therapy based strategies be integrated into physiotherapy for the prevention of chronic low back pain? A systematic review. *Disability and rehabilitation* 2013;**35**:1-10

Buchanan JA, Zakrzewska JM. Burning mouth syndrome. *BMJ clinical evidence* 2010:pii: 1301

Busse JW, Montori VM, Krasnik C, Patelis-Siotis I, Guyatt GH. Psychological intervention for premenstrual syndrome: A meta-analysis of randomized controlled trials. *Psychotherapy and Psychosomatics* 2009;**78**:6-15

Butler R, Radhakrishnan R. Dementia. *BMJ clinical evidence* 2012:pii: 1001

Cameron LC. *Treatment of bulimia nervosa: A meta-analysis*: The University of Mississippi; 1999.

Cape J, Barker C, Buszewicz M, Pistrang N. General practitioner psychological management of common emotional problems (I): Definitions and literature review. *British Journal of General Practice* 2000;**50**:313-8

Carson KV, Brinn MP, Peters M, Veale A, Esterman AJ, Smith BJ. Interventions for smoking cessation in Indigenous populations. *Cochrane Database Syst Rev* 2012;**18(1)**:CD009046

Carville SF, Arendt-Nielsen S, Bliddal H, Blotman F, Branco JC, Buskila D*, et al.* EULAR evidence-based recommendations for the management of fibromyalgia syndrome. *Annals of the Rheumatic Diseases* 2008;**67**:536-41

Casement MD, Swanson LM. A meta-analysis of imagery rehearsal for post-trauma nightmares: effects on nightmare frequency, sleep quality, and posttraumatic stress. *Clinical Psychology Review* 2012;**32**:566-74

Castelnuovo G, Giusti EM, Manzoni GM, Saviola D, Gatti A, Gabrielli S*, et al.* Psychological Treatments and Psychotherapies in the Neurorehabilitation of Pain: Evidences and Recommendations from the Italian Consensus Conference on Pain in Neurorehabilitation. *Frontiers in Psychology* 2016;**7**:115

Castillo-Bueno MD, Moreno-Pina JP, Martinez-Puente MV, Artiles-Suarez MM, Company-Sancho MC, Garcia-Andres MC*, et al.* Effectiveness of nursing intervention for adult patients experiencing chronic pain: a systematic review. *JBI Library of Systematic Reviewis* 2010;**8**:1112-68

Cattelani R, Zettin M, Zoccolotti P. Rehabilitation Treatments for Adults with Behavioral and Psychosocial Disorders Following Acquired Brain Injury: A Systematic Review. *Neuropsychology review* 2010;**20(1)**:52-85

Chalfoun C, Karelis AD, Stip E, Abdel-Baki A. Running for your life: A review of physical activity and cardiovascular disease risk reduction in individuals with schizophrenia. *Journal of Sports Sciences* 2016;**34**:1500-15

Challet-Bouju G, Bruneau M, Ignace G, Victorri-Vigneau C, Grall-Bronnec M. Cognitive Remediation Interventions for Gambling Disorder: A Systematic Review. *Frontiers in Psychology* 2017;**8**:1961

Chambers SK, Pinnock C, Lepore SJ, Hughes S, O'Connell DL. A systematic review of psychosocial interventions for men with prostate cancer and their partners. *Patient Education and Counseling* 2011;**85**:e75-e88

Chan E, Fogler JM, Hammerness PG. Treatment of Attention-Deficit/Hyperactivity Disorder in Adolescents: A Systematic Review. *JAMA* 2016;**315(18)**:1997-2008

Chatterton ML, Stockings E, Berk M, Barendregt JJ, Carter R, Mihalopoulos C. Psychosocial therapies for the adjunctive treatment of bipolar disorder in adults: network meta-analysis. *British Journal of Psychiatry* 2017;**210**:333-41

Chen I, Opiyo N, Tavender E, Mortazhejri S, Rader T, Petkovic J*, et al.* Non-clinical interventions for reducing unnecessary caesarean section. *Cochrane Database Syst Rev* 2018;**28(9)**:CD005528

Cheney G. *Psychology in schools : understanding and intervening within vulnerable populations*: University of Hull; 2011.

Chew HSJ, Cheng HY, Chair SY. The suitability of motivational interviewing versus cognitive behavioural interventions on improving self-care in patients with heart failure: A literature review and discussion paper. *Applied Nursing Research* 2019;**45**:17-22

Chi NC, Demiris G, Lewis FM, Walker AJ, Langer SL. Behavioral and Educational Interventions to Support Family Caregivers in End-of-Life Care: A Systematic Review. *The American journal of hospice & palliative care* 2016;**33**:894-908

Clare L, Teale JC, Toms G, Kudlicka A, Evans I, Abrahams S*, et al.* Cognitive rehabilitation, self-management, psychotherapeutic and caregiver support interventions in progressive neurodegenerative conditions: A scoping review. *NeuroRehabilitation* 2018;**43**:443-71

Clough BA, March S, Chan RJ, Casey LM, Phillips R, Ireland MJ. Psychosocial interventions for managing occupational stress and burnout among medical doctors: A systematic review. *Systematic reviews* 2017;**6(1)** 144

Clucas C, Sibley E, Harding R, Liu L, Catalan J, Sherr L. A systematic review of Interventions for anxiety in people with HIV. *Psychology, Health & Medicine* 2011;**16**:528-47

Coleman T, Chamberlain C, Davey M, Cooper SE, Leonardi-Bee J. Pharmacological interventions for promoting smoking cessation during pregnancy. *Cochrane Database Syst Rev* 2012;**12(9)**:CD010078

Coleman T, Chamberlain C, Davey MA, Cooper SE, Leonardi-Bee J. Pharmacological interventions for promoting smoking cessation during pregnancy. *Cochrane Database Syst Rev* 2015;**22(12)**

Collatz A, Johnston SC, Staines DR, Marshall-Gradisnik SM. A Systematic Review of Drug Therapies for Chronic Fatigue Syndrome/Myalgic Encephalomyelitis. *Clinical Therapeutics* 2016;**38**:1263-71.e9

Conway A, Schadewaldt V, Clark R, Ski CF, Thompson DR, Kynoch K*, et al.* The effectiveness of non-pharmacologic interventions in improving psychological outcomes for heart transplant recipients: a systematic review. *JBI Database of Systematic Reviews & Implementation Reports* 2013;**11**:112-32

Cooney P, Tunney C, O'Reilly G. A systematic review of the evidence regarding cognitive therapy skills that assist cognitive behavioural therapy in adults who have an intellectual disability. *Journal of Applied Research in Intellectual Disabilities* 2018;**31**:23-42

Couch R, Jetha M, Dryden DM, Hooton N, Liang Y, Durec T*, et al. Diabetes education for children with type 1 diabetes mellitus and their families.* Rockville (MD): Agency for Healthcare Research and Quality (US). Report No: 08-E011; 2008.

Coughtrey A, Millington A, Bennett S, Christie D, Hough R, Su MT*, et al.* The Effectiveness of Psychosocial Interventions for Psychological Outcomes in Pediatric Oncology: A Systematic Review. *Journal of Pain & Symptom Management* 2018;**55**:1004-17

Covin R, Ouimet AJ, Seeds PM, Dozois DJ. A meta-analysis of CBT for pathological worry among clients with GAD. *Journal of Anxiety Disorders* 2008;**22**:108-16

Cramer H, Lauche R, Paul A, Langhorst J, Kummel S, Dobos GJ. Hypnosis in breast cancer care: A systematic review of randomized controlled trials. *Integrative Cancer Therapies* 2015;**14**:5-15

Cuijpers P, Andersson G, Donker T, Van Straten A. Psychological treatment of depression: Results of a series of meta-analyses. *Nordic Journal of Psychiatry* 2011;**65**:354-64

Cuijpers P, Berking M, Andersson G, Quigley L, Kleiboer A, Dobson KS. A meta-analysis of cognitive-behavioural therapy for adult depression, alone and in comparison with other treatments. *Canadian Journal of Psychiatry* 2013;**58**:376-85

Cuijpers P, Cristea I, Weitz E, Gentili C, Berking M. The effects of cognitive and behavioural therapies for anxiety disorders on depression: A meta-analysis. *Psychological Medicine* 2016;**46**:3451-62

Cuijpers P, Donker T, Weissman MM, Ravitz P, Cristea IA. Interpersonal Psychotherapy for Mental Health Problems: A Comprehensive Meta-Analysis. *American Journal of Psychiatry* 2016;**173**:680-7

Cuijpers P, Weitz E, Lamers F, Penninx BW, Twisk J, DeRubeis RJ, *et al.* Melancholic and atypical depression as predictor and moderator of outcome in cognitive behavior therapy and pharmacotherapy for adult depression. *Depression and Anxiety* 2017;**34**:246-56

Cullen KL, Irvin E, Collie A, Clay F, Gensby U, Jennings PA, *et al.* Effectiveness of Workplace Interventions in Return-to-Work for Musculoskeletal, Pain-Related and Mental Health Conditions: An Update of the Evidence and Messages for Practitioners. *Journal of Occupational Rehabilitation* 2018;**28**:1-15

Cusack K, Jonas DE, Forneris CA, Wines C, Sonis J, Middleton JC, *et al.* Psychological treatments for adults with posttraumatic stress disorder: A systematic review and meta-analysis. *Clinical Psychology Review* 2016;**43**:128-41

Cusimano MD, Nastis S, Zuccaro L, Cusimano MD, Nastis S, Zuccaro L. Effectiveness of interventions to reduce aggression and injuries among ice hockey players: a systematic review. *CMAJ: Canadian Medical Association Journal* 2013;**185**:E57-69

Cwikel JG, Behar LC. Social work with adult cancer patients: a vote-count review of intervention research. *Social Work in Health Care* 1999;**29**:39-67

Daigle MS, Pouliot L, Chagnon F, Greenfield B, Mishara B. Suicide attempts: Prevention of repetition. *Canadian Journal of Psychiatry* 2011;**56**:621-9

Dainty AD. Irritable bowel syndrome: psychological comorbidities and cognitive behavioural therapy. A review of the literature. *Gastrointestinal Nursing* 2012;**10**:44-50

Damen L, Bruijn J, Koes BW, Berger MY, Passchier J, Verhagen AP. Prophylactic treatment of migraine in children. Part 1. A systematic review of non-pharmacological trials. *Cephalalgia* 2006;**26**:373-83

Damgaard P, Bartels EM, Ris I, Christensen R, Juul-Kristensen B. Evidence of Physiotherapy Interventions for Patients with Chronic Neck Pain: A Systematic Review of Randomised Controlled Trials. *ISRN Pain* 2013;**2013**:567175

David D, Cotet C, Matu S, Mogoase C, Stefan S. 50 years of rational-emotive and cognitive-behavioral therapy: A systematic review and meta-analysis. *Journal of Clinical Psychology* 2017;**74**:304-18

Davis AM, MacKay C. Osteoarthritis year in review: Outcome of rehabilitation. *Osteoarthritis and Cartilage* 2013;**21**:1414-24

Davis ML, Powers MB, Handelsman P, Medina JL, Zvolensky M, Smits JA. Behavioral therapies for treatment-seeking cannabis users: a meta-analysis of randomized controlled trials. *Evaluation & the Health Professions* 2015;**38**:94-114

D'Egidio V, Sestili C, Mancino M, Sciarra I, Cocchiara R, Backhaus I, *et al.* Counseling interventions delivered in women with breast cancer to improve health-related quality of life: A systematic review. *Quality of Life Research* 2017;**26(10)**:2573-92

Devine EC. Meta-analysis of the effect of psychoeducational interventions on pain in adults with cancer. *Oncology Nursing Forum* 2003;**30**:75-89

Dirmaier J, Steinmann M, Krattenmacher T, Watzke B, Barghaan D, Koch U, *et al.* Non-pharmacological treatment of depressive disorders: A review of evidence-based treatment options. *Reviews on Recent Clinical Trials* 2012;**7**:141-9

Dixon S, Dantas JA. Best practice for community-based management of postnatal depression in developing countries: A systematic review. *Health Care for Women International* 2017;**38**:118-43

Dolemeyer R, Tietjen A, Kersting A, Wagner B. Internet-based interventions for eating disorders in adults: A systematic review. *BMC Psychiatry* 2013;**13**

Dolle K, Schulte-Korne G. The treatment of depressive disorders in children and adolescents. . *Deutsches Arzteblatt International* 2013;**110(50)**:854-60

Dorstyn D, Mathias J, Denson L. Efficacy of cognitive behavior therapy for the management of psychological outcomes following spinal cord injury a meta-analysis. *Journal of Health Psychology* 2011;**16**:374-91

Dossa NI, Hatem M. Cognitive-behavioral therapy versus other PTSD psychotherapies as treatment for women victims of war-related violence: a systematic review. *Scientific World Journal* 2012: 181847

Dubicka B, Elvins R, Roberts C, Chick G, Wilkinson P, Goodyer IM. Combined treatment with cognitive-behavioural therapy in adolescent depression: meta-analysis. *British Journal of Psychiatry* 2010;**197**:433-40

Duncan LR, Pearson ES, Maddison R. Smoking prevention in children and adolescents: A systematic review of individualized interventions. *Patient Education & Counseling* 2018;**101**:375-88

DuPaul GJ, Eckert TL, Vilardo B. The effects of school-based interventions for attention deficit hyperactivity disorder: A meta-analysis 1996-2010. *School Psychology Review* 2012;**41**:387-412

Eilender P, Ketchen B, Maremmani I, Saenger M, Fareed A. Treatment Approaches for Patients With Opioid Use Disorder and Chronic Noncancer Pain: a Literature Review. *Addictive Disorders & Their Treatment* 2016;**15**:85-98

Eisenberg DM, Delbanco TL, Berkey CS, Kaptchuk TJ, Kupelnick B, Kuhl J*, et al.* Cognitive behavioral techniques for hypertension: Are they effective? *Annals of Internal Medicine* 1993;**118**:964-72

Elderton AJ. *Posttraumatic growth in survivors of interpersonal violence in additional*: University of Oxford (United Kingdom); 2013.

El-Serag HB, Olden K, Bjorkman D. Health-related quality of life among persons with irritable bowel syndrome: a systematic review. *Alimentary pharmacology & therapeutics* 2002;**16**:1171-85

Enck P, Junne F, Klosterhalfen S, Zipfel S, Martens U. Therapy options in irritable bowel syndrome. *European Journal of Gastroenterology & Hepatology* 2010;**22**:1402-11

Engels GI, Vermey M. Efficacy of nonmedical treatments of depression in elders: A quantitative analysis>. *Journal of Clinical Geropsychology* 1997;**3**:17-35

Everitt H, Baldwin DS, Stuart B, Lipinska G, Mayers A, Malizia AL*, et al.* Antidepressants for insomnia in adults. *Cochrane Database Syst Rev* 2018;**14(5)**:CD010753

Fann JR, Hart T, Schomer KG. Treatment for depression after traumatic brain injury: a systematic review. *Journal of NeuroTrauma* 2009;**26**:2383-402

Farooq S, Sherin A. Interventions for psychotic symptoms concomitant with epilepsy. *Cochrane Database Syst Rev* 2008;**8(4)**:CD006118

Farooq S, Sherin A. Interventions for psychotic symptoms concomitant with epilepsy. *Cochrane Database Syst Rev* 2015;**21(12)**:CD006118

Faulkner G, Cohn T, Remington G. Interventions to reduce weight gain in schizophrenia. *Cochrane Database Syst Rev* 2007;**24(1)**:CD005148

Fernandez E, Malvaso C, Day A, Guharajan D. 21st Century Cognitive Behavioural Therapy for Anger: A Systematic Review of Research Design, Methodology and Outcome. *Behavioural & Cognitive Psychotherapy* 2018;**46(4)**:385-404

Feske U, Chambless DL. Cognitive behavioral versus exposure only treatment for social phobia: A meta-analysis. *Behavior Therapy* 1995;**26**:695-720

Field T. Postnatal anxiety prevalence, predictors and effects on development: A narrative review. *Infant Behavior & Development* 2018;**51**:24-32

Fontenelle LF, Coutinho ES, Lins-Martins NM, Fitzgerald PB, Fujiwara H, Yucel M. Electroconvulsive therapy for obsessive-compulsive disorder: a systematic review. *Journal of Clinical Psychiatry* 2015;**76**:949-57

Forbes D, Creamer M, Phelps A, Bryant R, McFarlane A, Devilly GJ*, et al.* Australian guidelines for the treatment of adults with acute stress disorder and post-traumatic stress disorder. *Australian & New Zealand Journal of Psychiatry* 2007;**41**:637-48

Forman-Hoffman VL, Zolotor AJ, McKeeman JL, Blanco R, Knauer SR, Lloyd SW*, et al.* Comparative Effectiveness of Interventions for Children Exposed to Nonrelational Traumatic Events. *Pediatrics* 2013;**131**:526-39

Foster ER, Bedekar M, Tickle-Degnen L. Systematic Review of the Effectiveness of Occupational Therapy-Related Interventions for People With Parkinson's Disease. *American Journal of Occupational Therapy* 2014;**68**:39-49

Frazer CJ, Christensen H, Griffiths KM. Effectiveness of treatments for depression in older people. *Medical Journal of Australia* 2005;**182**:627-32

Frechette-Simard C, Plante I, Bluteau J. Strategies included in cognitive behavioral therapy programs to treat internalized disorders: A systematic review. *Cognitive behaviour therapy* 2018;**47**:263-85

Freire RC, Zugliani MM, Garcia RF, Nardi AE. Treatment-resistant panic disorder: A systematic review. *Expert Opinion on Pharmacotherapy* 2016;**17**:159-68

Freudenstein U, Jagger C, Arthur A, Donner-Banzhoff N. Treatments for late life depression in primary care - A systematic review. *Family Practice* 2001;**18**:321-7

Fricton JR, Ouyang W, Nixdorf DR, Schiffman EL, Velly AM, Look JO. Critical appraisal of methods used in randomized controlled trials of treatments for temporomandibular disorders. *Journal of Orofacial Pain* 2010;**24**:139-51

Friedman MA, Detweiler-Bedell JB, Leventhal HE, Horne R, Keitner GI, Miller IW. Combined psychotherapy and pharmacotherapy for the treatment of major depressive disorder. *Clinical Psychology: Science and Practice* 2004;**11**:47-68

Fu F, Zhao H, Tong F, Chi I. A Systematic Review of Psychosocial Interventions to Cancer Caregivers. *Frontiers in Psychology* 2017;**8**:834

Fuller TE, Haider HF, Kikidis D, Lapira A, Mazurek B, Norena A*, et al.* Different Teams, Same Conclusions? A Systematic Review of Existing Clinical Guidelines for the Assessment and Treatment of Tinnitus in Adults. *Frontiers in Psychology* 2017;**8**:206

Furlong M, McGilloway S, Bywater T, Hutchings J, Smith SM, Donnelly M. Behavioural and cognitive-behavioural group-based parenting programmes for early-onset conduct problems in children aged 3 to 12 years. *Cochrane Database Syst Rev* 2012;**15(2)**:CD008225

Furlong M, McGilloway S, Bywater T, Hutchings J, Smith SM, Donnelly M. Cochrane review: behavioural and cognitive-behavioural group-based parenting programmes for early-onset conduct problems in children aged 3 to 12 years. *Evidence-Based Child Health* 2013;**8(2)** 318-692

Garvey WT, Mechanick JI, Brett EM, Garber AJ, Hurley DL, Jastreboff AM*, et al.* American Association of Clinical Endocrinologists and American College of Endocrinology Comprehensive Clinical Practice Guidelines for Medical Care of Patients with Obesity. *Endocrine Practice* 2016;**22 Suppl 3**:1-203

Gerson S, Belin TR, Kaufman A, Mintz J, Jarvik L. Pharmacological and psychological treatments for depressed older patients: A meta-analysis and overview of recent findings. *Harvard Review of Psychiatry* 1999;**7**:1-28

Ghadiri-Sani M, Silver N. Headache (chronic tension-type). *Clinical Evidence* 2016;**05**:05

Gibson E, Sabo MT. Can pain catastrophizing be changed in surgical patients? A scoping review. *Canadian journal of surgery* 2018;**61**:311-8

Gilchrist G, Swan D, Widyaratna K, Marquez-Arrico JE, Hughes E, Mdege ND*, et al.* A Systematic Review and Meta-analysis of Psychosocial Interventions to Reduce Drug and Sexual Blood Borne Virus Risk Behaviours Among People Who Inject Drugs. *AIDS and behavior* 2017;**21**:1791-811

Gilpin HR, Keyes A, Stahl DR, Greig R, McCracken LM. Predictors of Treatment Outcome in Contextual Cognitive and Behavioral Therapies for Chronic Pain: A Systematic Review. *Journal of Pain* 2017;**18**:1153-64

Goldbeck L, Fidika A, Herle M, Quittner AL. Psychological interventions for individuals with cystic fibrosis and their families. *Cochrane Database Syst Rev* 2014;**18(6)**:CD003148

Gonzalez S, Artal J, Gomez E, Caballero P, Mayoral J, Moreno T*, et al.* Early intervention in Bipolar Disorder: The jano program at hospital universitario marques de valdecilla. *Actas Espanolas de Psiquiatria* 2012;**40**:51-6

Gordon J, King NJ, Gullone E, Muris P, Ollendick TH. Treatment of children's nighttime fears: the need for a modern randomised controlled trial. *Clinical Psychology Review* 2007;**27**:98-113

Gould RA, Safren SA, Washington DON, Otto MW. A Meta-Analytic Review of Cognitive-Behavioral Treatments. In: Meimberg RG, Turk CL, Mennin DS, editors. *Generalized anxiety disorder: advances in research and practice*New York, NY (US): Guilford Publications, Inc; 2004:248-64.

Gould RL, Coulson MC, Brown RG, Goldstein LH, Al-Chalabi A, Howard RJ. Psychotherapy and pharmacotherapy interventions to reduce distress or improve well-being in people with amyotrophic lateral sclerosis: A systematic review. *Amyotrophic Lateral Sclerosis and Frontotemporal Degeneration* 2015;**16**:293-302

Gouzoulis-Mayfrank E, Hartel-Petri R, Hamdorf W, Havemann-Reinecke U, Muhlig S, Wodarz N. Methamphetamine-related disorders. *Deutsches Arzteblatt International* 2017;**114**:455-61

Grewal R, Spielmann PM, Jones SEM, Hussain SSM. Clinical efficacy of tinnitus retraining therapy and cognitive behavioural therapy in the treatment of subjective tinnitus: a systematic review. *Journal of Laryngology & Otology* 2014;**128**:1028-33

Gu W, Storch EA, Zhao Q, Xu T, Wang Z. Effects of D-cycloserine augmentation on cognitive behavioral therapy in patients with obsessive-compulsive disorder: A systematic review and meta-analysis. *Journal of Obsessive-Compulsive and Related Disorders* 2017;**13**:24-9

Hackett KL, Gotts ZM, Ellis J, Deary V, Rapley T, Ng WF*, et al.* An investigation into the prevalence of sleep disturbances in primary Sjogren's syndrome: A systematic review of the literature. *Rheumatology (United Kingdom)* 2017;**56**:570-80

Haines T, Gross A, Burnie SJ, Goldsmith CH, Perry L. Patient education for neck pain with or without radiculopathy. *Cochrane Database Syst Rev* 2009;**21(1)**:CD005106

Haines T, Gross A, Goldsmith CH, Perry L. Patient education for neck pain with or without radiculopathy. *Cochrane Database Syst Rev* 2008;**8(4)**:CD005106

Haines T, Gross AR, Burnie S, Goldsmith CH, Perry L, Graham N. A Cochrane review of patient education for neck pain. *Spine Journal* 2009;**9**:859-71

Hartnett D, Carr A, Hamilton E, O'Reilly G. The Effectiveness of Functional Family Therapy for Adolescent Behavioral and Substance Misuse Problems: A Meta-Analysis. *Family Process* 2017;**56**:607-19

Häuser W, Thieme K, Turk DC. Guidelines on the management of fibromyalgia syndrome - a systematic review. *European Journal of Pain* 2010;**14**:5-10

Hay P. Australian and New Zealand clinical practice guidelines for the treatment of anorexia nervosa. *Australian and New Zealand Journal of Psychiatry* 2004;**38**:659-70

Heaton LJ. Behavioral Interventions May Reduce Dental Anxiety and Increase Acceptance of Dental Treatment in Dentally Fearful Adults. *Journal of Evidence-Based Dental Practice* 2013;**13**:160-2

Hegedus A, Kozel B. Does adherence therapy improve medication adherence among patients with schizophrenia? A systematic review. *International Journal of Mental Health Nursing* 2014;**23**:490-7

Hersch J, Juraskova I, Price M, Mullan B. Psychosocial interventions and quality of life in gynaecological cancer patients: a systematic review. *Psycho-Oncology* 2009;**18**:795-810

Hesse M. Achieving abstinence by treating depression in the presence of substance-use disorders. *Addictive Behaviors* 2004;**29**:1137-41

Hetrick Sarah E, Purcell R, Garner B, Parslow R. Combined pharmacotherapy and psychological therapies for post traumatic stress disorder (PTSD). *Cochrane Database Syst Rev* 2010;**(7)**:CD007316

Hilfiker R, Meichtry A, Eicher M, Nilsson BL, Knols RH, Verra ML*, et al.* Exercise and other non-pharmaceutical interventions for cancer-related fatigue in patients during or after cancer treatment: a systematic review incorporating an indirect-comparisons meta-analysis. *British Journal of Sports Medicine* 2017;**13**:13

Hill A, Brettle A. The effectiveness of counselling with older people: results of a systematic review. *Counselling & Psychotherapy Research* 2005;**5**:265-72

Himelhoch S, Medoff DR, Oyeniyi G. Efficacy of group psychotherapy to reduce depressive symptoms among HIV-infected individuals: a systematic review and meta-analysis. *AIDS Patient Care & STDs* 2007;**21**:732-9

Hirschtritt ME, Bloch MH, Mathews CA. Obsessive-Compulsive Disorder: Advances in Diagnosis and Treatment. *JAMA: Journal of the American Medical Association* 2017;**317**:1358-67

Hjorthoj C, Fohlmann A, Nordentoft M. Reprint of "Treatment of cannabis use disorders in people with schizophrenia spectrum disorders - A systematic review". *Addictive Behaviors* 2009;**34**:846-51

Hjorthoj CR, Baker A, Fohlmann A, Nordentoft M. Intervention efficacy in trials targeting cannabis use disorders in patients with comorbid psychosis systematic review and meta-analysis. *Current Pharmaceutical Design* 2014;**20**:2205-11

Ho BP, Carter M, Stephenson J. Anger management using a cognitive-behavioural approach for children with special education needs: a literature review and meta-analysis. *International Journal of Disability Development and Education* 2010;**57**:245-65

Ho ECM, Siu AMH. Occupational Therapy Practice in Sleep Management: A Review of Conceptual Models and Research Evidence. *Occupational Therapy International* 2018:8637498

Hoch E, Preuss UW, Ferri M, Simon R. Digital Interventions for Problematic Cannabis Users in Non-Clinical Settings: Findings from a Systematic Review and Meta-Analysis. *European Addiction Research* 2016;**22**:233-42

Hodgkinson B, Evans D, O'Donnell A, Nicholson J, Walsh K. The effectiveness of individual therapy and group therapy in the treatment of schizophrenia. *JBI Library of Systematic Reviews* 2000;**2(2)**:1-44

Hoffman BM, Papas RK, Chatkoff DK, Kerns RD. Meta-analysis of psychological interventions for chronic low back pain. *Health Psychology* 2007;**26**:1-9

Hofmann SG, Curtiss J, Carpenter JK, Kind S. Effect of treatments for depression on quality of life: a meta-analysis. *Cognitive Behaviour Therapy* 2017;**46**:265-86

Hofmann SG, Sawyer AT, Korte KJ, Smits JA. Is it beneficial to add pharmacotherapy to cognitive-behavioral therapy when treating anxiety disorders? A meta-analytic review. *International Journal of Cognitive Therapy* 2009;**2**:160-75

Hofmann SG, Wu JQ, Boettcher H. Effect of cognitive-behavioral therapy for anxiety disorders on quality of life: A meta-analysis. *Journal of Consulting and Clinical Psychology* 2014;**82**:375-91

Hoogsteder LM, Stams GJJM, Figge MA, Changoe K, van Horn JE, Hendriks J*, et al.* A meta-analysis of the effectiveness of individually oriented Cognitive Behavioral Treatment (CBT) for severe aggressive behavior in adolescents. *Journal of Forensic Psychiatry and Psychology* 2015;**26**:22-37

Howard MC. *Science fiction meets scientific inquiry: A task-technology fit/computer-based training framework and a meta-analysis of virtual reality applications for intervention, training, and therapy purposes*: Pennsylvania State University; 2018.

Hubbard JB. *Psychotherapy outcome for eating disorders: A meta-analysis*: Brigham Young University; 2014.

Huguet A, McGrath PJ, Stinson J, Tougas ME, Doucette S. Efficacy of psychological treatment for headaches: an overview of systematic reviews and analysis of potential modifiers of treatment efficacy. *Clinical Journal of Pain* 2014;**30**:353-69

Hui PS, Yi SM, Kuswanto CN, Kang S. Evidence-based options for treatment-resistant adult bipolar disorder patients. *Annals of the Academy of Medicine Singapore* 2012;**(1)**:S230

Hunot V, Moore TH, Caldwell DM, Furukawa TA, Davies P, Jones H*, et al.* 'Third wave' cognitive and behavioural therapies versus other psychological therapies for depression. *Cochrane Database Syst Rev* 2013;**18(10)**:CD008704

Hunt GE, Siegfried N, Morley K, Sitharthan T, Cleary M. Psychosocial interventions for people with both severe mental illness and substance misuse. *Cochrane Database Syst Rev* 2013;**3(10)**:CD001088

In-Albon T, Schneider S. Psychotherapy of childhood anxiety disorders: A meta-analysis. *Psychotherapy and Psychosomatics* 2007;**76**:15-24

Irwin MR, Cole JC, Nicassio PM. Comparative meta-analysis of behavioral interventions for insomnia and their efficacy in middle-aged adults and in older adults 55+ years of age. *Health Psychology* 2006;**25**:3-14

IsHak WW, Bolton MA, Bensoussan JC, Dous GV, Nguyen TT, Powell-Hicks AL*, et al.* Quality of life in body dysmorphic disorder. *CNS Spectrums* 2012;**17**:167-75

IsHak WW, Wen RY, Naghdechi L, Vanle B, Dang J, Knosp M*, et al.* Pain and Depression: A Systematic Review. *Harvard Review of Psychiatry* 2018;**26**:352-63

Itlescas SR, Sanchez-Meca J, Genoves VG. Treatment of offenders and recidivism: Assessment of the effectiveness of programmes applied in Europe. *Psychology in Spain* 2001;**5**:47-62

Jackson CF, Makin SM, Marson AG, Kerr M. Non-pharmacological interventions for people with epilepsy and intellectual disabilities. *Cochrane Database Syst Rev* 2015;**(9)**:CD005502

Jarrell JF, Vilos GA, Allaire C, Burgess S, Fortin C, Gerwin R*, et al.* Consensus Guidelines for the Management of Chronic Pelvic Pain. *Journal of Obstetrics and Gynaecology Canada* 2005;**27**:869-87

Jarvinen M, Stolt M, Honkala E, Leino-Kilpi H, Pollanen M. Behavioural interventions that have the potential to improve self-care in adults with periodontitis: a systematic review. *Acta Odontologica Scandinavica* 2018;**76**:612-20

Jaworska-Burzynska L, Kanaffa-Kilijanska U, Przysiezna E, Szczepanska-Gieracha J. The role of therapy in reducing the risk of job burnout - A systematic review of literature. *Archives of Psychiatry and Psychotherapy* 2016;**18**:43-52

Jenkinson E, Williamson H, Byron-Daniel J, Moss TP. Systematic Review: Psychosocial Interventions for Children and Young People With Visible Differences Resulting From Appearance Altering Conditions, Injury, or Treatment Effects. *Journal of Pediatric Psychology* 2015;**40**:1017-33

Jewell LM, Wormith J. Variables associated with attrition from domestic violence treatment programs targeting male batterers: A meta-analysis. *Criminal Justice and Behavior* 2010;**37**:1086-113

Jones B, de CWAC. CBT to reduce healthcare use for medically unexplained symptoms: systematic review and meta-analysis. *British Journal of General Practice* 2019;**28**:28

Jones C, Hacker D, Cormac I, Meaden A, Irving CB. Cognitive behavior therapy versus other psychosocial treatments for schizophrenia. *Schizophrenia Bulletin* 2012;**38**:908-10

Jong E, Oudhoff LA, Epskamp C, Wagener MN, Van Duijn M, Fischer S*, et al.* Predictors and treatment strategies of HIV-related fatigue in the combined antiretroviral therapy era. *Aids* 2010;**24**:1387-405

Jorm AF, Christensen H, Griffiths KM, Rodgers B. Effectiveness of complementary and self-help treatments for depression. *Medical Journal of Australia* 2002;**176**:S84-S96

Kaptein AA, Scharloo M, Fischer MJ, Snoei L, Cameron LD, Sont JK*, et al.* Illness perceptions and COPD: An emerging field for COPD patient management. *Journal of Asthma* 2008;**45**:625-9

Kelly KP, Kirschenbaum DS. Immersion treatment of childhood and adolescent obesity: The first review of a promising intervention. *Obesity Reviews* 2011;**12**:37-49

Kennedy CA, Amick BC, III, Dennerlein JT, Brewer S, Catli S, Williams R*, et al.* Systematic review of the role of occupational health and safety interventions in the prevention of upper extremity musculoskeletal symptoms, signs, disorders, injuries, claims and lost time. *Journal of Occupational Rehabilitation* 2010;**20**:127-62

Kent P, Kjaer P. The efficacy of targeted interventions for modifiable psychosocial risk factors of persistent nonspecific low back pain – A systematic review. *Manual Therapy* 2012;**17**:385-401

Kenworthy T, Adams CE, Bilby C, Brooks-Gordon B, Fenton M. Psychological interventions for those who have sexually offended or are at risk of offending. *Cochrane Database Syst Reviews* 2004;**(3)**:CD004858

Kim HS, Kim EJ. Effects of Relaxation Therapy on Anxiety Disorders: A Systematic Review and Meta-analysis. *Archives of Psychiatric Nursing* 2018;**32**:278-84

King DL, Delfabbro PH, Wu AMS, Doh YY, Kuss DJ, Pallesen S*, et al.* Treatment of Internet gaming disorder: An international systematic review and CONSORT evaluation. *Clinical Psychology Review* 2017;**54**:123-33

Klinsophon T, Thaveeratitham P, Sitthipornvorakul E, Janwantanakul P. Effect of exercise type on smoking cessation: a meta-analysis of randomized controlled trials. *BMC Research Notes* 2017;**10**:442

Knight L, Mukumbang FC, Schatz E. Behavioral and cognitive interventions to improve treatment adherence and access to HIV care among older adults in sub-Saharan Africa: an updated systematic review. *Systematic Reviews* 2018;**7**:114

Koehler JA, Losel F, Akoensi TD, Humphreys DK. A systematic review and meta-analysis on the effects of young offender treatment programs in Europe. *Journal of Experimental Criminology* 2013;**9**:19-43

Krishnan A, Silver N. Headache (chronic tension-type). *BMJ clinical evidence* 2009:pii: 1205
Kroll C, Doebler P, Nuesch S. Meta-analytic evidence of the effectiveness of stress management at work. *European Journal of Work and Organizational Psychology* 2017;**26**:677-93

Kuder S, Accardo A. What works for college students with autism spectrum disorder. *Journal of Autism and Developmental Disorders* 2018;**48**:722-31

Kuerbis A, Sacco P. A review of existing treatments for substance abuse among the elderly and recommendations for future directions. *Substance Abuse: Research and Treatment* 2013;**7**:13-37

Kuin N, Masthoff E, Kramer M, Scherder E. The role of risky decision-making in aggression: A systematic review. *Aggression and Violent Behavior* 2015;**25(A)**:159-72

Kwan I, Onwude JL. Premenstrual syndrome. *BMJ clinical evidence* 2007:pii: 0806

Kwekkeboom KL, Bratzke LC. A Systematic Review of Relaxation, Meditation, and Guided Imagery Strategies for Symptom Management in Heart Failure. *Journal of Cardiovascular Nursing* 2016;**31**:457-68

Kyle SD, Aquino MRJ, Miller CB, Henry AL, Crawford MR, Espie CA*, et al.* Towards standardisation and improved understanding of sleep restriction therapy for insomnia disorder: A systematic examination of CBT-I trial content. *Sleep Medicine Reviews* 2015;**23**:83-8

Lancee J, Spoormaker VI, Krakow B, van den Bout J. A systematic review of cognitive-behavioral treatment for nightmares: Toward a well-established treatment. *Journal of Clinical Sleep Medicine* 2008;**4**:475-80

Lapp LK, Agbokou C, Peretti CS, Ferreri F. Management of post traumatic stress disorder after childbirth: A review. *Journal of Psychosomatic Obstetrics and Gynecology* 2010;**31**:113-22

Lee J, Kim Y, Kim YL. Non-pharmacological therapies for sleep disturbances in people with Parkinson's disease: A systematic review. *Journal of Advanced Nursing* 2018;**27**:27

Leichsenring F, Leibing E. The effectiveness of psychodynamic therapy and cognitive behavior therapy in the treatment of personality disorders: a meta-analysis. *American Journal of Psychiatry* 2003;**160**:1223-32

Lemos IL, De Abreu CN, Sougey EB. Internet and video game addictions: A cognitive behavioral approach. *Revista de Psiquiatria Clinica* 2014;**41**:82-8

Lewandowski LM, Gebing TA, Anthony JL, O'Brien WH. Meta-analysis of cognitive-behavioral treatment studies for bulimia. *Clinical Psychology Review* 1997;**17**:703-18

Lilliengren P, Johansson R, Lindqvist K, Mechler J, Andersson G. Efficacy of experiential dynamic therapy for psychiatric conditions: A meta-analysis of randomized controlled trials. *Psychotherapy* 2016;**53**:90-104

Lim Hoon SH, Chan WCS, He HG. Effect of psychosocial interventions on outcomes of patients with colorectal cancer: A review of the literature. *European Journal of Oncology Nursing* 2013;**17**:883-91

Lin M-Y, Liu MF, Hsu L-F, Tsai P-S. Effects of self-management on chronic kidney disease: A meta-analysis. *International Journal of Nursing Studies* 2017;**74**:128-37

Linardon J, Fitzsimmons-Craft EE, Brennan L, Barillaro M, Wilfley DE. Dropout from interpersonal psychotherapy for mental health disorders: A systematic review and meta-analysis. *Psychotherapy Research* 2018;**29(7)**:870-81

Linde K, Treml J, Steinig J, Nagl M, Kersting A. Grief interventions for people bereaved by suicide: A systematic review. *Plos One* 2017;**12(6)**

Lindhiem O, Higa J, Trentacosta C, Herschell A, Kolko D. Skill Acquisition and Utilization During Evidence-Based Psychosocial Treatments for Childhood Disruptive Behavior Problems: A Review and Meta-analysis. *Clinical Child & Family Psychology Review* 2014;**17**:41-66

Liu C, Liao M, Smith DC. An Empirical Review of Internet Addiction Outcome Studies in China. *Research on Social Work Practice* 2012;**22**:282-92

Lockwood C, Page T, Conroy-Hiller T. Comparing the effectiveness of cognitive behaviour therapy using individual or group therapy in the treatment of depression. *JBI Library of Systematic Reviews* 2004;**2**:1-33

Lorenzo-Luaces L, Johns E, Keefe JR. The Generalizability of Randomized Controlled Trials of Self-Guided Internet-Based Cognitive Behavioral Therapy for Depressive Symptoms: Systematic Review and Meta-Regression Analysis. *Journal of Medical Internet Research* 2018;**20**:e10113

Losel F, Schmucker M. The effectiveness of treatment for sexual offenders: A comprehensive meta-analysis. *Journal of Experimental Criminology* 2005;**1**:117-46

Lyman D, Kurtz MM, Farkas M, George P, Dougherty RH, Daniels AS*, et al.* Skill building: Assessing the evidence. *Psychiatric Services* 2014;**65**:727-38

Lynch D, Laws KR, McKenna PJ. Cognitive behavioural therapy for major psychiatric disorder: does it really work? A meta-analytical review of well-controlled trials. *Psychological Medicine* 2010;**40**:9-24

Mackin RS, Arean PA. Evidence-based psychotherapeutic interventions for geriatric depression. *Psychiatric Clinics of North America* 2005;**28**:805-20

Makdissi M, Schneider KJ, Feddermann-Demont N, Guskiewicz KM, Hinds S, Leddy JJ*, et al.* Approach to investigation and treatment of persistent symptoms following sport-related concussion: a systematic review. *British Journal of Sports Medicine* 2017;**51**:958-68

Manassis K, Lee TC, Bennett K, Zhao XY, Mendlowitz S, Duda S*, et al.* Types of parental involvement in CBT with anxious youth: a preliminary meta-analysis. *Journal of Consulting & Clinical Psychology* 2014;**82**:1163-72

Manfredini D, Ahlberg J, Winocur E, Lobbezoo F. Management of sleep bruxism in adults: a qualitative systematic literature review. *Journal of Oral Rehabilitation* 2015;**42**:862-74

Marc I, Toureche N, Ernst E, Hodnett ED, Blanchet C, Dodin S*, et al.* Mind-body interventions during pregnancy for preventing or treating women's anxiety. *Cochrane Database Syst Rev* 2011;**6(7)**:CD007559

Mariano TY, Urman RD, Hutchison CA, Jamison RN, Edwards RR. Cognitive Behavioral Therapy (CBT) for Subacute Low Back Pain: a Systematic Review. *Current Pain and Headache Reports* 2018;**22(3)**

Marker I, Norton PJ. The efficacy of incorporating motivational interviewing to cognitive behavior therapy for anxiety disorders: A review and meta-analysis. *Clinical Psychology Review* 2018;**62**:1-10

Marques DR, Gomes AA, Clemente V, dos Santos JM, Caetano G, Castelo-Branco M. Neurobiological correlates of psychological treatments for insomnia: A review. *European Psychologist* 2016;**21**:195-205

Marshall M, Rathbone J. Early intervention for psychosis. *Cochrane Database Syst Rev* 2006;**18(4)**:CD004718

Marshall M, Rathbone J. Early intervention for psychosis. *Cochrane Database Syst Rev* 2011;**15(6)**:CD004718

Martlew J, Baker GA, Goodfellow L, Bodde N, Aldenkamp A. Behavioural treatments for non-epileptic attack disorder. *Cochrane Database Syst Rev* 2009;**(4)**:CD006370

Mas-Exposito L, Amador-Campos JA, Gomez-Benito J, Lalucat-Jo L. Review of psychotherapeutic interventions for people with schizophrenia. *Anuario de Psicologia* 2013;**43**:101-16

Masterton KJ, Tariman JD. Effective Transitional Therapy for Adolescent and Young Adult Patients With Cancer: An Integrative Literature Review. *Clinical Journal of Oncology Nursing* 2016;**20**:391-7

Mataix-Cols D, De La Cruz LF, Monzani B, Rosenfield D, Andersson E, Perez-Vigil A, *et al.* D-cycloserine augmentation of exposure-based cognitive behavior therapy for anxiety, obsessive-compulsive, and posttraumatic stress disorders a systematic review and meta-analysis of individual participant data. *JAMA Psychiatry* 2017;**74**:501-10

Matthys F, Stes S, van den Brink W, Joostens P, Mobius D, Tremmery S, *et al.* Guideline for screening, diagnosis and treatment of ADHD in adults with substance use disorders. *International Journal of Mental Health and Addiction* 2014;**12**:629-47

McCart MR, Priester PE, Davies WH, Azen R. Differential effectiveness of behavioral parent-training and cognitive-behavioral therapy for antisocial youth: a meta-analysis. *Journal of Abnormal Child Psychology* 2006;**34**:527-43

McCart MR, Sheidow AJ. Evidence-based psychosocial treatments for adolescents with disruptive behavior. *Journal of Clinical Child and Adolescent Psychology* 2016;**45**:529-63

McClintock SM, Brandon AR, Husain MM, Jarrett RB. A systematic review of the combined use of electroconvulsive therapy and psychotherapy for depression. *Journal of ECT* 2011;**27**:236-43

McDonald P, Colwell B, Backinger CL, Husten C, Maule CO. Better practices for youth tobacco cessation: evidence of review panel. *American Journal of Health Behavior* 2003;**27**:S144-58

McGuire JF, Orr SP, Essoe JK, McCracken JT, Storch EA, Piacentini J. Extinction learning in childhood anxiety disorders, obsessive compulsive disorder and post-traumatic stress disorder: implications for treatment. *Expert Review of Neurotherapeutics* 2016;**16**:1155-74

McGuire JF, Piacentini J, Lewin AB, Brennan EA, Murphy TK, Storch EA. A Meta-Analysis of Cognitive Behaviour Therapy and Medication for Child Obsessive-Compulsive Disorder: Moderators of Treatment Efficacy, Response and Remission. *Depression and Anxiety* 2015;**32**:580-93

McKenzie PS. *Chronic low back pain and insomnia: understanding the experience and attributions made by out-patients about sleeplessness, pain and their interaction*: University of Edinburgh (United Kingdom); 2012.

McLaughlin B, Ryder D, Taylor MF. Effectiveness of interventions for grandparent caregivers: A systematic review. *Marriage & Family Review* 2017;**53**:509-31

McMurran M, Theodosi E. Is treatment non-completion associated with increased reconviction over no treatment? *Psychology, Crime & Law* 2007;**13**:333-43

Mehta S, Orenczuk S, Hansen KT, Aubut JAL, Hitzig SL, Legassic M, *et al.* An Evidence-Based Review of the Effectiveness of Cognitive Behavioral Therapy for Psychosocial Issues Post-Spinal Cord Injury. *Rehabilitation Psychology* 2011;**56**:15-25

Melnik T, Hawton K, McGuire H. Interventions for vaginismus. *Cochrane Database Syst Rev* 2012;**(12)**:CD001760

Menzies RE, Zuccala M, Sharpe L, Dar-Nimrod I. The effects of psychosocial interventions on death anxiety: A meta-analysis and systematic review of randomised controlled trials. *Journal of Anxiety Disorders* 2018;**59**:64-73

Meyer C, Denis CM, Berquin AD. Secondary prevention of chronic musculoskeletal pain: A systematic review of clinical trials. *Annals of Physical and Rehabilitation Medicine* 2018;**61(5)**:323-38

Miller BJ. A review of second-generation antipsychotic discontinuation in first-episode psychosis. *Journal of Psychiatric Practice* 2008;**14**:289-300

Milling LS, Gover MC, Moriarty CL. The effectiveness of hypnosis as an intervention for obesity: A meta-analytic review. *Psychology of Consciousness: Theory, Research, and Practice* 2018;**5**:29-45

Mirza SK, Deyo, R A. Systematic review of randomized trials comparing lumbar fusion surgery to nonoperative care for treatment of chronic back pain. *Spine* 2007;**32**:816-23

Mogul A, Irby MB, Skelton JA. A systematic review of pediatric obesity and family communication through the lens of addiction literature. *Childhood obesity* 2014;**10**:197-206

Molenaar NM, Kamperman AM, Boyce P, Bergink V. Guidelines on treatment of perinatal depression with antidepressants: An international review. *Australian & New Zealand Journal of Psychiatry* 2018;**52**:320-7

Momani TG, Berry DL. Integrative Therapeutic Approaches for the Management and Control of Nausea in Children Undergoing Cancer Treatment: A Systematic Review of Literature. *Journal of pediatric oncology nursing* 2017;**34**:173-84

Montgomery P, Lilly J. Insomnia in the elderly. *BMJ clinical evidence* 2007:pii: 2302

Monticone M, Ambrosini E, Cedraschi C, Rocca B, Fiorentini R, Restelli M*, et al.* Cognitive-behavioral Treatment for Subacute and Chronic Neck Pain: A Cochrane Review. *Spine* 2015;**40(19)**:1495-504

Moore KE, Hacker RL, Oberleitner L, McKee SA. Reentry interventions that address substance use: A systematic review. *Psychological services* 2018;**11**:11

Moore M, Carr A. Depression and grief. In: Carr A, editor. *What Works with Children and Adolescents? A Critical Review of Psychological Interventions with Children, Aolescents and their Families.*Florence, KY: Taylor & Frances/Routledge; London; 2000:203-32.

Moore THM, King AJL, Evans M, Sharp D, Persad R, Huntley AL. Supportive care for men with prostate cancer: Why are the trials not working? A systematic review and recommendations for future trials. *Cancer Medicine* 2015;**4**:1240-51

Moreno Gil PJ, Mendez Carrillo FX, Sanchez Meca J. Effectiveness of cognitive-behavioural treatment in social phobia: a meta-analytic review. *Psychology in Spain* 2001;**5**:17-25

Morley S, Eccleston C, Williams A. Systematic review and meta-analysis of randomized controlled trials of cognitive behaviour therapy and behaviour therapy for chronic pain in adults, excluding headache. *Pain* 1999;**80**:1-13

Morrell CJ, Sutcliffe P, Booth A, Stevens J, Scope A, Stevenson M*, et al.* A systematic review, evidence synthesis and meta-analysis of quantitative and qualitative studies evaluating the clinical effectiveness, the cost-effectiveness, safety and acceptability of interventions to prevent postnatal depression. *Health Technology Assessment* 2016;**20**:1-414

Morris L, Stander J, Ebrahim W, Eksteen S, Meaden OA, Ras A*, et al.* Effect of exercise versus cognitive behavioural therapy or no intervention on anxiety, depression, fitness and quality of life in adults with previous methamphetamine dependency: a systematic review. *Addiction science & clinical practice* 2018;**13**:4

Mpofu E, Athanasou JA, Rafe C, Belshaw SH. Cognitive-behavioral therapy efficacy for reducing recidivism rates of moderate- and high-risk sexual offenders: A scoping systematic literature review. *International journal of offender therapy and comparative criminology* 2018;**62**:170-86

Munn Z, Jordan Z. The effectiveness of interventions to reduce anxiety, claustrophobia, sedation and non-completion rates of patients undergoing high technology medical imaging. *JBI Library of Systematic Reviewis* 2012;**10**:1122-85

Munn Z, Jordan Z. Interventions to reduce anxiety, distress and the need for sedation in adult patients undergoing magnetic resonance imaging: a systematic review. *International Journal of Evidence-Based Healthcare* 2013;**11**:265-74

Munn Z, Jordan Z. Interventions to Reduce Anxiety, Distress, and the Need for Sedation in Pediatric Patients Undergoing Magnetic Resonance Imaging: A Systematic Review. *Journal of Radiology Nursing* 2013;**32**:87-96

Murawski B, Wade L, Plotnikoff RC, Lubans DR, Duncan MJ. A systematic review and meta-analysis of cognitive and behavioral interventions to improve sleep health in adults without sleep disorders. *Sleep Medicine Reviews* 2018;**40**:160-9

Murray M, Murray L, Donnelly M. Systematic review of interventions to improve the psychological well-being of general practitioners. *BMC Family Practice* 2016;**17**:36

Nagayama Hall GC. Sexual offender recidivism revisited: A meta-analysis of recent treatment studies. *Journal of Consulting and Clinical Psychology* 1995;**63**:802-9

Nagy D, Szamoskozi S. Efficacy of cognitive behavioral interventions on complicated grief in adults: A quantitative meta-analysis. *Erdelyi Pszichologiai Szemle* 2013;**14**:39-54

Nelson MK. *Meta-analysis: Hypnotherapy/cognitive-behavioral therapy and its efficacy on depression compared to pharmacotherapy.* Alliant International University; 2002.

Nicoll M, Beail N, Saxon D. Cognitive behavioural treatment for anger in adults with intellectual disabilities: a systematic review and meta-analysis. *Journal of Applied Research in Intellectual Disabilities* 2013;**26**:47-62

Nielson WR, Weir R, Smith B, Gribbin M. Biopsychosocial approaches to the treatment of chronic pain. *Clinical Journal of Pain* 2001;**17**:S114-S27

Nieuwlaat R, Wilczynski N, Navarro T, Hobson N, Jeffery R, Keepanasseril A*, et al.* Interventions for enhancing medication adherence. *Cochrane Database Syst Rev* 2014;**(11)**:CD000011

Nieuwsma JA, Williams JW, Jr., Namdari N, Washam JB, Raitz G, Blumenthal JA*, et al.* Diagnostic Accuracy of Screening Tests and Treatment for Post-Acute Coronary Syndrome Depression: A Systematic Review. *Annals of Internal Medicine* 2017;**167**:725-35

Nolan M, Carr A. Attention deficit hyperactivity disorder. In: Carr A, editor. *What Works with Children and Adolescents? A Critical Review of Psychological Interventions with Children, Adolescents and their Families*Florence, KY: Taylor & Frances/Routledge; 2000:65-101.

Norman A, Moss TP. Psychosocial interventions for adults with visible differences: A systematic review. *PeerJ* 2015;**(3)**

Norton C, Czuber-Dochan W, Artom M, Sweeney L, Hart A. Systematic review: interventions for abdominal pain management in inflammatory bowel disease. *Alimentary Pharmacology and Therapeutics* 2017;**46**:115-25

Nowak I, Sabariego C, Switaj P, Anczewska M. Disability and recovery in schizophrenia: A systematic review of cognitive behavioral therapy interventions. *BMC Psychiatry* 2016;**16**

Nyenhuis N, Golm D, Kroner-Herwig B. A Systematic Review and Meta-Analysis on the Efficacy of Self-Help Interventions in Tinnitus. *Cognitive Behaviour Therapy* 2013;**42**:159-69

Ochentel O, Humphrey C, Pfeifer K. Efficacy of Exercise Therapy in Persons with Burnout. A Systematic Review and Meta-Analysis. *Journal of Sports Science & Medicine* 2018;**17**:475-84

O'Connor M, Halkett GK. A systematic review of interventions to reduce psychological distress in pediatric patients receiving radiation therapy. *Patient Education & Counseling* 2018;**29**:29

Odeen M, Magnussen LH, Maeland S, Larun L, Eriksen HR, Tveito TH. Systematic review of active workplace interventions to reduce sickness absence. *Occupational medicine (Oxford, England)* 2013;**63**:7-16

Oei TPS, Dingle G. The effectiveness of group cognitive behaviour therapy for unipolar depressive disorders. *Journal of Affective Disorders* 2008;**107**:5-21

Okoniewski W, Lu KD, Forno E. Weight Loss for Children and Adults with Obesity and Asthma: A Systematic Review of Randomized Controlled Trials. *Annals of the American Thoracic Society* 2019;**03**:03

Okumura Y, Ichikura K. Efficacy and acceptability of group cognitive behavioral therapy for depression: A systematic review and meta-analysis. *Journal of Affective Disorders* 2014;**164**:155-64

Olatunji BO, Davis ML, Powers MB, Smits JAJ. Cognitive-behavioral therapy for obsessive-compulsive disorder: A meta-analysis of treatment outcome and moderators. *Journal of Psychiatric Research* 2013;**47**:33-41

Oliver K, Cronan TA, Walen HR. A review of multidisciplinary interventions for fibromyalgia patients: where do we go from here? *Journal of Musculoskeletal Pain* 2001;**9**:63-80

Olsson KL, Cooper RL, Nugent WR, Reid RC. Addressing negative affect in substance use relapse prevention. *Journal of Human Behavior in the Social Environment* 2016;**26**:2-14

Ost LG, Havnen A, Hansen B, Kvale G. Cognitive behavioral treatments of obsessive-compulsive disorder. A systematic review and meta-analysis of studies published 1993-2014. *Clinical Psychology Review* 2015;**40**:156-69

Othman A, Blunden S. Psychological interventions for parents of children who have cancer: A meta-analytic review. *Current Pediatric Reviews* 2009;**5**:118-27

O'Toole MS, Zachariae R, Renna ME, Mennin DS, Applebaum A. Cognitive behavioral therapies for informal caregivers of patients with cancer and cancer survivors: a systematic review and meta-analysis. *Psycho-Oncology* 2017;**26**:428-37

Ougrin D. Efficacy of exposure versus cognitive therapy in anxiety disorders: Systematic review and meta-analysis. *BMC Psychiatry* 2011;**11**:200

Oustric P, Gibbons C, Beaulieu K, Blundell J, Finlayson G. Changes in food reward during weight management interventions - a systematic review. *Obesity Reviews* 2018;**19**:1642-58

Oyebode JR, Parveen S. Psychosocial interventions for people with dementia: An overview and commentary on recent developments. *Dementia* 2016;**04**:04

Ozabaci N. Cognitive behavioural therapy for violent behaviour in children and adolescents: a meta-analysis. *Children and Youth Services Review* 2011;**33**:1989-93

Paintain E, Cassidy S. First-line therapy for post-traumatic stress disorder: A systematic review of cognitive behavioural therapy and psychodynamic approaches. *Counselling & Psychotherapy Research* 2018;**18**:237-50

Parahoo K, McDonough S, McCaughan E, Noyes J, Semple C, Halstead EJ, *et al.* Psychosocial interventions for men with prostate cancer. *Cochrane Database Syst Rev* 2013;**24(12)**:CD008529

Parahoo K, McDonough S, McCaughan E, Noyes J, Semple C, Halstead EJ, *et al.* Psychosocial interventions for men with prostate cancer: A Cochrane systematic review. *BJU International* 2015;**116**:174-83

Parcesepe AM, Martin SL, Pollock MD, Garcia-Moreno C. The effectiveness of mental health interventions for adult female survivors of sexual assault: A systematic review. *Aggression and Violent Behavior* 2015;**25(A)**:15-25

Paul CL, Carey ML, Sanson-Fisher RW, Houlcroft LE, Turon HE. The impact of web-based approaches on psychosocial health in chronic physical and mental health conditions. *Health education research* 2013;**28**:450-71

Paulik G. The role of social schema in the experience of auditory hallucinations: a systematic review and a proposal for the inclusion of social schema in a cognitive behavioural model of voice hearing. *Clinical psychology & psychotherapy* 2012;**19**:459-72

Payne KT, Marcus DK. The efficacy of group psychotherapy for older adult clients: A meta-analysis. *Group Dynamics: Theory, Research, and Practice* 2008;**12**:268-78

Pearson FS, Lipton DS. A meta-analytic review of the effectiveness of corrections-based treatments for drug abuse. *The Prison Journal* 1999;**79**:384-410

Pengel HM, Maher CG, Refshauge KM. Systematic review of conservative interventions for subacute low back pain. *Clinical Rehabilitation* 2002;**16**:811-20

Perkes SJ, Bowman J, Penkala S. Psychological therapies for the management of co-morbid depression following a spinal cord injury: A systematic review. *Journal of Health Psychology* 2014;**19**:1597-612

Pham H, Torres H, Sharma P. Mental health implications in bladder cancer patients: A review. *Urologic Oncology* 2019;**37**:97-107

Phianmongkhol Y, Thongubon K, Woottiluk P. Effectiveness of Cognitive Behavioral Therapy Techniques for Control of Pain in Lung Cancer Patients: An Integrated Review. *Asian Pacific journal of cancer prevention* 2015;**16**:6033-8

Phillips AS. *A meta-analysis of treatments for pediatric obsessive-compulsive disorder*: Kansas State University; 2004.

Pinquart M, Duberstein PR, Lyness JM. Effects of psychotherapy and other behavioral interventions on clinically depressed older adults: a meta-analysis. *Aging & Mental Health* 2007;**11**:645-57

Pomaki G, Franche RL, Murray E, Khushrushahi N, Lampinen TM. Workplace-based work disability prevention interventions for workers with common mental health conditions: a review of the literature. *Journal of Occupational Rehabilitation* 2012;**22**:182-95

Poole JL, Siegel P. Effectiveness of Occupational Therapy Interventions for Adults With Fibromyalgia: A Systematic Review. *American Journal of Occupational Therapy* 2017;**71**:1-10

Posadzki P, Choi J, Lee MS, Ernst E. Yoga for addictions: a systematic review of randomised clinical trials. *Focus on Alternative & Complementary Therapies* 2014;**19**:1-8

Preti A, Cella M. Randomized-controlled trials in people at ultra high risk of psychosis: A review of treatment effectiveness. *Schizophrenia Research* 2010;**123**:30-6

Qaseem A, Barry MJ, Kansagara D, Forciea MA, Denberg TD, Boyd C*, et al.* Nonpharmacologic versus pharmacologic treatment of adult patients with major depressive disorder: A clinical practice guideline from the American College of Physicians. *Annals of Internal Medicine* 2016;**164**:350-9

Qaseem A, Kansagara D, Forciea MA, Cooke M, Denberg TD, Barry MJ*, et al.* Management of chronic insomnia disorder in adults: A clinical practice guideline from the American college of physicians. *Annals of Internal Medicine* 2016;**165**:125-33

Qaseem A, Wilt TJ, McLean RM, Forciea MA, Clinical Guidelines Committee of the American College of P. Noninvasive Treatments for Acute, Subacute, and Chronic Low Back Pain: A Clinical Practice Guideline From the American College of Physicians. *Annals of Internal Medicine* 2017;**166**:514-30

Quinn J, Kolla NJ. From Clozapine to Cognitive Remediation: A Review of Biological and Psychosocial Treatments for Violence in Schizophrenia. *Canadian Journal of Psychiatry* 2017;**62**:94-101

Raine R, Haines A, Sensky T, Hutchings A, Larkin K, Black N. Systematic review of mental health interventions for patients with common somatic symptoms: Can research evidence from secondary care be extrapolated to primary care? *British Medical Journal* 2002;**325**:1082-5

Rakofsky JJ, Dunlop BW. Treating nonspecific anxiety and anxiety disorders in patients with bipolar disorder: A review. *Journal of Clinical Psychiatry* 2011;**72**:81-90

Ranasinghe I, Sin J. A systematic review of evidence-based treatment for individuals with treatment-resistant schizophrenia and a suboptimal response to clozapine monotherapy. *Psychosis: Psychological, Social and Integrative Approaches* 2014;**6**:253-65

Reavley N, Jorm AF. Prevention and early intervention to improve mental health in higher education students: A review. *Early Intervention in Psychiatry* 2010;**4**:132-42

Reddy LA, De Thomas CA, Newman E, Chun V. School-based prevention and intervention programs for children with emotional disturbance: A review of treatment components and methodology. *Psychology in the Schools* 2009;**46**:132-53

Redondo S, Sanchez-Meca J, Garrido V. The influence of treatment programmes on the recidivism of juvenile and adult offenders: An European meta-analytic review. *Psychology, Crime & Law* 1999;**5**:251-78

Rees G, Ponczek E, Hassell J, Keeffe JE, Lamoureux EL. Psychological outcomes following interventions for people with low vision: A systematic review. *Expert Review of Ophthalmology* 2010;**5**:385-403

Renton T, Tang H, Ennis N, Cusimano MD, Bhalerao S, Schweizer TA*, et al.* Web-based intervention programs for depression: A scoping review and evaluation. *Journal of Medical Internet Research* 2014;**16(9)**:50-71

Richards MC, Ford JJ, Slater SL, Hahne AJ, Surkitt LD, Davidson M*, et al.* The effectiveness of physiotherapy functional restoration for post-acute low back pain: A systematic review. *Manual Therapy* 2013;**18**:4-25

Richardson A, McNoe B, Derrett S, Harcombe H. Interventions to prevent and reduce the impact of musculoskeletal injuries among nurses: A systematic review. *International Journal of Nursing Studies* 2018;**82**:58-67

Richardson J, Smith JE, McCall G, Richardson A, Pilkington K, Kirsch I. Hypnosis for nausea and vomiting in cancer chemotherapy: a systematic review of the research evidence. *European Journal of Cancer Care* 2007;**16**:402-12

Richardson KM, Rothstein HR. Effects of Occupational Stress Management Intervention Programs: A Meta-Analysis. *Journal of Occupational Health Psychology* 2008;**13**:69-93

Rihn JA, Radcliff K, Norvell DC, Eastlack R, Phillips FM, Berland D*, et al.* Comparative Effectiveness of Treatments for Chronic Low Back Pain: A Multiple Treatment Comparison Analysis. *Clinical Spine Surgery : A Spine Publication* 2017;**30**:204-25

Ritvo PG, Irvine MJ, Lindsay EA, Kraetschmer N, Blair N, Shnek ZM. A critical review of research related to family physician-assisted smoking cessation interventions. *Cancer Prevention and Control* 1997;**1(4)**:289-303

Roberts Neil P, Kitchiner Neil J, Kenardy J, Bisson Jonathan I. Early psychological interventions to treat acute traumatic stress symptoms. *Cochrane Database Syst Rev* 2010;**(3)**:CD007944

Robinson PD, Oberoi S, Tomlinson D, Duong N, Davis H, Cataudella D*, et al.* Management of fatigue in children and adolescents with cancer and in paediatric recipients of haemopoietic stem-cell transplants: a clinical practice guideline. *The Lancet Child and Adolescent Health* 2018;**2**:371-8

Rodrigues H, Figueira I, Lopes A, Goncalves R, Mendlowicz MV, Coutinho ESF*, et al.* Does d-cycloserine enhance exposure therapy for anxiety disorders in humans? A meta-analysis. *Plos One* 2014;**9(7)**:e93519

Rogers D. Which educational interventions improve healthcare professionals' resilience? *Medical Teacher* 2016;**38**:1236-41

Rometsch-Ogioun El Sount C, Windthorst P, Denkinger J, Ziser K, Nikendei C, Kindermann D*, et al.* Chronic pain in refugees with posttraumatic stress disorder (PTSD): A systematic review on patients' characteristics and specific interventions. *Journal of Psychosomatic Research* 2018;**30**:30

Roozen HG, de Waart R, van der Windt DAWM, van den Brink W, de Jong CAJ, Kerkhof AJFM. A systematic review of the effectiveness of naltrexone in the maintenance treatment of opioid and alcohol dependence. *European Neuropsychopharmacology* 2006;**16**:311-23

Rosa-Alcazar AI, Sanchez-Meca J, Rosa-Alcazar A, Iniesta-Sepulveda M, Olivares-Rodriguez J, Parada-Navas JL. Psychological treatment of obsessive-compulsive disorder in children and adolescents: a meta-analysis. *The Spanish journal of psychology* 2015;**18**:E20

Rosendal M, Blankenstein AH, Morriss R, Fink P, Sharpe M, Burton C. Enhanced care by generalists for functional somatic symptoms and disorders in primary care. *Cochrane Database Syst Rev* 2013;**(10)**:CD008142

Ross SD, Estok RP, Frame D, Stone LR, Ludensky V, Levine CB. Disability and chronic fatigue syndrome: A focus on function. *Archives of Internal Medicine* 2004;**164**:1098-107

Ruiz-Perez I, Murphy M, Pastor-Moreno G, Rojas-Garcia A, Rodriguez-Barranco M. The Effectiveness of HIV Prevention Interventions in Socioeconomically Disadvantaged Ethnic Minority Women: A Systematic Review and Meta-Analysis. *American Journal of Public Health* 2017;**107**:e13-e21

Ruotsalainen JH, Verbeek JH, Marine A, Serra C. Preventing occupational stress in healthcare workers. *Cochrane Database Syst Rev* 2014;**(11)**:CD002892

Ruotsalainen JH, Verbeek JH, Marine A, Serra C. Preventing occupational stress in healthcare workers. *Cochrane Database Syst Rev* 2015;**(4)**:CD002892

Sadowski L, Casteel C. Intimate partner violence towards women. *BMJ clinical evidence* 2010

Salathe CR, Melloh M, Crawford R, Scherrer S, Boos N, Elfering A. Treatment Efficacy, Clinical Utility, and Cost-Effectiveness of Multidisciplinary Biopsychosocial Rehabilitation Treatments for Persistent Low Back Pain: A Systematic Review. *Global Spine Journal* 2018;**8**:872-86

Salt S, Mulvaney CA, Preston NJ. Drug therapy for symptoms associated with anxiety in adult palliative care patients. *Cochrane Database Syst Rev* 2017;**(5)**:CD004596

Sanchez-Meca J, Rosa-Alcazar AI, Iniesta-Sepulveda M, Rosa-Alcazar T. Differential efficacy of cognitive-behavioral therapy and pharmacological treatments for pediatric obsessive-compulsive disorder: A meta-analysis. *Journal of Anxiety Disorders* 2014;**28**:31-44

Sanchez-Meca J, Rosa-Alcazar AI, Lopez-Soler C. The psychological treatment of sexual abuse in children and adolescents: A meta-analysis. *International Journal of Clinical and Health Psychology* 2011;**11**:67-93

Sande R, Buskens E, Allart E, Graaf Y, Engeland H. Psychosocial intervention following suicide attempt: a systematic review of treatment interventions. *Acta Psychiatrica Scandinavica* 1997;**96**:43-50

Sankar A, Melin A, Lorenzetti V, Horton P, Costafreda SG, Fu CHY. A systematic review and meta-analysis of the neural correlates of psychological therapies in major depression. *Psychiatry Research: Neuroimaging* 2018;**279**:31-9

Satapathy S, Kaushal T, Bakhshi S, Chadda RK. Non-pharmacological Interventions for Pediatric Cancer Patients: A Comparative Review and Emerging Needs in India. *Indian Pediatrics* 2018;**55**:225-32

Savage J, Waddell A. Tinnitus. *Clinical Evidence* 2014;**20**:20

Scaini S, Belotti R, Ogliari A, Battaglia M. A comprehensive meta-analysis of cognitive-behavioral interventions for social anxiety disorder in children and adolescents. *Journal of Anxiety Disorders* 2016;**42**:105-12

Scanlan JN. Interventions to reduce the use of seclusion and restraint in inpatient psychiatric settings: what we know so far a review of the literature. *International Journal of Social Psychiatry* 2010;**56**:412-23

Schaafsma F, Schonstein E, Ojajarvi A, Verbeek J. Physical conditioning programs for improving work outcomes among workers with back pain. *Scandinavian Journal of Work, Environment and Health, Supplement* 2011;**37**:1-5

Schaefert R, Hausteiner-Wiehle C, Hauser W, Ronel J, Herrmann M, Henningsen P. Non-specific, functional, and somatoform bodily complaints. *Deutsches Arzteblatt International* 2012;**109**:803-13

Scheer SJ, Watanabe TK, Radack KL. Randomized controlled trials in industrial low back pain. Part 3. Subacute/chronic pain interventions. *Archives of Physical Medicine and Rehabilitation* 1997;**78**:414-23

Schirmbeck F, Zink M. Cognitive behavioural therapy for obsessive-compulsive symptoms in schizophrenia. *The Cognitive Behaviour Therapist* 2013;**6**

Schleider JL, Weisz JR. Little Treatments, Promising Effects? Meta-Analysis of Single-Session Interventions for Youth Psychiatric Problems. *Journal of the American Academy of Child & Adolescent Psychiatry* 2017;**56**:107-15

Schmucker M, Losel F. Does sexual offender treatment work? A systematic review of outcome evaluations. *Psicothema* 2008;**20**:10-9

Schmucker M, Losel F. The effects of sexual offender treatment on recidivism: An international meta-analysis of sound quality evaluations. *Journal of Experimental Criminology* 2015;**11**:597-630

Schneider M, Vernon H, Ko G, Lawson G, Perera J. Chiropractic management of fibromyalgia syndrome: a systematic review of the literature. *Journal of Manipulative & Physiological Therapeutics* 2009;**32**:25-40

Schneider RL, Arch JJ, Wolitzky-Taylor KB. The state of personalized treatment for anxiety disorders: A systematic review of treatment moderators. *Clinical Psychology Review* 2015;**38**:39-54

Schonstein E, Kenny D, Keating J, Koes B, Herbert RD. Physical conditioning programs for workers with back and neck pain: a Cochrane Systematic Review. *Spine* 2003;**28**:E391-5

Schonstein E, Kenny DT, Keating J, Koes BW. Work conditioning, work hardening and functional restoration for workers with back and neck pain. *Cochrane Database Syst Rev* 2003;**(1)**:CD001822

Schroeck JL, Ford J, Conway EL, Kurtzhalts KE, Gee ME, Vollmer KA*, et al.* Review of Safety and Efficacy of Sleep Medicines in Older Adults. *Clinical Therapeutics* 2016;**38**:2340-72

Schwartz C, Schlegl S, Kuelz AK, Voderholzer U. Treatment-seeking in OCD community cases and psychological treatment actually provided to treatment-seeking patients: A systematic review. *Journal of Obsessive-Compulsive and Related Disorders* 2013;**2**:448-56

Seda G, Sanchez-Ortuno MM, Welsh CH, Halbower AC, Edinger JD. Comparative meta-analysis of prazosin and imagery rehearsal therapy for nightmare frequency, sleep quality, and posttraumatic stress. *Journal of Clinical Sleep Medicine* 2015;**11**:11-22

Seferiadis A, Rosenfeld M, Gunnarsson R. A review of treatment interventions in whiplash-associated disorders. *European Spine Journal* 2004;**13**:387-97

Segool NK, Carlson JS. Efficacy of cognitive-behavioral and pharmacological treatments for children with social anxiety. *Depression and Anxiety* 2008;**25**:620-31

Segredou I, Xenitidis K, Panagiotopoulou M, Bochtsou V, Antoniadou O, Livaditis M. Group psychosocial interventions for adults with schizophrenia and bipolar illness: the evidence base in the light of publications between 1986 and 2006. *The International journal of social psychiatry* 2012;**58**:229-38

Sepede G, Sarchione F, Matarazzo I, Di Giannantonio M, Salerno RM. Premenstrual dysphoric disorder without comorbid psychiatric conditions: A systematic review of therapeutic options. *Clinical Neuropharmacology* 2016;**39**:241-61

Sereda M, Xia J, El Refaie A, Hall DA, Hoare DJ. Sound therapy (using amplification devices and/or sound generators) for tinnitus. *Cochrane Database Syst Rev* 2018;**12**:CD013094

Sesel AL, Sharpe L, Naismith SL. Efficacy of Psychosocial Interventions for People with Multiple Sclerosis: A Meta-Analysis of Specific Treatment Effects. *Psychotherapy and Psychosomatics* 2018;**87**:105-11

Sharma E, Thennarasu K, Reddy YCJ. Long-term outcome of obsessive-compulsive disorder in adults: A meta-analysis. *Journal of Clinical Psychiatry* 2014;**75**:1019-27

Shen J, Rouse J, Godbole M, Wells HL, Boppana S, Schwebel DC. Systematic Review: Interventions to Educate Children About Dog Safety and Prevent Pediatric Dog-Bite Injuries: A Meta-Analytic Review. *Journal of Pediatric Psychology* 2017;**42**:779-91

Shergis JL, Ni X, Jackson ML, Zhang AL, Guo X, Li Y*, et al.* A systematic review of acupuncture for sleep quality in people with insomnia. *Complementary Therapies in Medicine* 2016;**26**:11-20

Sherr L, Clucas C, Harding R, Sibley E, Catalan J. HIV and Depression - a systematic review of interventions. *Psychology, Health & Medicine* 2011;**16**:493-527

Shingler E, Robles LA, Perry R, Penfold C, Ness AR, Thomas S*, et al.* Systematic review evaluating randomized controlled trials of smoking and alcohol cessation interventions in people with head and neck cancer and oral dysplasia. *Head and Neck* 2018;**40(8)**:1845-53

Sim J, Adams N. Systematic review of randomized controlled trials of nonpharmacological interventions for fibromyalgia. *Clinical Journal of Pain* 2002;**18**:324-36

Simon SS, Cordas TA, Bottino CMC. Cognitive behavioral therapies in older adults with depression and cognitive deficits: A systematic review. *International Journal of Geriatric Psychiatry* 2015;**30**:223-33

Singer GH, Ethridge BL, Aldana SI. Primary and secondary effects of parenting and stress management interventions for parents of children with developmental disabilities: a meta-analysis. *Mental Retardation and Developmental Disabilities Research Reviews* 2007;**13**:357-69

Singh N, Reece J. Psychotherapy, pharmacotherapy, and their combination for adolescents with major depressive disorder: A meta-analysis. *The Australian Educational and Developmental Psychologist* 2014;**31**:47-65

Sivaraman B, Nye E, Bowes L. School-based anti-bullying interventions for adolescents in low- and middle-income countries: A systematic review. *Aggression and Violent Behavior* 2018;**45**:154-62

Slotema CW, Blom JD, Niemantsverdriet MBA, Sommer IEC. Auditory Verbal Hallucinations in Borderline Personality Disorder and the Efficacy of Antipsychotics: A Systematic Review. *Frontiers in psychiatry Frontiers Research Foundation* 2018;**9**:347

Smith CA, Armour M, Lee MS, Wang LQ, Hay PJ. Acupuncture for depression. *Cochrane Database Syst Rev* 2018;**(3)**:CD004046

Smith CA, Hay PP. Acupuncture for depression. *Cochrane Database Syst Rev* 2005;**(2)**:CD004046

Smith CA, Hay PP, MacPherson H. Acupuncture for depression. *Cochrane Database Syst Rev* 2010;**(1)**:CD004046

Smith DP, Dunn KI, Harvey PW, Battersby MW, Pols RG. Assessing Randomised Clinical Trials of Cognitive and Exposure Therapies for Gambling Disorders: A Systematic Review. *Behaviour Change* 2013;**30**:139-58

Smits JAJ, Hofmann SG. A meta-analytic review of the effects of psychotherapy control conditions for anxiety disorders. *Psychological Medicine* 2009;**39**:229-39

Soomro GM. Obsessive compulsive disorder. *BMJ clinical evidence* 2012:pii: 1004

Spahn JM, Reeves RS, Keim KS, Laquatra I, Kellogg M, Jortberg B*, et al.* State of the evidence regarding behavior change theories and strategies in nutrition counseling to facilitate health and food behavior change. *Journal of the American Dietetic Association* 2010;**110**:879-91

Spence JD, Barnett PA, Linden W, Ramsden V, Taenzer P. Recommendations on stress management. *Cmaj* 1999;**160**:S46-S50

Spiller R, Aziz Q, Creed F, Emmanuel A, Houghton L, Hungin P*, et al.* Guidelines on the irritable bowel syndrome: Mechanisms and practical management. *Gut* 2007;**56**:1770-98

Spoelstra SL, Schueller M, Hilton M, Ridenour K. Interventions combining motivational interviewing and cognitive behaviour to promote medication adherence: a literature review. *Journal of Clinical Nursing* 2015;**24**:1163-73

Springer KS, Levy HC, Tolin DF. Remission in CBT for adult anxiety disorders: A meta-analysis. *Clinical Psychology Review* 2018;**61**:1-8

Stanton A, Grimshaw G. Tobacco cessation interventions for young people. *Cochrane Database Syst Rev* 2013;**(8)**:CD003289

Steel JL, Bress K, Popichak L, Evans JS, Savkova A, Biala M*, et al.* A systematic review of randomized controlled trials testing the efficacy of psychosocial interventions for gastrointestinal cancers. *Journal of Gastrointestinal Cancer* 2014;**45**:181-9

Steinert C, Hofmann M, Leichsenring F, Kruse J. What do we know today about the prospective long-term course of social anxiety disorder? A systematic literature review. *Journal of Anxiety Disorders* 2013;**27**:692-702

Sullivan PJ. *A meta-analysis of the effectiveness and efficiency of dcycloserine-augmented exposure therapy with treatment resistant pediatric OCD patients*: Palo Alto University; 2018.

Sumathipala A. What is the evidence for the efficacy of treatments for somatoform disorders? A critical review of previous intervention studies. *Psychosomatic Medicine* 2007;**69**:889-900

Tan L, Wang MJ, Modini M, Joyce S, Mykletun A, Christensen H*, et al.* Preventing the development of depression at work: A systematic review and meta-analysis of universal interventions in the workplace. *BMC Medicine* 2014;**12(1)**

Tarrier N, Taylor K, Gooding P. Cognitive-behavioral interventions to reduce suicide behavior: A systematic review and meta-analysis. *Behavior Modification* 2008;**32**:77-108

Taylor TL, Killaspy H, Wright C, Turton P, White S, Kallert TW*, et al.* A systematic review of the international published literature relating to quality of institutional care for people with longer term mental health problems. *BMC Psychiatry* 2009;**9**:55

Thabane L, Chu R, Cuddy K, Douketis J. What is the quality of reporting in weight loss intervention studies? A systematic review of randomized controlled trials. *International Journal of Obesity* 2007;**31**:1554-9

Thombs BD, de Jonge P, Coyne JC, Whooley MA, Frasure-Smith N, Mitchell AJ*, et al.* Depression screening and patient outcomes in cardiovascular care: a systematic review. *JAMA* 2008;**300**:2161-71

Tobon JI, Ouimet AJ, Dozois DJA. Attentional bias in anxiety disorders following cognitive behavioral treatment. *Journal of Cognitive Psychotherapy* 2011;**25**:114-29

Tolin DF. Is cognitive-behavioral therapy more effective than other therapies?. A meta-analytic review. *Clinical Psychology Review* 2010;**30**:710-20

Tolin DF. Can Cognitive Behavioral Therapy for Anxiety and Depression Be Improved with Pharmacotherapy? A Meta-analysis. *Psychiatric Clinics of North America* 2017;**40**:715-38

Tolin DF, Frost RO, Steketee G, Muroff J. Cognitive behavioral therapy for hoarding disorder: A meta-analysis. *Depression and Anxiety* 2015;**32**:158-66

Tumur I, Kaltenthaler E, Ferriter M, Beverley C, Parry G. Computerised cognitive behaviour therapy for obsessive-compulsive disorder: A systematic review. *Psychotherapy and Psychosomatics* 2007;**76**:196-202

Turner BJ, Austin SB, Chapman AL. Treating nonsuicidal self-injury: a systematic review of psychological and pharmacological interventions. *Canadian Journal of Psychiatry* 2014;**59**:576-85

Turner W, Macdonald GM, Dennis JA. Behavioural and cognitive behavioural training interventions for assisting foster carers in the management of difficult behaviour. *Cochrane Database Syst Rev* 2007;**(1)**:CD003760

Van Cauwenbergh D, De Kooning M, Ickmans K, Nijs J. How to exercise people with chronic fatigue syndrome: Evidence-based practice guidelines. *European Journal of Clinical Investigation* 2012;**42**:1136-44

van den Heuvel JF, Groenhof TK, Veerbeek JH, van Solinge WW, Lely AT, Franx A*, et al.* eHealth as the Next-Generation Perinatal Care: An Overview of the Literature. *Journal of Medical Internet Research* 2018;**20**:e202

Van Der Sande R, Buskens E, Allart E, Van Der Graaf Y, Van Engeland H. Psychosocial intervention following suicide attempt: A systematic review of treatment interventions. *Acta Psychiatrica Scandinavica* 1997;**96**:43-50

van der Straten AL, Denys D, van Wingen GA. Impact of treatment on resting cerebral blood flow and metabolism in obsessive compulsive disorder: a meta-analysis. *Scientific Reports* 2017;**7**:17464

Vancampfort D, Vanderlinden J, De Hert M, Adamkova M, Skjaerven LH, Catalan-Matamoros D*, et al.* A systematic review on physical therapy interventions for patients with binge eating disorder. *Disability and rehabilitation* 2013;**35**:2191-6

Vandborg SK, Hartmann TB, Bennedsen BE, Pedersen AD, Eskildsen A, Videbech PB*, et al.* Do cognitive functions in obsessive-compulsive disorder change after treatment? A systematic review and a double case report. *Nordic Journal of Psychiatry* 2012;**66**:60-7

Vaughn MG, Howard MO. Adolescent substance abuse treatment: a synthesis of controlled evaluations. *Research on Social Work Practice* 2004;**14**:325-35

Veale D, Naismith I, Miles S, Gledhill LJ, Stewart G, Hodsoll J. Outcomes for residential or inpatient intensive treatment of obsessive-compulsive disorder: A systematic review and meta-analysis. *Journal of Obsessive-Compulsive and Related Disorders* 2016;**8**:38-49

Veehof MM, Oskam MJ, Schreurs KM, Bohlmeijer ET. Acceptance-based interventions for the treatment of chronic pain: a systematic review and meta-analysis. *Pain* 2011;**152**:533-42

Verheul R, Herbrink M. The efficacy of various modalities of psychotherapy for personality disorders: A systematic review of the evidence and clinical recommendations. *International Review of Psychiatry* 2007;**19**:25-38

Verhey R, Chibanda D, Brakarsh J, Seedat S. Psychological interventions for post-traumatic stress disorder in people living with HIV in Resource poor settings: a systematic review. *Tropical Medicine and International Health* 2016;**21**:1198-208

Vittengl JR, Clark LA, Dunn TW, Jarrett RB. Reducing relapse and recurrence in unipolar depression: a comparative meta-analysis of cognitive-behavioral therapy's effects. *Journal of Consulting and Clinical Psychology* 2007;**75**:475-88

Vocks S, Tuschen-Caffier B, Pietrowsky R, Rustenbach SJ, Kersting A, Herpertz S. Meta-analysis of the effectiveness of psychological and pharmacological treatments for binge eating disorder. *International Journal of Eating Disorders* 2010;**43**:205-17

Voshaar RCO, Couvee JE, Van Balkom AJLM, Mulder PGH, Zitman FG. Strategies for discontinuing long-term benzodiazepine use: Meta-analysis. *British Journal of Psychiatry* 2006;**189**:213-20

Wade AG. Use of the internet to assist in the treatment of depression and anxiety: A systematic review. *Primary Care Companion to the Journal of Clinical Psychiatry* 2010;**12**:e1-e11

Wagner KD, Unger JB, Bluthenthal RN, Andreeva VA, Pentz MA. Cognitive behavioral theories used to explain injection risk behavior among injection drug users: a review and suggestions for the integration of cognitive and environmental models. *Health Education & Behavior* 2010;**37**:504-32

Walker DF, McGovern SK, Poey EL, Otis KE. Treatment effectiveness for male adolescent sexual offenders: a meta-analysis and review. *Journal of Child Sexual Abuse* 2004;**13**:281-93

Wariki WM, Ota E, Mori R, Koyanagi A, Hori N, Shibuya K. Behavioral interventions to reduce the transmission of HIV infection among sex workers and their clients in low- and middle-income countries. *Cochrane Database Syst Rev* 2012;**(2)**:CD005272

Warner JP, Butler R, Gupta S. Dementia. *BMJ clinical evidence* 2010:pii: 1001

Watanabe N, Churchill R, Furukawa TA. Combined psychotherapy plus benzodiazepines for panic disorder. *Cochrane Database Syst Rev* 2009;**(1)**:CD005335

Wegner I, Hall DA, Smit AL, McFerran D, Stegeman I. Betahistine for tinnitus. *Cochrane Database Syst Rev* 2018;**(12)**:CD013093

Weisz JR, Kuppens S, Ng MY, Eckshtain D, Ugueto AM, Vaughn-Coaxum R*, et al.* What five decades of research tells us about the effects of youth psychological therapy: A multilevel meta-analysis and implications for science and practice. *The American psychologist* 2017;**72**:79-117

Werneke U, Taylor D, Sanders TAB, Wessely S. Behavioural management of antipsychotic-induced weight gain: A review. *Acta Psychiatrica Scandinavica* 2003;**108**:252-9

Wheeler S, Acord-Vira A, Davis D. Effectiveness of Interventions to Improve Occupational Performance for People With Psychosocial, Behavioral, and Emotional Impairments After Brain Injury: A Systematic Review. *The American journal of occupational therapy* 2016;**70**:p1-p9

Williams M, Viscusi JA. Hoarding Disorder and a Systematic Review of Treatment with Cognitive Behavioral Therapy. *Cognitive Behaviour Therapy* 2016;**45**:93-110

Wilson DB, Gottfredson DC, Najaka SS. School-Based Prevention of Problem Behaviors: A Meta-Analysis. *Journal of Quantitative Criminology* 2001;**17**:247-

Winkley K, Landau S, Eisler I, Ismail K. Psychological interventions to improve glycaemic control in patients with type 1 diabetes: Systematic review and meta-analysis of randomised controlled trials. *British Medical Journal* 2006;**333**:65-8

Winter D, Bradshaw S, Bunn F, Wellsted D. A systematic review of the literature on counselling and psychotherapy for the prevention of suicide: 1. Quantitative outcome and process studies. *Counselling & Psychotherapy Research* 2013;**13**:164-83

Witt K, de Moraes DP, Salisbury TT, Aren SME, Gunnell D, Hazell P*, et al.* Treatment as usual (TAU) as a control condition in trials of cognitive behavioural-based psychotherapy for self-harm: Impact of content and quality on outcomes in a systematic review. *Journal of Affective Disorders* 2018;**235**:434-47

Woodruff SC. *The effects of mindfulness and acceptance-based interventions and cognitive-behavioral interventions on positive and negative affect: A meta-analysis*: The Catholic University of America; 2015.

Wozney L, Huguet A, Bennett K, Radomski AD, Hartling L, Dyson M*, et al.* How do eHealth Programs for Adolescents With Depression Work? A Realist Review of Persuasive System Design Components in Internet-Based Psychological Therapies. *Journal of Medical Internet Research* 2017;**19**:e266

Yorke J, Fleming SL, Shuldham C. Psychological interventions for adults with asthma. *Cochrane Database Syst Rev* 2006;**(1)**:CD007676

Young SC. *A failure to self-regulate? a research synthesis of the cognitive-behavioral literature targeting the improvement of self-regulation among school-age males*: Regent University; 2015.

Yusufov M, Nicoloro-SantaBarbara J, Grey NE, Moyer A, Lobel M. Meta-analytic evaluation of stress reduction interventions for undergraduate and graduate students. *International Journal of Stress Management* 2019;**26(2)**:132-45

Zakrzewska J, Buchanan JA. Burning mouth syndrome. *Clinical Evidence* 2016;**07**:07

Zakrzewska JM, Forssell H, Glenny A. Interventions for the treatment of burning mouth syndrome. *Cochrane Database Syst Rev* 2005;**(1)**:CD002779

Zalta AK. A meta-analysis of anxiety symptom prevention with cognitive-behavioral interventions. *Journal of Anxiety Disorders* 2011;**25**:749-60

Zech N, Hansen E, Bernardy K, Häuser W, Häuser W. Efficacy, acceptability and safety of guided imagery/hypnosis in fibromyalgia - A systematic review and meta-analysis of randomized controlled trials. *European Journal of Pain* 2017;**21**:217-27

Zeppegno P, Gattoni E, Mastrangelo M, Gramaglia C, Sarchiapone M. Psychosocial Suicide Prevention Interventions in the Elderly: A Mini-Review of the Literature. *Frontiers in Psychology* 2018;**9**:2713

Zhang H, Zhang Y, Yang L, Yuan S, Zhou X, Pu J*, et al.* Efficacy and acceptability of psychotherapy for anxious young children a meta-analysis of randomized controlled trials. *Journal of Nervous and Mental Disease* 2017;**205**:931-41

d. References of studies excluded due no CBT RCT summary (520)

Aafjes-van Doorn K, Barber JP. Systematic Review of In-Session Affect Experience in Cognitive Behavioral Therapy for Depression. *Cognitive Therapy and Research* 2017;**41**:807-28

Aderka IM, Nickerson A, Boe HJ, Hofmann SG. Sudden gains during psychological treatments of anxiety and depression: A meta-analysis. *Journal of Consulting and Clinical Psychology* 2012;**80**:93-101

Aggarwal VR, Tickle M, Javidi H, Peters S. Reviewing the evidence: can cognitive behavioral therapy improve outcomes for patients with chronic orofacial pain? *Journal of Orofacial Pain* 2010;**24**:163-71
Alam-Mehrjerdi Z, Daneshmand R, Samiei M, Samadi R, Abdollahi M, Dolan K. Women-only drug treatment services and needs in Iran: The first review of current literature. *DARU* 2016;**24(3)**

Aldi GA, Bertoli G, Ferraro F, Pezzuto A, Cosci F. Effectiveness of pharmacological or psychological interventions for smoking cessation in smokers with major depression or depressive symptoms: A systematic review of the literature. *Substance Abuse* 2018;**39(3)**:289-306

Alexander H. *Coping with sickle cell disease using cognitive behavior therapy*: Walden University; 2018.

Allen S, Dalton WT. Treatment of eating disorders in primary care: a systematic review. *Journal of Health Psychology* 2011;**16**:1165-76

Altayar O, Sharma V, Prokop LJ, Sood A, Murad MH. Psychological Therapies in Patients with Irritable Bowel Syndrome: A Systematic Review and Meta-Analysis of Randomized Controlled Trials. *Gastroenterology Research and Practice* 2015;**2015**:549308

Amand A, Bard DE, Silovsky JF. Meta-analysis of treatment for child sexual behavior problems: practice elements and outcomes. *Child Maltreatment* 2008;**13(2)**:145-66

Andersson G, Rozental A, Shafran R, Carlbring P. Long-term effects of internet-supported cognitive behaviour therapy. *Expert Review of Neurotherapeutics* 2018;**18**:21-8

Andreasson S, Ojehagen A. Psychosocial treatment for alcohol dependence. In: Berglund M, Thelander S, Johnsson E, editors. *Treating Alcohol and Drug Abuse: An Evidence Based Review*: Wiley-VCH Veriag GmbH and Co KGaA; 2003:43-188.

Apolinario-Hagen J. Internet-Delivered Psychological Treatment Options for Panic Disorder: A Review on Their Efficacy and Acceptability. *Psychiatry Investigation* 2018;**20**:20

Arroyo K, Lundahl B, Butters R, Vanderloo M, Wood DS. Short-Term Interventions for Survivors of Intimate Partner Violence. *Trauma, Violence & Abuse* 2017;**18**:155-71

Astin JA, Beckner W, Soeken K, Hochberg MC, Berman B. Psychological interventions for rheumatoid arthritis: a meta-analysis of randomized controlled trials. *Arthritis & Rheumatism: Arthritis Care & Research* 2002;**47**:291-302

Aydin A. Parental involvement in cognitive-behavioral therapy for children with anxiety disorders. *Turk Psikiyatri Dergisi* 2014;**25**:181-9

Ayers CR, Sorrell JT, Thorp SR, Wetherell JL. Evidence-based psychological treatments for late-life anxiety. *Psychology and Aging* 2007;**22**:8-17

Baandrup L, Ostrup Rasmussen J, Klokker L, Austin S, Bjornshave T, Fuglsang Bliksted V*, et al.* Treatment of adult patients with schizophrenia and complex mental health needs - A national clinical guideline. *Nordic Journal of Psychiatry* 2016;**70**:231-40

Baker AL, Thornton LK, Hiles S, Hides L, Lubman DI. Psychological interventions for alcohol misuse among people with co-occurring depression or anxiety disorders: a systematic review. *Journal of Affective Disorders* 2012;**139**:217-29

Ballesio A, Aquino M, Feige B, Johann AF, Kyle SD, Spiegelhalder K*, et al.* The effectiveness of behavioural and cognitive behavioural therapies for insomnia on depressive and fatigue symptoms: A systematic review and network meta-analysis. *Sleep Medicine Reviews* 2018;**37**:114-29

Bandelow B, Lichte T, Rudolf S, Wiltink J, Beutel ME. The diagnosis of and treatment recommendations for anxiety disorders. *Deutsches Arzteblatt International* 2014;**111**:473-80

Bandelow B, Reitt M, Rover C, Michaelis S, Gorlich Y, Wedekind D. Efficacy of treatments for anxiety disorders: A meta-analysis. *International Clinical Psychopharmacology* 2015;**30**:183-92

Bandelow B, Sagebiel A, Belz M, Gorlich Y, Michaelis S, Wedekind D. Enduring effects of psychological treatments for anxiety disorders: meta-analysis of follow-up studies. *British Journal of Psychiatry* 2018;**212**:333-8

Baraniak A, Sheffield D. The efficacy of psychologically based interventions to improve anxiety, depression and quality of life in COPD: A systematic review and meta-analysis. *Patient Education & Counseling* 2011;**83**:29-36

Barkowski S, Schwartze D, Strauss B, Burlingame GM, Barth J, Rosendahl J. Efficacy of group psychotherapy for social anxiety disorder: A meta-analysis of randomized-controlled trials. *Journal of Anxiety Disorders* 2016;**39**:44-64

Barlow J, Bergman H, Kornor H, Wei Y, Bennett C. Group-based parent training programmes for improving emotional and behavioural adjustment in young children. *Cochrane Database Syst Rev* 2016;**(8)**:CD003680

Barlow J, Smailagic N, Huband N, Roloff V, Bennett C. Group-based parent training programmes for improving parental psychosocial health. *Cochrane Database Syst Rev* 2012;**6**:CD002020

Barlow JH, Ellard DR, Hainsworth JM, Jones FR, Fisher A. A review of self-management interventions for panic disorders, phobias and obsessive-compulsive disorders. *Acta Psychiatrica Scandinavica* 2005;**111**:272-85

Barrett PM, Farrell L, Pina AA, Peris TS, Piacentini J. Evidence-based psychosocial treatments for child and adolescent obsessive-compulsive disorder. *Journal of Clinical Child and Adolescent Psychology* 2008;**37**:131-55

Barth J, Munder T, Gerger H, Nuesch E, Trelle S, Znoj H*, et al.* Comparative Efficacy of Seven Psychotherapeutic Interventions for Patients with Depression: A Network Meta-Analysis. *PLoS Medicine* 2013;**10(5)**:e1001454

Beck R, Fernandez E. Cognitive-behavioral therapy in the treatment of anger: A meta-analysis. *Cognitive Therapy and Research* 1998;**22**:63-74

Belleville G, Cousineau H, Levrier K, St-Pierre-Delorme ME, Marchand A. The impact of cognitive-behavior therapy for anxiety disorders on concomitant sleep disturbances: a meta-analysis. *Journal of Anxiety Disorders* 2010;**24**:379-86

Bennett DS, Gibbons TA. Efficacy of child cognitive-behavioral interventions for antisocial behavior: a meta-analysis. *Child and Family Behavior Therapy* 2000;**22**:1-15

Berkman ND, Bulik CM, Brownley KA, Lohr KN, Sedway JA, Rooks A*, et al.* Management of eating disorders. *Evidence Report/Technology Assessment* 2006;**(135)**:1-166

Bernardy K, Klose P, Welsch P, Hauser W. Efficacy, acceptability and safety of cognitive behavioural therapies in fibromyalgia syndrome - A systematic review and meta-analysis of randomized controlled trials. *European Journal of Pain (United Kingdom)* 2018;**22**:242-60

Berner M, Gunzler C. Efficacy of Psychosocial Interventions in Men and Women with Sexual Dysfunctions-A Systematic Review of Controlled Clinical Trials: Part 1-The efficacy of psychosocial interventions for male sexual dysfunction Berner and Gunzler Psychosocial Interventions in Male Sexual Dysfunction. *Journal of Sexual Medicine* 2012;**9**:3089-107

Berryhill MB, Halli-Tierney A, Culmer N, Williams N, Betancourt A, King M*, et al.* Videoconferencing psychological therapy and anxiety: a systematic review. *Family Practice* 2018;**04**:04

Bethel NJ. *Self-applied interventions for social anxiety*: University of Sheffield; 2010.

Beumont P, Hay P, Beumont D, Birmingham L, Derham H, Jordan A*, et al.* Australian and New Zealand clinical practice guidelines for the treatment of anorexia nervosa.[Erratum appears in Aust N Z J Psychiatry. 2004 Nov-Dec;38(11-12):987]. *Australian & New Zealand Journal of Psychiatry* 2004;**38**:659-70

Bhatia U, Nadkarni A, Murthy P, Rao R, Crome I. Recent advances in treatment for older people with substance use problems: An updated systematic and narrative review. *European Geriatric Medicine* 2015;**6**:580-6

Biajar A, Mollayeva T, Sokoloff S, Colantonio A. Assistive technology to enable sleep function in patients with acquired brain injury: Issues and opportunities. *British Journal of Occupational Therapy* 2017;**80**:225-49

Binnie J, Blainey S. The use of cognitive behavioural therapy for adults with autism spectrum disorders: A review of the evidence. *Mental Health Review Journal* 2013;**18**:93-104

Birur B, Moore NC, Davis LL. An Evidence-Based Review of Early Intervention and Prevention of Posttraumatic Stress Disorder. *Community Mental Health Journal* 2017;**53**:183-201

Blake MJ, Sheeber LB, Youssef GJ, Raniti MB, Allen NB. Systematic Review and Meta-analysis of Adolescent Cognitive-Behavioral Sleep Interventions. *Clinical Child & Family Psychology Review* 2017;**20**:227-49

Bodde N, Brooks J, Baker G, Boon P, Hendriksen J, Mulder O*, et al.* Psychogenic non-epileptic seizures-Definition, etiology, treatment and prognostic issues: A critical review. *Seizure* 2009;**18**:543-53

Bogdanov S, Naismith S, Lah S. Sleep outcomes following sleep-hygiene-related interventions for individuals with traumatic brain injury: A systematic review. *Brain Injury* 2017;**31**:422-33

Bomasang-Layno E, Fadlon I, Murray AN, Himelhoch S. Antidepressive treatments for Parkinson's disease: A systematic review and meta-analysis. *Parkinsonism and Related Disorders* 2015;**21**:833-42

Bomasang-Layno E, Fadlon I, Murray AN, Himelhoch S. Antidepressive treatments for Parkinson's disease: A systematic review and meta-analysis. *American Journal of Geriatric Psychiatry* 2016;**24 (3 Supple 1)**:S102-S3

Bouman TK. Psychological treatments for hypochondriasis: A narrative review. *Current Psychiatry Reviews* 2014;**10**:58-69

Bourke L, Boorjian SA, Briganti A, Klotz L, Mucci L, Resnick MJ*, et al.* Survivorship and Improving Quality of Life in Men with Prostate Cancer. *European Urology* 2015;**68**:374-83

Braun SR, Gregor B, Tran US. Comparing Bona Fide Psychotherapies of Depression in Adults with Two Meta-Analytical Approaches. *Plos One* 2013;**8(6)**:e68135

Brendel KE. A systematic review and meta-analysis of the effectiveness of child-parent interventions for children and adolescents with anxiety disorders. *Dissertation Abstracts International Section A: Humanities and Social Sciences* 2012;**72**:2965

Brent M, Lobato D, Leleiko N. Psychological treatments for pediatric functional gastrointestinal disorders. *Journal of Pediatric Gastroenterology and Nutrition* 2009;**48**:13-21

Britton EP. *A meta-analysis of group cognitive behavior therapy for children and adolescents with social phobia*: The Wright Institute; 2007.

Brooks AT, Wallen GR. Sleep disturbances in individuals with alcohol-related disorders: a review of cognitive-behavioral therapy for insomnia (CBT-I) and associated non-pharmacological therapies. *Subst Abuse* 2014;**(8)**:55-62

Brown R, Witt A, Fegert J, Keller F, Rassenhofer M, Plener P. Psychosocial interventions for children and adolescents after man-made and natural disasters: A meta-analysis and systematic review. *Psychological Medicine* 2017;**47**:1893-905

Browne C, Smith IC. Psychological interventions for anger and aggression in people with intellectual disabilities in forensic services. *Aggression and Violent Behavior* 2018;**39**:1-14

Brownley KA, Berkman ND, Sedway JA, Lohr KN, Bulik CM. Binge eating disorder treatment: a systematic review of randomized controlled trials. *International Journal of Eating Disorders* 2007;**40**:337-48

Brunwasser SM, Gillham JE, Kim ES. A Meta-Analytic Review of the Penn Resiliency Program's Effect on Depressive Symptoms. *Journal of Consulting and Clinical Psychology* 2009;**77**:1042-54

Buhrman M, Gordh T, Andersson G. Internet interventions for chronic pain including headache: A systematic review. *Internet Interventions* 2016;**Part 1. 4**:17-34

Bulik CM, Berkman ND, Brownley KA, Sedway JA, Lohr KN. Anorexia nervosa treatment: a systematic review of randomized controlled trials. *International Journal of Eating Disorders* 2007;**40**:310-20

Burghardt S, Koranyi S, Magnucki G, Strauss B, Rosendahl J. Non-pharmacological interventions for reducing mental distress in patients undergoing dental procedures: Systematic review and meta-analysis. *Journal of Dentistry* 2018;**69**:22-31

Burin AB, Osorio FL. Interventions for music performance anxiety: Results from a systematic literature review. *Revista de Psiquiatria Clinica* 2016;**43**:116-31

Cairns R, Hotopf M. A systematic review describing the prognosis of chronic fatigue syndrome. *Occupational Medicine* 2005;**55**:20-31

Calati R, Pedrini L, Alighieri S, Alvarez MI, Desideri L, Durante D*, et al.* Is cognitive behavioural therapy an effective complement to antidepressants in adolescents? A meta-analysis. *Acta Neuropsychiatr* 2011;**23(6)**:263-71

Calear AL, Christensen H. Systematic review of school-based prevention and early intervention programs for depression. *Journal of Adolescence* 2009;**33**:429-38

Calear AL, Christensen H. Review of internet-based prevention and treatment programs for anxiety and depression in children and adolescents. *Medical Journal of Australia* 2010;**192**:S12-S4

Cameron SK, Rodgers J, Dagnan D. The relationship between the therapeutic alliance and clinical outcomes in cognitive behaviour therapy for adults with depression: A meta-analytic review. *Clinical psychology & psychotherapy* 2018;**26**:26

Campbell CL, Campbell LC, Campbell CL, Campbell LC. A systematic review of cognitive behavioral interventions in advanced cancer. *Patient Education & Counseling* 2012;**89**:15-24

Carnevale TD. Universal Adolescent Depression Prevention Programs: A Review. *Journal of School Nursing* 2013;**29**:181-95

Carolan S, Harris PR, Cavanagh K. Improving Employee Well-Being and Effectiveness: Systematic Review and Meta-Analysis of Web-Based Psychological Interventions Delivered in the Workplace. *Journal of Medical Internet Research* 2017;**19**:e271

Carrico AW, Zepf R, Meanley S, Batchelder A, Stall R. Critical Review: When the Party is Over: A Systematic Review of Behavioral Interventions for Substance-Using Men Who Have Sex with Men. *Journal of Acquired Immune Deficiency Syndromes* 2016;**73**:299-306

Chaiyachati KH, Ogbuoji O, Price M, Suthar AB, Negussie EK, Barnighausen T. Interventions to improve adherence to antiretroviral therapy: A rapid systematic review. *Aids* 2014;**28**:S187-S204

Chambers CT, Taddio A, Uman LS, McMurtry CM. Psychological interventions for reducing pain and distress during routine childhood immunizations: a systematic review. *Clinical Therapeutics* 2009;**31(S2)**:S77-S103

Chambers D, Bagnall AM, Hempel S, Forbes C. Interventions for the treatment, management and rehabilitation of patients with chronic fatigue syndrome/myalgic encephalomyelitis: An updated systematic review. *Journal of the Royal Society of Medicine* 2006;**99**:506-20

Chambers SK, Hyde MK, Smith DP, Hughes S, Yuill S, Egger S*, et al.* New Challenges in Psycho-Oncology Research III: A systematic review of psychological interventions for prostate cancer survivors and their partners: Clinical and research implications. *Psycho-Oncology* 2017;**26**:873-913

Chan EK-H. *Efficacy of cognitive-behavioral, pharmacological, and combined treatments of depression: A meta-analysis*: University of Calgary (Canada); 2006.

Chandler ML. Psychotherapy for adult attention deficit/hyperactivity disorder: a comparison with cognitive behaviour therapy. *Journal of Psychiatric & Mental Health Nursing* 2013;**20**:814-20

Chapman A, Liu S, Merkouris S, Enticott JC, Yang H, Browning CJ*, et al.* Psychological Interventions for the Management of Glycemic and Psychological Outcomes of Type 2 Diabetes Mellitus in China: A Systematic Review and Meta-Analyses of Randomized Controlled Trials. *Frontiers in Public Health* 2015;**3**:252

Chatters R, Cooper K, Day E, Knight M, Lagundoye O, Wong R*, et al.* Psychological and psychosocial interventions for cannabis cessation in adults: A systematic review. *Addiction Research & Theory* 2016;**24**:93-110

Chen L, Zhang G, Hu M, Liang X. Eye movement desensitization and reprocessing versus cognitive-behavioral therapy for adult posttraumatic stress disorder: systematic review and meta-analysis. *Journal of Nervous & Mental Disease* 2015;**203**:443-51

Chiang KJ, Tsai JC, Liu D, Lin CH, Chiu HL, Chou KR. Efficacy of cognitive-behavioral therapy in patients with bipolar disorder: A metaanalysis of randomized controlled trials. *Plos One* 2017;**12(5)**:e0176849

Chien CH, Liu KL, Chien HT, Liu HE. The effects of psychosocial strategies on anxiety and depression of patients diagnosed with prostate cancer: A systematic review. *International Journal of Nursing Studies* 2014;**51**:28-38

Christensen H, Pallister E, Smale S, Hickie IB, Calear AL. Community-based prevention programs for anxiety and depression in youth: a systematic review *Journal of Primary Prevention* 2010;**31**:139-70

Chu BC, Harrison TL. Disorder-specific effects of CBT for anxious and depressed youth: a meta-analysis of candidate mediators of change. *Clinical Child & Family Psychology Review* 2007;**10**:352-72

Chun J, Shim H, Kim S. A Meta-Analysis of Treatment Interventions for Internet Addiction Among Korean Adolescents. *Cyberpsychology, behavior and social networking* 2017;**20**:225-31

Churchill R, Hunot V, Corney R, Knapp M, McGuire H, Tylee A*, et al.* A systematic review of controlled trials of the effectiveness and cost-effectiveness of brief psychological treatments for depression. *Health Technology Assessment* 2001;**5**:1-173

Ciketic S, Hayatbakhsh MR, Doran CM, Najman JM, McKetin R. A review of psychological and pharmacological treatment options for methamphetamine dependence. *Journal of Substance Use* 2012;**17**:363-83

Cima RFF, Andersson G, Schmidt CJ, Henry JA. Cognitive-Behavioral Treatments for Tinnitus: A Review of the Literature. *Journal of the American Academy of Audiology* 2014;**25**:29-61

Clarke K, Mayo-Wilson E, Kenny J, Pilling S. Can non-pharmacological interventions prevent relapse in adults who have recovered from depression? A systematic review and meta-analysis of randomised controlled trials. *Clinical Psychology Review* 2015;**39**:58-70

Cleary M, Hunt GE, Matheson S, Walter G. Psychosocial treatments for people with co-occurring severe mental illness and substance misuse: systematic review. *Journal of Advanced Nursing* 2009;**65**:238-58

Clery P, Stahl D, Ismail K, Treasure J, Kan C. Systematic review and meta-analysis of the efficacy of interventions for people with Type 1 diabetes mellitus and disordered eating. *Diabetic Medicine* 2017;**34**:1667-75

Cobb B, Sample PL, Alwell M, Johns NR. Cognitive-Behavioral Interventions, Dropout, and Youth With Disabilities: A Systematic Review. *Remedial and Special Education* 2006;**27**:259-75

Cobeanu O, David D. Alleviation of Side Effects and Distress in Breast Cancer Patients by Cognitive-Behavioral Interventions: A Systematic Review and Meta-analysis. *Journal of Clinical Psychology in Medical Settings* 2018;**25(4)**:335-55

Cohan SL, Chavira DA, Stein MB. Practitioner Review: Psychosocial interventions for children with selective mutism: A critical evaluation of the literature from 1990-2005. *Journal of Child Psychology and Psychiatry and Allied Disciplines* 2006;**47**:1085-97

Cohen G, Harvey J. The use of psychological interventions for adult male sex offenders with a learning disability: A systematic review. *Journal of Sexual Aggression* 2016;**22**:206-23

Collado A, Lim AC, MacPherson L. A systematic review of depression psychotherapies among Latinos. *Clinical Psychology Review* 2016;**45**:193-209

Comer JS, Hong N, Poznanski B, Silva K, Wilson M. Evidence Base Update on the Treatment of Early Childhood Anxiety and Related Problems. *Journal of Clinical Child & Adolescent Psychology* 2019;**48(1)**:1-15

Compton SN, March JS, Brent D, Albano AM, Weersing VR, Curry J. Cognitive-behavioral psychotherapy for anxiety and depressive disorders in children and adolescents: an evidence-based medicine review. *Journal of the American Academy of Child and Adolescent Psychiatry* 2004;**43**:930-59

Conejo-Ceron S, Moreno-Peral P, Rodriguez-Morejon A, Motrico E, Navas-Campana D, Rigabert A*, et al.* Effectiveness of Psychological and Educational Interventions to Prevent Depression in Primary Care: A Systematic Review and Meta-Analysis. *Annals of Family Medicine* 2017;**15**:262-71

Cong X, Perry M, Bernier KM, Young EE, Starkweather A. Effects of Self-Management Interventions in Patients With Irritable Bowel Syndrome: Systematic Review. *Western Journal of Nursing Research* 2017;**40(11)**:1698-720

Cooper C, Balamurali TB, Selwood A, Livingston G. A systematic review of intervention studies about anxiety in caregivers of people with dementia. *International Journal of Geriatric Psychiatry* 2007;**22**:181-8

Cooper K, Chatters R, Kaltenthaler E, Wong R. Psychological and psychosocial interventions for cannabis cessation in adults: a systematic review short report. *Health Technology Assessment* 2015;**19**:1-130

Coss-Adame E, Erdogan A, Rao SS. Treatment of esophageal (noncardiac) chest pain: an expert review. *Clinical Gastroenterology & Hepatology* 2014;**12**:1224-45

Cossu G, Cantone E, Pintus M, Cadoni M, Pisano A, Otten R*, et al.* Integrating children with psychiatric disorders in the classroom: A systematic review. *Clinical Practice and Epidemiology in Mental Health* 2015;**11**:41-57

Costa MB, Melnik T. Effectiveness of psychosocial interventions in eating disorders: an overview of Cochrane systematic reviews. *Einstein (Sao Paulo, Brazil)* 2016;**14**:235-77

Coull G, Morris PG. The clinical effectiveness of CBT-based guided self-help interventions for anxiety and depressive disorders: a systematic review. *Psychological Medicine* 2011;**41**:2239-52

Cowlishaw S, Merkouris S, Dowling N, Anderson C, Jackson A, Thomas S. Psychological therapies for pathological and problem gambling. *Cochrane Database Syst Rev* 2012;**(11)**:CD008937

Crawford C, Wallerstedt DB, Khorsan R, Clausen SS, Jonas WB, Walter JAG. A systematic review of biopsychosocial training programs for the self-management of emotional stress: Potential applications for the military. *Evidence-based Complementary and Alternative Medicine* 2013;**2013**:747694

Creswell C, Cartwright-Hatton S. Family treatment of child anxiety: outcomes, limitations and future directions. *Clinical Child & Family Psychology Review* 2007;**10**:232-52

Crumlish N, O'Rourke K. A systematic review of treatments for post-traumatic stress disorder among refugees and asylum-seekers. *Journal of Nervous & Mental Disease* 2010;**198**:237-51

Cruz CG. *Integrative review and development of algorithm for the treatment of insomnia in the geriatric population*: New Mexico State University; 2016.

Cuijpers P. A psychoeducational approach to the treatment of depression: A meta- analysis of Lewinsohn's 'Coping With Depression' course. *Behavior Therapy* 1998;**29**:521-33

Cuijpers P. Psychological outreach programmes for the depressed elderly: a meta-analysis of effects and dropout. *International Journal of Geriatric Psychiatry* 1998;**13**:41-8

Cuijpers P, Ebert DD, Acarturk C, Andersson G, Cristea IA. Personalized Psychotherapy for Adult Depression: A Meta-Analytic Review. *Behavior Therapy* 2016;**47**:966-80

Cuijpers P, Gentili C, Banos RM, Garcia-Campayo J, Botella C, Cristea IA. Relative effects of cognitive and behavioral therapies on generalized anxiety disorder, social anxiety disorder and panic disorder: A meta-analysis. *Journal of Anxiety Disorders* 2016;**43**:79-89

Cuijpers P, Karyotaki E, Pot AM, Park M, Reynolds CF. Managing depression in older age: Psychological interventions. *Maturitas* 2014;**79**:160-9

Cuijpers P, van Straten A, Andersson G, van Oppen P. Psychotherapy for Depression in Adults: A Meta-Analysis of Comparative Outcome Studies. *Journal of Consulting and Clinical Psychology* 2008;**76**:909-22

Cuijpers P, van Straten A, Smit F. Psychological treatment of late-life depression: a meta-analysis of randomized controlled trials. *International Journal of Geriatric Psychiatry* 2006;**21**:1139-49

Cullen KL, Irvin A, Collie F. Effectiveness of Workplace Interventions in Return-to-Work for Musculoskeletal, Pain-Related and Mental Health Conditions An Update of the Evidence and Messages for Practitioners. *Orthopaedic Physical Therapy Practice* 2018;**30**:179

Cunningham JEA, Shapiro CM. Cognitive Behavioural Therapy for Insomnia (CBT-I) to treat depression: A systematic review. *Journal of Psychosomatic Research* 2018;**106**:1-12

Da Costa RT, Sardinha A, Nardi AE. Virtual reality exposure in the treatment of fear of flying. *Aviation, Space, and Environmental Medicine* 2008;**79**:899-903

da Silva Roggi PM, da Gama MFN, Neves FS, Garcia F. Update on treatment of craving in patients with addiction using cognitive behavioral therapy. *Clinical Neuropsychiatry* 2015;**12**:118-27

Dagnan D, Jackson I, Eastlake L. A systematic review of cognitive behavioural therapy for anxiety in adults with intellectual disabilities. *Journal of Intellectual Disability Research* 2018;**62**:974-91

Dale HL, Adair PM, Humphris GM. Systematic review of post-treatment psychosocial and behaviour change interventions for men with cancer. *Psycho-Oncology* 2010;**19**:227-37

David AR, Szamoskozi S. A meta-analytical study on the effects of cognitive behavioral techniques for reducing distress in organizations. *Journal of Cognitive and Behavioral Psychotherapies* 2011;**11**:221-36

David D, Cotet C, Matu S, Mogoase C, Stefan S. 50 years of rational-emotive and cognitive-behavioral therapy: A systematic review and meta-analysis. *Journal of clinical psychology* 2018;**74**:304-18

Davies C, Cipriani A, Ioannidis JPA, Radua J, Stahl D, Provenzani U*, et al.* Lack of evidence to favor specific preventive interventions in psychosis: a network meta-analysis. *World Psychiatry* 2018;**17**:196-209

de Arellano MA, Lyman D, Jobe-Shields L, George P, Dougherty RH, Daniels AS*, et al.* Trauma-focused cognitive-behavioral therapy for children and adolescents: Assessing the evidence. *Psychiatric Services* 2014;**65**:591-602

de Bitencourt Machado D, Braga Laskoski P, Trelles Severo C, Margareth Bassols A, Sfoggia A, Kowacs C*, et al.* A Psychodynamic Perspective on a Systematic Review of Online Psychotherapy for Adults. *British Journal of Psychotherapy* 2016;**32**:79-108

De Swart JJW, Van den Broek H, Stams GJJM, Asscher JJ, Van der Laan PH, Holsbrink-Engels GA*, et al.* The effectiveness of institutional youth care over the past three decades: A meta-analysis. *Children & Youth Services Review* 2012;**34**:1818-24

Deady M, Choi I, Calvo RA, Glozier N, Christensen H, Harvey SB. eHealth interventions for the prevention of depression and anxiety in the general population: A systematic review and meta-analysis. *BMC Psychiatry* 2017;**17(1)**:310

Deas G, Kelly C, Hadjinicolaou AV, Holt C, Agius M, Zaman R. An update on: Meta-analysis of medical and non-medicaltreatments of the prodromal phase of Psychotic Illness in at risk Mental States. *Psychiatria Danubina* 2016;**28**:31-8

De-Bacco C, Marzola E, Fassino S, Abbate-Daga G. Psychodynamic psychotherapies for feeding and eating disorders. *Minerva Psichiatrica* 2017;**58**:162-80

Deenadayalan Y, Perraton L, Machotka Z, Kumar S. Day therapy programs for adolescents with mental health problems: a systematic review. *Internet Journal of Allied Health Sciences and Practice* 2010;**8**:1-14

Denis C, Lavie E, Fatseas M, Auriacombe M. Psychotherapeutic interventions for cannabis abuse and/or dependence in outpatient settings. *Cochrane Database Syst Rev* 2006;**3**:CD005336

Dennis CL. Treatment of postpartum depression, part 2: a critical review of nonbiological interventions. *The Journal of clinical psychiatry* 2004;**65**:1252-65

Devoe DJ, Peterson A, Addington J. Negative Symptom Interventions in Youth at Risk of Psychosis: A Systematic Review and Network Meta-analysis. *Schizophrenia Bulletin* 2017;**24**:24

Di Giulio G. *Therapist, client factors, and efficacy in cognitive behavioural therapy: A meta-analytic exploration of factors that contribute to positive outcome*: University of Ottowa (Canada); 2007.

Dickerson SS, Connors LM, Fayad A, Dean GE. Sleep-wake disturbances in cancer patients: narrative review of literature focusing on improving quality of life outcomes. *Nature & Science of Sleep* 2014;**6**:85-100

Doki S, Sasahara S, Matsuzaki I. Psychological approach of occupational health service to sick leave due to mental problems: a systematic review and meta-analysis. *International archives of occupational and environmental health* 2015;**88**:659-67

Dopp AR, Borduin CM, Brown CE. Evidence-based treatments for juvenile sexual offenders: Review and recommendations. *Journal of Aggression, Conflict and Peace Research* 2015;**7**:223-36

Dorsey S, McLaughlin KA, Kerns SE, Harrison JP, Lambert HK, Briggs EC*, et al.* Evidence base update for psychosocial treatments for children and adolescents exposed to traumatic events. *Journal of Clinical Child and Adolescent Psychology* 2017;**46**:303-30

Dowell KA, Ogles BM. The effects of parent participation on child psychotherapy outcome: A meta-analytic review. *Journal of Clinical Child and Adolescent Psychology* 2010;**39**:151-62

Dowling N, Merkouris S, Lorains F. Interventions for comorbid problem gambling and psychiatric disorders: Advancing a developing field of research. *Addictive Behaviors* 2016;**58**:21-30

Dray J, Bowman J, Campbell E, Freund M, Wolfenden L, Hodder RK*, et al.* Systematic Review of Universal Resilience-Focused Interventions Targeting Child and Adolescent Mental Health in the School Setting. *Journal of the American Academy of Child & Adolescent Psychiatry* 2017;**56**:813-24

Duncan EAS, Nicol MM, Ager A, Dalgleish L. A systematic review of structured group interventions with mentally disordered offenders. *Criminal Behaviour and Mental Health* 2006;**16**:217-41

Dutra L, Stathopoulou G, Basden SL, Leyro TM, Powers MB, Otto MW. A meta-analytic review of psychosocial interventions for substance use disorders. *American Journal of Psychiatry* 2008;**165**:179-87

Easthall C, Song F, Bhattacharya D. A meta-analysis of cognitive-based behaviour change techniques as interventions to improve medication adherence. *BMJ Open* 2013;**3(8)**:e002749

Eccleston C, Fisher E, Craig L, Duggan GB, Rosser BA, Keogh E. Psychological therapies (Internet-delivered) for the management of chronic pain in adults. *Cochrane Database Syst Rev* 2014;**26(2)**:CD010152

Eccleston C, Morley S, Williams A, Yorke L, Mastroyannopoulou K. Systematic review of randomised controlled trials of psychological therapy for chronic pain in children and adolescents, with a subset meta-analysis of pain relief. *Pain* 2002;**99**:157-65

Edwards AR. *Psychotherapy and pharmacotherapy for social anxiety disorder: A comprehensive meta-analysis*: Temple University; 2011.

Ekers D. *Behavioural activation for depression: a systematic review and controlled clinical trial*: University of York; 2011.

Ekers D, Richards D, Gilbody S. A meta-analysis of randomized trials of behavioural treatment of depression. *Psychological Medicine* 2008;**38**:611-23

Escobar KM, Gorey KM. Cognitive-behavioral interventions for anxiety disorders: Rapid review suggestion of larger effects among Hispanic than non-Hispanic White people. *Journal of Social Service Research* 2018;**44**:132-40

Faizi F, Tavallaee A, Rahimi A, Saburi A, Saghafinia M. Quality assessment of randomized control trials applied psychotherapy for chronic pains in Iran: A systematic review of domestic trials. *Iranian Red Crescent Medical Journal* 2014;**16(9)**:e15312

Farhood Z, Ong AA, Discolo CM. PANDAS: A systematic review of treatment options. *International Journal of Pediatric Otorhinolaryngology* 2016;**89**:149-53

Farronato NS, Dursteler-MacFarland KM, Wiesbeck GA, Petitjean SA. A systematic review comparing cognitive-behavioral therapy and contingency management for cocaine dependence. *Journal of Addictive Diseases* 2013;**32**:274-87

Farver-Vestergaard I, Jacobsen D, Zachariae R. Efficacy of psychosocial interventions on psychological and physical health outcomes in chronic obstructive pulmonary disease: A systematic review and meta-analysis. *Psychotherapy and Psychosomatics* 2015;**84**:37-50

Ferriter M, Kaltenthaler E, Parry G, Beverley C. Computerised cognitive behaviour therapy for phobias and panic disorder: a systematic review. *Journal of Public Mental Health* 2008;**7**:15-22

Feuerstein M, Burrell LM, Miller VI, Lincoln A, Huang GD, Berger R. Clinical management of carpal tunnel syndrome: A 12-year review of outcomes. *American Journal of Industrial Medicine* 1999;**35**:232-45

Firth J, Torous J, Nicholas J, Carney R, Pratap A, Rosenbaum S*, et al.* The efficacy of smartphone-based mental health interventions for depressive symptoms: a meta-analysis of randomized controlled trials. *World Psychiatry* 2017;**16**:287-98

Fisher PL. The Efficacy of Psychological Treatments for Generalised Anxiety Disorder? In: Davey GCL, Wells A, editors. *Worry and its Psychological Disorders: Theory, Assessment and Treatment.* Hoboken, NJ: Wiley Publishing; US; 2006:359-77.

Flik CE, Bakker L, Laan W, van Rood YR, Smout AJ, de Wit NJ. Systematic review: The placebo effect of psychological interventions in the treatment of irritable bowel syndrome. *World Journal of Gastroenterology* 2017;**23**:2223-33

Ford AC, Vandvik PO. Irritable bowel syndrome. *BMJ clinical evidence* 2010:pii: 0410

Ford AC, Vandvik PO. Irritable bowel syndrome. *BMJ clinical evidence* 2012:pii: 0410

Forte AL, Hill M, Pazder R, Feudtner C. Bereavement care interventions: A systematic review. *BMC Palliative Care* 2004;**3**:1-14

Fossum S, Handegard BH, Adolfsen F, Vis SA, Wynn R. A meta-analysis of long-term outpatient treatment effects for children and adolescents with conduct problems. *Journal of Child and Family Studies* 2016;**25**:15-29

Frechette-Simard C, Plante I, Bluteau J. Strategies included in cognitive behavioral therapy programs to treat internalized disorders: a systematic review. *Cognitive Behaviour Therapy* 2017;**47(4)**:263-85

Frederiksen Y, Farver-Vestergaard I, Skovgard NG, Ingerslev HJ, Zachariae R. Efficacy of psychosocial interventions for psychological and pregnancy outcomes in infertile women and men: A systematic review and meta-analysis. *BMJ Open* 2015;**5(1)**:e006592

Fredette C, El-Baalbaki G, Palardy V, Rizkallah E, Guay S. Social support and cognitive-behavioral therapy for posttraumatic stress disorder: A systematic review. *Traumatology* 2016;**22**:131-44

Freeman JB, Choate-Summers ML, Moore PS, Garcia AM, Sapyta JJ, Leonard HL*, et al.* Cognitive behavioral treatment for young children with obsessive-compulsive disorder. *Biological Psychiatry* 2007;**61**:337-43

French L, Turner K, Dawson S, Moran P. Psychological treatment of depression and anxiety in patients with co-morbid personality disorder: A scoping study of trial evidence. *Personality and Mental Health* 2017;**11**:101-17

Friedrich A, Schlarb AA. Let's talk about sleep: a systematic review of psychological interventions to improve sleep in college students. *Journal of Sleep Research* 2018;**27**:4-22

Fristad MA, MacPherson HA. Evidence-based psychosocial treatments for child and adolescent bipolar spectrum disorders. *Journal of Clinical Child and Adolescent Psychology* 2014;**43**:339-55

Furukawa TA, Watanabe N, Churchill R. Combined psychotherapy plus antidepressants for panic disorder with or without agoraphobia. *Cochrane Database Syst Rev* 2007;**24(1)**:CD004364

Galdston MR, John RM. Mind over gut: Psychosocial management of pediatric functional abdominal pain. *Journal of Pediatric Health Care* 2016;**30**:535-45

Gale CK, Millichamp J. Generalised anxiety disorder. *BMJ clinical evidence* 2011:pii: 1002

Gallagher-Thompson D, Coon DW. Evidence-based psychological treatments for distress in family caregivers of older adults. *Psychology and Aging* 2007;**22**:37-51

Galvao-de Almeida A, Araujo Filho GM, Berberian Ade A, Trezsniak C, Nery-Fernandes F, Araujo Neto CA*, et al.* The impacts of cognitive-behavioral therapy on the treatment of phobic disorders measured by functional neuroimaging techniques: a systematic review. *Braz J Psychiatry* 2013;**35(3)**:279-83

Gandy M, Sharpe L, Perry KN. Cognitive behavior therapy for depression in people with epilepsy: a systematic review. *Epilepsia* 2013;**54**:1725-34

Garland SN, Johnson JA, Savard J, Gehrman P, Perlis M, Carlson L*, et al.* Sleeping well with cancer: A systematic review of cognitive behavioral therapy for insomnia in cancer patients. *Neuropsychiatric Disease and Treatment* 2014;**10**:1113-23

Gartlehner G, Forneris CA, Brownley KA, Gaynes BN, Sonis J, Coker-Schwimmer E*, et al. Interventions for the Prevention of Posttraumatic Stress Disorder (PTSD) in Adults After Exposure to Psychological Trauma*. Rockville (US): Agency for Healthcare Research and Quality; 2013.

Gaudiano BA. Is symptomatic improvement in clinical trials of cognitive-behavioral therapy for psychosis clinically significant? *Journal of Psychiatric Practice* 2006;**12**:11-23

Gaudiano BA, Weinstock LM, Miller IW. Improving treatment adherence in bipolar disorder: a review of current psychosocial treatment efficacy and recommendations for future treatment development. *Behavior Modification* 2008;**32**:267-301

Gaynor D, Cock H, Agrawal N. Psychological treatments for functional non-epileptic attacks: A systematic review. *Acta Neuropsychiatrica* 2009;**21**:158-68

Gearing RE, Schwalbe CSJ, Lee R, Hoagwood KE. The effectiveness of booster sessions in CBT treatment for child and adolescent mood and anxiety disorders. *Depression and Anxiety* 2013;**30**:800-8

Geiger-Brown JM, Rogers VE, Liu W, Ludeman EM, Downton KD, Diaz-Abad M. Cognitive behavioral therapy in persons with comorbid insomnia: A meta-analysis. *Sleep Medicine Reviews* 2015;**23**:54-67

George N, Abdallah J, Maradey-Romero C, Gerson L, Fass R. Review article: The current treatment of non-cardiac chest pain. *Alimentary Pharmacology and Therapeutics* 2016;**43**:213-39

Gibby BA, Casline EP, Ginsburg GS. Long-term outcomes of youth treated for an anxiety disorder: A critical review. *Clinical Child and Family Psychology Review* 2017;**20**:201-25

Gillen PA, Sinclair M, Kernohan WG, Begley CM, Luyben AG. Interventions for prevention of bullying in the workplace. *Cochrane Database Syst Rev* 2017;**(1)**:CD009778

Giroux I, Goulet A, Mercier J, Jacques C, Bouchard S. Online and Mobile Interventions for Problem Gambling, Alcohol, and Drugs: A Systematic Review. *Frontiers in Psychology* 2017;**8**:954

Glenn CR, Franklin JC, Nock MK. Evidence-based psychosocial treatments for self-injurious thoughts and behaviors in youth. *Journal of clinical child and adolescent psychology : the official journal for the Society of Clinical Child and Adolescent Psychology, American Psychological Association, Division 53* 2015;**44**:1-29

Glombiewski JA, Sawyer AT, Gutermann J, Koenig K, Rief W, Hofmann SG. Psychological treatments for fibromyalgia: A meta-analysis. *Pain* 2010;**151**:280-95

Goldberg SB, Buck B, Raphaely S, Fortney JC. Measuring Psychiatric Symptoms Remotely: a Systematic Review of Remote Measurement-Based Care. *Current Psychiatry Reports* 2018;**20**:81

Gomes HS, Viana KA, Batista AC, Costa LR, Hosey MT, Newton T. Cognitive behaviour therapy for anxious paediatric dental patients: a systematic review. *International Journal of Paediatric Dentistry* 2018;**08**:08

Goncalves R, Lages AC, Rodrigues H, Pedrozo AL, Coutinho ESF, Neylan T*, et al.* Potential biomarkers of cognitive behavior-therapy for post-traumatic stress disorder: A systematic review *Revista de Psiquiatria Clinica* 2011;**38**:155-60

Goncalves R, Pedrozo AL, Coutinho ESF, Figueira I, Ventura P. Efficacy of Virtual Reality Exposure Therapy in the Treatment of PTSD: A Systematic Review. *Plos One* 2012;**7(12)**:e48469

Gonzalez-Pinto A, Gonzalez C, Enjuto S, de Corres B, Lopez P, Palomo J*, et al.* Psychoeducation-and cognitive-behavioral therapy in bipolar disorder: An update. *Acta Psychiatrica Scandinavica* 2004;**109**:83-90

Gooding P, Tarrier N. A systematic review and meta-analysis of cognitive-behavioural interventions to reduce problem gambling: hedging our bets? *Behaviour Research and Therapy* 2009;**47**:592-607

Goodyer IM, Wilkinson PO. Practitioner Review: Therapeutics of unipolar major depressions in adolescents. *Journal of Child Psychology & Psychiatry & Allied Disciplines* 2018;**25**:25

Gordon D, Heimberg RG, Tellez M, Ismail AI. A critical review of approaches to the treatment of dental anxiety in adults. *Journal of Anxiety Disorders* 2013;**27**:365-78

Gorin SS, Badr H, Jacobsen PB, Janke EA, Jim HS, Krebs P. Meta-analysis of behavioral interventions to reduce cancer pain. *Journal of Clinical Oncology* 2011;**29**:S1

Goslin RE, Gray RN, McCrory DC, Penzien D, Rains J, Hasselblad V. *Behavioural and Physical Treatments for Migraine Headache*. Rockville (US): Agency for Health Care Policy and Research; 1999.

Gottdiener WH. *The benefits of individual psychotherapy for schizophrenic patients: A meta-analytic review of the psychotherapy outcome literature*: New School for Social Research; 2000.

Gottdiener WH, Haslam N. The benefits of individual psychotherapy for people diagnosed with schizophrenia: A meta-analytic review. *Ethical Human Sciences and Services* 2002;**4**:163-87

Gould RA, Buckminster S, Pollack MH, Otto MW, Yap L. Cognitive-behavioral and pharmacological treatment for social phobia: A meta-analysis. *Clinical Psychology: Science and Practice* 1997;**4**:291-306

Gould RA, Otto MW, Pollack MH. A meta-analysis of treatment outcome for panic disorder. *Clinical Psychology Review* 1995;**15**:819-44

Gould RA, Otto MW, Pollack MH, Yap L. Cognitive behavioral and pharmacological treatment of generalized anxiety disorder: A preliminary meta-analysis. *Behavior Therapy* 1997;**28**:285-305

Goulding L, Furze G, Birks Y. Randomized controlled trials of interventions to change maladaptive illness beliefs in people with coronary heart disease: systematic review *Journal of Advanced Nursing* 2010;**66**:946-61

Gregory B, Peters L. Changes in the self during cognitive behavioural therapy for social anxiety disorder: A systematic review. *Clinical Psychology Review* 2017;**52**:1-18

Gregory Jr VL. Cognitive-behavioral therapy for comorbid bipolar and substance use disorders: A systematic review of controlled trials. *Mental Health and Substance Use: Dual Diagnosis* 2011;**4**:302-13

Gregory VL, Jr. Cognitive-behavioral therapy for bipolar disorder: Implications for clinical social workers. *Journal of Social Service Research* 2010;**36**:460-9

Gregory VL, Jr. Cognitive-behavioral therapy for depressive symptoms in persons of African descent: A meta-analysis. *Journal of Social Service Research* 2016;**42**:113-29

Groff SE. Is enhanced cognitive behavioral therapy an effective intervention in eating disorders? A review. *Journal of evidence-informed social work* 2015;**12**:272-88

Guggisberg KW. *Methodological review and meta-analysis of treatments for child and adolescent obsessive-compulsive disorder*: The University of Utah; 2005.

Gunzler C, Berner MM. Efficacy of psychosocial interventions in men and women with sexual dysfunctions: a systematic review of controlled clinical trials: part 2--The efficacy of psychosocial interventions for female sexual dysfunction. *Journal of Sexual Medicine* 2012;**9**:3108-25

Gutermann J, Schreiber F, Matulis S, Schwartzkopff L, Deppe J, Steil R. Psychological Treatments for Symptoms of Posttraumatic Stress Disorder in Children, Adolescents, and Young Adults: A Meta-Analysis. *Clinical Child & Family Psychology Review* 2016;**19**:77-93

Hans E, Hiller W. Effectiveness of and dropout from outpatient cognitive behavioral therapy for adult unipolar depression: A meta-analysis of nonrandomized effectiveness studies. *Journal of Consulting and Clinical Psychology* 2013;**81**:75-88

Hans E, Hiller W. A meta-analysis of nonrandomized effectiveness studies on outpatient cognitive behavioral therapy for adult anxiety disorders. *Clinical Psychology Review* 2013;**33**:954-64

Hanson RK, Gordon A, Harris AJ, Marques JK, Murphy W, Quinsey VL*, et al.* First report of the collaborative outcome data project on the effectiveness of psychological treatment for sex offenders. *Sexual abuse : a journal of research and treatment* 2002;**14**:169-94

Harding R, Liu L, Catalan J, Sherr L. What is the evidence for effectiveness of interventions to enhance coping among people living with HIV disease? A systematic review. *Psychology, Health & Medicine* 2011;**16**:564-87

Hassiotis A, Hall I. Behavioural and cognitive-behavioural interventions for outwardly-directed aggressive behaviour in people with learning disabilities. *Cochrane Database Syst Rev* 2004;**18(4)**:CD003406

Hassiotis AA, Hall I. Behavioural and cognitive-behavioural interventions for outwardly-directed aggressive behaviour in people with learning disabilities. *Cochrane Database Syst Rev* 2008;**16(3)**
Hawley DJ. Psycho-educational interventions in the treatment of arthritis. *Bailliere's Clinical Rheumatology* 1995;**9**:803-23

Hay P. A systematic review of evidence for psychological treatments in eating disorders: 2005-2012. *International Journal of Eating Disorders* 2013;**46**:462-9

Hazell CM, Hayward M, Cavanagh K, Strauss C. A systematic review and meta-analysis of low intensity CBT for psychosis. *Clinical Psychology Review* 2016;**45**:183-92

Hazell P. Depression in children and adolescents. *BMJ clinical evidence* 2009:pii: 1008

Hedman E, Ljotsson B, Lindefors N. Cognitive behavior therapy via the Internet: a systematic review of applications, clinical efficacy and cost-effectiveness. *Expert Review of Pharmacoeconomics and Outcomes Research* 2012;**12**:745-64

Hellenbach M, Brown M, Karatzias T, Robinson R. Psychological interventions for women with intellectual disabilities and forensic care needs: a systematic review of the literature. *Journal of Intellectual Disability Research* 2015;**59**:319-31

Hembree EA, Foa EB, Dorfan NM, Street GP, Kowalski J, Tu X. Do patients drop out prematurely from exposure therapy for PTSD? *Journal of traumatic stress* 2003;**16**:555-62

Henwood KS, Chou S, Browne KD. A systematic review and meta-analysis on the effectiveness of CBT informed anger management. *Aggression and Violent Behavior* 2015;**Part B. 25**:280-92

Herbert V, Kyle SD, Pratt D. Does cognitive behavioural therapy for insomnia improve cognitive performance? A systematic review and narrative synthesis. *Sleep Medicine Reviews* 2018;**39**:37-51

Hernandez JP, Macgowan MJ. Psychosocial Interventions for Women with HIV/AIDS: A Critical Review. *Research on Social Work Practice* 2015;**25**:103-16

Hershcovici T, Achem SR, Jha LK, Fass R. Systematic review: The treatment of noncardiac chest pain. *Alimentary Pharmacology and Therapeutics* 2012;**35**:5-14

Hetzel-Riggin MD, Brausch AM, Montgomery BS. A meta-analytic investigation of therapy modality outcomes for sexually abused children and adolescents: an exploratory study. *Child Abuse and Neglect* 2007;**31**:125-41

Hilfiker R, Meichtry A, Eicher M, Nilsson Balfe L, Knols RH, Verra ML*, et al.* Exercise and other non-pharmaceutical interventions for cancer-related fatigue in patients during or after cancer treatment: a systematic review incorporating an indirect-comparisons meta-analysis. *British journal of sports medicine* 2018;**52**:651-8

Hill A, Brettle A. *Counselling older people: a systematic review*: British Association for Counselling and Psychotherapy; 2005.

Hind D, Cotter J, Thake A, Bradburn M, Cooper C, Isaac C*, et al.* Cognitive behavioural therapy for the treatment of depression in people with multiple sclerosis: a systematic review and meta-analysis. *BMC Psychiatry* 2014;**14**:5

Hogue A, Henderson CE, Ozechowski TJ, Robbins MS. Evidence base on outpatient behavioral treatments for adolescent substance use: updates and recommendations 2007-2013. *Journal of clinical child and adolescent psychology* 2014;**43**:695-720

Hoifodt RS, Strom C, Kolstrup N, Eisemann M, Waterloo K. Effectiveness of cognitive behavioural therapy in primary health care: A review. *Family Practice* 2011;**28**:489-504

Hollis C, Falconer CJ, Martin JL, Whittington C, Stockton S, Glazebrook C*, et al.* Annual Research Review: Digital health interventions for children and young people with mental health problems-A systematic and meta-review. *Journal of Child Psychology and Psychiatry* 2017;**58**:474-503

Hollon SD, Ponniah K. A review of empirically supported psychological therapies for mood disorders in adults. *Depression and Anxiety* 2010;**27**:891-932

Holvast F, Massoudi B, Oude Voshaar RC, Verhaak PFM. Non-pharmacological treatment for depressed older patients in primary care: A systematic review and meta-analysis. *Plos One* 2017;**12(9)**:e0184666

Hoon LS, Chi Sally CW, Hong-Gu H. Effect of psychosocial interventions on outcomes of patients with colorectal cancer: a review of the literature. *European Journal of Oncology Nursing* 2013;**17**:883-91

Hout MS, Wekking EM, Berg IJ, Deelman BG. Psychological treatment of patients with chronic toxic encephalopathy: lessons from studies of chronic fatigue and whiplash. *Psychotherapy and Psychosomatics* 2003;**72**:235-44

Howell D, Oliver TK, Keller-olaman S, Davidson JR, Garland S, Samuels C*, et al.* Sleep disturbance in adults with cancer: A systematic review of evidence for best practices in assessment and management for clinical practice. *Annals of Oncology* 2014;**25**:791-800

Huertas-Ceballos A, Logan S, Bennett C, Macarthur C. Psychosocial interventions for recurrent abdominal pain (RAP) and irritable bowel syndrome (IBS) in childhood. *Cochrane Database Syst Rev* 2008;**(1)**:CD003014

Hulgaard D, Dehlholm-Lambertsen G, Rask CU. Family-based interventions for children and adolescents with functional somatic symptoms: a systematic review. *Journal of Family Therapy* 2019;**41**:4-28

Inouye J, Braginsky N, Kataoka-Yahiro M. Randomized Clinical Trials of Self-Management With Asian/Pacific Islanders. *Clinical Nursing Research* 2011;**20**:366-403

Ipser JC, Singh L, Stein DJ. Meta-analysis of functional brain imaging in specific phobia. *Psychiatry and Clinical Neurosciences* 2013;**67**:311-22

Iruthayarajah J, Alibrahim F, Mehta S, Janzen S, McIntyre A, Teasell R. Cognitive behavioural therapy for aggression among individuals with moderate to severe acquired brain injury: a systematic review and meta-analysis. *Brain Injury* 2018;**32**:1443-9

IsHak WW, Bagot K, Thomas S, Magakian N, Bedwani D, Larson D*, et al.* Quality of life in patients suffering from insomnia. *Innovations in Clinical Neuroscience* 2012;**9**

Ismail A, Moore C, Alshishani N, Yaseen K, Alshehri MA. Cognitive behavioural therapy and pain coping skills training for osteoarthritis knee pain management: a systematic review. *Journal of Physical Therapy Science* 2017;**29**:2228-35

Ismail K, Winkley K, Rabe-Hesketh S. Systematic review and meta-analysis of randomised controlled trials of psychological interventions to improve glycaemic control in patients with type 2 diabetes. *Lancet* 2004;**363**:1589-97

Itza F, Zarza D, Gomez-Sancha F, Salinas J, Bautrant E. Update on the diagnosis and treatment of vulvodynia. *Actas Urologicas Espanolas* 2012;**36**:431-8

Ivarsson T, Skarphedinsson G, Kornor H, Axelsdottir B, Biedilae S, Heyman I*, et al.* The place of and evidence for serotonin reuptake inhibitors (SRIs) for obsessive compulsive disorder (OCD) in children and adolescents: Views based on a systematic review and meta-analysis. *Psychiatry Research* 2015;**227**:93-103

Jarry JL, Ip K. The effectiveness of stand-alone cognitive-behavioural therapy for body image: a meta-analysis. *Body Image* 2005;**2**:317-31

Jibb LA, Nathan PC, Stevens BJ, Seto E, Cafazzo JA, Stephens N*, et al.* Psychological and Physical Interventions for the Management of Cancer-Related Pain in Pediatric and Young Adult Patients: An Integrative Review. *Oncology Nursing Forum* 2015;**42**:E339-E57

Johnsen TJ, Friborg O. The effects of cognitive behavioral therapy as an anti-depressive treatment is falling: A meta-analysis [Erratum appears in Psychol Bull. 2016 Mar;142(3):290; PMID: 26890388]. *Psychological Bulletin* 2015;**141**:747-68

Johnsen TJ, Thimm JC. A meta-analysis of group cognitive-behavioral therapy as an antidepressive treatment: Are we getting better? *Canadian Psychology/Psychologie canadienne* 2018;**59**:15-30

Jones HC, McKenzie-McHarg K, Horsch A. Standard care practices and psychosocial interventions aimed at reducing parental distress following stillbirth: A systematic narrative review. *Journal of Reproductive and Infant Psychology* 2015;**33**:448-65

Jonsson U, Bertilsson G, Allard P, Gyllensvard H, Soderlund A, Tham A*, et al.* Psychological treatment of depression in people aged 65 years and over: A systematic review of efficacy, safety, and cost-effectiveness. *Plos One* 2016;**11(8)**:e0160859

Kaltenthaler E, Parry G, Beverley C. Computerized cognitive behaviour therapy: A systematic review. *Behavioural and Cognitive Psychotherapy* 2004;**32**:31-55

Kaltenthaler E, Parry G, Beverley C, Ferriter M. Computerised cognitive-behavioural therapy for depression: systematic review. *British Journal of Psychiatry* 2008;**193**:181-4

Kampman O, Lehtinen K. Compliance in psychoses. *Acta Psychiatrica Scandinavica* 1999;**100**:167-75

Kanapathy J, Bogle V. The effectiveness of cognitive behavioural therapy for depressed patients with diabetes: A systematic review. *Journal of Health Psychology* 2017;**24(1)**:137-49

Kanapathy J, Bogle V. The effectiveness of cognitive behavioural therapy for depressed patients with diabetes: A systematic review. *Journal of Health Psychology* 2019;**24**:137-49

Kang HS, Kim HK, Park SM, Kim JH. Online-based interventions for sexual health among individuals with cancer: a systematic review. *BMC health services research* 2018;**18**:167

Kangas M, Bovbjerg DH, Montgomery GH. Cancer-related fatigue: a systematic and meta-analytic review of non-pharmacological therapies for cancer patients. *Psychological Bulletin* 2008;**134**:700-41

Kani AS, Shinn AK, Lewandowski KE, Ongur D. Converging effects of diverse treatment modalities on frontal cortex in schizophrenia: A review of longitudinal functional magnetic resonance imaging studies. *Journal of Psychiatric Research* 2017;**84**:256-76

Kanters S, Park JJ, Chan K, Socias ME, Ford N, Forrest JI*, et al.* Interventions to improve adherence to antiretroviral therapy: a systematic review and network meta-analysis. *The Lancet HIV* 2017;**4**:e31-e40

Kar N. Cognitive behavioral therapy for the treatment of post-traumatic stress disorder: A review. *Neuropsychiatric Disease and Treatment* 2011;**7**:167-81

Kazlauskas E, Zelviene P, Lorenz L, Quero S, Maercker A. A scoping review of ICD-11 adjustment disorder research. *European Journal of Psychotraumatology* 2017;**8**:1421819

Kennedy CE, Fonner VA, Armstrong KA, O'Reilly KR, Sweat MD. Increasing HIV serostatus disclosure in low and middle-income countries: A systematic review of intervention evaluations. *Aids* 2015;**29**:S7-S23

Kenny DT. A Systematic Review of Treatments for Music Performance Anxiety. *Anxiety, Stress & Coping: An International Journal* 2005;**18**:183-208

Khoury B, Lecomte T, Fortin G, Masse M, Therien P, Bouchard V*, et al.* Mindfulness-based therapy: A comprehensive meta-analysis. *Clinical Psychology Review* 2013;**33**:763-71

Kleinstauber M, Witthoft M, Hiller W. Efficacy of short-term psychotherapy for multiple medically unexplained physical symptoms: a meta-analysis. *Clin Psychol Rev* 2011;**31**:146-60

Kleinstauber M, Witthoft M, Hiller W. Cognitive-behavioral and pharmacological interventions for premenstrual syndrome or premenstrual dysphoric disorder: a meta-analysis. *Journal of Clinical Psychology in Medical Settings* 2012;**19**:308-19

Kliem S, Kroger C. Prevention of chronic PTSD with early cognitive behavioral therapy. A meta-analysis using mixed-effects modeling. *Behaviour Research and Therapy* 2013;**51**:753-61

Kneebone II, Dunmore E. Psychological management of post-stroke depression. *British Journal of Clinical Psychology* 2000;**39**:53-65

Knight SJ, Scheinberg A, Harvey AR. Interventions in Pediatric Chronic Fatigue Syndrome/Myalgic Encephalomyelitis: A Systematic Review. *Journal of Adolescent Health* 2013;**53**:154-65

Knowles SR, Monshat K, Castle DJ. The efficacy and methodological challenges of psychotherapy for adults with inflammatory bowel disease: A review. *Inflammatory Bowel Diseases* 2013;**19**:2704-15

Kornor H, Winje D, Ekeberg O, Weisaeth L, Kirkehei I, Johansen K*, et al.* Early trauma-focused cognitive-behavioural therapy to prevent chronic post-traumatic stress disorder and related symptoms: a systematic review and meta-analysis. *BMC Psychiatry* 2008;**8:81**

Korotana LM, Dobson KS, Pusch D, Josephson T. A review of primary care interventions to improve health outcomes in adult survivors of adverse childhood experiences. *Clinical Psychology Review* 2016;**46**:59-90

Koulil S, Effting M, Kraaimaat FW, Lankveld W, Helmond T, Cats H*, et al.* Cognitive-behavioural therapies and exercise programmes for patients with fibromyalgia: state of the art and future directions. *Annals of the Rheumatic Diseases* 2007;**66**:571-81

Kozasa EH, Hachul H, Monson C, Pinto L, Jr., Garcia MC, de Araujo Moraes Mello LE*, et al.* Mind-body interventions for the treatment of insomnia: A review. *Revista Brasileira de Psiquiatria* 2010;**32**:437-43

Krishna M, Honagodu A, Rajendra R, Sundarachar R, Lane S, Lepping P. A systematic review and meta-analysis of group psychotherapy for sub-clinical depression in older adults. *International Journal of Geriatric Psychiatry* 2013;**28**:881-8

Krishna M, Jauhari A, Lepping P, Turner J, Crossley D, Krishnamoorthy A. Is group psychotherapy effective in older adults with depression? A systematic review. *International Journal of Geriatric Psychiatry* 2011;**26**:331-40

Kroenke K. Efficacy of treatment for somatoform disorders: A review of randomized controlled trials. *Psychosomatic Medicine* 2007;**69**:881-8

Kroenke K, Swindle R. Cognitive-behavioral therapy for somatization and symptom syndromes: a critical review of controlled clinical trials. *Psychotherapy and Psychosomatics* 2000;**69**:205-15

Kuester A, Niemeyer H, Knaevelsrud C. Internet-based interventions for posttraumatic stress: A meta-analysis of randomized controlled trials. *Clinical Psychology Review* 2016;**43**:1-16

Kwekkeboom KL, Cherwin CH, Lee JW, Wanta B. Mind-Body Treatments for the Pain-Fatigue-Sleep Disturbance Symptom Cluster in Persons with Cancer. *Journal of Pain and Symptom Management* 2010;**39**:126-38

Labelle R, Pouliot L, Janelle A. A systematic review and meta-analysis of cognitive behavioural treatments for suicidal and self-harm behaviours in adolescents. *Canadian Psychology/Psychologie canadienne* 2015;**56**:368-78

Lami MJ, Martinez MP, Sanchez AI. Systematic review of psychological treatment in fibromyalgia. *Current Pain and Headache Reports* 2013;**17(7)**:345

Lang R, Regester A, Lauderdale S, Ashbaugh K, Haring A. Treatment of anxiety in autism spectrum disorders using cognitive behaviour therapy: a systematic review. *Developmental Neurorehabilitation* 2010;**13**:53-63

Lavenberg JG. *Effects of school-based cognitive-behavioral anger interventions: A meta-analysis*: University of Pennsylvania; 2007.

Lee NK, Rawson RA. A systematic review of cognitive and behavioural therapies for methamphetamine dependence. *Drug and Alcohol Review* 2008;**27**:309-17

Leenarts LEW, Diehle J, Doreleijers TAH, Jansma EP, Lindauer RJL. Evidence-based treatments for children with trauma-related psychopathology as a result of childhood maltreatment: A systematic review. *European Child and Adolescent Psychiatry* 2013;**22**:269-83

Leichsenring F. Comparative effects of short-term psychodynamic psychotherapy and cognitive-behavioral therapy in depression: a meta-analytic approach. *Clinical Psychology Review* 2001;**21**:401-19

Leland NE, Marcione N, Niemiec SLS, Kelkar K, Fogelberg D. What is occupational therapy's role in addressing sleep problems among older adults? *Occupation, Participation and Health* 2014;**34**:141-9

Lenz A, Hollenbaugh K. Meta-analysis of trauma-focused cognitive behavioral therapy for treating PTSD and co-occurring depression among children and adolescents. *Counseling Outcome Research and Evaluation* 2015;**6**:18-32

Letourneau NL, Dennis CL, Cosic N, Linder J. The effect of perinatal depression treatment for mothers on parenting and child development: A systematic review. *Depression and Anxiety* 2017;**34**:928-66

Lewey JH, Smith CL, Burcham B, Saunders NL, Elfallal D, O'Toole SK. Comparing the Effectiveness of EMDR and TF-CBT for Children and Adolescents: a Meta-Analysis. *Journal of Child & Adolescent Trauma* 2018;**11**:457-72

Lim C, Sim K, Renjan V, Sam HF, Quah SL. Adapted cognitive-behavioral therapy for religious individuals with mental disorder: A systematic review. *Asian Journal of Psychiatry* 2014;**9**:3-12

Linardon J. Meta-analysis of the effects of cognitive-behavioral therapy on the core eating disorder maintaining mechanisms: implications for mechanisms of therapeutic change. *Cognitive Behaviour Therapy* 2018;**47**:107-25

Linardon J. Rates of abstinence following psychological or behavioral treatments for binge-eating disorder: Meta-analysis. *International Journal of Eating Disorders* 2018;**51**:785-97

Linardon J, Brennan L. The effects of cognitive-behavioral therapy for eating disorders on quality of life: A meta-analysis. *International Journal of Eating Disorders* 2017;**50**:715-30

Linardon J, Fairburn CG, Fitzsimmons-Craft EE, Wilfley DE, Brennan L. The empirical status of the third-wave behaviour therapies for the treatment of eating disorders: A systematic review. *Clinical Psychology Review* 2017;**58**:125-40

Linardon J, Wade T, De La Piedad Garcia X, Brennan L. Psychotherapy for bulimia nervosa on symptoms of depression: A meta-analysis of randomized controlled trials. *International Journal of Eating Disorders* 2017;**50**:1124-36

Linardon J, Wade TD. How many individuals achieve symptom abstinence following psychological treatments for bulimia nervosa? A meta-analytic review. *International Journal of Eating Disorders* 2018;**51(4)**:287-94

Linardon J, Wade TD, De La Piedad Garcia X, Brennan L. The efficacy of cognitive-behavioral therapy for eating disorders: A systematic review and meta-analysis. *Journal of Consulting and Clinical Psychology* 2017;**85**:1080-94

Lincoln TM, Peters E. A systematic review and discussion of symptom specific cognitive behavioural approaches to delusions and hallucinations. *Schizophrenia Research* 2018;**203**:66-79

Liu J, Nie J, Wang Y. Effects of group counseling programs, cognitive behavioral therapy, and sports intervention on internet addiction in east Asia: A systematic review and meta-analysis. *International Journal of Environmental Research and Public Health* 2017;**14(12)**:pii: E1470

Liu ZQ, Zeng X, Duan CY. Neuropsychological rehabilitation and psychotherapy of adult traumatic brain injury patients with depression: A systematic review and meta-analysis. *Journal of Neurosurgical Sciences* 2018;**62**:24-35

Loades ME, Sheils EA, Crawley E. Treatment for paediatric chronic fatigue syndrome or myalgic encephalomyelitis (CFS/ME) and comorbid depression: A systematic review. *BMJ Open* 2016;**6(10)**:e012271

Lohnberg JA. A review of outcome studies on cognitive-behavioral therapy for reducing fear-avoidance beliefs among individuals with chronic pain. *Journal of Clinical Psychology in Medical Settings* 2007;**14**:113-22

Lopes AP, Macedo TF, Coutinho ESF, Figueira I, Ventura PR. Systematic review of the efficacy of cognitive-behavior therapy related treatments for victims of natural disasters: A worldwide problem. *Plos One* 2014;**9(10)**:e109013

Lopez-Pinar C, Martinez-Sanchis S, Carbonell-Vaya E, Fenollar-Cortes J, Sanchez-Meca J. Long-Term Efficacy of Psychosocial Treatments for Adults With Attention-Deficit/Hyperactivity Disorder: A Meta-Analytic Review. *Frontiers in Psychology* 2018;**9**:638

Lopresti AL. Cognitive behaviour therapy and inflammation: A systematic review of its relationship and the potential implications for the treatment of depression. *Australian & New Zealand Journal of Psychiatry* 2017;**51**:565-82

Lovell K, Bee P. Optimising treatment resources for OCD: A review of the evidence base for technology-enhanced delivery. *Journal of Mental Health* 2011;**20**:525-42

Luk BH, Loke AY. A Review of Supportive Interventions Targeting Individuals or Couples Undergoing Infertility Treatment: Directions for the Development of Interventions. *Journal of Sex & Marital Therapy* 2016;**42**:515-33
Ma ZR, Shi LJ, Deng MH. Efficacy of cognitive behavioral therapy in children and adolescents with insomnia: a systematic review and meta-analysis. *Revista brasileira de pesquisas medicas e biologicas* 2018;**51(6)**:e7070

Magill M. Cognitive-behavioral treatment with adult substance users: A meta-analysis. *Dissertation Abstracts International Section A: Humanities and Social Sciences* 2008;**68**:4479

Magill M, Ray LA. Cognitive-behavioral treatment with adult alcohol and illicit drug users: A meta-analysis of randomized controlled trials. *Journal of Studies on Alcohol and Drugs* 2009;**70**:516-27

Malejko K, Abler B, Plener PL, Straub J. Neural Correlates of Psychotherapeutic Treatment of Post-traumatic Stress Disorder: A Systematic Literature Review. *Frontiers in psychiatr* 2017;**8**:85

Malouff JM, Thorsteinsson EB, Rooke SE, Bhullar N, Schutte NS. Efficacy of cognitive behavioral therapy for chronic fatigue syndrome: a meta-analysis. *Clinical Psychology Review* 2008;**28**:736-45

Marchesi C, Ossola P, Amerio A, Daniel BD, Tonna M, De Panfilis C. Clinical management of perinatal anxiety disorders: A systematic review. *Journal of Affective Disorders* 2016;**190**:543-50

Marotta PL. A Systematic Review of Behavioral Health Interventions for Sex Offenders With Intellectual Disabilities. *Sexual abuse : a journal of research and treatment* 2017;**29**:148-85

Martorella G, Boitor M, Berube M, Fredericks S, Le May S, Gelinas C. Tailored Web-Based Interventions for Pain: Systematic Review and Meta-Analysis. *Journal of Medical Internet Research* 2017;**19**:e385

Mason L, Peters E, Kumari V. Functional connectivity predictors and mechanisms of cognitive behavioural therapies: A systematic review with recommendations. *Australian & New Zealand Journal of Psychiatry* 2016;**50**:311-21

Matcham F, Rayner L, Hutton J, Monk A, Steel C, Hotopf M. Self-help interventions for symptoms of depression, anxiety and psychological distress in patients with physical illnesses: A systematic review and meta-analysis. *Clinical Psychology Review* 2014;**34**:141-57

Matthews E, Carter P, Page M, Dean G, Berger A. Sleep-Wake Disturbance: A systematic review of evidence-based interventions for management in patients with cancer. *Clinical Journal of Oncology Nursing* 2018;**22**:37-52

Matthews H, Grunfeld EA, Turner A. The efficacy of interventions to improve psychosocial outcomes following surgical treatment for breast cancer: a systematic review and meta-analysis. *Psycho-Oncology* 2017;**26**:593-607

Mayo-Wilson E, Dias S, Mavranezouli I, Kew K, Clark DM, Ades AE*, et al.* Psychological and pharmacological interventions for social anxiety disorder in adults: A systematic review and network meta-analysis. *The Lancet Psychiatry* 2014;**1**:368-76

McCombie A, Gearry R, Andrews J, Mikocka-Walus A, Mulder R. Computerised Cognitive Behavioural Therapy for Psychological Distress in Patients with Physical Illnesses: A Systematic Review. *Journal of Clinical Psychology in Medical Settings* 2015;**22**:20-44

McCurry SM, Logsdon RG, Teri L, Vitiello MV. Evidence-based psychological treatments for insomnia in older adults. *Psychology and Aging* 2007;**22**:18-27

McDonagh MS, Dana T, Selph S, Devine EB, Cantor A, Bougatsos C*, et al. Treatments for Schizophrenia in Adults: A Systematic Review*. Rockville (US): Agency for Healthcare Research and Quality; 2017.

McNaughton JL. Brief interventions for depression in primary care: a systematic review. *Canadian Family Physician* 2009;**55**:789-96

Mellentin AI, Skot L, Nielsen B, Schippers GM, Nielsen AS, Stenager E*, et al.* Cue exposure therapy for the treatment of alcohol use disorders: A meta-analytic review. *Clinical Psychology Review* 2017;**57**:195-207

Mendes DD, Mello MF, Ventura P, Passarela CM, Mari JJ. A systematic review on the effectiveness of cognitive behavioral therapy for posttraumatic stress disorder. *International Journal of Psychiatry in Medicine* 2008;**38**:241-59

Merlin JS, Bulls HW, Vucovich LA, Edelman EJ, Starrels JL. Pharmacologic and non-pharmacologic treatments for chronic pain in individuals with HIV: a systematic review. *AIDS Care* 2016;**28**:1506-15

Mewton L, Smith J, Rossouw P, Andrews G. Current perspectives on Internet-delivered cognitive behavioral therapy for adults with anxiety and related disorders. *Psychology Research & Behavior Management* 2014;**7**:37-46

Miklowitz DJ, Scott J. Psychosocial treatments for bipolar disorder: Cost-effectiveness, mediating mechanisms, and future directions. *Bipolar Disorders* 2009;**11**:110-22

Miles LA, Cooper RL, Nugent WR, Ellis RA. Sexual addiction: A literature review of treatment interventions. *Journal of Human Behavior in the Social Environment* 2016;**26**:89-99

Miller-Graff LE, Campion K. Interventions for Posttraumatic Stress With Children Exposed to Violence: Factors Associated With Treatment Success. *Journal of Clinical Psychology* 2016;**72**:226-48

Minen MT, Torous J, Raynowska J, Piazza A, Grudzen C, Powers S*, et al.* Electronic behavioral interventions for headache: a systematic review. *Journal of Headache and Pain* 2016;**17(1)** 51

Mitchell K, Carr A. Anorexia and bulimia. In: Carr A, editor. *What Works with Children, Adolescents and Adults? A Critical Review of Psychological Interventions with Children, Adolescents and their Families*Florence, KY: Taylor & Frances/Routledge; US; 2000:233-57.

Montgomery L, Robinson C, Seaman EL, Haeny AM. A scoping review and meta-analysis of psychosocial and pharmacological treatments for cannabis and tobacco use among African Americans. *Psychology of Addictive Behaviors* 2017;**31**:922-43

Moore M, Carr A. Anxiety disorders. In: Carr A, editor. *What Works with Children, Adolescents and Adults? A Critical Review of Psychological Interventions with Children, Adolescents and their Families*Florence, KY: Taylor & Frances/Routledge; US; 2000:178-202.

Moore TH, Kapur N, Hawton K, Richards A, Metcalfe C, Gunnell D. Interventions to reduce the impact of unemployment and economic hardship on mental health in the general population: a systematic review. *Psychological Medicine* 2017;**47**:1062-84

Morin CM, Bootzin RR, Buysse DJ, Edinger JD, Espie CA, Lichstein KL. Psychological and behavioral treatment of insomnia: Update of the recent evidence (1998-2004). *Sleep* 2006;**29**:1398-414

Morrison AP. Cognitive behaviour therapy for first episode psychosis: Good for nothing or fit for purpose? *Psychosis: Psychological, Social and Integrative Approaches* 2009;**1**:103-12

Mueller C, Wesenberg S, Nestmann F, Stubbs B, Bebbington P, Raymont V. Interventions to enhance coping after traumatic brain injury: A systematic review. *International Journal of Therapy & Rehabilitation* 2018;**25**:107-19

Muller I, Yardley L. Telephone-delivered cognitive behavioural therapy: A systematic review and meta-analysis. *Journal of Telemedicine and Telecare* 2011;**17**:177-84

Murphy E, Carr A. Paediatric pain problems. In: Carr A, editor. *What Works with Children and Adolescents? A Critical Review of Psychological Interventions with Children, Adolescents and their Families*Florence, KY: Taylor & Frances/Routledge; US; 2000:258-79.

Nadiga DN, Hensley PL, Uhlenhuth EH. Review of the long-term effectiveness of cognitive behavioral therapy compared to medications in panic disorder. *Depression and Anxiety* 2003;**17**:58-64

Necrason E. *Cognitive behavioral interventions for pediatric procedure-related pain: A comprehensive methodological review*: University of Hartford; 2016.

Neil AL, Christensen H. Australian school-based prevention and early intervention programs for anxiety and depession: A systematic review. *Medical Journal of Australia* 2007;**186**:305-8

Neil AL, Christensen H. Efficacy and effectiveness of school-based prevention and early intervention programs for anxiety. *Clinical Psychology Review* 2009;**29**:208-15

Nevo GA, Manassis K. Outcomes for treated anxious children: A critical review of long-term-follow-up studies. *Depression and Anxiety* 2009;**26**:650-60

Newby JM, McKinnon A, Kuyken W, Gilbody S, Dalgleish T. Systematic review and meta-analysis of transdiagnostic psychological treatments for anxiety and depressive disorders in adulthood. *Clinical Psychology Review* 2015;**40**:91-110

Nicholls JL, Azam MA, Burns LC, Englesakis M, Sutherland AM, Weinrib AZ*, et al.* Psychological treatments for the management of postsurgical pain: a systematic review of randomized controlled trials. *Patient Related Outcome Measures* 2018;**9**:49-64

Nieuwsma JA, Trivedi RB, McDuffie J, Kronish I, Benjamin D, Williams JW. Brief psychotherapy for depression: A systematic review and meta-analysis. *International Journal of Psychiatry in Medicine* 2012;**43**:129-51

Nigatu YT, Huang J, Rao S, Gillis K, Merali Z, Wang J. Indicated Prevention Interventions in the Workplace for Depressive Symptoms: A Systematic Review and Meta-analysis. *American Journal of Preventive Medicine* 2019;**56**:e23-e33

Normann N, Van Emmerik AAP, Morina N. The efficacy of metacognitive therapy for anxiety and depression: A meta-analytic review. *Depression and Anxiety* 2014;**31**:402-11

Norton AR, Abbott MJ, Norberg MM, Hunt C. A systematic review of mindfulness and acceptance-based treatments for social anxiety disorder. *Journal of Clinical Psychology* 2015;**71**:283-301

O'Brien AP, McNeil KA, Fletcher R, Conrad A, Wilson AJ, Jones D*, et al.* New Fathers' Perinatal Depression and Anxiety-Treatment Options: An Integrative Review. *American journal of men's health* 2017;**11**:863-76

O'Connor E, Rossom RC, Henninger M, Groom HC, Burda BU. Primary Care Screening for and Treatment of Depression in Pregnant and Postpartum Women: Evidence Report and Systematic Review for the US Preventive Services Task Force. *JAMA* 2016;**315**:388-406

O'Connor E, Rossom RC, Henninger M, Groom HC, Burda BU, Henderson JT*, et al. Screening for Depression in Adults: An Updated Systematic Evidence Review for the U.S. Preventative Services Task Force*. Rockville (US): Agency for Healthcare Research and Quality; 2016.

Oei TPS, Llamas M, Devilly GJ. The efficacy and cognitive processes of cognitive behaviour therapy in the treatment of panic disorder with agoraphobia. *Behavioural and Cognitive Psychotherapy* 1999;**27**:63-88

Oing T, Prescott J. Implementations of Virtual Reality for Anxiety-Related Disorders: Systematic Review. *JMIR Serious Games* 2018;**6**:e10965

Okuzawa N, Kline E, Fuertes J, Negi S, Reeves G, Himelhoch S*, et al.* Psychotherapy for adolescents and young adults at high risk for psychosis: A systematic review. *Early Intervention in Psychiatry* 2014;**8**:307-22

Ost LG, Ollendick TH. Brief, intensive and concentrated cognitive behavioral treatments for anxiety disorders in children: A systematic review and meta-analysis. *Behaviour Research and Therapy* 2017;**97**:134-45

Ost LG, Riise EN, Wergeland GJ, Hansen B, Kvale G. Cognitive behavioral and pharmacological treatments of OCD in children: A systematic review and meta-analysis. *Journal of Anxiety Disorders* 2016;**43**:58-69

Ostadhashemi L, Khankeh HR, Eghlima M, Arshi M, Nafei A, Asangari B*, et al.* Family-oriented psychosocial intervention in children with cancer: A systematic review. *Journal of Kermanshah University of Medical Sciences* 2016;**20**:43-50

Pajak R, Lackner J, Kamboj SK. A systematic review of minimal-contact psychological treatments for symptom management in Irritable Bowel Syndrome. *Journal of Psychosomatic Research* 2013;**75**:103-12

Patel MX, Baker D, Nosarti C. Injection phobia: A systematic review of psychological treatments. *Behavioural and Cognitive Psychotherapy* 2005;**33**:343-9

Pearl SB, Norton PJ. Transdiagnostic versus diagnosis specific cognitive behavioural therapies for anxiety: A meta-analysis. *Journal of Anxiety Disorders* 2017;**46**:11-24

Pedersen SS, Van Den Broek KC, Sears Jr SF. Psychological intervention following implantation of an implantable defibrillator: A review and future recommendations. *PACE - Pacing and Clinical Electrophysiology* 2007;**30**:1546-54

Pei-Fan M, Yu-Chih C, Shu-Chen C. The effectiveness of non-pharmacological pain management in relieving chronic pain for children and adolescents. *JBI Library of Systematic Reviews* 2009;**7**:1489-543

Penn DL, Waldheter EJ, Perkins DO, Mueser KT, Lieberman JA. Psychosocial treatment for first-episode psychosis: A research update. *American Journal of Psychiatry* 2005;**162**:2220-32

Petry NM, Ginley MK, Rash CJ. A systematic review of treatments for problem gambling. *Psychology of Addictive Behaviors* 2017;**31**:951-61

Phillipou A, Rossell SL, Wilding HE, Castle DJ. Randomised controlled trials of psychological & pharmacological treatments for body dysmorphic disorder: A systematic review. *Psychiatry Research* 2016;**245**:179-85

Pichora-Fuller MK, Santaguida P, Hammill A, Oremus M, Westerberg B, Ali U*, et al. Evaluation and Treatment of Tinnitus: Comparative Effectiveness*. Rockville (US): Agency for Healthcare Research and Quality; 2013.

Pilling S, Bebbington P, Kuipers E, Garety P, Geddes J, Orbach G*, et al.* Psychological treatments in schizophrenia: I. Meta-analysis of family intervention and cognitive behaviour therapy. *Psychological Medicine* 2002;**32**:763-82

Pineros-Leano M, Liechty JM, Piedra LM. Latino immigrants, depressive symptoms, and cognitive behavioral therapy: A systematic review. *Journal of Affective Disorders* 2017;**208**:567-76

Pittock A, Mair E. Are psychotherapies effective in the treatment of Anorexia Nervosa? - A systematic review. *Journal of Indian Association for Child and Adolescent Mental Health* 2010;**6**:55-71

Polizzi DM, MacKenzie DL, Hickman LJ. What works in adult sex offender treatment? A review of prison- and non-prison-based treatment programs. *International Journal of Offender Therapy and Comparative Criminology* 1999;**43**:357-74

Ponniah K, Hollon SD. Empirically supported psychological treatments for adult acute stress disorder and posttraumatic stress disorder: a review. *Depression and Anxiety* 2009;**26**:1086-109

Ponniah K, Magiati I, Hollon SD. An update on the efficacy of psychological treatments for obsessive-compulsive disorder in adults. *Journal of Obsessive-Compulsive and Related Disorders* 2013;**2**:207-18

Pontillo M, De Crescenzo F, Vicari S, Pucciarini ML, Averna R, Santonastaso O*, et al.* Cognitive behavioural therapy for auditory hallucinations in schizophrenia: A review. *World Journal of Psychiatry* 2016;**6**:372-80

Post KE, Flanagan J. Web based survivorship interventions for women with breast cancer: An integrative review. *European Journal of Oncology Nursing* 2016;**25**:90-9

Prazeres AM, Nascimento AL, Fontenelle LF. Cognitive-behavioral therapy for body dysmorphic disorder: A review of its efficacy. *Neuropsychiatric Disease and Treatment* 2013;**9**:307-16

Priemer M, Talbot F. CBT guided self-help compares favourably to gold standard therapist-administered CBT and shows unique benefits over traditional treatment. *Behaviour Change* 2013;**30**:227-40

Probyn K, Bowers H, Mistry D, Caldwell F, Underwood M, Patel S*, et al.* Non-pharmacological self-management for people living with migraine or tension-type headache: A systematic review including analysis of intervention components. *BMJ Open* 2017;**7(8)**:e016670

Querstret D, Cropley M. Assessing treatments used to reduce rumination and/or worry: A systematic review. *Clinical Psychology Review* 2013;**33**:996-1009

Quinn A, Mowbray O. Effective Treatments for Older Adult Baby Boomers with Alcohol-Use Disorders: A Literature Review. *Journal of Social Work Practice in the Addictions* 2018;**18**:389-410

Radu M, Moldovan R, Pintea S, Baban A, Dumitrascu D. Predictors of outcome in cognitive and behavioural interventions for irritable bowel syndrome. A meta-analysis. *Journal of Gastrointestinal & Liver Diseases* 2018;**27**:257-63

Ramchandani P, Jones DP. Treating psychological symptoms in sexually abused children: From research findings to service provision. *The British Journal of Psychiatry* 2003;**183**:484-90

Rampling J, Furtado V, Winsper C, Marwaha S, Lucca G, Livanou M*, et al.* Non-pharmacological interventions for reducing aggression and violence in serious mental illness: A systematic review and narrative synthesis. *European Psychiatry* 2016;**34**:17-28

Rathbone AL, Clarry L, Prescott J. Assessing the Efficacy of Mobile Health Apps Using the Basic Principles of Cognitive Behavioral Therapy: Systematic Review. *Journal of Medical Internet Research* 2017;**19**:e399

Read H, Roush S, Downing D. Early Intervention in Mental Health for Adolescents and Young Adults: A Systematic Review. *American Journal of Occupational Therapy* 2018;**72**:1-8

Rees-Jones A. *Examining the utility of assessment tools and group intervention programmes for mentally disordered offenders*: University of Birmingham; 2011.

Regan B, Varanelli L. Adjustment, depression, and anxiety in mild cognitive impairment and early dementia: a systematic review of psychological intervention studies. *International Psychogeriatrics* 2013;**25**:1963-84

Regehr C, Glancy D, Pitts A. Interventions to reduce stress in university students: A review and meta-analysis. *Journal of Affective Disorders* 2013;**148**:1-11

Reger MA, Gahm GA. A meta-analysis of the effects of Internet- and computer-based cognitive-behavioral treatments for anxiety. *Journal of Clinical Psychology* 2009;**65**:53-75

Rice SM, Goodall J, Hetrick SE, Parker AG, Gilbertson T, Amminger GP*, et al.* Online and social networking interventions for the treatment of depression in young people: a systematic review. *Journal of Medical Internet Research* 2014;**16**:e206

Richardson T, Stallard P, Velleman S. Computerised cognitive behavioural therapy for the prevention and treatment of depression and anxiety in children and adolescents: a systematic review. *Clinical Child and Family Psychology Review* 2010;**13**:275-90

Richter K, Acker J, Adam S, Niklewski G. Prevention of fatigue and insomnia in shift workers-a review of non-pharmacological measures. *EPMA Journal* 2016;**7(1)**:16

Riemann D D. Does Effective Management of Sleep Disorders Reduce Depressive Symptoms and the Risk of Depression? *Drugs* 2009;**69**:43-64

Rith-Najarian LR, Mesri B, Park AL, Sun M, Chavira DA, Chorpita BF. Durability of Cognitive Behavioral Therapy Effects for Youth and Adolescents With Anxiety, Depression, or Traumatic Stress:A Meta-Analysis on Long-Term Follow-Ups. *Behavior Therapy* 2019;**50**:225-40

Rodgers M, Asaria M, Walker S, McMillan D, Lucock M, Harden M*, et al.* The clinical effectiveness and cost-effectiveness of low-intensity psychological interventions for the secondary prevention of relapse after depression: a systematic review. *Health Technology Assessment* 2012;**16**:1-130

Rolfsnes ES, Idsoe T. School-based intervention programs for PTSD symptoms: a review and meta-analysis. *Journal of traumatic stress* 2011;**24**:155-65

Romanelli RJ, Wu FM, Gamba R, Mojtabai R, Segal JB. Behavioral therapy and serotonin reuptake inhibitor pharmacotherapy in the treatment of obsessive-compulsive disorder: a systematic review and meta-analysis of head-to-head randomized controlled trials. *Depression and Anxiety* 2014;**31**:641-52

Romero-Martinez A, Hidalgo-Moreno G, Moya-Albiol L. Neuropsychological consequences of chronic stress: the case of informal caregivers. *Aging & Mental Health* 2018;**18**:1-13

Rooksby M, Elouafkaoui P, Humphris G, Clarkson J, Freeman R. Internet-assisted delivery of cognitive behavioural therapy (CBT) for childhood anxiety: Systematic review and meta-analysis. *Journal of Anxiety Disorders* 2015;**29**:83-92

Rossy LA, Buckelew SP, Dorr N, Hagglund KJ, Thayer JF, McIntosh MJ*, et al.* A meta-analysis of fibromyalgia treatment interventions. *Annals of Behavioral Medicine* 1999;**21**:180-91

Rubin GJ, Das Munshi J, Wessely S. A systematic review of treatments for electromagnetic hypersensitivity. *Psychotherapy and Psychosomatics* 2006;**75**:12-8

Rutten JM, Korterink JJ, Venmans LM, Benninga MA, Tabbers MM. Nonpharmacologic treatment of functional abdominal pain disorders: a systematic review. *Pediatrics* 2015;**135**:522-35

Sajid S, Kotwal AA, Dale W. Interventions to improve decision making and reduce racial and ethnic disparities in the management of prostate cancer: A systematic review. *Journal of General Internal Medicine* 2012;**27**:1068-78

Salcedo S, Gold AK, Sheikh S, Marcus PH, Nierenberg AA, Deckersbach T*, et al.* Empirically supported psychosocial interventions for bipolar disorder: Current state of the research. *Journal of Affective Disorders* 2016;**201**:203-14

Salmoirago-Blotcher E, Ockene IS. Methodological limitations of psychosocial interventions in patients with an implantable cardioverter-defibrillator (ICD) A systematic review. *BMC Cardiovascular Disorders* 2009;**9(56)**

Salomonsson S, Hedman-Lagerlof E, Ost LG. Sickness absence: a systematic review and meta-analysis of psychological treatments for individuals on sick leave due to common mental disorders. *Psychological Medicine* 2018;**48(12)**:1954-65

Sawyer MC, Nunez DE. Cognitive-Behavioral Therapy for Anxious Children: From Evidence to Practice. *Worldviews on Evidence-Based Nursing* 2014;**11**:65-71

Schmid G, Henningsen P, Dieterich M, Sattel H, Lahmann C, Schmid G*, et al.* Psychotherapy in dizziness: a systematic review. *Journal of Neurology, Neurosurgery & Psychiatry* 2011;**82**:601-6

Scott RW, Mughelli K, Deas D. An overview of controlled studies of anxiety disorders treatment in children and adolescents. *Journal of the National Medical Association* 2005;**97**:13-24

Seidler GH, Wagner FE. Comparing the efficacy of EMDR and trauma-focused cognitive-behavioral therapy in the treatment of PTSD: a meta-analytic study. *Psychological Medicine* 2006;**36**:1515-22

Shapiro JR, Berkman ND, Brownley KA, Sedway JA, Lohr KN, Bulik CM. Bulimia nervosa treatment: a systematic review of randomized controlled trials. *International Journal of Eating Disorders* 2007;**40**:321-36

Shepherd C, Beail N. A systematic review of the effectiveness of psychoanalysis, psychoanalytic and psychodynamic psychotherapy with adults with intellectual and developmental disabilities: progress and challenges. *Psychoanalytic Psychotherapy* 2017;**31**:94-117

Shin JC, Kim J, Grigsby-Toussaint D. Mobile Phone Interventions for Sleep Disorders and Sleep Quality: Systematic Review. *JMIR MHealth and UHealth* 2017;**5**:e131

Sigra S, Hesselmark E, Bejerot S. Treatment of PANDAS and PANS: a systematic review. *Neuroscience and Biobehavioral Reviews* 2018;**86**:51-65

Sikorski C, Lakhanpaul M, Costello A, Heys M. A systematic review: 'Can postnatal women's groups improve health outcomes for women and children in high-income countries?'. *Archives of Disease in Childhood* 2014;**(1)**:A200

Silverman WK, Pina AA. Psychosocial treatments for phobic and anxiety disorders in youth. In: Steele RG, Elkin TD, Roberts MC, editors. *Handbook of Evidence-Based Therapies for Children and Adolescents.* New York, NY: Springer Science + Business Media; US; 2008:65-82.

Silverman WK, Pina AA, Viswesvaran C. Evidence-based psychosocial treatments for phobic and anxiety disorders in children and adolescents. *Journal of Clinical Child and Adolescent Psychology* 2008;**37**:105-30

Simblett S, Birch J, Matcham F, Yaguez L, Morris R. A Systematic Review and Meta-Analysis of e-Mental Health Interventions to Treat Symptoms of Posttraumatic Stress. *JMIR Mental Health* 2017;**4**:e14

Simning A, Simons KV. Treatment of depression in nursing home residents without significant cognitive impairment: a systematic review. *International Psychogeriatrics* 2017;**29**:209-26

Sinclair S, Ob Sutherland R, Henderson S, O'Callaghan V, Dalton T, Jefford M*, et al.* The impact of fear of cancer recurrence on wellnessn. *Asia-Pacific Journal of Clinical Oncology* 2013;**3)**:84-5

Sinha JW, Rosenberg LB. A critical review of trauma interventions and religion among youth exposed to community violence. *Journal of Social Service Research* 2013;**39**:436-54

Skapinakis P, Caldwell D, Hollingworth W, Bryden P, Fineberg N, Salkovskis P*, et al.* A systematic review of the clinical effectiveness and cost-effectiveness of pharmacological and psychological interventions for the management of obsessive-compulsive disorder in children/adolescents and adults. *Health Technology Assessment* 2016;**20**

Skarphedinsson G, Hanssen-Bauer K, Kornor H, Heiervang ER, Landro NI, Axelsdottir B*, et al.* Standard individual cognitive behaviour therapy for paediatric obsessive-compulsive disorder: A systematic review of effect estimates across comparisons. *Nordic Journal of Psychiatry* 2015;**69**:81-92

Smith TE, Weston CA, Lieberman JA. Schizophrenia (maintenance treatment). *BMJ clinical evidence* 2009:pii: 1007

Sockol LE. A systematic review of the efficacy of cognitive behavioral therapy for treating and preventing perinatal depression. *Journal of Affective Disorders* 2015;**177**:7-21

Sockol LE, Epperson CN, Barber JP. A meta-analysis of treatments for perinatal depression. *Clinical Psychology Review* 2011;**31**:839-49

Song F, Huttunen-Lenz M, Holland R. Effectiveness of complex psycho-educational interventions for smoking relapse prevention: an exploratory meta-analysis. *Journal of Public Health* 2010;**32**:350-9

St Amand A, Bard DE, Silovsky JF. Meta-analysis of treatment for child sexual behavior problems: practice elements and outcomes. *Child Maltreatment* 2008;**13**:145-66

Stafford MR, Mayo-Wilson E, Loucas CE, James A, Hollis C, Birchwood M*, et al.* Efficacy and safety of pharmacological and psychological interventions for the treatment of psychosis and schizophrenia in children, adolescents and young adults: A systematic review and meta-analysis. *Plos One* 2015;**10(2)**:e0117166

Stapleton JL, Hillhouse J, Levonyan-Radloff K, Manne SL. Review of interventions to reduce ultraviolet tanning: Need for treatments targeting excessive tanning, an emerging addictive behavior. *Psychology of Addictive Behaviors* 2017;**31**:962-78

Stefanopoulou E, Grunfeld EA. Mind-body interventions for vasomotor symptoms in healthy menopausal women and breast cancer survivors. A systematic review. *Journal of Psychosomatic Obstetrics and Gynecology* 2017;**38**:210-25

Steinert C, Munder T, Rabung S, Hoyer J, Leichsenring F. Psychodynamic Therapy: As Efficacious as Other Empirically Supported Treatments? A Meta-Analysis Testing Equivalence of Outcomes. *American Journal of Psychiatry* 2017;**174**:943-53

Stevanovic D, Tadic I, Knez R. Are antidepressants effective in quality of life improvement among children and adolescents? A systematic review. *CNS Spectrums* 2014;**19**:134-41

Stratton E, Lampit A, Choi I, Calvo RA, Harvey SB, Glozier N. Effectiveness of eHealth interventions for reducing mental health conditions in employees: A systematic review and meta-analysis. *Plos One* 2017;**12(12)**:e0189904

Subnis UB, Starkweather AR, McCain NL, Brown RF. Psychosocial therapies for patients with cancer: A current review of interventions using psychoneuroimmunology-based outcome measures. *Integrative Cancer Therapies* 2014;**13**:85-104

Sylvia LG, Tilley CA, Lund HG, Sachs GS. Psychosocial interventions: Empirically-derived treatments for bipolar disorder. *Current Psychiatry Reviews* 2008;**4**:108-13

Sztein DM, Koransky CE, Fegan L, Himelhoch S. Efficacy of cognitive behavioural therapy delivered over the Internet for depressive symptoms: A systematic review and meta-analysis. *Journal of Telemedicine & Telecare* 2017;**24(8)**:527-39

Tatrow K, Montgomery GH. Cognitive behavioral therapy techniques for distress and pain in breast cancer patients: a meta-analysis. *Journal of Behavioral Medicine* 2006;**29**:17-27

Tavares LR, Barbosa MR. Efficacy of group psychotherapy for geriatric depression: A systematic review. *Archives of Gerontology & Geriatrics* 2018;**78**:71-80

Teng EJ, Hiatt EL, McClair V, Kunik ME, Frueh B, Stanley MA. Efficacy of posttraumatic stress disorder treatment for comorbid panic disorder: A critical review and future directions for treatment research. *Clinical Psychology: Science and Practice* 2013;**20**:268-84

Thoma NC, McKay D, Gerber AJ, Milrod BL, Edwards AR, Kocsis JH. A quality-based review of randomized controlled trials of cognitive-behavioral therapy for depression: An assessment and metaregression. *American Journal of Psychiatry* 2012;**169**:22-30

Thomas WJ, Hauson AO, Lambert JE, Stern MJ, Gamboa JM, Allen KE*, et al.* A meta-analysis of the effectiveness of cognitive-behavioural therapies for late-life depression. *Canadian Journal of Counselling and Psychotherapy* 2018;**52**:78-117

Thomas WJF. *Effectiveness of cognitive-behavioral therapies for late-life depression: Research synthesis and meta-analysis*: Alliant International University; 2015.

Thompson C, Fernandez de la Cruz L, Mataix-Cols D, Onwumere J. A systematic review and quality assessment of psychological, pharmacological, and family-based interventions for hoarding disorder. *Asian Journal of Psychiatry* 2017;**27**:53-66

Thompson DM, Hall DA, Walker DM, Hoare DJ. Psychological Therapy for People with Tinnitus: A Scoping Review of Treatment Components. *Ear and hearing* 2017;**38**:149-58

Thompson-Brenner HJ. *Implications for the treatment of bulimia nervosa: A meta-analysis of efficacy trials and a naturalistic study of treatment in the community*: University of Michigan; 2003.

Thorp SR, Ayers CR, Nuevo R, Stoddard JA, Sorrell JT, Wetherell JL. Meta-analysis comparing different behavioral treatments for late-life anxiety. *The American journal of geriatric psychiatry* 2009;**17**:105-15

Tol WA, Stavrou V, Greene MC, Mergenthaler C, van Ommeren M, Garcia Moreno C. Sexual and gender-based violence in areas of armed conflict: a systematic review of mental health and psychosocial support interventions. *Conflict & Health* 2013;**7**:16

Trautmann E, Lackschewitz H, Kroner-Herwig B. Psychological treatment of recurrent headache in children and adolescents - A meta-analysis. *Cephalalgia* 2006;**26**:1411-26

Tribe RH, Sendt KV, Tracy DK. A systematic review of psychosocial interventions for adult refugees and asylum seekers. *Journal of Mental Health* 2017; **Early online**:1-15

Trinidade A, Goebel JA. Persistent Postural-Perceptual Dizziness-A Systematic Review of the Literature for the Balance Specialist. *Otology & Neurotology* 2018;**39**:1291-303

Tullar JM, Brewer S, Amick IBC, Irvin E, Mahood Q, Pompeii LA*, et al.* Occupational Safety and Health Interventions to Reduce Musculoskeletal Symptoms in the Health Care Sector. *Journal of Occupational Rehabilitation* 2010;**20(2)**:199-219

Tundo A, Necci R. Cognitive-behavioural therapy for obsessive-compulsive disorder co-occurring with psychosis: Systematic review of evidence. *World Journal of Psychiatry* 2016;**6**:449-55

Turner DT, McGlanaghy E, Cuijpers P, Van Der Gaag M, Karyotaki E, MacBeth A. A Meta-Analysis of Social Skills Training and Related Interventions for Psychosis. *Schizophrenia Bulletin* 2018;**44**:475-91

Uman LS, Birnie KA, Noel M, Parker JA, Chambers CT, McGrath PJ*, et al.* Psychological interventions for needle-related procedural pain and distress in children and adolescents. *Cochrane Database Syst Rev* 2013;**(10)**:CD005179

Ung D, Selles R, Small BJ, Storch EA. A Systematic Review and Meta-Analysis of Cognitive-Behavioral Therapy for Anxiety in Youth with High-Functioning Autism Spectrum Disorders. *Child psychiatry and human development* 2015;**46**:533-47

Unwin G, Tsimopoulou I, Kroese BS, Azmi S. Effectiveness of cognitive behavioural therapy (CBT) programmes for anxiety or depression in adults with intellectual disabilities: A review of the literature. *Research in Developmental Disabilities* 2016;**51-52**:60-75

Vallarino M, Henry C, Etain B, Gehue LJ, Macneil C, Scott EM*, et al.* An evidence map of psychosocial interventions for the earliest stages of bipolar disorder. *The Lancet Psychiatry* 2015;**2**:548-63

Vally Z, Maggott C. Evaluating the outcome of cultural adaptations of cognitive-behavioural therapy for adult depression: A meta-analysis of treatment studies in developing countries. *International Journal for the Advancement of Counselling* 2015;**37**:293-304

Van der Oord S, Prins PJM, Oosterlaan J, Emmelkamp PMG. Efficacy of methylphenidate, psychosocial treatments and their combination in school-aged children with ADHD: A meta-analysis. *Clinical Psychology Review* 2008;**28**:783-800

Van Hout MSE, Wekking EM, Berg IJ, Deelman BG. Psychological treatment of patients with chronic toxic encephalopathy: Lessons from studies of chronic fatigue and whiplash. *Psychotherapy and Psychosomatics* 2003;**72**:235-44

van Straten A, Geraedts A, Verdonck-de Leeuw I, Andersson G, Cuijpers P. Psychological treatment of depressive symptoms in patients with medical disorders: A meta-analysis. *Journal of Psychosomatic Research* 2010;**69**:23-32

van Straten A, van der Zweerde T, Kleiboer A, Cuijpers P, Morin CM, Lancee J. Cognitive and behavioral therapies in the treatment of insomnia: A meta-analysis. *Sleep Medicine Reviews* 2018;**38**:3-16

van Vilsteren M, van Oostrom SH, de Vet HCW, Franche RL, Boot CRL, Anema JR. Workplace interventions to prevent work disability in workers on sick leave. *Cochrane Database Syst Rev* 2015;**(10)**:CD006955

van Zoonen K, Buntrock C, Ebert DD, Smit F, Reynolds ICF, Beekman ATF*, et al.* Preventing the onset of major depressive disorder: A meta-analytic review of psychological interventions. *International Journal of Epidemiology* 2014;**43**:318-29

Vasa RA, Carroll LM, Nozzolillo AA, Mahajan R, Mazurek MO, Bennett AE*, et al.* A Systematic Review of Treatments for Anxiety in Youth with Autism Spectrum Disorders. *Journal of Autism and Developmental Disorders* 2014;**44**:3215-29

Verdejo-Garcia A, Alcazar-Corcoles MA, Albein-Urios N. Neuropsychological Interventions for Decision-Making in Addiction: a Systematic Review. *Neuropsychology review* 2018;**26**:26

Vereenooghe L, Langdon PE. Psychological therapies for people with intellectual disabilities: A systematic review and meta-analysis. *Research in Developmental Disabilities* 2013;**34**:4085-102

Visser E, Gosens T, Den Oudsten BL, De Vries J. The course, prediction, and treatment of acute and posttraumatic stress in trauma patients: A systematic review. *Journal of Trauma & Acute Care Surgery* 2017;**82**:1158-83

von der Embse N, Barterian J, Segool N. Test Anxiety Interventions for Children and Adolescents: A Systematic Review of Treatment Studies from 2000-2010. *Psychology in the Schools* 2013;**50**:57-71

Wadephul F, Jones C, Jomeen J. The Impact of Antenatal Psychological Group Interventions on Psychological Well-Being: A Systematic Review of the Qualitative and Quantitative Evidence. *Healthcare* 2016;**4**:08

Walczak M, Ollendick T, Ryan S, Esbjorn BH. Does comorbidity predict poorer treatment outcome in pediatric anxiety disorders? An updated 10-year review. *Clinical Psychology Review* 2018;**60**:45-61

Waldron, Casserly, Lisa M, O'Sullivan. Cognitive behavioural therapy for depression and anxiety in adults with acquired brain injury. What works for whom? *Neuropsychological Rehabilitation* 2013;**23**:64-101

Waldron HB, Turner CW. Evidence-based psychosocial treatments for adolescent substance abuse. *Journal of Clinical Child and Adolescent Psychology* 2008;**37**:238-61

Wan Mohd Yunus WMA, Musiat P, Brown JSL. Systematic review of universal and targeted workplace interventions for depression. *Occupational and Environmental Medicine* 2018;**75**:66-75

Wang L, Chang Y, Kennedy SA, Hong PJ, Chow N, Couban RJ*, et al.* Perioperative psychotherapy for persistent post-surgical pain and physical impairment: a meta-analysis of randomised trials. *British Journal of Anaesthesia* 2018;**120**:1304-14

Wang MY, Wang SY, Tsai PS. Cognitive behavioural therapy for primary insomnia: A systematic review. *Journal of Advanced Nursing* 2005;**50**:553-64

Watson H, Bulik C. Update on the treatment of anorexia nervosa: Review of clinical trials, practice guidelines and emerging interventions. *Psychological Medicine* 2013;**43**:2477-500

Weisz JR, McCarty CA, Valeri SM. Effects of Psychotherapy for Depression in Children and Adolescents: A Meta-Analysis. *Psychological Bulletin* 2006;**132**:132-49

Weitlauf AS, McPheeters ML, Peters B, Sathe N, Travis R, Aiello R*, et al. Therapies for Children with Autism Spectrum Disorder: Behavioural Interventions Update*. Rockville (US): Agency for Healthcare Research and Quality; 2014.

Werner-Seidler A, Johnston L, Christensen H. Digitally-delivered cognitive-behavioural therapy for youth insomnia: A systematic review. *Internet Interventions* 2018;**11**:71-8

Weydert JA, Ball TM, Davis MF. Systematic review of treatments for recurrent abdominal pain. *Pediatrics* 2003;**111**:e1-11

Whiting P, Bagnall A, Sowden AJ, Cornell JE, Mulrow CD, Ramírez G*, et al.* Interventions for the treatment and management of chronic fatigue syndrome: a systematic review. *JAMA: Journal of the American Medical Association* 2001;**286**:1360-90

Williams S, Dale J. The effectiveness of treatment for depression/depressive symptoms in adults with cancer: A systematic review. *British Journal of Cancer* 2006;**94**:372-90

Wilson DB, Bouffard LA, Mackenzie DL. A Quantitative Review of Structured, Group-Oriented, Cognitive-Behavioral Programs for Offenders. *Criminal Justice and Behavior* 2005;**32**:172-204

Wolpert M, Dalzell K, Ullman R, Garland L, Cortina M, Hayes D*, et al.* Strategies not accompanied by a mental health professional to address anxiety and depression in children and young people: a scoping review of range and a systematic review of effectiveness. *The Lancet Psychiatry* 2019;**6**:46-60

Wu Y, Lang Z, Zhang H. Efficacy of Cognitive-Behavioral Therapy in Pediatric Obsessive-Compulsive Disorder: A Meta-Analysis. *Medical science monitor* 2016;**22**:1646-53

Yang B, Xu J, Xue Q, Wei T, Xu J, Ye C*, et al.* Non-pharmacological interventions for improving sleep quality in patients on dialysis: systematic review and meta-analysis. *Sleep Medicine Reviews* 2015;**23**:68-82

Yang S, Sajatovic M, Walter BL. Psychosocial interventions for depression and anxiety in Parkinson's disease. *Journal of Geriatric Psychiatry and Neurology* 2012;**25**:113-21

Yatham S, Sivathasan S, Yoon R, Da Silva T, Ravindran AV, Levitt SE. Depression and anxiety disorders in child and adolescent populations in low and middle income countries: A review. *Annals of Global Health* 2017;**83(1)**:87

Ye YY, Chen NK, Chen J, Liu J, Lin L, Liu YZ*, et al.* Internet-based cognitive-behavioural therapy for insomnia (ICBT-i): A meta-analysis of randomised controlled trials. *BMJ Open* 2016;**6(11)**:e010707

Ye YY, Zhang YF, Chen J, Liu J, Li XJ, Liu YZ*, et al.* Internet-based cognitive behavioral therapy for insomnia (ICBT-i) improves comorbid anxiety and depression - A meta-analysis of randomized controlled trials. *Plos One* 2015;**10(11)**:e0142258

Yeh ML, Chung YC, Hsu MYF, Hsu CC. Quantifying psychological distress among cancer patients in interventions and scales: A systematic review topical collection on cancer pain. *Current Pain and Headache Reports* 2014;**18(3)**:399

Ying L, Wu LH, Loke AY. The effects of psychosocial interventions on the mental health, pregnancy rates, and marital function of infertile couples undergoing in vitro fertilization: a systematic review. *Journal of Assisted Reproduction and Genetics* 2016;**33**:689-701

Yohannes AM, Caton S. Management of depression in older people with osteoarthritis: a systematic review. *Aging and Mental Health* 2010;**14**:637-51

Yohannes AM, Junkes-Cunha M, Smith J, Vestbo J. Management of Dyspnea and Anxiety in Chronic Obstructive Pulmonary Disease: A Critical Review. *Journal of the American Medical Directors Association* 2017;**18**:1096.e1-.e17

Yoon IA, Slade K, Fazel S. Outcomes of psychological therapies for prisoners with mental health problems: A systematic review and meta-analysis. *Journal of Consulting and Clinical Psychology* 2017;**85**:783-802

Zantvoord JB, Diehle J, Lindauer RJL. Using neurobiological measures to predict and assess treatment outcome of psychotherapy in posttraumatic stress disorder: Systematic review. *Psychotherapy and Psychosomatics* 2013;**82**:142-51

Zhou SG, Hou YF, Liu D, Zhang XY. Effect of Cognitive Behavioral Therapy Versus Interpersonal Psychotherapy in Patients with Major Depressive Disorder: A Meta-analysis of Randomized Controlled Trials. *Chinese Medical Journal* 2017;**130**:2844-51

Zhou X, Hetrick SE, Cuijpers P, Qin B, Barth J, Whittington CJ*, et al.* Comparative efficacy and acceptability of psychotherapies for depression in children and adolescents: A systematic review and network meta-analysis. *World Psychiatry* 2015;**14**:207-22

Zimmermann G, Favrod J, Trieu VH, Pomini V. The effect of cognitive behavioral treatment on the positive symptoms of schizophrenia spectrum disorders: A meta-analysis. *Schizophrenia Research* 2005;**77**:1-9

Zimmermann P, Muhlig S, Sonntag D, Buhringer G, Wittchen HU. Review on psychotherapeutic interventions for cannabis disorders. *Sucht* 2004;**50**:334-42

e. References of studies excluded due to broad definition of CBT (119)

Alvarez-Jimenez M, Hetrick SE, Gonzalez-Blanch C, Gleeson JF, McGorry PD. Non-pharmacological management of antipsychotic-induced weight gain: Systematic review and meta-analysis of randomised controlled trials. *British Journal of Psychiatry* 2008;**193**:101-7

Amick HR, Gartlehner G, Gaynes BN, Forneris C, Asher GN, Morgan LC*, et al.* Comparative benefits and harms of second generation antidepressants and cognitive behavioral therapies in initial treatment of major depressive disorder: Systematic review and meta-analysis. *BMJ* 2015;**351**:h6019

Andersson G, Nordgren LB, Buhrman M, Carlbring P. Psychological treatments for depression delivered via the internet and supported by a clinician: An update. *Revista de Psicopatologia y Psicologia Clinica* 2014;**19**:217-25

Arnberg A, Ost LG. CBT for children with depressive symptoms: a meta-analysis. *Cognitive Behaviour Therapy* 2014;**43**:275-88

Barlow J, Smailagic N, Huband N, Roloff V, Bennett C. Group-based parent training programmes for improving parental psychosocial health. *Cochrane Database Syst Rev* 2014;**(5)**:CD002020

Barrera TL, Mott JM, Hofstein RF, Teng EJ. A meta-analytic review of exposure in group cognitive behavioral therapy for posttraumatic stress disorder. *Clinical Psychology Review* 2013;**33**:24-32

Battagliese G, Caccetta M, Luppino OI, Baglioni C, Cardi V, Mancini F*, et al.* Cognitive-behavioral therapy for externalizing disorders: A meta-analysis of treatment effectiveness. *Behaviour Research and Therapy* 2015;**75**:60-71

Bennett K, Manassis K, Walter SD, Cheung A, Wilansky-Traynor P, Diaz-Granados N*, et al.* Cognitive behavioral therapy age effects in child and adolescent anxiety: An individual patient data metaanalysis. *Depression and Anxiety* 2013;**30**:829-41

Bighelli I, Huhn M, Schneider-Thoma J, Krause M, Reitmeir C, Wallis S*, et al.* Response rates in patients with schizophrenia and positive symptoms receiving cognitive behavioural therapy: a systematic review and single-group meta-analysis. *BMC Psychiatry* 2018;**18**:380

Bisson J, Andrew M. Psychological therapies for chronic post-traumatic stress disorder (PTSD) in adults. *Cochrane Database Syst Rev* 2013;**(12)**:CD003388

Burns AM, Erickson DH, Brenner CA. Cognitive-behavioral therapy for medication-resistant psychosis: a meta-analytic review. *Psychiatric Services* 2014;**65**:874-80

Buscemi N, Vandermeer B, Friesen C, Bialy L, Tubman M, Ospina M*, et al.* Manifestations and management of chronic insomnia in adults. *Evidence Reports/Technology Assessments, No 125* 2005:135

Chung K-F, Lee C-T, Yeung W-F, Chan M-S, Chung EW-Y, Lin W-L. Sleep hygiene education as a treatment of insomnia: A systematic review and meta-analysis. *Family Practice* 2018;**35**:365-75

Chung KF, Lee CT, Yeung WF, Chan MS, Chung EW, Lin WL. Sleep hygiene education as a treatment of insomnia: a systematic review and meta-analysis. *Family Practice* 2017;**29**:29

Cooper K, Gregory JD, Walker I, Lambe S, Salkovskis PM. Cognitive Behaviour Therapy for Health Anxiety: A Systematic Review and Meta-Analysis. *Behavioural and Cognitive Psychotherapy* 2017;**45**:110-23

Crepaz N, Passin WF, Herbst JH, Rama SM, Malow RM, Purcell DW*, et al.* Meta-analysis of cognitive-behavioral interventions on HIV-positive persons' mental health and immune functioning. *Health Psychology* 2008;**27**:4-14

Cristea IA, Stefan S, Karyotaki E, David D, Hollon SD, Cuijpers P. The effects of cognitive behavioral therapy are not systematically falling: A revision of Johnsen and Friborg (2015). *Psychological Bulletin* 2017;**143**:326-40

Cuijpers P, Cristea IA, Karyotaki E, Reijnders M, Huibers MJH. How effective are cognitive behavior therapies for major depression and anxiety disorders? A meta-analytic update of the evidence. *World Psychiatry* 2016;**15**:245-58

Cuijpers P, Sijbrandij M, Koole S, Huibers M, Berking M, Andersson G. Psychological treatment of generalized anxiety disorder: A meta-analysis. *Clinical Psychology Review* 2014;**34**:130-40

Cuijpers P, Straten A, Andersson G. Internet-administered cognitive behavior therapy for health problems: a systematic review. *Journal of Behavioral Medicine* 2008;**31(2)**:169-77

Cuijpers P, Straten A, Warmerdam L. Are individual and group treatments equally effective in the treatment of depression in adults: a meta-analysis. *European Journal of Psychiatry* 2008;**22**:38-51

Cuijpers P, Straten A, Warmerdam L, Andersson G. Psychotherapy versus the combination of psychotherapy and pharmacotherapy in the treatment of depression: a meta-analysis. *Depression and Anxiety* 2009;**26**:279-88

Cuijpers P, van Straten A, Smit F, Mihalopoulos C, Beekman A. Preventing the onset of depressive disorders: a meta-analytic review of psychological interventions. *American Journal of Psychiatry* 2008;**165**:1272-80

De Los Reyes A. *Identifying evidence-based interventions using the range of possible changes model: A meta-analytic illustration*: Yale University; 2008.

Dickens C, Cherrington A, Adeyemi I, Roughley K, Bower P, Garrett C*, et al.* Characteristics of psychological interventions that improve depression in people with coronary heart disease: a systematic review and meta-regression. *Psychosomatic Medicine* 2013;**75**:211-21

Diehle J, Schmitt K, Daams JG, Boer F, Lindauer RJ. Effects of psychotherapy on trauma-related cognitions in posttraumatic stress disorder: a meta-analysis. *Journal of traumatic stress* 2014;**27**:257-64

Dissanayake RK, Bertouch JV. Psychosocial interventions as adjunct therapy for patients with rheumatoid arthritis: A systematic review. *International Journal of Rheumatic Diseases* 2010;**13**:324-34

Dorrepaal E, Thomaes K, Hoogendoorn AW, Veltman DJ, Draijer N, van Balkom AJ. Evidence-based treatment for adult women with child abuse-related Complex PTSD: a quantitative review. *European Journal of Psychotraumatology* 2014;**5**:23613

Eccleston C, Palermo TM, Fisher E, Law E. Psychological interventions for parents of children and adolescents with chronic illness. *Cochrane Database Syst Rev* 2012;**8**:CD009660

Feng CY, Chu H, Chen CH, Chang YS, Chen TH, Chou YH*, et al.* The effect of cognitive behavioral group therapy for depression: a meta-analysis 2000-2010. *Worldviews on evidence-based nursing / Sigma Theta Tau International, Honor Society of Nursing* 2012;**9**:2-17

Fentz HN, Arendt M, O'Toole MS, Hoffart A, Hougaard E. The mediational role of panic self-efficacy in cognitive behavioral therapy for panic disorder: A systematic review and meta-analysis. *Behaviour Research and Therapy* 2014;**60**:23-33

Filges T, Jorgensen A-MK. Cognitive–Behavioral Therapies for Young People in Outpatient Treatment for Nonopioid Drug Use. *Research on Social Work Practice* 2018;**28**:363-85

Flint J, Cuijpers P, Horder J, Koole S, Munafo M. Is there an excess of significant findings in published studies of psychotherapy for depression? *Psychological Medicine* 2015;**45**:439-46

Fors EA, Bertheussen GF, Thune I, Juvet LK, Elvsaas IK, Oldervoll L*, et al.* Psychosocial interventions as part of breast cancer rehabilitation programs? Results from a systematic review. *Psycho-Oncology* 2011;**20**:909-18

Forti-Buratti MA, Saikia R, Wilkinson EL, Ramchandani PG. Psychological treatments for depression in pre-adolescent children (12 years and younger): systematic review and meta-analysis of randomised controlled trials. *European Child and Adolescent Psychiatry* 2016;**25**:1045-54

Furukawa T, Noma H, Caldwell D, Honyashiki M, Shinohara K, Imai H*, et al.* Waiting list may be a nocebo condition in psychotherapy trials: A contribution from network meta-analysis. *Acta Psychiatrica Scandinavica* 2014;**130**:181-92

Furukawa TA, Watanabe N, Omori IM, Churchill R. Can pill placebo augment cognitive-behavior therapy for panic disorder? *BMC Psychiatry* 2007;**7**:73

Furukawa TA, Weitz ES, Tanaka S, Hollon SD, Hofmann SG, Andersson G*, et al.* Initial severity of depression and efficacy of cognitive-behavioural therapy: Individual-participant data meta-analysis of pill-placebo-controlled trials. *British Journal of Psychiatry* 2017;**210**:190-6

Galsworthy-Francis L, Allan S. Cognitive Behavioural Therapy for anorexia nervosa: A systematic review. *Clinical Psychology Review* 2014;**34**:54-72

Garg S, Garg D, Turin TC, Chowdhury MF. Web-Based Interventions for Chronic Back Pain: A Systematic Review. *Journal of Medical Internet Research* 2016;**18**:e139

Gartlehner G, Gaynes BN, Amick HR, Asher G, Morgan LC, Coker-Schwimmer E*, et al. Nonpharmacological Versus Pharmacological Treatments for Adult Patients with Major Depressive Disorder. Comparative Effectiveness Review No. 161*. Rockville (MD): Agency for Healthcare Research and Quality; 2015.

Ghahramanlou M. *Cognitive behavioral treatment efficacy for anxiety disorders: A meta-analytic review*: Fairleigh Dickinson University; 2003.

Gotzsche PC, Gotzsche PK. Cognitive behavioural therapy halves the risk of repeated suicide attempts: systematic review. *Journal of the Royal Society of Medicine* 2017;**110**:404-10

Gould RL, Coulson MC, Howard RJ. Cognitive behavioral therapy for depression in older people: a meta-analysis and meta-regression of randomized controlled trials. *Journal of the American Geriatrics Society* 2012;**60**:1817-30

Gracie DJ, Irvine AJ, Sood R, Mikocka-Walus A, Hamlin PJ, Ford AC. Effect of psychological therapy on disease activity, psychological comorbidity, and quality of life in inflammatory bowel disease: a systematic review and meta-analysis. *The Lancet Gastroenterology and Hepatology* 2017;**2**:189-99

Haby MM, Donnelly M, Corry J, Vos T. Cognitive behavioural therapy for depression, panic disorder and generalized anxiety disorder: a meta-regression of factors that may predict outcome. *Australian and New Zealand Journal of Psychiatry* 2006;**40**:9-19

Harrington R, Whittaker J, Shoebridge P, Campbell F. Systematic review of efficacy of cognitive behaviour therapies in childhood and adolescent depressive disorder. *British Medical Journal* 1998;**316**:1559-63

Hawton K, Witt KG, Taylor Salisbury TL, Arensman E, Gunnell D, Hazell P*, et al.* Psychosocial interventions for self-harm in adults. *Cochrane Database Syst Rev* 2016;**(5)**:CD012189

Hesser H, Weise C, Westin VZ, Andersson G. A systematic review and meta-analysis of randomized controlled trials of cognitive-behavioral therapy for tinnitus distress. *Clinical Psychology Review* 2011;**31**:545-53

Ho FY, Chan CS, Tang KN. Cognitive-behavioral therapy for sleep disturbances in treating posttraumatic stress disorder symptoms: A meta-analysis of randomized controlled trials. *Clinical Psychology Review* 2016;**43**:90-102

Ho FY, Chung KF, Yeung WF, Ng TH, Kwan KS, Yung KP*, et al.* Self-help cognitive-behavioral therapy for insomnia: a meta-analysis of randomized controlled trials. *Sleep Medicine Reviews* 2015;**19**:17-28

Hockenhull J, Whittington R, Leitner M, Barr W, McGuire J, Cherry M*, et al.* A systematic review of prevention and intervention strategies for populations at high risk of engaging in violent behaviour: update 2002-8. *Health Technology Assessment* 2012;**16**:1-152

Hofmann SG, Smits JA. Cognitive-behavioral therapy for adult anxiety disorders: a meta-analysis of randomized placebo-controlled trials. *Journal of Clinical Psychiatry* 2008;**69**:621-32

Honyashiki M, Furukawa TA, Noma H, Tanaka S, Chen P, Ichikawa K*, et al.* Specificity of CBT for depression: A contribution from multiple treatments meta-analyses. *Cognitive Therapy and Research* 2014;**38**:249-60

Hopkinson MD, Reavell J, Lane DA, Mallikarjun P. Cognitive Behavioral Therapy for Depression, Anxiety, and Stress in Caregivers of Dementia Patients: A Systematic Review and Meta-Analysis. *Gerontologist* 2018;**24**:24

Hunot V, Churchill R, Teixeira V, Silva de Lima M. Psychological therapies for generalised anxiety disorder. *Cochrane Database Syst Rev* 2007;**24(1)**:CD001848

Iniesta-Sepulveda M, Rosa-Alcazar AI, Sanchez-Meca J, Parada-Navas JL, Rosa-Alcazar A. Cognitive-behavioral high parental involvement treatments for pediatric obsessive-compulsive disorder: A meta-analysis. *Journal of Anxiety Disorders* 2017;**49**:53-64

Ishikawa SI, Okajima I, Matsuoka H, Sakano Y. Cognitive behavioural therapy for anxiety disorders in children and adolescents: A meta-analysis. *Child and Adolescent Mental Health* 2007;**12**:164-72

Jassim GA, Whitford DL, Hickey A, Carter B. Psychological interventions for women with non-metastatic breast cancer. *Cochrane Database Syst Rev* 2015;**28(5)**:CD008729

Jauhar S, McKenna PJ, Radua J, Fung E, Salvador R, Laws KR. Cognitive-behavioural therapy for the symptoms of schizophrenia: systematic review and meta-analysis with examination of potential bias. *British Journal of Psychiatry* 2014;**204**:20-9

Jonas DE, Cusack K, Forneris CA, Wilkins TM, Sonis J, Middleton JC*, et al. Psychological and Pharmacological Treatments for Adults With Posttraumatic Stress Disorder (PTSD)*. Rockville (MD): Agency for Healthcare Research and Quality (US); 2013.

Jonsson H, Hougaard E. Group cognitive behavioural therapy for obsessive-compulsive disorder: a systematic review and meta-analysis. *Acta Psychiatrica Scandinavica* 2009;**119**:98-106

Kaddour L, Kishita N, Schaller A. A meta-analysis of low-intensity cognitive behavioral therapy-based interventions for dementia caregivers. *International Psychogeriatrics* 2018;**12**:1-16

Kaltenthaler E, Shackley P, Stevens K, Beverley C, Parry G, Chilcott J. A systematic review and economic evaluation of computerised cognitive behaviour therapy for depression and anxiety. *Health Technology Assessment* 2002;**6(22)**

Kampmann IL, Emmelkamp PM, Morina N. Meta-analysis of technology-assisted interventions for social anxiety disorder. *Journal of Anxiety Disorders* 2016;**42**:71-84

Keles S, Idsoe T. A meta-analysis of group Cognitive Behavioral Therapy (CBT) interventions for adolescents with depression. *Journal of Adolescence* 2018;**67**:129-39

Kishita N, Hammond L, Dietrich CM, Mioshi E. Which interventions work for dementia family carers?: an updated systematic review of randomized controlled trials of carer interventions. *International Psychogeriatrics* 2018;**30**:1679-96

Kwon OY, Ahn HS, Kim HJ, Park KW. Effectiveness of Cognitive Behavioral Therapy for Caregivers of People with Dementia: A Systematic Review and Meta-Analysis. *Journal of Clinical Neurology* 2017;**13**:394-404

Laird KT, Tanner-Smith EE, Russell AC, Hollon SD, Walker LS. Comparative efficacy of psychological therapies for improving mental health and daily functioning in irritable bowel syndrome: A systematic review and meta-analysis. *Clinical Psychology Review* 2017;**51**:142-52

Le LKD, Barendregt JJ, Hay P, Mihalopoulos C. Prevention of eating disorders: A systematic review and meta-analysis. *Clinical Psychology Review* 2017;**53**:46-58

Lee M, Ryoo JH, Chung M, Anderson JG, Rose K, Williams IC. Effective interventions for depressive symptoms among caregivers of people with dementia: A systematic review and meta-analysis. *Dementia (London)* 2019;**12**:1471301218822640

Liu J, Gill NS, Teodorczuk A, Li ZJ, Sun J. The efficacy of cognitive behavioural therapy in somatoform disorders and medically unexplained physical symptoms: A meta-analysis of randomized controlled trials. *Journal of Affective Disorders* 2018;**245**:98-112

Lo K, Waterland J, Todd P, Gupta T, Bearman M, Hassed C*, et al.* Group interventions to promote mental health in health professional education: a systematic review and meta-analysis of randomised controlled trials. *Advances in Health Sciences Education* 2018;**23**:413-47

Lutgens D, Gariepy G, Malla A. Psychological and psychosocial interventions for negative symptoms in psychosis: systematic review and meta-analysis. *British Journal of Psychiatry* 2017;**210**:324-32

Maag JW, Swearer SM, Toland MD. Cognitive-behavioral interventions for depression in children and adolescents: Meta-analysis, promising programs, and implications for school personnel. 2009:235-65

Maddock JE. *Statistical power and effect size in the field of health psychology*: University of Rhode Island; 2000.

Martinez Devesa P, Waddell A, Perera R, Theodoulou M. Cognitive behavioural therapy for tinnitus. *Cochrane Database Syst Rev* 2007;**(1)**:CD005233

Martinez-Devesa P, Perera R, Theodoulou M, Waddell A. Cognitive behavioural therapy for tinnitus. *Cochrane Database Syst Rev* 2010;**8(9)**:CD005233

Mello P, Silva G, Donat J, Kristensen C. An update on the efficacy of cognitive-behavioral therapy, cognitive therapy, and exposure therapy for posttraumatic stress disorder. *International Journal of Psychiatry in Medicine* 2013;**46**:339-57

Menon V, Rajan TM, Kuppili PP, Sarkar S. Cognitive Behavior Therapy for Medically Unexplained Symptoms: A Systematic Review and Meta-analysis of Published Controlled Trials. *Indian Journal of Psychological Medicine* 2017;**39**:399-406

Montero-Marin J, Garcia-Campayo J, Lopez-Montoyo A, Zabaleta-Del-Olmo E, Cuijpers P. Is cognitive-behavioural therapy more effective than relaxation therapy in the treatment of anxiety disorders? A meta-analysis. *Psychological Medicine* 2018;**48(9)**:1427-36

Morina N, Koerssen R, Pollet TV. Interventions for children and adolescents with posttraumatic stress disorder: A meta-analysis of comparative outcome studies. *Clinical Psychology Review* 2016;**47**:41-54

Muresan V, Montgomery GH, David D. Emotional outcomes and mechanisms of change in online cognitive-behavioral interventions: a quantitative meta-analysis of clinical controlled studies. *Journal of Technology in Human Services* 2012;**30**:1-13

Nardi B, Laurenzi S, Di Nicolo M, Bellantuono C. Is the cognitive-behavioral therapy an effective intervention to prevent the postnatal depression? A critical review. *International Journal of Psychiatry in Medicine* 2012;**43**:211-25

Nenova M, Morris L, Paul L, Li Y, Applebaum A, DuHamel K. Psychosocial interventions with cognitive-behavioral components for the treatment of cancer-related traumatic stress symptoms: a review of randomized controlled trials. *Journal of Cognitive Psychotherapy* 2013;**27**:258-84

Newton-Howes G, Wood R. Cognitive behavioural therapy and the psychopathology of schizophrenia: systematic review and meta-analysis. *Psychology and Psychotherapy* 2013;**86**:127-38

Ng QX, Venkatanarayanan N, Kumar L. A Systematic Review and Meta-Analysis of the Efficacy of Cognitive Behavioral Therapy for the Management of Pediatric Migraine. *Headache: The Journal of Head & Face Pain* 2017;**57**:349-62

Nose M, Ballette F, Bighelli I, Turrini G, Purgato M, Tol W*, et al.* Psychosocial interventions for post-Traumatic stress disorder in refugees and asylum seekers resettled in high-income countries: Systematic review and meta-Analysis. *Plos One* 2017;**12(2)**:e0171030

Nuesch E, Hauser W, Bernardy K, Barth J, Juni P. Comparative efficacy of pharmacological and non-pharmacological interventions in fibromyalgia syndrome: network meta-analysis. *Annals of the Rheumatic Diseases* 2013;**72**:955-62

Okajima I, Inoue Y. Efficacy of cognitive behavioral therapy for comorbid insomnia: a meta-analysis. *Sleep and Biological Rhythms* 2018;**16**:21-35

Olatunji BO, Kauffman BY, Meltzer S, Davis ML, Smits JAJ, Powers MB. Cognitive-behavioral therapy for hypochondriasis/health anxiety: A meta-analysis of treatment outcome and moderators. *Behaviour Research and Therapy* 2014;**58**:65-74

Perrot S, Russell IJ. More ubiquitous effects from non-pharmacologic than from pharmacologic treatments for fibromyalgia syndrome: A meta-analysis examining six core symptoms. *European Journal of Pain* 2014;**18**:1067-80

Philipp R, Kriston L, Lanio J, Kuhne F, Harter M, Moritz S*, et al.* Effectiveness of metacognitive interventions for mental disorders in adults-A systematic review and meta-analysis (METACOG). *Clinical Psychology & Psychotherapy* 2018;**19**:19

Phyo AZZ, Demaneuf T, De Livera AM, Jelinek GA, Brown CR, Marck CH*, et al.* The efficacy of psychological interventions for managing fatigue in people with multiple sclerosis: A systematic review and meta-analysis. *Frontiers in Neurology* 2018;**4(9)**:149

Pijnenborg GHM, Van Donkersgoed RJM, David AS, Aleman A. Changes in insight during treatment for psychotic disorders: A meta-analysis. *Schizophrenia Research* 2013;**144**:109-17

Pozza A, Andersson G, Antonelli P, Dettore D. Computer-delivered cognitive-behavioural treatments for obsessive compulsive disorder: preliminary meta-analysis of randomized and non-randomized effectiveness trials. *The Cognitive Behaviour Therapist* 2014;**7**:e16

Pozza A, Dettore D. Drop-out and efficacy of group versus individual cognitive behavioural therapy: What works best for Obsessive-Compulsive Disorder? A systematic review and meta-analysis of direct comparisons. *Psychiatry Research* 2017;**258**:24-36

Reynolds S, Wilson C, Austin J, Hooper L. Effects of psychotherapy for anxiety in children and adolescents: A meta-analytic review. *Clinical Psychology Review* 2012;**32**:251-62

Saddichha S, Al-Desouki M, Lamia A, Linden IA, Krausz M. Online interventions for depression and anxiety - a systematic review. *Health Psychology & Behavioral Medicine* 2014;**2**:841-81

Santoft F, Axelsson E, Ost LG, Hedman-Lagerlof M, Fust J, Hedman-Lagerlof E. Cognitive behaviour therapy for depression in primary care: systematic review and meta-analysis. *Psychological Medicine* 2019;**49(8)**:1266-74

Shinohara K, Honyashiki M, Imai H, Hunot V, Caldwell DM, Davies P*, et al.* Behavioural therapies versus other psychological therapies for depression. *Cochrane Database Syst Rev* 2013;**16(10)**:CD008696

Sijbrandij M, Kunovski I, Cuijpers P. Effectiveness of Internet-Delivered Cognitive Behavioral Therapy for Posttraumatic Stress Disorder: A Systematic Review and Meta-Analysis. *Depression and Anxiety* 2016;**33**:783-91

Smeets KC, Leeijen AAM, van der Molen MJ, Scheepers FE, Buitelaar JK, Rommelse NNJ. Treatment moderators of cognitive behavior therapy to reduce aggressive behavior: a meta-analysis. *European Child and Adolescent Psychiatry* 2015;**24**:255-64

Spielmans GI, Benish SG, Marin C, Bowman WM, Menster M, Wheeler AJ. Specificity of psychological treatments for bulimia nervosa and binge eating disorder? A meta-analysis of direct comparisons. *Clinical Psychology Review* 2013;**33**:460-9

Stafford MR, Jackson H, Mayo-Wilson E, Morrison AP, Kendall T. Early interventions to prevent psychosis: Systematic review and meta-analysis. *BMJ* 2013;**346**:f185

Stephens S, Ford E, Paudyal P, Smith H. Effectiveness of Psychological Interventions for Postnatal Depression in Primary Care: A Meta-Analysis. *Annals of Family Medicine* 2016;**14**:463-72

Tirado-Munoz J, Gilchrist G, Farre M, Hegarty K, Torrens M. The efficacy of cognitive behavioural therapy and advocacy interventions for women who have experienced intimate partner violence: A systematic review and meta-analysis. *Annals of Medicine* 2014;**46**:567-86

van Bronswijk S, Moopen N, Beijers L, Ruhe HG, Peeters F. Effectiveness of psychotherapy for treatment-resistant depression: a meta-analysis and meta-regression. *Psychological Medicine* 2019;**49**:366-79

van den Akker LE, Beckerman H, Collette EH, Eijssen ICJM, Dekker J, de Groot V. Effectiveness of cognitive behavioral therapy for the treatment of fatigue in patients with multiple sclerosis: A systematic review and meta-analysis. *Journal of Psychosomatic Research* 2016;**90**:33-42

Van Der Gaag M, Smit F, Bechdolf A, French P, Linszen DH, Yung AR*, et al.* Preventing a first episode of psychosis: Meta-analysis of randomized controlled prevention trials of 12month and longer-term follow-ups. *Schizophrenia Research* 2013;**149**:56-62

van der Heijden I, Abrahams N, Sinclair D. Psychosocial group interventions to improve psychological well-being in adults living with HIV. *Cochrane Database Syst Rev* 2017;**14(3)**:CD010806

Vugts MAP, Joosen MCW, van der Geer JE, Zedlitz A, Vrijhoef HJM. The effectiveness of various computer-based interventions for patients with chronic pain or functional somatic syndromes: A systematic review and meta-analysis. *PLoS ONE* 2018;**13(5)**:e0196467

Weitz ES, Hollon SD, Twisk J, Van Straten A, Huibers MJH, David D*, et al.* Baseline depression severity as moderator of depression outcomes between cognitive behavioral therapy vs pharmacotherapy: An individual patient data meta-analysis. *JAMA Psychiatry* 2015;**72**:1102-9

Wells MJ, Owen JJ, McCray LW, Bishop LB, Eells TD, Brown GK*, et al.* Computer-Assisted Cognitive-Behavior Therapy for Depression in Primary Care: Systematic Review and Meta-Analysis. *The Primary Care Companion to CNS Disorders* 2018;**20**:01

Wethington HR, Hahn RA, Fuqua-Whitley DS, Sipe TA, Crosby AE, Johnson RL*, et al.* The effectiveness of interventions to reduce psychological harm from traumatic events among children and adolescents. A systematic review. *American Journal of Preventive Medicine* 2008;**35**:287-313

Whittal ML, Agras WS, Gould RA. Bulimia nervosa: a meta-analysis of psychosocial and pharmacological treatments. *Behavior Therapy* 1999;**30**:117-35

Williams ACC, Eccleston C, Morley S. Psychological therapies for the management of chronic pain (excluding headache) in adults. *Cochrane Database Syst Rev* 2012;**(8)**

Young Z, Moghaddam N, Tickle A. The Efficacy of Cognitive Behavioral Therapy for Adults With ADHD: A Systematic Review and Meta-Analysis of Randomized Controlled Trials. *Journal of Attention Disorders* 2016;**22**:22

Zhang M, Huang L, Feng Z, Shao L, Chen L. Effects of cognitive behavioral therapy on quality of life and stress for breast cancer survivors: A meta-analysis. *Minerva Medica* 2017;**108**:84-93

f. References of studies excluded due to not relevant outcomes (125)

Abreu Costa M, D'Alo de Oliveira GS, Tatton-Ramos T, Manfro GG, Salum GA. Anxiety and stress-related disorders and mindfulness-based interventions: A systematic review and multilevel meta-analysis and meta-regression of multiple outcomes. *Mindfulness* 2018;**10**:996-1005

Alammehrjerdi Z, Ezard N, Dolan K. Methamphetamine dependence in methadone treatment services in Iran: the first literature review of a new health concern. *Asian Journal of Psychiatry* 2018;**31**:49-55

Anderson N, Heywood-Everett S, Siddiqi N, Wright J, Meredith J, McMillan D. Faith-adapted psychological therapies for depression and anxiety: Systematic review and meta-analysis. *Journal of Affective Disorders* 2015;**176**:183-96

Applebaum AJ, Breitbart W. Care for the cancer caregiver: a systematic review. *Palliative & Supportive Care* 2013;**11**:231-52

Ashman LL, Duggan L. Interventions for learning disabled sex offenders. *Cochrane Database Syst Rev* 2008;**1**:CD003682

Babcock JC, Green CE, Robie C. Does batterers' treatment work? A meta-analytic review of domestic violence treatment. *Clinical Psychology Review* 2004;**23**:1023-53

Benbow AA, Anderson PL. A meta-analytic examination of attrition in virtual reality exposure therapy for anxiety disorders. *Journal of Anxiety Disorders* 2019;**61**:18-26

Bernecker SL, Coyne AE, Constantino MJ, Ravitz P. For whom does interpersonal psychotherapy work? A systematic review. *Clinical Psychology Review* 2017;**56**:82-93

Bhui K, Aslam RW, Palinski A, McCabe R, Johnson MR, Weich S*, et al.* Interventions designed to improve therapeutic communications between black and minority ethnic people and professionals working in psychiatric services: a systematic review of the evidence for their effectiveness. *Health technology assessment (Winchester, England)* 2015;**19**:vii-xxiv, 1

Brendel KE, Maynard BR. Child–Parent Interventions for Childhood Anxiety Disorders: A Systematic Review and Meta-Analysis. *Research on Social Work Practice* 2014;**24**:287-95

Brooks SJ, Stein DJ. A systematic review of the neural bases of psychotherapy for anxiety and related disorders. *Dialogues in Clinical Neuroscience* 2015;**17**:261-79

Clarke C, Skokauskas N. CBT for adolescent pathological gambling - lessons from adult research. *Irish Journal of Psychological Medicine* 2009;**26**:140-6

Clements KM, Hydery T, Tesell MA, Greenwood BC, Angelini MC. A systematic review of community-based interventions to improve oral chronic disease medication regimen adherence among individuals with substance use disorder. *Drug & Alcohol Dependence* 2018;**188**:141-52

Cole RL. A systematic review of cognitive-behavioural interventions for adolescents with anger-related difficulties. *Educational and Child Psychology* 2008;**25**:27-47

Cristea IA, Huibers MJ, David D, Hollon SD, Andersson G, Cuijpers P. The effects of cognitive behavior therapy for adult depression on dysfunctional thinking: A meta-analysis. *Clinical Psychology Review* 2015;**42**:62-71

Cuijpers P, Smit F, Bohlmeijer E, Hollon SD, Andersson G. Efficacy of cognitive-behavioural therapy and other psychological treatments for adult depression: meta-analytic study of publication bias. *British Journal of Psychiatry* 2010;**196**:173-8

Cuijpers P, Weitz E, Twisk J, Kuehner C, Cristea I, David D*, et al.* Gender as predictor and moderator of outcome in cognitive behavior therapy and pharmacotherapy for adult depression: An "individual patient data" meta-analysis. *Depression and Anxiety* 2014;**31**:941-51

da Silva JA, Siegmund G, Bredemeier J. Crisis interventions in online psychological counseling. *Trends in Psychiatry & Psychotherapy* 2015;**37**:171-82

Darker CD, Sweeney BP, Barry JM, Farrell MF, Donnelly-Swift E. Psychosocial interventions for benzodiazepine harmful use, abuse or dependence. *Cochrane Database Syst Rev* 2015;**5**:CD009652

De Crescenzo F, Ciabattini M, D'Alo GL, De Giorgi R, Del Giovane C, Cassar C*, et al.* Comparative efficacy and acceptability of psychosocial interventions for individuals with cocaine and amphetamine addiction: A systematic review and network meta-analysis. *PLoS Medicine / Public Library of Science* 2018;**15**:e1002715

De Giorgi R, Cassar C, Loreto D'alo G, Ciabattini M, Minozzi S, Economou A*, et al.* Psychosocial interventions in stimulant use disorders: a systematic review and qualitative synthesis of randomized controlled trials. *Rivista di Psichiatria* 2018;**53**:233-55

Dennis JA, Khan O, Ferriter M, Huband N, Powney MJ, Duggan C. Psychological interventions for adults who have sexually offended or are at risk of offending. *Cochrane Database Syst Rev* 2012;**12**:CD007507

Depont F, Berenbaum F, Filippi J, Le Maitre M, Nataf H, Paul C*, et al.* Interventions to improve adherence in patients with immune-mediated inflammatory disorders: A systematic Review. *Plos One* 2015;**10**

Dou C, Rebane J, Bardal S. Interventions to improve benzodiazepine tapering success in the elderly: a systematic review. *Aging & Mental Health* 2018;**23(4)**:411-6

Dumont M, Thériault J, Briand C, Dumais A, Potvin S. Psychosocial approaches for individuals with schizophrenia in correctional and forensic psychiatric settings: a rapid review. *Journal of Forensic Practice* 2018;**20**:152-66

Ebrahim S. Psychotherapy for depression in claimants receiving wage replacement benefits: review of the evidence. *Journal of insurance medicine (New York, NY)* 2014;**44**:53-7

Eskildsen A, Hougaard E, Rosenberg NK. Pre-treatment patient variables as predictors of drop-out and treatment outcome in cognitive behavioural therapy for social phobia: A systematic review. *Nordic Journal of Psychiatry* 2010;**64**:94-105

Evans K, Spiby H, Morrell JC. Non-pharmacological interventions to reduce the symptoms of mild to moderate anxiety in pregnant women. A systematic review and narrative synthesis of women's views on the acceptability of and satisfaction with interventions. *Archives of Women's Mental Health* 2019 Jan 7 [Epub ahead of print]

Fisher H, Gardner F, Montgomery P. Cognitive-behavioural interventions for preventing youth gang involvement for children and young people (7-16). *Cochrane Database Syst Rev* 2008;**16(2)**:CD007008

Fjermestad KW, Haugland BSM, Heiervang E, Ost L. Relationship factors and outcome in child anxiety treatment studies. *Clinical Child Psychology & Psychiatry* 2009;**14**:195-214

Franklin G, Carson AJ, Welch KA. Cognitive behavioural therapy for depression: Systematic review of imaging studies. *Acta Neuropsychiatrica* 2016;**28**:61-74

Gallagher M, McLeod HJ, McMillan TM. A systematic review of recommended modifications of CBT for people with cognitive impairments following brain injury. *Neuropsychological Rehabilitation* 2016;**29(1)**:1-21

Gilchrist G, Munoz JT, Easton CJ. Should we reconsider anger management when addressing physical intimate partner violence perpetration by alcohol abusing males? A systematic review. *Aggression and Violent Behavior* 2015;**25 (Part A)**:124-32

Gilinsky A, Swanson V, Power K. Interventions delivered during antenatal care to reduce alcohol consumption during pregnancy: A systematic review. *Addiction Research & Theory* 2011;**19**:235-50

Ginsburg GS, Kingery JN, Drake KL, Grados MA. Predictors of treatment response in pediatric obsessive-compulsive disorder. *Journal of the American Academy of Child & Adolescent Psychiatry* 2008;**47**:868-78

Gray H, Howe T. Physiotherapists' assessment and management of psychosocial factors (Yellow and Blue Flags) in individuals with back pain. *Physical Therapy Reviews* 2013;**18**:379-94

Hay P, Chinn D, Forbes D, Madden S, Newton R, Sugenor L*, et al.* Royal Australian and New Zealand College of Psychiatrists clinical practice guidelines for the treatment of eating disorders. *Australian and New Zealand Journal of Psychiatry* 2014;**48**:977-1008

Heber E, Ebert DD, Lehr D, Cuijpers P, Berking M, Nobis S*, et al.* The Benefit of Web- and Computer-Based Interventions for Stress: A Systematic Review and Meta-Analysis. *Journal of Medical Internet Research* 2017;**19**:e32

Henry C, Ghaemi SN. Insight in psychosis: A systematic review of treatment interventions. *Psychopathology* 2004;**37**:194-9

Heron-Speirs HA, Harvey ST, Baken DM. Moderators of Psycho-Oncology Therapy Effectiveness: Meta-Analysis of Therapy Characteristics. *Journal of Psychosocial Oncology* 2013;**31**:617-41

Hesser H, Weise C, Rief W, Andersson G. The effect of waiting: A meta-analysis of wait-list control groups in trials for tinnitus distress. *Journal of Psychosomatic Research* 2011;**70**:378-84

Ho BP, Stephenson J, Carter M. Cognitive-behavioural approach for children with autism spectrum disorder: A literature review. *Journal of Intellectual and Developmental Disability* 2015;**40**:213-29

Ho M, Jensen ME, Burrows T, Neve M, Garnett SP, Baur L*, et al.* Best practice dietetic management of overweight and obese children and adolescents: a 2010 update of a systematic review. *JBI Database of Systematic Reviews & Implementation Reports* 2013;**11**:190-293

Hodgkinson B, Evans D, O'Donnell A, Walsh K. Comparing the effectiveness of individual therapy and group therapy in the treatment of depression: systematic review. *Joanna Briggs Institute for Evidence Based Nursing and Midwifery* 1999;**3**

Hogue A, Henderson CE, Becker SJ, Knight DK. Evidence Base on Outpatient Behavioral Treatments for Adolescent Substance Use, 2014-2017: Outcomes, Treatment Delivery, and Promising Horizons. *Journal of Clinical Child & Adolescent Psychology* 2018;**47**:499-526

Honagodu AR, Krishna M, Sundarachar R, Lepping P. Group psychotherapies for depression in persons with HIV: A systematic review. *Indian Journal of Psychiatry* 2013;**55**:323-30

Huguet A, Rao S, McGrath PJ, Wozney L, Wheaton M, Conrod J*, et al.* A Systematic Review of Cognitive Behavioral Therapy and Behavioral Activation Apps for Depression. *Plos One* 2016;**11**:e0154248

Hundt NE, Mignogna J, Underhill C, Cully JA. The relationship between use of cbt skills and depression treatment outcome: a theoretical and methodological review of the literature *Behavior Therapy* 2013;**44(1)**:12-26

Ince P, Haddock G, Tai S. A systematic review of the implementation of recommended psychological interventions for schizophrenia: Rates, barriers, and improvement strategies. *Psychology and Psychotherapy* 2016;**89**:324-50

Jonsson H, Kristensen M, Arendt M. Intensive cognitive behavioural therapy for obsessive-compulsive disorder: A systematic review and meta-analysis. *Journal of Obsessive-Compulsive and Related Disorders* 2015;**6**:83-96

Joyce S, Shand F, Tighe J, Laurent SJ, Bryant RA, Harvey SB. Road to resilience: A systematic review and meta-analysis of resilience training programmes and interventions. *BMJ Open* 2018;**8(6)**:e017858

Kaltenthaler E, Sutcliffe P, Parry G, Beverley C, Rees A, Ferriter M. The acceptability to patients of computerized cognitive behaviour therapy for depression: a systematic review. *Psychological Medicine* 2008;**38(11)**:1521-30

Karyotaki E, Kemmeren L, Riper H, Twisk J, Hoogendoorn A, Kleiboer A*, et al.* Is self-guided internet-based cognitive behavioural therapy (iCBT) harmful? An individual participant data meta-analysis. *Psychological Medicine* 2018;**48(15)**:2456-66

Kazantzis N, Whittington C, Zelencich L, Kyrios M, Norton PJ, Hofmann SG. Quantity and Quality of Homework Compliance: A Meta-Analysis of Relations With Outcome in Cognitive Behavior Therapy. *Behavior Therapy* 2016;**47**:755-72

Khan A, Tansel A, White DL, Kayani WT, Bano S, Lindsay J*, et al.* Efficacy of Psychosocial Interventions in Inducing and Maintaining Alcohol Abstinence in Patients With Chronic Liver Disease: A Systematic Review. *Clinical Gastroenterology and Hepatology* 2016;**14**:191-202.e4

Klimas J, Tobin H, Field CA, O'Gorman CS, Glynn LG, Keenan E*, et al.* Psychosocial interventions to reduce alcohol consumption in concurrent problem alcohol and illicit drug users. *The Cochrane database of systematic reviews* 2014;**12**:CD009269

Koffel E, Bramoweth AD, Ulmer CS. Increasing access to and utilization of cognitive behavioral therapy for insomnia (CBT-I): a narrative review. *Journal of General Internal Medicine* 2018;**33(6)**:955-62

Kregel J, Coppieters I, Depauw R, Malfliet A, Danneels L, Nijs J*, et al.* Does conservative treatment change the brain in patients with chronic musculoskeletal pain? A systematic review. *Pain Physician* 2017;**20**:139-54

Kurtz MM. Neurocognition as a predictor of response to evidence-based psychosocial interventions in schizophrenia: What is the state of the evidence? *Clinical Psychology Review* 2011;**31**:663-72

Lam LT, Lam MK. eHealth Intervention for Problematic Internet Use (PIU). *Current Psychiatry Reports* 2016;**18(12)**:107

Lavielle M, Puyraimond-Zemmour D, Romand X, Gossec L, Senbel E, Pouplin S*, et al.* Methods to improve medication adherence in patients with chronic inflammatory rheumatic diseases: a systematic literature review. *RMD Open* 2018;**4**:e000684

Law EF, Fisher E, Fales J, Noel M, Eccleston C. Systematic review and meta-analysis of parent and family-based interventions for children and adolescents with chronic medical conditions. *Journal of Pediatric Psychology* 2014;**39**:866-86

Lee EB, An W, Levin ME, Twohig MP. An initial meta-analysis of Acceptance and Commitment Therapy for treating substance use disorders. *Drug & Alcohol Dependence* 2015;**155**:1-7

Linardon J, Hindle A, Brennan L. Dropout from cognitive-behavioral therapy for eating disorders: A meta-analysis of randomized, controlled trials. *International Journal of Eating Disorders* 2018;**51(5)**:381-91

Linardon J, Piedad Garcia X, Brennan L. Predictors, Moderators, and Mediators of Treatment Outcome Following Manualised Cognitive-Behavioural Therapy for Eating Disorders: A Systematic Review. *European Eating Disorders Review* 2017;**25**:3-12

Lundkvist-Houndoumadi I, Hougaard E, Thastum M. Pre-treatment child and family characteristics as predictors of outcome in cognitive behavioural therapy for youth anxiety disorders. *Nordic Journal of Psychiatry* 2014;**68**:524-35

Markel C. *Child-focused psychosocial interventions for children and adolescents with attention-deficit hyperactivity disorder (ADHD): A systematic review and meta-analysis*: University of Toronto, Canada; 2018.

Martire LM. The 'relative' efficacy of involving family in psychosocial interventions for chronic illness: are there added benefits to patients and family members? *Families, Systems & Health: The Journal of Collaborative Family HealthCare* 2005;**23**:312-28

Maseroli E, Scavello I, Rastrelli G, Limoncin E, Cipriani S, Jannini E, *et al.* Outcome of medical and psychosexual interventions for vaginismus: A systematic review and meta-analysis. *Journal of Sexual Medicine* 2018;**15(12)**:1752-64

Matteson ML, Russell C. Interventions to improve hemodialysis adherence: A systematic review of randomized-controlled trials. *Hemodialysis International* 2010;**14**:370-82

Matthews EE, Arnedt JT, McCarthy MS, Cuddihy LJ, Aloia MS. Adherence to cognitive behavioral therapy for insomnia: a systematic review. *Sleep Medicine Reviews* 2013;**17(6)**:453-64

Mbuagbaw L, Sivaramalingam B, Navarro T, Hobson N, Keepanasseril A, Wilczynski NJ, *et al.* Interventions for Enhancing Adherence to Antiretroviral Therapy (ART): A Systematic Review of High Quality Studies. *AIDS Patient Care & STDs* 2015;**29**:248-66

McLean SM, Burton M, Bradley L, Littlewood C. Interventions for enhancing adherence with physiotherapy: A systematic review. *Manual Therapy* 2010;**15**:514-21

Melendez-Torres GJ, Bonell C. Systematic review of cognitive behavioural interventions for HIV risk reduction in substance-using men who have sex with men. *International Journal of STD and AIDS* 2014;**25**:627-35

Montgomery EC, Kunik ME, Wilson N, Stanley MA, Weiss B. Can paraprofessionals deliver cognitive-behavioral therapy to treat anxiety and depressive symptoms? *Bulletin of the Menninger Clinic* 2010;**74(1)**:45-62

Mukhtar F, Oei TP. A review on assessment and treatment for depression in malaysia. *Depression Research and Treatment* 2011;**2011**:123642

Naeem F, Farooq S, Kingdon D. Cognitive behavioural therapy (brief versus standard duration) for schizophrenia. *Cochrane Database of Systematic Reviews* 2015;**21(10)**:CD010646

Nascimento SS, Oliveira LR, DeSantana JM. Correlations between brain changes and pain management after cognitive and meditative therapies: A systematic review of neuroimaging studies. *Complementary Therapies in Medicine* 2018;**39**:137-45

Nesset MB, Lara-Cabrera ML, Dalsbo TK, Pedersen SA, Bjorngaard JH, Palmstierna T. Cognitive behavioural group therapy for male perpetrators of intimate partner violence: a systematic review. *BMC Psychiatry* 2019;**19**:11

Nieuwenhuijsen K, Bültmann U, Neumeyer-Gromen A, Verhoeven AC, Verbeek JH, Feltz-Cornelis CM. Interventions to improve return to work in depressed people. *Cochrane Database Syst Rev* 2014;**3(12)**:CD006237

O'Keeffe J, Conway R, McGuire B. A systematic review examining factors predicting favourable outcome in cognitive behavioural interventions for psychosis. *Schizophrenia Research* 2017;**183**:22-30

Ougrin D, Tranah T, Stahl D, Moran P, Asarnow JR. Therapeutic Interventions for Suicide Attempts and Self-Harm in Adolescents: Systematic Review and Meta-Analysis. *Journal of the American Academy of Child & Adolescent Psychiatry* 2015;**54**:97-107

Brazier J, Tumur I, Holmes M, Ferriter M, Parry G, Dent-Brown K*, et al.* Psychological therapies including dialectical behaviour therapy for borderline personality disorder: a systematic review and preliminary economic evaluation. *Health Technology Assessment* 2006;**10**:iii-131

Perkins DO. Predictors of noncompliance in patients with schizophrenia. *The Journal of clinical psychiatry* 2002;**63**:1121-8

Petry NM, Armentano C. Prevalence, assessment, and treatment of pathological gambling: A review. *Psychiatric Services* 1999;**50**:1021-7

Pihlaja S, Stenberg JH, Joutsenniemi K, Mehik H, Ritola V, Joffe G. Therapeutic alliance in guided internet therapy programs for depression and anxiety disorders - A systematic review. *Internet Interventions* 2018;**11**:1-10

Pompoli A, Furukawa TA, Efthimiou O, Imai H, Tajika A, Salanti G. Dismantling cognitive-behaviour therapy for panic disorder: a systematic review and component network meta-analysis. *Psychological Medicine* 2018;**48(12)**:1945-53

Porter E, Chambless DL. A systematic review of predictors and moderators of improvement in cognitive-behavioral therapy for panic disorder and agoraphobia. *Clinical Psychology Review* 2015;**42**:179-92

Porto PR, Oliveira L, Mari J, Volchan E, Figueira I, Ventura P. Does cognitive behavioral therapy change the brain? A systematic review of neuroimaging in anxiety disorders. *Journal of Neuropsychiatry and Clinical Neurosciences* 2009;**21**:114-25

Potier F, Degryse JM, de Saint-Hubert M. Impact of caregiving for older people and pro-inflammatory biomarkers among caregivers: a systematic review. *Aging Clinical and Experimental Research* 2018;**30**:119-32

Powers MB, Vedel E, Emmelkamp PMG. Behavioral couples therapy (BCT) for alcohol and drug use disorders: A meta-analysis. *Clinical Psychology Review* 2008;**28**:952-62

Rapley HA, Loades ME. A systematic review exploring therapist competence, adherence, and therapy outcomes in individual CBT for children and young people. *Psychotherapy Research* 2018;**29(8)**:1110-019

Rathbun AM, Reed GW, Harrold LR. The temporal relationship between depression and rheumatoid arthritis disease activity, treatment persistence and response: A systematic review. *Rheumatology (United Kingdom)* 2013;**52(10)**:1785-94

Rhodes RE, Fiala B. Building motivation and sustainability into the prescription and recommendations for physical activity and exercise therapy: the evidence. *Physiotherapy Theory & Practice* 2009;**25**:424-41

Riblet NBV, Shiner B, Young-Xu Y, Watts BV. Strategies to prevent death by suicide: Meta-analysis of randomised controlled trials. *British Journal of Psychiatry* 2017;**210**:396-402

Rohden AI, Benchaya MC, Camargo RS, Moreira TDC, Barros HMT, Ferigolo M. Dropout Prevalence and Associated Factors in Randomized Clinical Trials of Adolescents Treated for Depression: Systematic Review and Meta-analysis. *Clinical Therapeutics* 2017;**39**:971-92.e4

Room J, Hannink E, Dawes H, Barker K. What interventions are used to improve exercise adherence in older people and what behavioural techniques are they based on? A systematic review. *BMJ Open* 2017;**7**:e019221

Rudge S, Feigenbaum JD, Fonagy P. Mechanisms of change in dialectical behaviour therapy and cognitive behaviour therapy for borderline personality disorder: a critical review of the literature. *Journal of Mental Health* 2017 May 8:1-11

Rueda S, Park-Wyllie LY, Bayoumi A, Tynan A, Antoniou T, Rourke S*, et al.* Patient support and education for promoting adherence to highly active antiretroviral therapy for HIV/AIDS. *Cochrane Database Syst Rev* 2006;**19(3)**:CD001442

Sajatovic M, Davies M, Hrouda DR. Enhancement of treatment adherence among patients with bipolar disorder. *Psychiatric Services* 2004;**55**:264-9

Schaafsma F, Schonstein E, Whelan KM, Ulvestad E, Kenny DT, Verbeek JH. Physical conditioning programs for improving work outcomes in workers with back pain. *Cochrane Database Syst Rev* 2010;**20(1)**:CD001822

Schaafsma FG, Whelan K, van der Beek AJ, van der Es-Lambeek LC, Ojajarvi A, Verbeek JH. Physical conditioning as part of a return to work strategy to reduce sickness absence for workers with back pain. *Cochrane Database of Systematic Reviews* 2013;**30(8)**

Schutze R, Rees C, Smith A, Slater H, Campbell JM, O'Sullivan P. How Can We Best Reduce Pain Catastrophizing in Adults With Chronic Noncancer Pain? A Systematic Review and Meta-Analysis. *Journal of Pain* 2018;**19**:233-56

Simon W. Follow-up psychotherapy outcome of patients with dependent, avoidant and obsessive-compulsive personality disorders: A meta-analytic review. *International Journal of Psychiatry in Clinical Practice* 2009;**13**:153-65

Smits JA, Berry AC, Tart CD, Powers MB. The efficacy of cognitive-behavioral interventions for reducing anxiety sensitivity: a meta-analytic review. . *Behaviour Research and Therapy* 2008;**46(9)**:1047-54

Smits JAJ, Julian K, Rosenfield D, Powers MB. Threat reappraisal as a mediator of symptom change in cognitive-behavioral treatment of anxiety disorders: A systematic review. *Journal of Consulting and Clinical Psychology* 2012;**80**:624-35

Spinhoven P, Klein N, Kennis M, Cramer AOJ, Siegle G, Cuijpers P*, et al.* The effects of cognitive-behavior therapy for depression on repetitive negative thinking: A meta-analysis. *Behaviour Research & Therapy* 2018;**106**:71-85

Storebo OJ, Skoog M, Damm D, Thomsen PH, Simonsen E, Gluud C. Social skills training for Attention Deficit Hyperactivity Disorder (ADHD) in children aged 5 to 18 years. *Cochrane Database Syst Rev* 2011;**7(12)**:CD008223

Stott J, Charlesworth G, Scior K. Measures of readiness for cognitive behavioural therapy in people with intellectual disability: A systematic review. *Research in Developmental Disabilities* 2017;**60**:37-51

Surley L, Dagnan D. A review of the frequency and nature of adaptations to cognitive behavioural therapy for adults with intellectual disabilities. *Journal of Applied Research in Intellectual Disabilities* 2018;**32(2)**:219-37

Thulin U, Svirsky L, Serlachius E, Andersson G, Ost LG. The effect of parent involvement in the treatment of anxiety disorders in children: a meta-analysis. *Cognitive Behaviour Therapy* 2014;**43**:185-200

Thurgood SL, McNeill A, Clark-Carter D, Brose LS. A systematic review of smoking cessation interventions for adults in substance abuse treatment or recovery. *Nicotine and Tobacco Research* 2016;**18**:993-1001

Turner C, O'Gorman B, Nair A, O'Kearney R. Moderators and predictors of response to cognitive behaviour therapy for pediatric obsessive-compulsive disorder: A systematic review. *Psychiatry Research* 2018;**261**:50-60

Vallury KD, Jones M, Oosterbroek C. Computerized Cognitive Behavior Therapy for Anxiety and Depression in Rural Areas: A Systematic Review. *Journal of Medical Internet Research* 2015;**17**:e139

Van Ballegooijen W, Cuijpers P, Van Straten A, Karyotaki E, Andersson G, Smit JH, *et al.* Adherence to internet-based and face-to-face cognitive behavioural therapy for depression: A meta-analysis. *Plos One* 2014;**9(7)**:e100674

van der Put CE, Assink M, Gubbels J, Boekhout van Solinge NF. Identifying Effective Components of Child Maltreatment Interventions: A Meta-analysis. *Clinical Child & Family Psychology Review* 2018;**21**:171-202

Vazquez Rivera S, Gomez Magarinos S, Gonzalez-Blanch C. Effects on the brain of effective psychological treatments for anxiety disorders: a systematic review. . *Actas Espanolas de Psiquiatria* 2010;**(4)38**:239-48

Vigerland S, Lenhard F, Bonnert M, Lalouni M, Hedman E, Ahlen J, *et al.* Internet-delivered cognitive behavior therapy for children and adolescents: A systematic review and meta-analysis. *Clinical Psychology Review* 2016;**50**:1-10

Vujanovic AA, Farris SG, Bartlett BA, Lyons RC, Haller M, Colvonen PJ, *et al.* Anxiety sensitivity in the association between posttraumatic stress and substance use disorders: A systematic review. *Clinical Psychology Review* 2018;**62**:37-55

Waller R, Gilbody S. Barriers to the uptake of computerized cognitive behavioural therapy: A systematic review of the quantitative and qualitative evidence. *Psychological Medicine* 2009;**39**:705-12

Williamson JPA. *Assessing the suitability of cognitive-behavioural therapy for specialised client populations and clinical practice reports*: University of Birmingham; 2011.

Windgassen S, Moss-Morris R, Chilcot J, Sibelli A, Goldsmith K, Chalder T. The journey between brain and gut: A systematic review of psychological mechanisms of treatment effect in irritable bowel syndrome. *British Journal of Health Psychology* 2017;**22**:701-36

Windsor LC, Jemal A, Alessi EJ. Cognitive behavioral therapy: A meta-analysis of race and substance use outcomes. *Cultural Diversity and Ethnic Minority Psychology* 2015;**21**:300-13

Wray TB, Grin B, Dorfman L, Glynn TR, Kahler CW, Marshall BD, *et al.* Systematic review of interventions to reduce problematic alcohol use in men who have sex with men. *Drug and Alcohol Review* 2016;**35**:148-57

Xu Z, Huang F, Kosters M, Rusch N. Challenging mental health related stigma in China: Systematic review and meta-analysis. II. Interventions among people with mental illness. *Psychiatry Research* 2017;**255**:457-64

g. References of studies excluded as economic evaluation only (13)

Bereza BG, Machado M, Einarson TR. Systematic review and quality assessment of economic evaluations and quality-of-life studies related to generalized anxiety disorder. *Clinical Therapeutics* 2009;**31**:1279-308.

Bosmans JE, van Schaik DJF, de Bruijne MC, van Hout HPJ, van Marwijk HWJ, van Tulder MW, *et al.* Are psychological treatments for depression in primary care cost-effective? *Journal of Mental Health Policy and Economics* 2008;**11**:3-15.

Dieng M, Cust AE, Kasparian NA, Mann GJ, Morton RL. Economic evaluations of psychosocial interventions in cancer: a systematic review. *Psycho-Oncology* 2016;**25**:1380-92.

Gajic-Veljanoski O, Sanyal C, McMartin K, Xie X, Walter M, Higgins C, *et al.* Economic evaluations of commonly used structured psychotherapies for major depressive disorder and generalized anxiety disorder: A systematic review. *Canadian Psychology/Psychologie canadienne* 2018;**59**:301-14.

Heuzenroeder L, Donnelly M, Haby MM, Mihalopoulos C, Rossell R, Carter R, *et al.* Cost-effectiveness of psychological and pharmacological interventions for generalized anxiety disorder and panic disorder. *Australian & New Zealand Journal of Psychiatry* 2004;**38**:602-12.

Karyotaki E, Tordrup D, Buntrock C, Bertollini R, Cuijpers P. Economic evidence for the clinical management of major depressive disorder: A systematic review and quality appraisal of economic evaluations alongside randomised controlled trials. *Epidemiology and Psychiatric Sciences* 2017;**26**:501-16.

Konnopka A, Schaefert R, Heinrich S, Kaufmann C, Luppa M, Herzog W*, et al.* Economics of medically unexplained symptoms: A systematic review of the literature. *Psychotherapy and Psychosomatics* 2012;**81**:265-75.

Lin CWC, Haas M, Maher CG, MacHado LAC, Van Tulder MW. Cost-effectiveness of guideline-endorsed treatments for low back pain: A systematic review. *European Spine Journal* 2011;**20**:1024-38.

Mavranezouli I, Mayo-Wilson E, Dias S, Kew K, Clark DM, Ades AE*, et al.* The cost effectiveness of psychological and pharmacological interventions for social anxiety disorder: A model-based economic analysis. *Plos One* 2015;**10**.

Ophuis RH, Lokkerbol J, Heemskerk SCM, van Balkom AJLM, Hiligsmann M, Evers SMAA. Cost-effectiveness of interventions for treating anxiety disorders: A systematic review. *Journal of Affective Disorders* 2017;**210**:1-13.

Sanyal C, Stolee P, Juzwishin D, Husereau D. Economic evaluations of eHealth technologies: A systematic review. *Plos One* 2018;**13**.

Stevens M, Roberts H, Shiell A. Research review: economic evidence for interventions in children's social care: revisiting the What Works for Children project. *Child & Family Social Work* 2010;**15**:145-54.

van der Velde G, Yu H, Paulden M, Cote P, Varatharajan S, Shearer HM*, et al.* Which interventions are cost-effective for the management of whiplash-associated and neck pain-associated disorders? A systematic review of the health economic literature by the Ontario Protocol for Traffic Injury Management (OPTIMa) Collaboration. *Spine Journal* 2016;**16**:1582-97.

g. References of studies excluded as abstracts/protocol/erratum (278)

Adamus C, Pfammatter M. The effects of subjective recovery-oriented psychological interventions on schizophrenic disorders-a systematic review. *European Archives of Psychiatry and Clinical Neuroscience* 2017;**267 (Supple. 1)**:S53

Addington J, Devoe D. Meta-analytic review of treatment options for CHR youth. *Early Intervention in Psychiatry* 2018;**12 (Supple. 1)**:10

Aljudaibi S, Duane B. Non-pharmacological pain relief during orthodontic treatment. *Evidence-based dentistry* 2018;**19**:48-9

Alwani S. Promoting mothers' psychological health after fetal loss maternal mortality and morbidity is alarming and need extensive interventions. *BJOG: An International Journal of Obstetrics and Gynaecology* 2016;**123 (Supple. 2)**:110

Anagnostopoulou N, Kyriakopoulos M, Alba A. Psychological interventions in early onset psychosis: A systematic review. *European Psychiatry* 2018;**48 (Supple. 1)**:S193

Andrews L. Systematic review summary - Psychosocial interventions to improve quality of life and emotional well-being for recently diagnosed cancer patients. *Singapore Nursing Journal* 2013;**40**:47-9

Angerer P, Li J. Evaluation of short-term and long-term effects of work stress interventions on CVD risk factors: Focusing on the individual level. *European Journal of Preventive Cardiology* 2017;**24 (2S)**:13

Anonymous. Useful treatments for fibromyalgia syndrome. *Journal of Family Practice* 2005;**54**:105
Anonymous. Cognitive behavioral therapy helps ease tinnitus discomforts. *Harvard Women's Health Watch* 2007;**14**:2

Anonymous. "Computerised cognitive-behavioural therapy for depression: systematic review": Erratum. *British Journal of Psychiatry* 2008;**193**:346

Anonymous. Cognitive behavioral therapy and psychoeducation reduce relapse in bipolar disorder. *Journal of the National Medical Association* 2008;**100**:1108-9

Anonymous. Early treatment for PTSD. *Journal of the National Medical Association* 2009;**101**:742

Anonymous. "Effect of Cognitive-Behavioral Therapy for Anxiety Disorders on Quality of Life: A Meta-Analysis": Erratum to Stefan G. Hofmann, Jade Q. Wu, and Hannah Boettcher (2014). *Journal of Consulting and Clinical Psychology* 2014;**82**:1228

Anonymous. "The Effects of Cognitive Behavioral Therapy as an Anti-Depressive Treatment is Falling: A Meta-Analysis": Correction to Johnsen and Friborg (2015). *Psychological Bulletin* 2016;**142**:290

Anonymous. Depression severity does not moderate differences between medication and CBT. *Brown University Psychopharmacology Update* 2016;**27**:4

Anonymous. CBT results overstated. *Therapy Today* 2016: 5.

Anonymous. "Meta-analysis of cognitive-behavioral treatments for adult ADHD": Correction to Knouse, Teller, and Brooks (2017). *Journal of Consulting & Clinical Psychology* 2017;**85**:882

Anonymous. "Meta-analysis of cognitive-behavioral treatments for adult ADHD": Erratum. *Journal of Consulting and Clinical Psychology* 2017;**85**:882

Arts M, Petrykiv S, Fennema J, De Jonge L. The role of psychiatry in the approach of neurocardiogenic syncope. *European Psychiatry* 2018;**48 (Supple. 1)**:S218-S9

Aydin C, Tibbo P, Ursuliak Z. Psychosocial interventions to reduce cannabis use in the early psychosis population. *Early Intervention in Psychiatry* 2012;**6 (Supple.1)**:115

Baandrup L, Rasmussen JO, Klokker L, Austin S, Bjornshave T, Bliksted VF*, et al.* "Treatment of adult patients with schizophrenia and complex mental health needs-A national clinical guideline": Corrigendum. *Nordic Journal of Psychiatry* 2017;**71**:163

Baglioni C, Hertenstein E, Bostanova Z, Rucker G, Riemann D, Feige B. A systematic review and network meta-analysis of complementary and alternative interventions for insomnia. *Journal of Sleep Research* 2018;**27 (Supple. 1)**:172

Ballesio A, Aquino M, Feige B, Johann A, Kyle SD, Spiegelhalder K*, et al.* The impact of cognitive behavioral therapy for insomnia on fatigue symptoms: A systematic examination of randomized controlled trials. *Sleep* 2016;**39 (Supple.)**:A211

Ballesio A, Aquino MRJ, Feige B, Johann A, Kyle SD, Spiegelhalder K*, et al.* Network meta-analysis on the effectiveness of cognitive behavioral therapies for insomnia on daytime depression and fatigue. *Journal of Sleep Research* 2016;**25 (Supple. 1)**:90-1

Barbato A. Effective strategies for health information, self-help and psychoeducation in bipolar disorder. *European Psychiatry* 2011;**26 (Supple. 1)**:2214

Barber B, Zhang H, Mitchell N, O'Connell DA, Harris JR, Seikaly H. Optimal interventions for depression after diagnosis of head and neck cancer: A systematic review. *Otolaryngology - Head and Neck Surgery* 2014;**151 (1S)**:P174

Barrett D. Systematic review summary - Media-delivered cognitive behavioural therapy and behavioural therapy (self-help) for anxiety disorders in adults. *Singapore Nursing Journal* 2015;**42**:39-40

Basu A, Andrews G. Bandelow revisited: A conventional analysis of a meta-analysis of anxiety disorder treatments. *Australian and New Zealand Journal of Psychiatry* 2018;**52 (1S)**:128

Basu A, Andrews G, Cuijpers P, Craske M, McEvoy P, English C*, et al.* Computer therapy for the anxiety and depression disorders is effective, acceptable and practical healthcare: An updated meta-analysis. *Australian and New Zealand Journal of Psychiatry* 2017;**51 (S1)**:117

Bei B, Wiley JF, Trinder J, Member R. Beyond the mean: A systematic review on the correlates of daily sleep variability. *Sleep* 2015;**(1)**:A89

Belleville G, Cousineau H, Levrier K, St-Pierre-Delorme ME. Does CBT-I decrease concomitant anxiety? A meta-analytic review. *Sleep Medicine* 2011;**12 (Supple.1)**:S61

Benz F, Knoop T, Ballesio A, Bacaro V, Johann A, Rucker G*, et al.* The efficacy of cognitive and behavior therapies for insomnia on daytime symptoms: A systematic review and network meta-analysis. *Journal of Sleep Research* 2018;**27 (Supple. 1)**:289

Bertolin-Guillen JM, Bertolin-Colilla M. P.2.a.024 Effectiveness of mindfulness-based therapies as an alternative or adjuvant of antidepressants in the treatment of depression. *European Neuropsychopharmacology* 2011;**21 (Supple. 3)**:S367-S8

Bhattacharya R, Kelley G, Bhattacharjee S. Long-term follow-up effects of computerized or internet-based cognitive behavioral therapy for depression and anxiety: A metaanalysis. *Value in Health* 2012;**15 (4)**:A82

Bighelli I, Leucht S. Psychological interventions for positive symptoms in schizophrenia: A network meta-analysis. *Schizophrenia Bulletin* 2018;**44 (Supple. 1)**:S305

Blodgett JC, Maisel NC, Fuh IL, Wilbourne PL, Finney JW. How effective is continuing care for substance use disorders? A metaanalytic review. *Alcoholism: Clinical and Experimental Research* 2012;**36 (S1)**:156A

Bos T. The efficacy of CBT-I on insomnia and depressive symptoms in comorbid adult patients: A systematic review and meta-analysis. *Sleep* 2018;**41 (Supple. 1)**:A166-A7

Bougea A, Spantideas N, Despoti A, Belegri S, Kleisarhakis M, Chrousos G. Proposed practical recommendations of stress management for headaches in children and adolescents. *European Journal of Neurology* 2018;**25 (Supple. 2)**:160

Boyle JT, Muench A, Gencarelli A, Khader W, Perlis ML. How does intensive sleep retraining (ISR) compare to CBT-I? *Sleep* 2018;**41 (Supple. 1)**:A155-A6

Bradley R, Greene J, Russ E, Dutra L, Westen D. "A Multidimensional Meta-Analysis of Psychotherapy for PTSD": Correction. *The American journal of psychiatry* 2006;**163**:330

British Association for Counselling and Psychotherapy. CBT in schools: a new systematic review. *Therapy Today* 2009;**20(9)**:52

Brotherton H, Hallmark C, Rogers H. The impact of stress management on immune response in breast cancer patients: A systematic review and meta-analysis of lymphocyte outcomes shortly after intervention. *Psycho-Oncology* 2014;**23 (Supple. 3)**:124

Brown RF, Subnis U, Starkweather A, McCain N. The effect of psychosocial interventions for patients with cancer on psychoneuroimmunologic outcomes: A systematic review. *Asia-Pacific Journal of Clinical Oncology* 2012;**8 (Supple. 3)**:145

Bryden PA, Caldwell DM, Welton N, Churchill R, Baxter H, Lewis G*, et al.* A network meta-analysis of the relative efficacy of pharmacological and psychological interventions in adults with obsessive compulsive disorder. *Value in Health* 2014;**17 (7)**:A454-A5

Bucur M, Whale R. Systematic review of randomised interventions for patients at high risk of developing psychosis: 2012 update. *Early Intervention in Psychiatry* 2012;**6 (Supple.1)**:123

Carpenter JS. Nonhormonal Management of Menopause-Associated Vasomotor Symptoms: 2015 NAMS position statement. *Menopause* 2016;**23 (12)**:1365

Carson J. Mindful pain management. *Journal of Alternative and Complementary Medicine* 2016;**22 (6)**:A71

Carson K, Jayasinghe H, Ali A, Singh K, Peters M, Esterman A*, et al.* TO 012. Culturally-tailored interventions for smoking cessation in indigenous populations: A cochrane systematic review and meta-analysis. *Respirology* 2015;**20 (Supple. 2)**:17

Carson KV, Brinn MP, Peters M, Veale A, Esterman AJ, Smith BJ. Interventions for tobacco use cessation in indigenous populations: A cochrane meta-analysis. *American Journal of Respiratory and Critical Care Medicine* 2014;**189**:A1086

Castelein S, Knegtering H. Treatment of negative symptoms: Which psychosocial interventions are effective? *Schizophrenia Bulletin* 2011;**37 (Supple. 1)**:261

Cattelani R, Zettin M, Zoccolotti P. Erratum to: "Rehabilitation treatments for adults with behavioral and psychosocial disorders following acquired brain injury: A systematic review.". *Neuropsychology review* 2011;**21**:224

Cheung JMY, Jarrin DC, Ballot O, Bharwani A, Morin CM. A systematic review of adaptive cognitive behavioral therapy for insomnia implemented in primary care settings. *Sleep Medicine* 2017;**40 (Supple. 1)**:e59

Chow KM, CW HC, Chan JC. Effects of psychoeducational interventions on sexual functioning, quality of life and psychological outcomes in patients with gynaecological cancer: A systematic review. *JBI Library of Systematic Reviews* 2012;**10**:4077-164

Chung P, Khan F. Traumatic brain injury (TBI) diagnosis and treatment: A systematic review and update. *Brain Injury* 2014;**28 (5-6)**:744-5

Chvatalova B. A comparison of the effectiveness of various psychotherapeutic approaches to first-episode psychosis in terms of recovery: A systematic review. *Early Intervention in Psychiatry* 2018;**12 (Supple. 1)**:137

Coffey BJ. 39.3 Update on pharmacological treatments in tics and tourette's disorder. *Journal of the American Academy of Child and Adolescent Psychiatry* 2016;**55 (10)**:S61

Costa A, Melina F, Sansalone A, Iannacchero R. P004. Evidence based psychological treatments in pain management: A review of controlled and randomized trials about chronic headache, neuropathic pain and fibromyalgia. *Journal of Headache and Pain* 2015;**16**

Creasey N, Benger J, Wright I, Lyttle M. 0589 Non-pharmacological interventions to reduce psychological sequalae of mild traumatic brain injury in adults and children: A systematic review. *Brain Injury* 2016;**30 (5-6)**:709-10

Creasey NJ, Lyttle M. Psychological interventions for patients of all ages with mild traumatic brain injury: A systematic review. *Archives of Disease in Childhood* 2016;**101 (Supple. 1)**:A138-A9

Creed F, Rizzo M. W07-04-UK nice guidelines for depression in people with a chronic physical illness. *European Psychiatry Conference: 19th European Congress of Psychiatry, EPA* 2011;**26 (Supple. 1)**:2212

Cullen K, Irvin E, Van Eerd D, Saunders R. Preventing work disability in workers with depression; a systematic review. *Occupational and Environmental Medicine* 2018;**75 (Supple. 2)**:A589

Dahdah M, Driver S, Shafi S, Callender L, Collinsworth A, Brown R*, et al.* Development of evidence-based treatment guidelines for TBI rehabilitation care: The cognitive-behavioral treatment ARM. *Archives of Physical Medicine and Rehabilitation* 2016;**97 (10)**:e100-e1

Dancet E, Vermeulen N, Boivin J, Gameiro S. The effectiveness of psychosocial interventions that can be delivered by all staff members: A systematic review. *Human Reproduction* 2015;**30 (Supple. 1)**:i61

De Groot V, Beckerman H. Rehabilitation to treat MS-related fatigue: the TREFAMS research programme. *Multiple Sclerosis* 2016;**22 (Supple. 3)**:40-1

De Rooij A, Roorda L, Otten R, Dekker J, Steultjens M. Predictors of multidisciplinary rehabilitation outcome in fibromyalgia: A systematic review. *Physiotherapy* 2011;**97 (Supple. 1)**:eS269-eS70

De Vera MA, Galo J. FRI0212 What are the effects of interventions targetting medication adherence in rheumatic diseases: A systematic review. *Annals of the Rheumatic Diseases* 2014;**73 (Supple. 2)**:459

Desai P, Urosevic S, Butler M. Psychosocial and other non-drug treatments for bipolar disorder in adults: A systematic review. *Value in Health* 2018;**21 (Supple. 1)**:S180-S1

Devoe D, Farris M, Addington J. Symptoms of depression and anxiety in youth at risk for psychosis: A systematic review and metaanalysis. *Early Intervention in Psychiatry* 2018;**12 (Supple. 1)**:174

Devoe D, Farris MS, Townes P, Addington J. Interventions and transition in youth at risk of psychosis: A systematic review and meta-analysis. *Early Intervention in Psychiatry* 2018;**12 (Supple. 1)**:173

Devoe D, Farris MS, Townes P, Addington J. Interventions and social functioning in youth at risk of psychosis: A systematic review and meta-analysis. *Early Intervention in Psychiatry* 2018;**12 (Supple. 1)**:172

Dickens C, Cherrington A, Garrett C, Bower P, Bundy C, Gask L*, et al.* Characteristics of psychosocial interventions that improve depression in people with coronary heart disease: A systematic review with meta-regression. *Journal of Psychosomatic Research* 2010;**68 (6)**:619

Dickinson C, Whittingham K, Sheffield J, Wotherspoon J, Boyd R. Efficacy of interventions to improve psychological adjustment for parents who have an infant diagnosed with neurodevelopmental disability: A systematic review. *Developmental Medicine and Child Neurology* 2018;**60 (Supple. 1)**:20

Dieng M, Cust AE, Kasparian NA, Mann GJ, Morton RL. Economic evaluation of psychosocial interventions in cancer: A systematic review. *Asia-Pacific Journal of Clinical Oncology* 2015;**11 (Supple. 4)**:95

Doyle C, Foster N, Jordan J. Psychological interventions for long-term conditions: a review of approaches, content and outcomes. *Physiotherapy Research International* 2008;**13**:138

Dragioti E, Dimoliatis I, Evangelou V. An empirical assessment of psychotherapy allegiance in randomised controlled trials. *European Psychiatry* 2013;**28**

Ebrahim S, Montoya L, Truong W, Hsu S, El Din MK, Carrasco-Labra A*, et al.* Correction: Effectiveness of cognitive behavioral therapy for depression in patients receiving disability benefits: A systematic review and individual patient data meta-analysis (PLoS ONE). *Plos One* 2013;**8**

Elstner S. Psychotherapy for ADHD in people with IDD. *European Psychiatry* 2016;**33 (Supple.)**:S475-S6

Erdogan A, Coss-Adame E, Rao SSC. Systematic review of treatments for esophageal (noncardiac) chest pain. *Neurogastroenterology and Motility* 2013;**25 (Supple. 1)**:19

Evans J, Jack R, Harrison A. The scottish intercollegiate guideline network (SIGN) guideline on rehabilitation of cognitive and mood disorders after brain injury. *Brain Impairment* 2012;**13 (1)**:144

Eyrenci A, Ayalp GC. A meta-analysis of group psychotherapy studies for early stage breast cancer patients. *Psycho-Oncology* 2014;**23 (Supple. 3)**:195

Fagundes SBR, Fagundes DJL, Molen YF, Prado LBF, Carvalho JEC, Carvalho LBC*, et al.* What's new in primary Insomnia treatment? *Sleep Medicine* 2009;**10 (Supple. 2)**:S17

Falsaperla R, Saporito MAN, Di Stefano V, Pavone P. A31 Pandas: Tip of the iceberg. *Italian Journal of Pediatrics Conference: 73rd Congress of the Italian Society of Pediatrics Italy* 2017;**43 (Supple.2)**:15

Fangtham M, Nash JL, Hyon S, Bannuru RR, Wang C. Non-pharmacological treatment on fatigue, depression, disease activity, and quality of life of systemic lupus erythematosus: A systematic review. *Arthritis and Rheumatology* 2017;**69 (Supple. 10)**

Farris M, Devoe D, Addington J. Attrition rates in treatment trials: A systematic review and meta-analysis of clinical high-risk for psychosis interventions. *Early Intervention in Psychiatry* 2018;**12 (Supple. 1)**:182

Ferreira PH, Ho KK, Pinheiro MB, Aquino Silva D, Miller C, Grunstein R*, et al.* Sleep interventions for osteoarthritis and spinal pain: A systematic review of randomized control trials. *Osteoarthritis and Cartilage* 2018;**26 (Supple. 1)**:S243

Fishpool K, Jones B, Hewlett S, Ndosi M. 277 Online interventions for addressing psychological distress in people with rheumatoid arthritis and other long-term conditions: A systematic review. *Rheumatology* 2018;**57 (Supple. 3)**

Fishpool K, Jones B, Hewlett S, Ndosi M. THUR0730-HPR A systematic review of online interventions for addressing psychological distress in rheumatoid arthritis and other long-term conditions. *Annals of the Rheumatic Diseases* 2018;**77 (Supple. 2)**:1792

Forti Buratti M, Ramchandani P, Saikia R, Wilkinson E, Mehta N. Systematic review of psychological treatments for depression in children below 13 years old. *European Child and Adolescent Psychiatry* 2015;**24 (Supple.1)**:S167

Fredrikson M, Faria V, Agren T, Engman J, Furmark T. Meta-analytical evidence for segregating and integrating brain activation to symptom provocation in social anxiety disorder, specific phobia and post traumatic stress disorder. *Biological Psychiatry* 2011;**69 (9)**:S71-2

French LA, Nikolic-Novakovic L. P01-327 New dimensionsin assessing and treating traumatic stress: A meta-analysis of historical and recent contributions to the field. *European Psychiatry* 2010;**25 (Supple.1)**:540

Garbutt J. Predictors of response to naltrexone in alcohol dependence: A systematic review and integration of the world literature. *Journal of Addiction Medicine* 2013;**7 (4)**:E7-E8

Garg S, Garg D, Chowdhury F, Barron G, Turin TC. Web-based intervention for chronic back pain. *Canadian Family Physician* 2016;**62 (Supple. 1)**:S34

Garg SK, Wadhwa V, Anugwom CM, Gupta N, George J, Sanaka MR*, et al.* Mo1655 Effectiveness of pharmacological and non-pharmacological therapies for irritable bowel syndrome: A systematic review and Bayesian network meta-analysis. *Gastroenterology* 2016;**(Supple. 1)**:S-744

Gaudin D, Krafcik B, Mansour T, Alnemari AA. P160 - Considerations in spinal fusion surgery for chronic lumbar pain: Psychosocial factors, rating scales, and perioperative patient education: A review of the literature. *Global Spine Journal* 2017;**7 (2S)**:268S-9S

Gertler P, Tate RL, Cameron ID. A systematic review of non-pharmacological treatments for depression after TBI. *Brain Impairment* 2011;**(Supple. 1)**:70

Glozier N, Tofler G, Colquhoun D, Bunker S, Clarke D, D LH*, et al.* The national heart foundation of Australia consensus statement on depression, work stress and coronary heart disease (CHD). *Australian and New Zealand Journal of Psychiatry* 2013;**47 (S1)**:21

Goodman JH. Group treatment of postpartum depression: A systematic review. *Archives of Women's Mental Health* 2011;**14**:S68-S9

Goyal D, Parikh T, Fitzgerald J, Pruett J. 5.50 Assessment and Treatment of Irritability in Children and Adolescents: A Review of Literature for Evidence-Based Recommendations. *Journal of the American Academy of Child and Adolescent Psychiatry* 2018;**57 (10)**:S242-S3

Grigoriadis S, Kennedy S, Robinson G, VonderPorten E, Mamisashvili L, Peer M. A systematic review of treatments for depression in perimenopausal and postmenopausal women. *Journal of Women's Health* 2017;**26 (4)**:A22

Gritzner S, Antick J, Michael P, Cavanaugh R. Cognitive behavior therapy for fibromyalgia: A meta analysis. *Journal of Pain* 2012;**13(4)**:S97

Gudmundsdottir E. Support interventions for parents of children with cancer: A review of effectiveness reducing parental distress. *Pediatric Blood and Cancer* 2011;**57 (5)**:760

Hackett KL. Sleep disturbances in primary sjogren's syndrome: Evidence from the literature, patient sleep diaries and a qualitative focus group study. *Annals of the Rheumatic Diseases* 2018;**77 (Supple. 2)**:45

Hamrick N, Dickinson T. Spiritually-based interventions: Meta-analysis of impact on psychological well-being and comparison with cognitive interventions. *Psycho-Oncology* 2010;**19 (Supple. 1)**:S13

Han Shi Jocelyn Chew HSJ, Chair SY, Cheng HY. Comparing the effectiveness of cognitive behavioral interventions and motivational interviewing on improving self-care behaviors. *European Journal of Heart Failure* 2018;**20 (Supple. 1)**:72

Harsh R, Kundi PS, Ezeh D. A systematic review of the effectiveness of psychological interventions for adult sex-offenders in prisons, community clinics and forensic hospitals. *European Neuropsychopharmacology* 2015;**25(2)**:S653-S4

Hauser W. 141. Managing fibromyalgia: What works and what doesn't. *Rheumatology* 2014;**53 (Supple.1)**:i9

Hauser W, Kopp I. 0P0070 Update of the german evidence-based guideline on the management of fibromyalgia syndrome. *Annals of the Rheumatic Diseases* 2012;**71 (Supple.3)**:77

Hauser W, Nuesch E, Juni P. 0P0192 Comparative efficacy of pharmacological and non-pharmacological interventions in fibromyalgia. *Annals of the Rheumatic Diseases* 2012;**71 (Supple.3)**:119-20

Hauser W, Thieme K, Denis T. 150 A comparison of the US-American and German guideline with eular recommendations for the management of fibromyalgia. *European Journal of Pain* 2009;**13 (Supple. 1)**:S52

Hay P, Galletly C, Carter G, Andrews G, Chinn D, Forbes D*, et al.* RANZCP clinical practice guidelines for eating disorders. *Australian and New Zealand Journal of Psychiatry* 2015;**49 (Supple.1)**:30-1

Hazell P. Interventions for deliberate self-harm in adolescents. *Australian and New Zealand Journal of Psychiatry* 2017;**51 (Supple.1)**:82

Hegerl U. Self-management in affective disorders: How to use both patient generated data and Internet and CBT-based programmes in routine care. *European Psychiatry* 2018;**48 (Supple. 1)**:S42

Heidari E, Kamal KM, Giannetti V, Covvey JR. The impact of antidepressant medications on clinical outcomes of patients with type 2 diabetes: A systematic review. *Value in Health* 2018;**21 (Supple. 1)**:S70

Henwood KS, Chou S, Browne KD. "A systematic review and meta-analysis on the effectiveness of CBT informed anger management": Corrigendum. *Aggression and Violent Behavior* 2016;**27**:121

Heron H, Baken D, Harvey S. Meta-analysis of moderators of psycho-oncology therapy effectiveness: 'It's the sick who need a doctor'. *Psycho-Oncology* 2009;**18 (Supple. 2)**:S107

Hershberger A, Um M, Cyders M. The role of the UPPS-p impulsive personality traits in cognitive behavioral therapy based substance use treatment: A meta-analysis. *Alcoholism: Clinical and Experimental Research* 2017;**41 (Supple. 1)**:229A

Hewlett S. SP0147 Using cognitive-behavioural therapy to help patients self-manage their fatigue. *Annals of the Rheumatic Diseases* 2013;**72 (Supple.3)**:A34

Hochard KH, Burger K, Hulbert-Williams NJ. Comprehensive systematic review of insomnia treatments for people being treated for curable cancer. *Psycho-Oncology* 2016;**25 (Supple. 1)**:15

Hofmann SG, Asnaani A, Vonk IJJ, Sawyer AT, Fang A. "The efficacy of cognitive behavioral therapy: A review of meta-analyses": Erratum. *Cognitive Therapy and Research* 2014;**38**:368

Horton MS, Pugh M, Lawrence VA. Nonpharmacological therapy for insomnia in elders: A systematic review. *Journal of the American Geriatrics Society* 2010;**58(S1)**:S65

Howard LM. Partner violence in pregnancy and postpartum. *Archives of Women's Mental Health* 2013;**16**:S5

Hulbert-Williams N, Flynn S, Heaton-Brown L, Scanlon K. Interventions to improve the well-being of breast cancer survivors at the end of active treatment: A systematic review of the literature. *Psycho-Oncology* 2013;**22 (Supple.3)**:329-30

Hunt JIE. Treatments for OCD in pediatric patients...obsessive-compulsive disorder. *Brown University Child & Adolescent Psychopharmacology Update* 2008;**10(8)**:4-5

Huntley A, Moore T, King A, Evans M, Persad R, Sharp D. Supportive care interventions for men with prostate cancer: A systematic review. *Supportive Care in Cancer* 2015;**23**:S369

Indrielle T, Keay S. P0196 Does psychotherapy improve success rates of in vitro fertilisation? systematic review. *International Journal of Gynecology and Obstetrics* 2015;**(5)**:E371

Isaac D. Clinical benefits of exercise and psychological interventions in patients with cancer-related fatigue. *Journal of Clinical Outcomes Management* 2017;**24**:200-2

Jayasekara R. Cognitive behavioral therapy for men who physically abuse their female partner. *Journal of Advanced Nursing* 2008;**64**:129-30

Jomaa I, Saini B, Miller C. Is cognitive behavioural therapy for the treatment of insomnia harmful? A systematic review of the literature. *Journal of Sleep Research* 2017;**26 (Supple. 1)**:37-8

Junghan UM, Pfammatter M. What are the therapeutic ingredients of cognitive behavior therapy for psychosis? A systematic review. *European Archives of Psychiatry and Clinical Neuroscience* 2011;**261**:S36

Kangas M, Bovbjerg DH, Montgomery GH. "Cancer-related fatigue: A systematic and meta-analytic review of non-pharmacological therapies for cancer patients": Correction to Kangas, Bovbjerg, and Montgomery (2008). *Psychological Bulletin* 2009;**135**:172

Kaplan SG. Motivational interviewing in the treatment of pediatric obesity: Research and promise. *Psychosomatic Medicine* 2011;**73 (3)**:A7

Kazantzis N. Translating science into practice, collaborative empiricism and engagement in homework assignments in cognitive behavior therapy. *Bulletin of Clinical Psychopharmacology* 2011;**21 (Supple.2)**:S41

Khan A, Tansel A, White D, Blais P, Lindsay J, El-Serag HB*, et al.* Mo1248 Psychosocial interventions to achieve abstinence in patients with chronic liver disease and alcohol use disorders: A systematic review. *Gastroenterology* 2015;**148(4)**:S-649

Kingdon D. Advances in cognitive behaviour therapy for psychosis. *European Psychiatry Conference: 19th European Congress of Psychiatry, EPA* 2011;**26**

Kitsumban V, Thapinta D, Picheansathian W. The Effectiveness of Cognitive-Behavioural Therapy on Depression in the Elderly. *JBI Library of Systematic Reviews* 2012;**10**:1-9

Koehler M, Hoppe S, Peplinski D, Richter D, Frommer J, Flechtner HH*, et al.* Psycho-oncologic interventions for parents of cancer patients: Systematic review. *Oncology Research and Treatment* 2015;**38 (Supple.5)**:172

Kohli IS, Kataria A, Singla S, Kaushik P, Jindal R, Aggarwal A. Effect of cognitive behavioural therapy in multiple sclerosis fatigue: A systematic review of randomised controlled trials. *Value in Health* 2012;**15 (7)**:A545-A6

Konnopka A, Schaefert R, Heinrich S, Leicht H, Kaufmann C, Luppa M*, et al.* The economic burden of somatization syndromes: A systematic review of cost-of-illness studies and cost-effectiveness analyses. *Journal of Mental Health Policy and Economics* 2011;**(1)**:S16-S7

Kuehner C. S11-04 Psychological treatments and prevention in bipolar disorder - Recent developments. *European Psychiatry* 2009;**24 (Supple.1)**:S61

Kuehner C. S11-03 - Evidence-based psychotherapy for chronic depression. *European Psychiatry* 2010;**25 (Supple.1)**:41

Kuo MH, Brown CA, Phillips L, Berry R, Tan M. Non-pharmacological sleep interventions for youth with chronic health conditions: A systematic review. *Sleep* 2012;**35 (Supple.1)**:A307-A8

Laird K, Tanner-Smith E, Russell A, Hollon S, Walker L. Comparative efficacy of psychological therapies for improving mental health and functioning in irritable bowel syndrome: A systematic review and meta-analysis. *Journal of Alternative and Complementary Medicine* 2016;**22 (6)**:A93

Landa-Ramirez E, Rivero-Rosas A, Cardenas-Lopez G, Greer JA, Sanchez-Roman S, Field A. Cognitive-behavioral therapy for depression and anxiety in advanced cancer: A literature review. *Psycho-Oncology* 2013;**22 (Supple.3)**:226

Landmark B, Reinar LM, Brurberg KG, Hammerstrom KA, Almas E, Aars H*, et al.* Effects of sexological therapy for sex offenders. A systematic review. *Journal of Sexual Medicine* 2013;**10 (Supple.5)**:315

Larkin D, Lopez V, Aromataris E. Interventions for managing cancer-related fatigue in men treated for prostate cancer: A systematic review. *Supportive Care in Cancer* 2013;**21**:S101-S2

Lars Jerden L, Kiessling A, Wetterqvist A, Hambreus K, Perk J. Establishment of national guidelines in Sweden for lifestyle modification in healthcare. *European Journal of Preventive Cardiology* 2013;**20 (Supple.1)**:S65

Laska I, Swieca J, Meaklim H, Kelly D, Cunnington D. Psychologically-based treatment strategies for restless legs syndrome: A review. *Journal of Sleep Research* 2017;**26 (Supple. 1)**:38

Lee M, Patel T, Lee L. Nonpharmacologic outpatient interventions for benzodiazepine discontinuation in elderly persons. *Canadian Family Physician* 2015;**61 (Supple. 1)**:S55

Lee V, Balucani C, DeLuca J, Lederman YS, Arnedo V, Lushbough CA*, et al.* Abstract 3684: Post-stroke fatigue: A systematic evidence-based critique. *Stroke* 2012;**43 (Supple.1)**:A3684

Lehman A, Yohannes S, MacDonald C. Moving from patient-centred to family-centred care? A systematic review of psycho-educational programs for people and partners affected by arthritis. *Reumatologia Clinica Suplementos* 2011;**7** 167

Leverich AE, Acke S, Verbrugghe M, Schmickler MN, De Brouwer C. Efficiency of vocational rehabilitation programs for workers with schizophrenia: A systematic literature review according to the prisma guidelines. *Occupational and Environmental Medicine* 2018;**75 (Supple. 2)**:A612-A3

Li L, Xiong L. Meta-analysis: Cognitive-behavioral therapy for irritable bowel syndrome. *Gastroenterology* 2014;**146 (5)**:S-223

Lim C, Sam HF, Renjan V, Quah SL. A systematic review of adapted cognitive-behavioural therapy for religious individuals with severe mental illness. *Annals of the Academy of Medicine Singapore* 2011;**40 (Supple.)**:S62

Lipsey MW. Variability across treatments and outcomes: Meta-analysis of the effects of adolescent substance abuse treatment. *Alcoholism: Clinical and Experimental Research* 2012;**36 (S1)**:330A

Liu P, Parker A, Hetrick S, Purcell R. Evidence mapping for early psychotic disorders in young people. *Schizophrenia Research* 2010;**117 (2-3)**:438-9

Lock J. 3.3 Evidence-based psychosocial treatments for eating disorders in children and adolescents. *Journal of the American Academy of Child and Adolescent Psychiatry* 2016;**55 (10)**:S88-S9

Loucas C, Pennant M, Whittington C, Naqvi S, Sealey C, Stockton S, *et al.* G130 E-therapies for mental health problems in children and young people: A systematic review and focus group investigation. *Archives of Disease in Childhood* 2014;**99**:A58

Lynch MJ, George TP. Therapeutic mechanisms underlying the effects of alcoholics anonymous: Results of a systematic literature review. *American Journal on Addictions* 2013;**22 (3)**:306

MacMillan H, Jack SM, MacGregor J, Wathen N. 22.1 Identifying and responding to intimate partner violence exposure among children and adolescents in pediatric settings. *Journal of the American Academy of Child and Adolescent Psychiatry* 2017;**56 (10)**:S32

Marian A, Dumitrascu DL. Predictors of response to cognitive-behavioral therapy in patients with irritable bowel syndrome. *Journal of Gastrointestinal and Liver Diseases* 2017;**26 (Supple. 1)**:59

Matthews EE, McCarthy MS. Adherence to cognitive behavioral therapy for insomnia. Paper presented at: Western Institute of Nursing Annual Communicating Nursing Research Conference 2014.

Matthews H, Grunfeld B, Turner A. The efficacy of psychosocial interventions among women following surgical treatment for breast cancer: A systematic review and meta-analysis. *European Journal of Cancer* 2016;**57 (Supple. 2)**:S151

Mayo-Wilson E. Internet-based cognitive behaviour therapy for symptoms of depression and anxiety: a meta-analysis. *Psychological Medicine* 2007;**37**:1211

McDaid D, Park A, Parsonage M. Making the economic case for tackling somatoform disorders. *Psychiatrische Praxis* 2011;**38 (Supple. 1)**

McKay GD, Berkowitz Sturgis EK, Grandner MA, Gehrman P, Perlis ML. 0646 Response and remission definitions for CBT-I: A quantitative review. *Sleep* 2012;**35 (Supple. 1)**:A219

British Journal of Hospital Medicine. Early cognitive behavioural therapy reduces risk of psychosis, finds meta-analysis. *British Journal of Hospital Medicine* 2013: 250.

Mehta S, Orenczuk S, Teasell R. Poster 47. Evidence based management of depression following spinal cord injury: A meta-analysis. *Archives of Physical Medicine and Rehabilitation* 2011;**92**:1706-7

Mei Ling L, Creedy DK, Moon Fai C. Effectiveness of psychological-based interventions to enhance coping of nursing students: A systematic review. *JBI Database of Systematic Reviews and Implementation Reports* 2010;**8(34)**:S1-24

Menezes A. 50 -Psychosocial interventions for common mental disorders in Primary Health Care. *Journal of Psychosomatic Research* 2013;**74**:554

Messer SC. 1148. Differential effectiveness of psychological and pharmacotherapy interventions for ptsd: A meta-analytic review. *American Journal of Epidemiology* 2011;**173 (Supple.)**:S287

Michura D, Bonn J. O012. The effectiveness of group cognitive behavioural therapy in the management of chronic low back pain. *Physiotherapy* 2017;**103 (Supple. 1)**:e8-e9

Mikocka-Walus A. 51 - Psychosomatic medicine and inflammatory bowel disease: A new integrated model of care. *Journal of Psychosomatic Research* 2013;**74 (6)**:554

Mikocka-Walus A. 245. Cognitive-behavioural therapy in inflammatory bowel disease: What's the evidence? *Psychotherapy and Psychosomatics* 2013;**82 (Supple. 1)**:67

Mikocka-Walus AA, Turnbull DA, Holtmann G, Andrews JM. Sa1031. Coping with the unmet needs of gastroenterology and hepatology outpatients: A systematic approach towards an integrated model of care in South Australia. *Gastroenterology* 2011;**58(1)**:S-208

Minen M, Jinich S, Vallespir EG. Behavioral therapies and mind body interventions for post traumatic headache and post-concussive symptoms: A systematic review. *Headache* 2018;**58 (Supple. 2)**:201

Morell GFC. Insufficient evidence to support or reject effect of conservative TMD therapies on otologic signs and symptoms. *Evidence-Based Dentistry* 2018;**19**:26-7

Moritz S. A reply to "Effectiveness of an individually-tailored computerised CBT programme (Deprexis) for depression: A meta-analysis" by Twomey and colleagues. *Psychiatry Research* 2018;**263**:282

Moss-Morris R, Castell BD, Kazantzis N. Cognitive behavioral therapy and graded exercise for chronic fatigue syndrome: A comparative meta-analysis including moderators of effects. *Psychosomatic Medicine* 2012;**74 (3)**:A89

Munn Z, Yifan X, Moola S, McArthur A. Children's views about obesity, body size, shape and weight; Inequalities and the mental health of young people: a systematic review of secondary school-based cognitive behavioural interventions; Intervention strategies that support self-care activities: an integrative study across disease/impairment groupings; Advocacy interventions to reduce or eliminate violence and promote the physical and psychosocial well-being of women who experience intimate partner abuse. *Journal of Advanced Nursing* 2011;**67**:954-60

Natale P, Palmer S, Ruospo M, Saglimbene V, Hegbrant J, Strippoli G. FO025. Interventions to improve sleep quality in people with chronic kidney disease: a cochrane systematic review. *Nephrology Dialysis Transplantation* 2018;**33 (Supple. 1)**:i28

Nickel F, Kolominsky-Rabas PL. Health economic evidence on non-pharmacological interventions for persons with dementia: A systematic review. *Value in Health* 2017;**20 (9)**:A713-A4

Nikandrou D. What psychological interventions have been found to reduce overall distress in cancer patients? A systematic review. *Psycho-Oncology* 2016;**25 (Supple. 1)**:20

Niknejad B, Bolier R, Delgado D, Henderson C, Reid M. The efficacy of cognitive-behavioral interventions for chronic pain in older adults: A Meta-analysis. *Journal of the American Geriatrics Society* 2017;**65 (Supple 1)**:S144-S5

No authorship indicated. "Effect of cognitive-behavioral therapy for anxiety disorders on quality of life: A meta-analysis": Correction to Hofmann, Wu, and Boettcher (2014). *Journal of Consulting and Clinical Psychology* 2014;**82**:1228

No authorship indicated. "The Effects of Cognitive Behavioral Therapy as an Anti-Depressive Treatment is Falling: A Meta-Analysis": Correction to Johnsen and Friborg (2015). *Psychological Bulletin* 2016;**142**:290

Noble A, Reilly J, Temple J, Fisher P, Snape D. Cognitive-behavioural therapy does not meaningfully reduce depression in most people with epilepsy: A systematic review with a reliable change analysis. *Epilepsia* 2018;**59 (Supple. 3)**:S242-S3

Noble F, Marshman Z. The effectiveness of Cognitive Behavioural Therapy in the reduction of dental anxiety in children. *Evidence-based dentistry* 2018;**19**:104

Nordentoft M, Austin S, Jeppesen P, Melau M, Thorup A. Poster #244. Crucial elements in treatment of first-episode psychosis patient, psychosocial aspects. *Schizophrenia Research* 2012;**136 (Supple. 1)**:S369

Nordentoft M, Melau M. A90. Crucial elements in treatment of first-episode psychosis patient, psychosocial aspects. *Early Intervention in Psychiatry* 2014;**8 (Supple. 1)**:86

Norman L, Taylor S, Liu Y, Radua J, Abelson J, Angstadt M*, et al.* S20. Error-processing in OCD: A meta-analysis of fMRI studies and investigation of changes following CBT. *Biological Psychiatry* 2018;**83 (9)**:S354

Norton C, Czuber-Dochan W, Artom M, Sweeney L, Hart A. P555 Systematic review of interventions for chronic abdominal pain management in inflammatory bowel disease. *Journal of Crohn's and Colitis* 2017;**11 (Supple. 1)**:S363

O'Toole MS, Zachariae RS, Renna ME, Mennin D, Applebaum A. Cognitive behavioral therapies for informal caregivers of patients with cancer and cancer survivors: A systematic review and meta-analysis. *Psychosomatic Medicine* 2016;**78 (3)**:A99-A100

Olthuis JV, Watt MC, Bailey K, Hayden JA, Stewart SH. Therapist-supported internet cognitive-behavioural therapy for anxiety disorders in adults. *BJ Psych Advances* 2015;**21**:290

Omlin X, Ballesio A, Aquino MRJ, Espie CA, Kyle SD. The effect of cognitive behavioural therapy for insomnia on quality of life outcomes: A meta-analysis of randomised controlled trials. *Journal of Sleep Research* 2018;**27 (Supple. 1)**:174-5

Panciroli G, Marchi M, Mattei G, Galeazzi GM, Ferrari S. Efficacy of music therapy interventions in the treatment of mood disorders: A systematic review. *European Psychiatry* 2018;**48 (Supple. 1)**:S357

Parslow R, Purcell R, Garner B, Hetrick SE. Combined pharmacotherapy and psychological therapies for post traumatic stress disorder (PTSD). *Cochrane Database of Systematic Reviews* 2008;**(3)**:CD007316

Penades R. Integrating cognitive remediation and other interventions to improve social functioning. *European Archives of Psychiatry and Clinical Neuroscience* 2009;**259 (Supple. 1)**:S41-S2

Perez PV, De Azua SR, Martinez M, Ron S, Oliveros RG, Asua J*, et al.* P.3.a.006. Psychological treatment in the first psychotic episode. *European Neuropsychopharmacology* 2009;**19 (Supple. 3)**:S483-S4

Pfammatter M. The empirical status of CBT for psychosis: Controlled efficacy, indication and therapeutic factors. A systematic review of metaanalytic findings. *European Psychiatry* 2011;**26 (Supple. 1)**:1475

Phianmongkhol Y, Thongubon K, Woottiluk P. Cognitive Behavioral Therapy Techniques for Pain in Lung Cancer Patients: A Systematic Review. *JBI Library of Systematic Reviews* 2011;**9**:1-11

Pijnenborg M, Van Donkersgoed R, David A, Aleman A. Treatment of insight in psychosis: A meta-analysis. *Schizophrenia Research* 2012;**136 (Supple. 1)**:S76-S7

Poiraudeau S, Palazzo C. Therapeutic advances in chronic low back pain management: A systematic review of the litterature over the 5 past years. *Annals of Physical and Rehabilitation Medicine* 2012;**55 (Supple.1)**:e284-e5

Police R, Zhao Y, Russell M, Foster T. A systematic review of the burden, epidemiology, costs and treatment of chronic low back pain. *Journal of Pain* 2009;**10(4)**:S5

Pompoli A, Furukawa TA, Imai H, Tajika A, Efthimiou O, Salanti G. Psychological therapies for panic disorder with or without agoraphobia in adults: A network meta-analysis. *BJPsych Advances* 2018;**24**:2

Ponnuthurai SA, Brown J. Meta-Analysis of the Outcome of RCTs of Preventative or Early Intervention Universal Group Psychological Therapies in Under 18s. *Journal of the American Academy of Child and Adolescent Psychiatry* 2018;**57 (10)**:S189

Pozza A, Andersson G, Dettore D. Therapist-guided internet-based cognitive-behavioural therapy for adult obsessive-compulsive disorder: A meta-analysis. *European Psychiatry* 2016;**33 (Supple.)**:S276-S7

Puyraimond-Zemmour D, Romand X, Lavielle M, Molto A, Gaudin P, Soubrier M*, et al.* Adherence to disease-modifying drugs in chronic inflammatory rheumatic diseases: Several questionnaires, diverse patient characteristics and some efficacious interventions-a systematic literature review. *Annals of the Rheumatic Diseases* 2018;**77 (Supple. 2)**:1194

Raciti AE. Effectiveness of interventions for parasomnias among soldiers who have Post Traumatic stress disorder. *Sleep* 2016;**39 (Supple.)**:A282-A3

Ramsenthaler C, Siegert RJ, Weatherall M, Bausewein C, Koffman J, Higginson IJ*, et al.* Cognitive behavioural therapy (CBT) for panic and anxiety in chronic obstructive pulmonary disease-a systematic review. *Palliative Medicine* 2012;**26 (4)**:405-6

Rebbeck T, Stewart M, Cameron I, Stewart J. Treatment of chronic whiplash: A systematic review and clinical guidelines. *Physiotherapy* 2011;**97 (Supple. 1)**:eS1038-eS9

Rezaie L, Rassafiani. Is cognitive behavior therapy (CBT) an alternative treatment for pharmacotherapy for children with obsessive compulsive disorders? (A critically appraised topic). *Iranian Journal of Psychiatry* 2012;**(1)**:133

Rice S, Goodall J, Hetrick S, Parker A, Gilbertson T, Amminger P*, et al.* Online and social networking interventions for the treatment of depression in young people: Literature review and new horizons. *Early Intervention in Psychiatry* 2014;**8 (Supple. 1)**:74

Riehle M, Pillny M, Buggisch S, Lincoln T. The efficacy of psychosocial treatments for patients with present negative symptoms: A meta-analysis. *European Archives of Psychiatry and Clinical Neuroscience* 2017;**267 (Supple. 1)**:S52

Robbins R, Underwood PE, Jackson C, Chen M, Kuriakose S, Jean-Louis G*, et al.* A systematic review of worksite interventions and their impact on employee sleep. *Sleep* 2018;**41 (Supple. 1)**:A219

Rogers VE, Diaz-Abad M, Liu W, Geiger-Brown J. Meta-analysis of CBT-I for the treatment of comorbid insomnia. *Sleep* 2015;**38 (Supple. 1)**:A228

Rolls C, Prior Y. 285 Non-pharmacological interventions for people with fibromyalgia: A systematic review. *Rheumatology* 2018;**57 (Supple. 3)**

Rueda S, Rzeznikiewiz D, Park-Wyllie L, Bayoumi A, Tynan AM, Antoniou T*, et al.* A systematic review of the effectiveness of sociobehavioral interventions to improve adherence to combination antiretroviral therapy in HIV. *Journal of the International Association of Physicians in AIDS Care* 2010;**9 (1)**:58

Sahota P, Wordley J, Woodward J. Effective behavioural components in child and adolescent weight management programmes. *Obesity Reviews* 2011;**12 (Supple. 1)**:57-8

Sajid SS, Kotwal A, Dale W. Interventions to reduce health disparities in prostate cancer: A systematic literature review. *Journal of the American Geriatrics Society* 2011;**59**:S94

Sanchez Ortuno M, Seda G, Welsh C, Halbower A, Edinger J. Comparative meta-analysis of prazosin and imagery rehearsal therapy for nightmares, sleep disturbance and post-traumatic stress. *Sleep Medicine* 2013;**14 (Supple. 1)**:e48

Sanchez-Meca J, Rosa-Alcazar AI, Lopez-Soler C. "The psychological treatment of sexual abuse in children and adolescents: A meta-analysis": Erratum. *International Journal of Clinical and Health Psychology* 2011;**11(2)**

Sanjuan J, Liano V, Balaguer E, Aguilar EJ. Neuroimaging techniques and response to psychological intervention in psychotic patients: A systematic review. *Schizophrenia Bulletin* 2011;**37 (Supple. 1)**:151-2

Seda G, Sanchez-Ortuno MM, Welsh CH, Ann HC, Edinger JD. Comparative meta-analysis of prazosin and imagery rehearsal therapy for nightmares, sleep disturbance and post-traumatic stress. *Sleep* 2014;**37 (Supple. 1)**:A276

Sedlar N, Lainscak M, Farkas J. Psychological interventions to improve psychological outcomes in patients with heart failure: A systematic literature review. *European Journal of Heart Failure* 2018;**20 (Supple. 1)**:586

Seppala A, Miettunen J, Hirvonen N, Isohanni M, Moilanen J, Koponen H*, et al.* What do we know about treatment-resistant schizophrenia?-A systematic review. *European Psychiatry* 2016;**33 (Supple.)**:S586

Seppala J, Seppala A, Isohanni M, Miettunen J, Jaaskelainen E. Treatment-resistant and difficult-to treat schizophrenia as a challenge for clinical practices. Data from Finnish samples: Northern Finland birth cohort 1966 and Perfect-project. *European Archives of Psychiatry and Clinical Neuroscience* 2017;**267 (Supple. 1)**:S16-S7

Sesel AL, Sharpe L, Naismith S. The efficacy of psychosocial interventions for people with multiple sclerosis: A meta-analysis of specific treatment effects. *Multiple Sclerosis Journal* 2017;**23(13)**:NP1

Shi Q, Xiang T, Liangren L, Zhenhua L, Lu Y, Qiang W. 946. Psychosocial interventions to improve the quality of life for men with prostate cancer: A Bayesian network meta-analysis of 31 randomised controlled trails. *European Urology Supplements,* 2017;**16(3)**:e1648

Silva M, Figueiredo AR, Fornelos A, Macedo P, Nunes S. Psychotherapeutic interventions in Tinnitus. *European Psychiatry* 2016;**33 (Supple.)**:S561

Singh N, Dornadula I, Ramezani A, Sheth S, De Mesa C. Psychological preparation for neuromodulation: A systematic review. *Neuromodulation* 2018;**21(3)**:e72

Sjogren T, Avikainen L, Calleeuw N, Forsblom K, Keranen K, Lautamaki L*, et al.* Motor control exercise for non-specific low back pain: A systematic review and meta-analysis of randomized controlled trials. *Physiotherapy* 2015;**101 (Supple. 1)**:eS1405

Sleight A. Rehabilitation interventions and coping techniques for chemotherapy-related cognitive dysfunction. *Archives of Physical Medicine and Rehabilitation* 2015;**96 (10)**:e115-e6

Sleight A, Clark F. Chemotherapy-related cognitive dysfunction and the role of rehabilitation: A scoping review of the literature. *Supportive Care in Cancer* 2015;**23**:S310-S1

Smeets KC, Rommelse NNJ, Scheepers FE, Buitelaar JK. Responder and non-responder profiles of a cognitive behavior therapy to reduce aggression in adolescents. *European Child and Adolescent Psychiatry* 2015;**24**:S50-S1

Smith K. Network meta-analysis: A useful tool for comparing different psychological treatments for panic disorder?: Commentary on... Cochrane Corner. *BJPsych Advances* 2018;**24**:3-8

Spiegel D, Krizanec S, Kraemer H, Jo B, Ershadi M, Neri E*, et al.* Meta-analysis of psychosocial treatment effects on cancer survival and sources of heterogeneity. *Neuropsychopharmacology* 2017;**43 (Supple. 1)**:S331

Ssegonja R, Nystrand C, Feldman I, Sarkadi A, Langenskiold S, Jonsson U. Indicated preventive interventions for depression in children and adolescents: A meta-analysis and meta-regression. *Value in Health* 2018;**21 (Supple. 1)**:S181

Staugaard CF, Uhre V, Lonfeldt N, Pretzmann L, Vangkilde S, Plessen K*, et al.* Cognitive behavioral therapy for obsessivecompulsive disorder in children and adolescents-preliminary results from a systematic review. *Early Intervention in Psychiatry* 2018;**12 (Supple. 1)**:166

Strauss N, Rabin J, Temel B, Philpotts L, Ostroff J, Park ER*, et al.* Smoking cessation interventions for Black and Hispanic cancer patients: A systematic review of tobacco trials. *Psycho-Oncology* 2017;**26 (Supple. 1)**:87

Stuhldreher N, Konnopka A, Wild B, Herzog W, Zipfel S, Lowe B*, et al.* Cost-of-illness studies and cost-effectiveness analyses in eating disorders: A systematic review. *Journal of Mental Health Policy and Economics* 2013;**(1)**:S33

Sutherland R, O'Callaghan V, Henderson S, Nelson A, Jefford M, Zorbas H. Recommendations for the identification and management of fear of cancer recurrence in adult cancer survivors. *Psycho-Oncology* 2014;**23 (Supple. 3)**:109-10

Swanson S, Dolce A, Marsh K, Summers J, Sheldon LK. Interventions to prevent and treat anxiety: a review of the evidence and implications for practice. *Oncology Nursing Forum* 2008;**35**:525

Szeverenyi C, Kekecs Z, Elkins G, Csernatony Z, Varga K, Johnson A. Psychosocial interventions as adjuncts to orthopedic surgery: A systematic review and meta-analysis. *Global Advances in Health and Medicine* 2018;**7**:258-9

Teo SY, Loy FL. A physiotherapy clinical pathway for the management of chronic neck pain. *Annals of the Academy of Medicine Singapore* 2015;**44 (Supple.)**:S304

Thombs BD. Screening for depression in cardiac patients. *Journal of Psychosomatic Research* 2010;**68 (6)**:670

Tirado-Munoz J, Gilchrist G, Torrens M. Intimate partner violence among drug dependent women: State of the art. *Heroin Addiction and Related Clinical Problems* 2016;**18 (3 Supple. 1)**:25-6

Tolin DF. "Beating a dead dodo bird: Looking at signal vs. noise in cognitive-behavioral therapy for anxiety disorders": Corrigendum. *Clinical Psychology: Science and Practice* 2015;**22**:315-6

Towbin K, DeLonga K. 21.1 Integrated Treatment of Refractory Depression in Youth: A Review of Medication, Psychological Treatments, and Barriers to Optimal Care. *Journal of the American Academy of Child and Adolescent Psychiatry* 2018;**57 (10)**:S31

Trauer J, Qian M, Doyle J, Rajaratnum S, Cunnington D. 180 Efficacy of cognitive behavioural therapy for insomnia: A systematic review and meta-analysis. *Sleep and Biological Rhythms* 2014;**12 (Supple.)**:58

Troy D, Arora J, Siefferman J. (370) Addressing attention deficits in the treatment of chronic pain: A systematic review. *Journal of Pain* 2014;**15 (4)**:S68

Van Ameringen M, Patterson B. T92. The use of augmentation strategies in treatment resistant anxiety disorders: A systematic review and meta-analysis. *Neuropsychopharmacology* 2014;**39**:S350-S1

Van Berkel DM, Van Den Heuvel S, Van Ravensberg D. Effects of specialized psychosomatic physical therapy in patients with stress related health problems: A systematic literature review. *Physiotherapy* 2011;**97 (Supple.)**:eS1277-eS8

Van Der Gaag M, Ising H. Latest developments of psychological psychosis prevention strategies in the Netherlands. *Early Intervention in Psychiatry* 2018;**12 (Supple. 1)**:76

Van Der Gaag M, Smit F, Bechdolf A, French P, Linszen DH, Yung A*, et al.* Preventing a first episode of psychosis: A meta-analysis. *Schizophrenia Research* 2014;**153 (Supple. 1)**:S42

Van Der Gaag M, Smit F, Bechdolf A, French P, Linszen DH, Yung AR*, et al.* Preventing a first episode of psychosis: Meta-analysis of randomized controlled prevention trials. *Schizophrenia Bulletin* 2013;**39 (S1)**:S356

Van Der Gaag M, Velthorst E, Smit F, Meyer C, Koeter M, Fett AK*, et al.* The battle for ameliorating negative symptoms has a restart. *Early Intervention in Psychiatry* 2014;**8(S1)**:23

Van Der Gaag M, Velthorst E, Smit F, Meyer C, Koeter M, Fett AK*, et al.* Psychotherapy in people with negative symptoms. *European Archives of Psychiatry and Clinical Neuroscience* 2015;**265 (Supple.)**:S40

Van Straten A, Van Der Zweerde T, Morin C, Lancee J. Cognitive and behavioural therapies in the treatment of insomnia: A systematic metaanalysis of all the literature. *Journal of Sleep Research* 2018;**27 (Supple. 1)**:167

Vandepitte S, Van Den Noortgate N, Putman K, Verhaeghe S, Faes K, Annemans L. Effectiveness of supporting informal caregivers of people with dementia: A systematic review. *Value in Health* 2015;**18 (7)**:A407-A8

Vasa RA. 8.5 Assessment and Management of Anxiety in Youth With ASD. *Journal of the American Academy of Child and Adolescent Psychiatry* 2018;**57(10)**:S134-S5

Verberne D, Spauwen P, Van Heugten C. Neuropsychological interventions for treating neuropsychiatric consequences of acquired brain injury: A systematic review. *Brain Injury* 2017;**31 (6-7)**:797

Vitinius F, Bassou S, Albus C. Psychotherapeutic and psychiatric interventions after bonemarrow/stemcell transplantation –A systematic review. *Journal of Psychosomatic Research* 2011;**70 (6)**:621-2

Vitinius F, Imlau C, Albus C. Psychosocial strain and therapeutic approaches of hereditary neurological diseases: A systematic review. *Journal of Psychosomatic Research* 2013;**74 (6)**:560

Vitinius F, Piontek K, Albus C. Psychosocial interventions to improve quality of life, depression and anxiety in lung transplant patients -A systematic review of randomized controlled trials. *Journal of Psychosomatic Research* 2011;**70 (6)**:620

Vollm B, Gibbon S, Khalifa N, Duggan C, Stoffers J, Huband N, *et al.* S08-01-Cochrane reviews of pharmacological and psychological interventions for antisocial personality disorder (ASPD). *European Psychiatry Conference: 18th European Congress of Psychiatry Munich Germany Conference Publication:* 2010;**25 (Supple. 1)**

Walling AD. Which treatments are effective for reducing adolescent alcohol abuse? *American Family Physician* 2010;**82**:532-4

Wang C, Bayes S. Effectiveness of acupuncture as an add-on treatment for women with postnatal depression: a systematic review. *Women and Birth* 2018;**31 (Supple. 1)**:S27

Wang Z. Errors in Meta-analysis of Trial Comparing Effectiveness and Safety of Cognitive Behavioral Therapy With Pharmacotherapy for Childhood Anxiety Disorders. *JAMA Pediatrics* 2018;**172**:983-4

Wearden AJ, Russell C, Emsley R, Fairclough G, Kyle SD. Sleep and fatigue in chronic fatigue syndrome. *Psychosomatic Medicine* 2016;**78 (3)**:A24

Webb M. A review of web-based applications used to support self-management of non-specific chronic low back pain. *British Journal of Pain* 2017;**11 (Supple. 2)**:51-2

Wendebourg MJ, Heesen C, Finlayson M, Meyer B, Pottgen J, Kopke S. Patient education for people with multiple sclerosisassociated fatigue: A systematic review. *Multiple Sclerosis* 2016;**22 (Supple. 3)**:700

Whale R, Bucur M. Systematic review of randomised interventions for preonset phase psychosis. *Early Intervention in Psychiatry* 2010;**4 (S1)**:116

White D, Luther L. Efficacy of cognitive behavior therapy in early psychosis. *Schizophrenia Bulletin* 2017;**43 (Supple. 1)**:S209

Wiener J, Mehta S, Iruthayarajah J, Janssen S, Teasell R. The effectiveness of cognitive behavioural therapy for the management of post-stroke depressive symptoms. *Archives of Physical Medicine and Rehabilitation* 2017;**98 (10)**:e137

Williams A, Eccleston C, Morley S. 1012 Systematic review and meta-analysis of psychological treatments for persistent pain in adults, excluding headache. *European Journal of Pain* 2009;**13 (Supple.1)**:S284

Williams A, Morley S. Systematic review and meta-analysis of psychological treatments for persistent pain in adults, excluding headache. *Journal of Pain* 2009;**10 (4)**:S62

Yoshinaga N, Nosaki A, Unozawa K, Hayashi Y, Shimizu E. A systematic review of cognitive behavioral therapy in nursing field in Japan. *Asia-Pacific Psychiatry* 2015;**7 (Supple. 1)**:26

Zafar Usmani A, Ni Cheng J, Smith BJ, Carson KV. A meta-analysis (cochrane review) of pharmacological and psychological interventions for anxiety and depression in COPD. *Respirology* 2010;**15 (Supple. 1)**:A19

Zangi HA. The evidence for patient education in inflammatory arthritis. *Annals of the Rheumatic Diseases* 2014;**73 (Supple. 2)**:55-6

h. References of studies excluded as duplicates/superceded/withdrawn (170)

Abbott RA, Martin AE, Newlove-Delgado TV, Bethel A, Whear RS, Coon JT*, et al.* Recurrent Abdominal Pain in Children: Summary Evidence From 3 Systematic Reviews of Treatment Effectiveness. *Journal of Pediatric Gastroenterology & Nutrition* 2018;**21**:21

Aggarwal VR, Lovell K, Peters S, Javidi H, Joughin A, Goldthorpe J. Psychosocial interventions for the management of chronic orofacial pain. *Cochrane Database Syst Rev* 2011;**9(11)**:CD008456

Aggarwal VR, Lovell K, Peters S, Javidi H, Joughin A, Goldthorpe J. Psychosocial interventions for the management of chronic orofacial pain. *Cochrane Database Syst Rev* 2015;**17(12)**:CD008456

Akechi T, Okuyama T, Onishi J, Morita T, Furukawa TA. WITHDRAWN: Psychotherapy for depression among incurable cancer patients. *Cochrane Database Syst Rev* 2018;**11**:CD005537

Alessi C, Vitiello MV. Insomnia (primary) in older people. *Clinical Evidence* 2011;**11**:11

Andrews G, Cuijpers P, Craske MG, McEvoy P, Titov N. Computer therapy for the anxiety and depressive disorders is effective, acceptable and practical health care: a meta-analysis. *Plos One* 2010;**5**:e13196

Anie KA, Green J. Psychological therapies for sickle cell disease and pain. *Cochrane Database Syst Rev* 2002;**(2)**:CD001916

Anie KA, Green J. Psychological therapies for sickle cell disease and pain. *Cochrane Database Syst Reviews* 2012;**15(2)**:CD001916

Apostolo J, Bobrowicz-Campos E, Rodrigues M, Castro I, Cardoso D. The effectiveness of non-pharmacological interventions in older adults with depressive disorders: A systematic review. *International Journal of Nursing Studies* 2016;**58**:59-70

Arroyo K, Lundahl B, Butters R, Vanderloo M, Wood DS. Short-Term Interventions for Survivors of Intimate Partner Violence: A Systematic Review and Meta-Analysis. *Trauma Violence & Abuse* 2015;**02**:02

Ashman L, Duggan L. Interventions for learning disabled sex offenders. *Cochrane Database Syst Reviews* 2002;**(2)**:CD003682

Auclair V, Harvey P-O, Lepage M. La thérapie cognitive-comportementale dans le traitement du TDAH chez l'adulte. *Sante Mentale au Quebec* 2016;**41**:291-311

Bandelow B, Seidler-Brandler U, Becker A, Wedekind D, Ruther E. Meta-analysis of randomized controlled comparisons of psychopharmacological and psychological treatments for anxiety disorders. . *World Journal of Biological Psychiatry* 2007;**8(3)**:175-87

Beelmann A, Losel F. El entrenamiento en habilidades sociales en la prevencion temprana de la delincuencia: Los efectos en la conducta antisocial y la competencia social. *Psicothema* 2006;**18**:603-10

Belleville G, Cousineau H, Levrier K, St-Pierre-Delorme ME. Meta-analytic review of the impact of cognitive-behavior therapy for insomnia on concomitant anxiety. *Clinical Psychology Review* 2011;**31**:638-52

Beltman MW, Oude Voshaar RC, Speckens AE. Cognitive-behavioural therapy for depression in people with a somatic disease: meta-analysis of randomised controlled trials *British Journal of Psychiatry* 2010;**197(1)**:11-9

Bender JL, Radhakrishnan A, Diorio C, Englesakis M, Jadad AR. Can pain be managed through the Internet? A systematic review of randomized controlled trials. . *Pain* 2011;**152(8)**:1740-50

Bernardy K, Fuber N, Kollner V, Hauser W. Efficacy of cognitive-behavioral therapies in fibromyalgia syndrome: a systematic review and metaanalysis of randomized controlled trials. *Journal of Rheumatology* 2010;**37(10)**:1991-2005

Beugen S, Ferwerda M, Hoeve D, Rovers MM, Spillekom-van Koulil S, Middendorp H*, et al.* Internet-based cognitive behavioral therapy for patients with chronic somatic conditions: a meta-analytic review. *Journal of Medical Internet Research* 2014;**16(3)**:e88

Bisson J, Andrew M. Psychological treatment of post-traumatic stress disorder (PTSD). *Cochrane Database Syst Rev* 2005;**18(2)**:CD003388

Bisson J, Andrew M. Psychological treatment of post-traumatic stress disorder (PTSD). *Cochrane Database of Systematic Reviews* 2007;**18(3)**:CD003388

Bisson JI, Ehlers A, Matthews R, Pilling S, Richards D, Turner S. Psychological treatments for chronic post-traumatic stress disorder: systematic review and meta-analysis. *British Journal of Psychiatry* 2007;**190**:97-104

Brasure M, MacDonald R, Fuchs E, Olson CM, Carlyle M, Diem S*, et al. Management of Insomnia Disorder. Comparative Effectiveness Review No. 159*. Rockville (MD): Agency for Healthcare Research and Quality (US). Report No: 15(16)-EHC027-EF; 2015.

Brennan L, Murphy KD, Shaw KA, McKenzie JE. Psychological interventions for overweight or obesity. *Cochrane Database Syst Rev* 2014;**5**:CD003818

Buchanan J, Zakrzewska J. Burning mouth syndrome. *BMJ clinical evidence* 2008;**14**:1301

Buckley LA, Pettit T, Adams CE. Supportive therapy for schizophrenia. *Cochrane Database Syst Rev* 2007;**18(3)** CD004716

Casacalenda N, Perry JC, Looper K. Remission in major depressive disorder: a comparison of pharmacotherapy, psychotherapy, and control conditions. *American Journal of Psychiatry* 2002;**159(8)**:1354-60

Chang CW, Mu PF, Jou ST, Wong TT, Chen YC. The effectiveness of non-pharmacological interventions on fatigue in children and adolescents with cancer: a systematic review. *JBI Database of Systematic Reviews and Implementation Reports* 2012;**10(10)**:574-614

Chi N-C, Demiris G, Lewis FM, Walker AJ, Langer SL. Behavioral and Educational Interventions to Support Family Caregivers in End-of-Life Care. *American Journal of Hospice & Palliative Medicine* 2016;**33**:894-908

Cleary M, Hunt G, Matheson S, Siegfried N, Walter G. Psychosocial interventions for people with both severe mental illness and substance misuse. *Cochrane Database Syst Rev* 2008;**23(1)**:CD001088

Cormac I, Jones C, Campbell C. Cognitive behaviour therapy for schizophrenia. *Cochrane Database Syst Rev* 2002;**(1)**:CD000524

Covin R, Ouimet AJ, Seeds PM, Dozois DJ. A meta-analysis of CBT for pathological worry among clients with GAD. *Journal of Anxiety Disorders* 2008;**22**:108-16

Cuijpers P, Straten A, Andersson G. Internet-administered cognitive behavior therapy for health problems: a systematic review. *Journal of Behavioral Medicine* 2008;**31(2)**:169-77

Cuijpers P, Straten A, Smit F. Psychological treatment of late-life depression: a meta-analysis of randomized controlled trials. . *International Journal of Geriatric Psychiatry* 2006;**21(12)**:1139-49

Cuijpers P, Ven Straten A, Warmerdam L, Andersson G. Psychotherapy versus the combination of psychotherapy and pharmacotherapy in the treatment of depression: A meta-analysis. *Depression and Anxiety* 2009;**26**:279-88

Driessen E, Cuijpers P, Hollon SD, Van HL, Dekker JJ. The efficacy of psychological treatments for depression: a review of recent research findings. *Tijdschrift voor Psychiatrie* 2014;**56(7)**:455-62

Eccleston C, Williams ACDC, Morley S. Psychological therapies for the management of chronic pain (excluding headache) in adults. *Cochrane Database Syst Rev* 2009;**15(2)**:CD007407

Edwards AG, Hailey S, Maxwell M. Psychological interventions for women with metastatic breast cancer. *Cochrane Database Syst Rev* 2004;**(2)**:CD004253

Edwards AGK, Hulbert-Williams N, Neal RD. Psychological interventions for women with metastatic breast cancer. *Cochrane Database Syst Rev* 2008;**(3)**:CD004253

Elderton AJ. *Posttraumatic growth in survivors of interpersonal violence in adulthood*: University of Oxford; 2013.

Ewing DL, Monsen JJ, Thompson EJ, Cartwright-Hatton S, Field A. A meta-analysis of transdiagnostic cognitive behavioural therapy in the treatment of child and young person anxiety disorders. *Behavioural and Cognitive Psychotherapy* 2013;**43(5)**:562-77

Ford AC, Quigley EM, Lacy BE, Lembo AJ, Saito YA, Schiller LR*, et al.* Effect of antidepressants and psychological therapies, including hypnotherapy, in irritable bowel syndrome: systematic review and meta-analysis. *The American journal of gastroenterology* 2014;**109**:1350-65; quiz 66

Furlong M, McGilloway S, Bywater T, Hutchings J, Smith SM, Donnelly M. Behavioural and cognitive-behavioural group-based parenting programmes for early-onset conduct problems in children aged 3 to 12 years. *Evidence-Based Child Health* 2013;**7(8)**:318-692

Galsworthy-Francis L. *The development and exploration of the Experiences of Humiliation Scale (EHS) in an eating disordered population*: University of Leicester; 2012.

Gandy M, Sharpe L, Perry KN. Cognitive behavior therapy for depression in people with epilepsy: A systematic review. *Epilepsia* 2013;**54**:1725-34

Gartlehner G, Gaynes BN, Amick HR, Asher GN, Morgan LC, Coker-Schwimmer E*, et al.* Comparative benefits and harms of antidepressant, psychological, complementary, and exercise treatments for major depression: An evidence report for a clinical practice guideline from the American College of Physicians. *Annals of Internal Medicine* 2016;**164**:331-41

Gómez Puente JM, Martínez-Marcos M. Sobrepeso y obesidad: eficacia de las intervenciones en adultos. *Enfermeria Clinica* 2018;**28**:65-74

Goschwitz R, Plener P. Psychotherapeutic interventions for non-suicidal self-injury. *Nervenheilkunde: Zeitschrift fur interdisziplinaere Fortbildung* 2013;**32**:30-6

Gregory VL. Cognitive-behavioral therapy for comorbid bipolar and substance use disorders: a systematic review of controlled trials. *Mental Health and Substance Use* 2011;**4**:302-13

Haniffa M, Lasserson TJ, Smith I. Interventions to improve compliance with continuous positive airway pressure for obstructive sleep apnoea. *Cochrane Database Syst Rev* 2004;**18(4)**:CD003531

Hay PJ, Bacaltchuk J. Psychotherapy for bulimia nervosa and binging. *Cochrane Database Syst Rev* 2000;**(4)**:CD000562

Hay PJ, Bacaltchuk J. Psychotherapy for bulimia nervosa and binging. *Cochrane Database Syst Rev* 2001;**(3)**:CD000562

Hay PJ, Bacaltchuk J. Psychotherapy for bulimia nervosa and binging. *Cochrane Database Syst Rev* 2003;**(1)**:CD000562

Hay PJ, Bacaltchuk J. Bulimia nervosa. *BMJ clinical evidence* 2008;**12**

Hay PJ, Bacaltchuk J, Stefano S. Psychotherapy for bulimia nervosa and binging. *Cochrane Database Syst Rev* 2004;**(3)**:CD000562

Haynes RB, Ackloo E, Sahota N, McDonald HP, Yao X. Interventions for enhancing medication adherence. *Cochrane Database Syst Rev* 2014;**16(2)**:CD000011

Hazell P. Depression in children and adolescents. *BMJ clinical evidence* 2011;**8**

Hetrick SE, Cox GR, Merry SN. Treatment-resistant depression in adolescents: is the addition of cognitive behavioral therapy of benefit? *Psychology Research & Behavior Management* 2011;**4**:97-112

Hetrick SE, Cox GR, Merry SN. Where to go from Here? An Exploratory Meta-Analysis of the Most Promising Approaches to Depression Prevention Programs for Children and Adolescents. *International Journal of Environmental Research and Public Health* 2015;**12**:4758-95

Hjorthøj C, Fohlmann A, Nordentoft M. Treatment of cannabis use disorders in people with schizophrenia spectrum disorders — A systematic review. *Addictive Behaviors* 2009;**34**:520-5

Ho BPV, Carter M, Stephenson J. Anger Management Using a Cognitive-behavioural Approach for Children with Special Education Needs: A literature review and meta-analysis. *International Journal of Disability, Development and Education* 2010;**57(3)**:245-65

Hofmann SG, Sawyer AT, Korte KJ, Smits JA. Is it Beneficial to Add Pharmacotherapy to Cognitive-Behavioral Therapy when Treating Anxiety Disorders? A Meta-Analytic Review. *International Journal of Cognitive Therapy* 2009;**2**:160-75

Hofmann SG, Smits JAJ. Cognitive-behavioral therapy for adult anxiety disorders: A meta-analysis of randomized placebo-controlled trials. *Journal of Clinical Psychiatry* 2008;**69**:621-32

Huang, Ff, Li, Zj, Han, Hy*, et al.* Cognitive behavioral therapy combined with pharmacotherapy for obsessive compulsive disorder: a meta-analysis *Chinese Mental Health Journal* 2013;**27(9)**:643-9

Huertas-Ceballos AA, Logan S, Bennett C, Macarthur C. Psychosocial interventions for recurrent abdominal pain (RAP) and irritable bowel syndrome (IBS) in childhood. *Cochrane Database Syst Rev* 2014;**17(2)**:CD003014

Irwin MR, Cole JC, Nicassio PM. Comparative meta-analysis of behavioral interventions for insomnia and their efficacy in middle-aged adults and in older adults 55+ years of age. *Health Psychology* 2006;**25(1)**:3-14

James A, Soler A, Weatherall R. Cognitive behavioural therapy for anxiety disorders in children and adolescents. *Cochrane Database Syst Rev* 2005;**19(4)**:CD004690

James AC, James G, Cowdrey FA, Soler A, Choke A. Cognitive behavioural therapy for anxiety disorders in children and adolescents. *Cochrane Database Syst Rev* 2013;**6**:CD004690

Jones C, Cormac I, Mota J, Campbell C. Cognitive behaviour therapy for schizophrenia. *Cochrane Database Syst Rev* 2000;**(2)**:CD000524

Jones C, Cormac I, Silveira da Mota Neto JI, Campbell C. Cognitive behaviour therapy for schizophrenia. *Cochrane Database Syst Rev* 2004;**(4)**:CD000524

Jones C, Hacker D, Meaden A, Cormac I, Irving CB. WITHDRAWN: Cognitive behaviour therapy versus other psychosocial treatments for schizophrenia. *Cochrane Database Syst Rev* 2011;**(4)**:CD000524

Jones C, Hacker D, Meaden A, Cormac I, Irving Claire B. Cognitive behaviour therapy versus other psychosocial treatments for schizophrenia. *Cochrane Database Syst Rev* 2011;**13(4)**:CD000524

Kaltenthaler E, Shackley P, Stevens K, Beverley C, Parry G, Chilcott J. A systematic review and economic evaluation of computerised cognitive behaviour therapy for depression and anxiety. *Health Technology Assessment* 2002;**6(22)**:1-89

Kavanagh J, Oliver S, Lorenc T, Caird J, Tucker H, Harden A*, et al.* School-based cognitive-behavioural interventions: a systematic review of effects and inequalities. *Health Sociology Review* 2009;**18**:61-78

Kenworthy T, Adams CE, Bilby C, Brooks-Gordon B, Fenton M. WITHDRAWN: Psychological interventions for those who have sexually offended or are at risk of offending. *Cochrane Database Syst Rev* 2008;**(4)**:CD004858

Kisely S, Campbell LA, Skerritt P. Psychological interventions for symptomatic management of non-specific chest pain in patients with normal coronary anatomy. *Cochrane Database Syst Rev* 2005;**(1)**:CD004101

Kisely SR, Campbell LA, Skerritt P, Yelland MJ. Psychological interventions for symptomatic management of non-specific chest pain in patients with normal coronary anatomy. *Cochrane Database Syst Rev* 2010;**(1)**:CD004101

Kisely SR, Campbell LA, Yelland MJ, Paydar A. Psychological interventions for symptomatic management of non-specific chest pain in patients with normal coronary anatomy. *Cochrane Database Syst Rev* 2012;**6**:CD004101

Klimas J, Field CA, Cullen W, O'Gorman CS, Glynn LG, Keenan E*, et al.* Psychosocial interventions to reduce alcohol consumption in concurrent problem alcohol and illicit drug users. *Cochrane Database Syst Rev* 2012;**11**:CD009269

Klimas J, Field CA, Cullen W, O'Gorman CS, Glynn LG, Keenan E*, et al.* Psychosocial interventions to reduce alcohol consumption in concurrent problem alcohol and illicit drug users: Cochrane Review. *Systematic reviews* 2013;**2**:3

Kollner V, Hauser W, Klimczyk K, Kuhn-Becker H, Settan M, Weigl M*, et al.* Psychotherapy for patients with fibromyalgia syndrome: systematic review, meta-analysis and guideline. *Schmerz* 2012;**26**:291-6

Kollner V, Hauser W, Klimczyk K, Kuhn-Becker H, Settan M, Weigl M*, et al.* Psychotherapy for patients with fibromyalgia syndrome. Systematic review, meta-analysis and guideline. *Schmerz* 2012;**26**:291-6

Kroenke K. Efficacy of treatment for somatoform disorders: A review of randomized controlled trials. *Psychosomatic Medicine* 2007;**69**:881-8

Larkin D, Lopez V, Aromataris E. Non-pharmacological interventions for cancer-related fatigue in men treated for prostate cancer: A systematic review. *JBI Library of Systematic Reviewis* 2012;**10**:3764-811

Larun L, Brurberg KG, Odgaard-Jensen J, Price JR. Exercise therapy for chronic fatigue syndrome. *Cochrane Database Syst Rev* 2015;**(2)** CD003200

Larun L, Brurberg KG, Odgaard-Jensen J, Price JR. Exercise therapy for chronic fatigue syndrome. *Cochrane Database of Systematic Reviews* 2016;**(12)**:CD003200

Lewandowski LM, Gebing TA, Anthony JL, O'Brien WH. Meta-analysis of cognitive-behavioral treatment studies for bulimia. *Clinical Psychology Review* 1997;**17**:703-18

Linde K, Sigterman K, Kriston L, Rucker G, Jamil S, Meissner K*, et al.* Effectiveness of psychological treatments for depressive disorders in primary care: systematic review and meta-analysis. *Annals of Family Medicine* 2015;**13**:56-68

Lip GY, Lane DA, Millane TA, Tayebjee MH. Psychological interventions for depression in adolescent and adult congenital heart disease. *Cochrane Database Syst Rev* 2003;**(3)**:CD004394

Liu HX, Liang QJ, Xiao P, Jiao HX, Gao Y, Ahmetjiang A. The effectiveness of cognitive-behavioural therapy for temporomandibular disorders: a systematic review. *Journal of Oral Rehabilitation* 2012;**39**:55-62

Lockwood C, Page T, Conroy-Hiller T. Effectiveness of individual therapy and group therapy in the treatment of schizophrenia. *International Journal of Evidence-Based Healthcare* 2004;**2**:309-38

Macdonald G, Higgins JP, Ramchandani P, Valentine JC, Bronger LP, Klein P*, et al.* Cognitive-behavioural interventions for children who have been sexually abused. *Cochrane Database Syst Rev* 2012;**16(5)**:CD001930

Macdonald GM, Higgins JP, Ramchandani P. Cognitive-behavioural interventions for children who have been sexually abused. *Cochrane Database Syst Rev* 2006;**(4)**:CD001930

Macea DD, Gajos K, Daglia Calil YA, Fregni F. The efficacy of web-based cognitive behavioral interventions for chronic pain: a systematic review and meta-analysis. *Journal of Pain* 2010;**11**:917-29

Malouff JM, Thorsteinsson EB, Rooke SE, Bhullar N, Schutte NS. Efficacy of cognitive behavioral therapy for chronic fatigue syndrome: A meta-analysis. *Clinical Psychology Review* 2008;**28**:736-45

Marshall M, Lockwood A. Early Intervention for psychosis. *Cochrane Database Syst Rev* 2004;**(2)**:CD004718

Marson AG, Maguire M, Ramaratnam S. Epilepsy. *BMJ clinical evidence* 2009;**28**

Matthews EE, Arnedt JT, McCarthy MS, Cuddihy LJ, Aloia MS. Adherence to cognitive behavioral therapy for insomnia: A systematic review. *Sleep Medicine Reviews* 2013;**17**:453-64

Mayo-Wilson E. *Media-delivered behavioural and cognitive behavioural therapy for anxiety : a systematic review of effectiveness and an exploratory study of consumer preferences*: Oxford University; 2011.

Mayo-Wilson E, Montgomery P. Media-delivered cognitive behavioural therapy and behavioural therapy (self-help) for anxiety disorders in adults. *Cochrane Database Syst Rev* 2007;**(1)**:CD005330

McDaid C, Trowman R, Golder S, Hawton K, Sowden A. Interventions for people bereaved through suicide: systematic review. *British Journal of Psychiatry* 2008;**193**:438-43

Meyer T, Hautzinger M. Cognitive behavioral therapy in addition to pharmacotherapy for manic depressive disorders: Empirical results. *Der Nervenarzt* 2002;**73**:620-8

Montgomery P, Dennis J. Cognitive behavioural interventions for sleep problems in adults aged 60+. *Cochrane Database Syst Rev* 2002;**(2)**:CD003161

Montgomery P, Dennis J. A systematic review of non-pharmacological therapies for sleep problems in later life. *Sleep Medicine Reviews* 2004;**8**:47-62

Morin CM, Hauri PJ, Espie CA, Spielman AJ, Buysse DJ, Bootzin RR. Nonpharmacologic treatment of chronic insomnia. An American Academy of Sleep Medicine review. *Sleep* 1999;**22**:1134-56

Ng TK, Wong DFK. The efficacy of cognitive behavioral therapy for Chinese people: A meta-analysis. *Australian & New Zealand Journal of Psychiatry* 2018;**52**:620-37

Oakley-Browne MA, Adams P, Mobberley PM. Interventions for pathological gambling. *Cochrane Database Syst Rev* 2000;**(2)**:CD001521

Oakley-Browne MA, Adams P, Mobberley PM. WITHDRAWN: Interventions for pathological gambling. *Cochrane Database Syst Rev* 2007;**(3)**:CD001521

Oei TP, Dingle G. The effectiveness of group cognitive behaviour therapy for unipolar depressive disorders. *Journal of Affective Disorders* 2008;**107**:5-21

O'Kearney R. Benefits of cognitive-behavioural therapy for children and youth with obsessive-compulsive disorder: re-examination of the evidence. *Australian and New Zealand Journal of Psychiatry* 2007;**41**:199-212

Orgeta V, Qazi A, Spector A, Orrell M. Psychological treatments for depression and anxiety in dementia and mild cognitive impairment: systematic review and meta-analysis. *British Journal of Psychiatry* 2015;**207**:293-8

Parsons AC, Shraim M, Inglis J, Aveyard P, Hajek P. Interventions for preventing weight gain after smoking cessation. *Cochrane Database Syst Rev* 2009;**(1)** CD006219

Passarela CDM, Mendes DD, De Jesus Mari J. A systematic review to study the efficacy of cognitive behavioral therapy for sexually abused children and adolescents with posttraumatic stress disorder. . *Revista de Psiquiatria Clinica* 2010;**37**:63-73

Perveen T, Mahmood S, Gosadi I, Mehraj J, Sheikh SS. Long term effectiveness of cognitive behavior therapy for treatment of postpartum depression: A systematic review and meta-analysis. *Journal of Pakistan Medical Students* 2013;**3**:198-204

Pignone M, Gaynes BN, Rushton JL, Mulrow CD, Orleans CT, Whitener BL*, et al. Screening for depression. Systematic Evidence Reviews, No. 6*. Rockville (MD): Agency for Healthcare Research and Quality (US); 2002.

Pinquart M, Sörensen S. How effective are psychotherapeutic and other psychosocial interventions with older adults? *Journal of Mental Health & Aging* 2001:207-43

Ponniah K, Magiati I, Hollon SD. An update on the efficacy of psychological therapies in the treatment of obsessive-compulsive disorder in adults. *Journal of Obsessive-Compulsive & Related Disorders* 2013;**2**:207-18

Pratt HD. Psychotherapy in the age of pharmacology. *International Journal of Child and Adolescent Health* 2010;**3**:137-42

Pratt HD. Psychotherapy in the age of pharmacology. 2012:149-56

Price JR, Couper J. Cognitive behaviour therapy for adults with chronic fatigue syndrome. *Cochrane Database Syst Rev* 2000;**(2)**:CD001027

Ramaratnam S, Baker GA, Goldstein L. Psychological treatments for epilepsy. *Cochrane Database Syst Rev* 2001;**(4)**:CD002029

Ramaratnam S, Baker GA, Goldstein L. Psychological treatments for epilepsy. *Cochrane Database Syst Rev* 2003;**(4)**:CD002029

Ramaratnam S, Baker GA, Goldstein LH. Psychological treatments for epilepsy. *Cochrane Database Syst Rev* 2005;**19(4)**:CD002029

Ramaratnam S, Baker GA, Goldstein LH. Psychological treatments for epilepsy. *Cochrane Database Syst Rev* 2008;**16(3)**:CD002029

Ramaratnam S, Baker GA, Goldstein LH. Psychological treatments for epilepsy. *Cochrane Database Syst Rev* 2016;**25(2)** CD002029

Ramirez de Arellano MA, Lyman DR, Jobe-Shields L, George P, Dougherty RH, Daniels AS*, et al.* Trauma-focused cognitive-behavioral therapy for children and adolescents: assessing the evidence. *Psychiatr Serv* 2014;**65(5)**:591-602

Rector NA, Beck AT. Cognitive behavioral therapy for schizophrenia: An empirical review. *Journal of Nervous and Mental Disease* 2012;**200**:832-9

Reid SF, Chalder T, Cleare A, Hotopf M, Wessely S. Chronic fatigue syndrome. *BMJ clinical evidence* 2008:1101

Rimer J, Dwan K, Lawlor DA, Greig CA, McMurdo M, Morley W*, et al.* Exercise for depression. *Cochrane Database Syst Rev* 2012;**7**:CD004366

Roberts NP, Roberts PA, Jones N, Bisson JI. Psychological interventions for post-traumatic stress disorder and comorbid substance use disorder: A systematic review and meta-analysis. *Clinical Psychology Review* 2015;**38**:25-38

Schirmbeck F, Zink M. Cognitive behavioural therapy for obsessive-compulsive symptoms in schizophrenia *The Cognitive Behaviour Therapist* 2013;**6(e7)**:1-13

Schmid G, Henningsen P, Dieterich M, Sattel H, Lahmann C. Psychotherapy in dizziness: a systematic review. *Journal of Neurology, Neurosurgery and Psychiatry* 2011;**82**:601-6

Segal ZV, Whitney DK, Lam RW. Psychotherapy. *Canadian Journal of Psychiatry* 2001;**46**:29S-37S

Shaw K, O'Rourke P, Del Mar C, Kenardy J. Psychological interventions for overweight or obesity. *Cochrane Database Syst Rev* 2005:CD003818

Silver N. Headache (chronic tension-type). *BMJ clinical evidence* 2007:1205

Sin J, Spain D. Psychological interventions for trauma in individuals who have psychosis: A systematic review and meta-analysis. *Psychosis: Psychological, Social and Integrative Approaches* 2017;**9**:67-81

Smedslund G, Dalsbø TK, Steiro A, Winsvold A, Clench-Aas J. Cognitive behavioural therapy for men who physically abuse their female partner. *Cochrane Database Syst Rev* 2007;**18(3)**:CD006048

Soo S, Forman D, Delaney BC, Moayyedi P. A systematic review of psychological therapies for nonulcer dyspepsia. *American Journal of Gastroenterology* 2004;**99**:1817-22

Soo S, Moayyedi P, Deeks J, Delaney B, Lewis M, Forman D. Psychological interventions for non-ulcer dyspepsia. *Cochrane Databases Syst Rev* 2001;**(4)**:CD002301

Soo S, Moayyedi P, Deeks J, Delaney B, Lewis M, Forman D. Psychological interventions for non-ulcer dyspepsia. *Cochrane Databases Syst Rev* 2004;**(1)**:CD002301

Soo S, Moayyedi P, Deeks J, Delaney B, Lewis M, Forman D. Psychological interventions for non-ulcer dyspepsia. *Cochrane Databases Syst Rev* 2005;**18(2)**:CD002301

Soo S, Moayyedi P, Deeks JJ, Delaney B, Lewis M, Forman D. WITHDRAWN: Psychological interventions for non-ulcer dyspepsia. *Cochrane Databases Syst Rev* 2011;**16(2)**:CD002301

Soo S, Moayyedi P, Deeks Jonathan J, Delaney B, Lewis M, Forman D. Psychological interventions for non-ulcer dyspepsia. *Cochrane Database of Systematic Reviews* 2011;**16(2)**:CD002301

Soomro GM. Obsessive compulsive disorder. *BMJ clinical evidence* 2007

Spek V, Cuijpers P, NykIicek I, Riper H, Keyzer J, Pop V. Internet-based cognitive behaviour therapy for symptoms of depression and anxiety: A meta-analysis. *Psychological Medicine* 2007;**37**:319-28

Spence JD, Barnett PA, Linden W, Ramsden V, Taenzer P. Lifestyle modifications to prevent and control hypertension. 7. Recommendations on stress management. Canadian Hypertension Society, Canadian Coalition for High Blood Pressure Prevention and Control, Laboratory Centre for Disease Control at Health Canada, Heart and Stroke Foundation of Canada. *Canadian Medical Association journal* 1999;**160 (Suppl 9)**:S46-50

Stratford HJ. *Anxiety and bipolar spectrum disorders : psychological treatments and mental imagery*: University of Oxford; 2013.
Sumathipala A. What is the evidence for the efficacy of treatments for somatoform disorders?-A critical review of previous intervention studies. *Psychosomatic Medicine* 2009:103-30

Summerbell CD, Ashton V, Campbell KJ, Edmunds L, Kelly S, Waters E. Interventions for treating obesity in children. *Cochrane Database Syst Rev* 2003;**(3)**:CD001872

Sztein DM, Koransky CE, Fegan L, Himelhoch S. Efficacy of cognitive behavioural therapy delivered over the Internet for depressive symptoms: A systematic review and meta-analysis. *Journal of telemedicine and telecare* 2018;**24**:527-39

Tai-Wa LIU, Ng GYF, Chung RCK, Ng SSM. Cognitive behavioural therapy for fear of falling and balance among older people: a systematic review and meta-analysis. *Age & Ageing* 2018;**47**:520-7

Tatrow K, Montgomery GH. Cognitive behavioral therapy techniques for distress and pain in breast cancer patients: a meta-analysis. *Journal of Behavioral Medicine* 2006;**29**:17-27

Thakur ER, Shapiro J, Chan J, Lumley MA, Cully JA, Bradford A*, et al.* A Systematic Review of the Effectiveness of Psychological Treatments for IBS in Gastroenterology Settings: Promising but in Need of Further Study. *Digestive Diseases & Sciences* 2018;**10**:10

Torchalla I, Strehlau V. The Evidence Base for Interventions Targeting Individuals With Work-Related PTSD: A Systematic Review and Recommendations. *Behavior modification* 2018;**42**:273-303

Turner DT, Gaag M, Karyotaki E, Cuijpers P. Psychological interventions for psychosis: a meta-analysis of comparative outcome studies. . *Am J Psychiatry* 2014;**171(5)**:523-38

Turner W, Macdonald GM, Dennis JA. Cognitive-behavioural training interventions for assisting foster carers in the management of difficult behaviour. *Cochrane Database Syst Rev* 2005;**18(2)**:CD003760

Turner W, Macdonald G, Dennis JA. Cognitive-behavioural training interventions for assisting foster carers in the management of difficult behaviour. *Cochrane Database Syst Rev* 2007;**24(1)**:CD003760

Uman LS. *Psychological interventions for needle-related procedural pain and distress in children and adolescents: A systematic review and quality analysis*: Dalhousie University (Canada); 2010.

Uman LS, Chambers CT, McGrath PJ, Kisely S. Psychological interventions for needle-related procedural pain and distress in children and adolescents. *Cochrane Database Syst Rev* 2006;**(4)**:CD005179

Uman LS, Chambers CT, McGrath PJ, Kisely S. A systematic review of randomized controlled trials examining psychological interventions for needle-related procedural pain and distress in children and adolescents: an abbreviated Cochrane review. . *Journal of Pediatric Psychology* 2008;**33(8)**:842-54

Van Balkom AJLM, Barker A, Spinhoven P, Blaauw BMJW, Smeenk S, Ruesink B. A meta-analysis of the treatment of panic disorder with or without agoraphobia: A comparison of psychopharmacological, cognitive-behavioral, and combination treatments. *Journal of Nervous and Mental Disease* 1997;**185**:510-6

Veronese A, Hunot V, Cipriani A, Churchill R, Barbui C. Psychological therapies versus pharmacotherapy for obsessive compulsive disorder. *Cochrane Database Syst Rev* 2008;**(3)**:CD007319

Vulink N, Denys D. Body dysmorphic disorder: An overview. *Tijdschrift voor Psychiatrie* 2005;**47**:21-7

Waddell C, Hua JM, Garland OM, Peters RD, McEwan K. Preventing mental disorders in children. *Canadian Journal of Public Health* 2007;**98(3)** 166-73

Warren Z, Veenstra-VanderWeele J, Stone W, Bruzek JL, Nahmias AS, Foss-Feig JH*, et al. Therapies for Children with Autism Spectrum Disorders. Comparative Effectiveness Review No. 26*. Rockville (MD): Agency for Healthcare Research and Quality (US). Report No: 11-EHC029-EF; 2011.

Windmill J, Fisher E, Eccleston C, Derry S, Stannard C, Knaggs R*, et al.* Interventions for the reduction of prescribed opioid use in chronic non-cancer pain. *Cochrane Database Syst Rev* 2013;**1(9)**:CD010323

Yorke J, Fleming SL, Shuldham C. Psychological interventions for adults with asthma: A systematic review. *Respiratory Medicine* 2007;**101**:1-14

Zakrzewska JM, Forssell H, Glenny AM. Interventions for the treatment of burning mouth syndrome: a systematic review. *Journal of Orofacial Pain* 2003;**17**:293-300

Zakrzewska JM, Glenny AM, Forssell H. Interventions for the treatment of burning mouth syndrome. *Cochrane Database Syst Rev* 2001;**(3)**:CD002779

j. References of studies excluded as no CBT in title/abstract/keywords or not relevant to the overview (14)

Alharbi F, el-Guebaly N. Disulfiram: the survivor medication. *Addictive Disorders & Their Treatment* 2012;**11**:212-23.

Allen LA, Escobar JI, Lehrer PM, Gara MA, Woolfolk RL. Psychosocial treatments for multiple unexplained physical symptoms: a review of the literature. *Psychosomatic Medicine* 2002;**64**:939-50.

Anderson EM, Lambert MJ. Short-term dynamically oriented psychotherapy: A review and meta-analysis. *Clinical Psychology Review* 1995;**15**:503-14.

Anonymous. Exam 2: Efficacy of Psychosocial Interventions in Inducing and Maintaining Alcohol Abstinence in Patients With Chronic Liver Disease: A Systematic Review. *Clinical Gastroenterology and Hepatology* 2016;**14**:e20.

Berglund M. A better widget? Three lessons for improving addiction treatment from a meta-analytical study. *Addiction* 2005;**100**:742-50.

Bloom K, Tam JA. Walk-in services for child and family mental health. *Journal of Systemic Therapies* 2015;**34**:61-77.

Brown M, O'Neill N, van Woerden H, Eslambolchilar P, Jones M, John A. Gamification and Adherence to Web-Based Mental Health Interventions: A Systematic Review. *JMIR Mental Health* 2016;**3**:e39.

Chakraborty K, Basu D, Kumar KGV. Internet addiction: consensus, controversies, and the way ahead. *East Asian Archives of Psychiatry* 2010;**20**:123-32.

Clark GI, Rock AJ. Processes contributing to the maintenance of flying phobia: A narrative review. *Frontiers in Psychology* 2016;**7**.

Daker-White G, Rogers A. What is the potential for social networks and support to enhance future telehealth interventions for people with a diagnosis of schizophrenia: A critical interpretive synthesis. *BMC Psychiatry* 2013;**13**.

Edwards CJ, Cella M, Tarrier N, Wykes T. Investigating the empirical support for therapeutic targets proposed by the temporal experience of pleasure model in schizophrenia: A systematic review. *Schizophrenia Research* 2015;**168**:120-44.

Folmer RL, Theodoroff SM, Martin WH, Shi Y. Experimental, controversial, and futuristic treatments for chronic tinnitus. *Journal of the American Academy of Audiology* 2014;**25**:106-25.

Marks R, Allegrante JP. Effectiveness of psychoeducational interventions in osteoarthritis. *Critical Reviews in Physical & Rehabilitation Medicine* 2002;**14**:173-95.

Rozental A, Bennett S, Forsstrom D, Ebert DD, Shafran R, Andersson G*, et al.* Targeting Procrastination Using Psychological Treatments: A Systematic Review and Meta-Analysis. *Frontiers in Psychology* 2018;**9**:1588.

k. References of studies excluded as not able to obtain full-text (17)

Anders M, Christiansen H. Unaccompanied refugee minors: A systematic review of psychological interventions. *Kindheit und Entwicklung: Zeitschrift fur Klinische Kinderpsychologie* 2016;**25**:216-30.

Aslund L, Arnberg F, Kanstrup M, Lekander M. Cognitive and Behavioral Interventions to Improve Sleep in School-Age Children and Adolescents: A Systematic Review and Meta-Analysis. *Journal of Clinical Sleep Medicine* 2018;**14**:1937-47.

Cardi V, Treasure J. Treatments in eating disorders: Towards future directions. *Minerva Psichiatrica* 2010;**51**:191-206.

Chang CW, Mu PF, Jou ST, Wong TT, Chen YC. The effectiveness of non-pharmacological interventions on fatigue in children and adolescents with cancer: A systematic review. *JBI Library of Systematic Reviews* 2015;**10**:574-614.

Corbière M, Shen J. A systematic review of psychological return-to-work interventions for people with mental health problems and/or physical injuries. *Canadian Journal of Community Mental Health* 2006;**25**:261-88.

de Wit N, Rubin G, Jones RH. Irritable bowel syndrome. *BMJ clinical evidence* 2007.
DiMauro J. Exposure therapy for posttraumatic stress disorder: A meta-analysis. *Military Psychology* 2014;**26**:120-30.

Gherman A, David D. Are psychological interventions effective in diabetes care? A quantitative meta-analysis. *Erdelyi Pszichologiai Szemle* 2011;**12**:155-69.

Gratton S. *Evaluation of a Computer-Based CBT package for Exam Anxiety*: University of Surrey; 2007.

Heaton K. *Men with intellectual disabilities who display sexually abusive behaviour*: Lancaster University; 2010.

Hedgpeth NL. *Systematic review of psychosocial interventions for anxiety in adult cancer patients*: DNPc; 2012.

Kinsella PJ. *Paranoid ideation after traumatic brain injury : an exploration of related factors*: Bangor University; 2012.

Larkin D, Lopez V, Aromataris E. Non-pharmacological interventions for cancer-related fatigue in men treated for prostate cancer: A systematic review. *JBI Database of Systematic Reviews and Implementation Reports* 2014;**10**:3764-811.

Liu Z. *The effectiveness of cognitive behavioural interventions for the psychological morbidity of dementia family caregivers: A systematic review and meta-analysis*: The Chinese University of Hong Kong (Hong Kong); 2017.

Nicoll M. *Anger in offenders with intellectual disabilities*: University of Sheffield; 2011.

Paskins RT, Brady AM, Schultz JC. Applied Interventions in College Settings to Assist Adult Students with Autism Spectrum Disorder: A Systematic Review of the Literature. *Journal of Applied Rehabilitation Counseling* 2018;**49**:39-45.

Pratt HD. Psychotherapy in the age of pharmacology (2nd edition). In: DE Greydanus JC, DR Patel, A Nazeer and J Merrick, editor. *Clincial aspects of psychopharmacology in childhood and adolescence*Hauppauge, NY: Nova Science Publishers; US; 2017:83-91.

l. References of studies excluded as overviews of systematic reviews (44)

Aguiluz J, Alvarez M, Pimentel E, Abarca C, Moore P. How to face a patient with benzodiazepine dependence in primary health care? Strategies for withdrawal. *Medwave* 2018;**18**:e7159.

Astin JA, Shapiro SL, Eisenberg DM, Forys KL. Mind-body medicine: state of the science, implications for practice. *The Journal of the American Board of Family Practice / American Board of Family Practice* 2003;**16**:131-47.

Barlow JH, Ellard DR. Psycho-educational interventions for children with chronic disease, parents and siblings: an overview of the research evidence base. *Child: Care, Health & Development* 2004;**30**:637-45.

Bennett K, Manassis K, Duda S, Bagnell A, Bernstein GA, Garland EJ*, et al.* Treating child and adolescent anxiety effectively: Overview of systematic reviews. *Clinical Psychology Review* 2016;**50**:80-94.

Bhui KS, Dinos S, Stansfeld SA, White PD. A synthesis of the evidence for managing stress at work: A review of the reviews reporting on anxiety, depression, and absenteeism. *Journal of Environmental and Public Health* 2012;**2012**.

Bidonde J, Menseses J. *The effect of interventions for children who have experienced violence in close relationships: an overview of reviews*: Oslo: The Norwegian Institute of Public Health; 2017.

Butler AC, Chapman JE, Forman EM, Beck AT. The empirical status of cognitive-behavioral therapy: A review of meta-analyses. *Clinical Psychology Review* 2006;**26**:17-31.

Buysse DJ. Insomnia. *JAMA: Journal of the American Medical Association* 2013;**309**:706-16.

Chou R, Huffman LH. Nonpharmacologic therapies for acute and chronic low back pain: a review of the evidence for an American Pain Society/American College of Physicians clinical practice guideline [corrected] [published erratum appears in ANN INTERN MED 2008 Feb 5;148(3):247]. *Annals of Internal Medicine* 2007;**147**:492-45.

Cislak A, Safron M, Pratt M, Gaspar T, Luszczynska A. Family-related predictors of body weight and weight-related behaviours among children and adolescents: a systematic umbrella review. *Child: Care, Health & Development* 2012;**38**:321-31.

Cox G, Hetrick S. Psychosocial interventions for self-harm, suicidal ideation and suicide attempt in children and young people: What? How? Who? and Where? *Evidence Based Mental Health* 2017;**20**:35-40.

Crowe K, McKay D. Efficacy of cognitive-behavioral therapy for childhood anxiety and depression. *Journal of Anxiety Disorders* 2017;**49**:76-87.

Das JK, Salam RA, Lassi ZS, Khan MN, Mahmood W, Patel V*, et al.* Interventions for Adolescent Mental Health: An Overview of Systematic Reviews. *Journal of Adolescent Health* 2016;**59**:S49-S60.

Dragioti E, Dimoliatis I, Fountoulakis KN, Evangelou E. A systematic appraisal of allegiance effect in randomized controlled trials of psychotherapy. *Annals of General Psychiatry* 2015;**14**.

Dragioti E, Karathanos V, Gerdle B, Evangelou E. Does psychotherapy work? An umbrella review of meta-analyses of randomized controlled trials. *Acta Psychiatrica Scandinavica* 2017;**136**:236-46.

Driot D, Bismuth M, Maurel A, Soulie-Albouy J, Birebent J, Oustric S*, et al.* Management of first depression or generalized anxiety disorder episode in adults in primary care: A systematic meta review. *La Presse Medicale* 2017;**46**:1124-38.

Dufour S, Chamberland C. The effectiveness of selected interventions for previous maltreatment: enhancing the well-being of children who live at home. *Child & Family Social Work* 2004;**9**:39-56.

Duncan M, Moschopoulou E, Herrington E, Deane J, Roylance R, Jones L*, et al.* Review of systematic reviews of non-pharmacological interventions to improve quality of life in cancer survivors. *BMJ Open* 2017;**7**:e015860.

Eccleston C, Morley SJ, Williams AC. Psychological approaches to chronic pain management: Evidence and challenges. *British Journal of Anaesthesia* 2013;**111**:59-63.

Enns J, Holmqvist M, Wener P, Halas G, Rothney J, Schultz A*, et al.* Mapping interventions that promote mental health in the general population: A scoping review of reviews. *Preventive Medicine* 2016;**87**:70-80.

Farah WH, Alsawas M, Mainou M, Alahdab F, Farah MH, Ahmed AT*, et al.* Non-pharmacological treatment of depression: A systematic review and evidence map. *Evidence-Based Medicine* 2016;**21**:214-21.

Fishbain DA. Non-surgical chronic pain treatment outcome: A review. *International Review of Psychiatry* 2000;**12**:170-80.

Foroushani PS, Schneider J, Assareh N. Meta-review of the effectiveness of computerised CBT in treating depression. *BMC Psychiatry* 2011;**11**.

Gartlehner G, Wagner G, Matyas N, Titscher V, Greimel J, Lux L*, et al.* Pharmacological and non-pharmacological treatments for major depressive disorder: Review of systematic reviews. *BMJ Open* 2017;**7**.

Gustafsson C, Öjehagen A, Hansson L, Sandlund M, Nyström M, Glad J*, et al.* Effects of Psychosocial Interventions for People With Intellectual Disabilities and Mental Health Problems: A Survey of Systematic Reviews. *Research on Social Work Practice* 2009;**19**:281-90.

Health Quality Ontario. Psychotherapy for Major Depressive Disorder and Generalized Anxiety Disorder: A Health Technology Assessment. *Ontario Health Technology Assessment Series* 2017;**17**:1-167.

Health Quality Ontario. Cognitive Behavioural Therapy for Psychosis: A Health Technology Assessment. *Ontario Health Technology Assessment Series* 2018;**18**:1-141.

Hofmann SG, Asnaani A, Vonk IJJ, Sawyer AT, Fang A. The efficacy of cognitive behavioral therapy: A review of meta-analyses. *Cognitive Therapy and Research* 2012;**36**:427-40.

Jacobs S, Hassell K, Johnson S. The effectiveness of organisational stress management and prevention strategies: What can community pharmacy learn from existing evidence? *International Journal of Pharmacy Practice* 2013;**21**:78-9.

Joyce S, Modini M, Christensen H, Mykletun A, Bryant R, Mitchell P*, et al.* Workplace interventions for common mental disorders: A systematic meta-review. *Psychological Medicine* 2016;**46**:683-97.

Khan F, Amatya B. Rehabilitation in Multiple Sclerosis: A Systematic Review of Systematic Reviews. *Archives of Physical Medicine & Rehabilitation* 2017;**98**:353-67.

Lee AH, DiGiuseppe R. Anger and aggression treatments: a review of meta-analyses. *Current Opinion in Psychology* 2018;**19**:65-74.

Louw Q, Morris L, Sklaar J. Evidence of physiotherapeutic interventions for acute LBP patients. *South African Journal of Physiotherapy* 2007;**63**:7-14.

Miller P, Soundy A. The pharmacological and non-pharmacological interventions for the management of fatigue related multiple sclerosis. *Journal of the Neurological Sciences* 2017;**381**:41-54.

Minelli A, Vaona A. Effectiveness of cognitive behavioral therapy in the treatment of fibromyalgia syndrome: A meta-analytic literature review. *Reumatismo* 2012;**64**:151-7.

Morin L, Franck N. Rehabilitation interventions to promote recovery from schizophrenia: A systematic review. *Frontiers in Psychiatry* 2017;**8**:100.

Munthe-Kaas HM, Johansen S, Blaasvaer N, Hammerstrom KT, Nilsen W. *The effect of psychosocial interventions for prevention and treating depression and anxiety among at-risk children and adolescents (report 22)*: Oslo: The Norwegian Institute of Public Health (NIPH) 2014.

Palmer T. Programmatic and nonprogrammatic aspects of successful intervention. 1996:131-82.
Paulus FW, Ohmann S, Popow C. Practitioner Review: School-based interventions in child mental health. *Journal of Child Psychology & Psychiatry* 2016;**57**:1337-59.

Peters LW, Kok G, Ten Dam GT, Buijs GJ, Paulussen TG. Effective elements of school health promotion across behavioral domains: a systematic review of reviews. *BMC Public Health* 2009;**9**:182.

Prothero L, Barley E, Galloway J, Georgopoulou S, Sturt J. The evidence base for psychological interventions for rheumatoid arthritis: A systematic review of reviews. *International Journal of Nursing Studies* 2018;**82**:20-9.

Riemann D, Baglioni C, Bassetti C, Bjorvatn B, Dolenc Groselj L, Ellis JG*, et al.* European guideline for the diagnosis and treatment of insomnia. *Journal of Sleep Research* 2017;**26**:675-700.

Stamou G, Garcia-Palacios A, Botella C. Cognitive-Behavioural therapy and interpersonal psychotherapy for the treatment of post-natal depression: a narrative review. *BMC psychology* 2018;**6**:28.

Turrini G, Purgato M, Ballette F, Nose M, Ostuzzi G, Barbui C. Common mental disorders in asylum seekers and refugees: Umbrella review of prevalence and intervention studies. *International Journal of Mental Health Systems* 201

eFigure 1: Bubble map of CBT systematic review with < 1000 participants

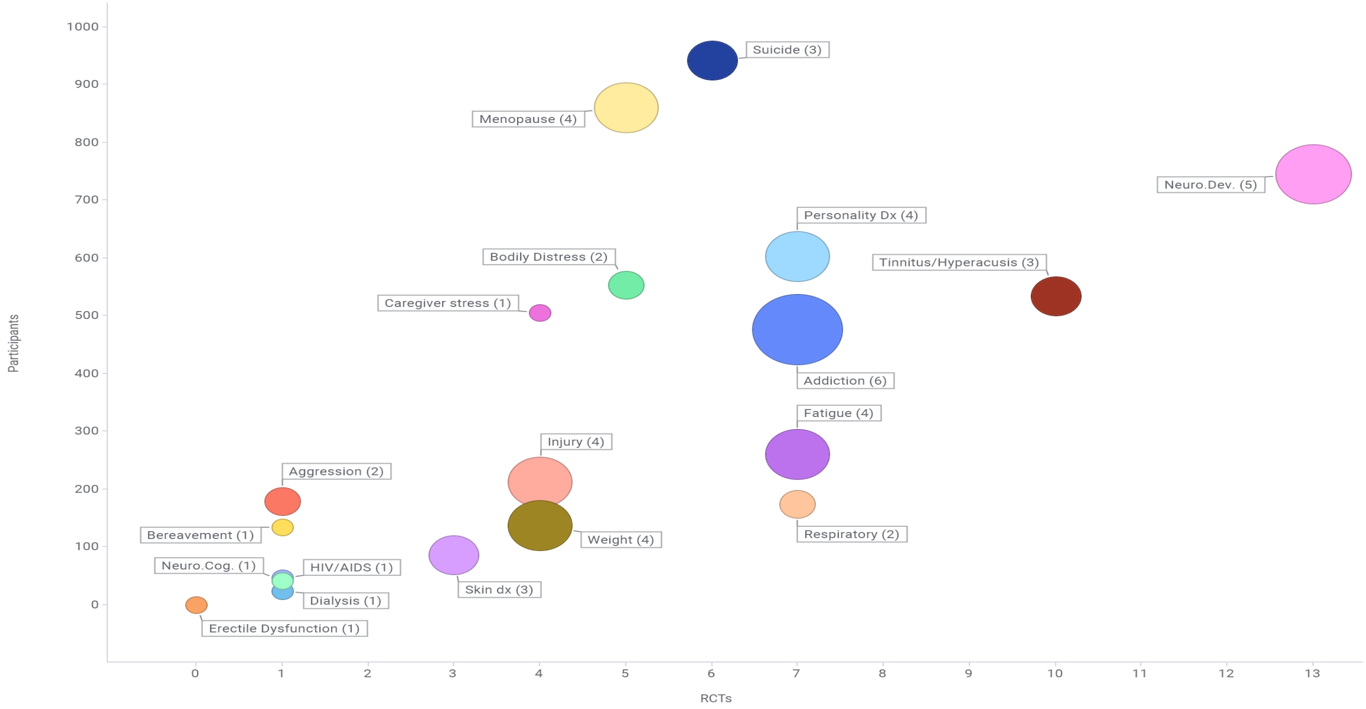

Key: Dx – diseases/disorders, Neuro.Cog – Neurocognitive, Neuro.Dev. – Neurodevelopmental. The size of the bubble represents the number of reviews included in our map. This is also denoted by the number in the brackets following the health condition description label.

eFigure 2: Bubble map of CBT systematic review with >1000 participants

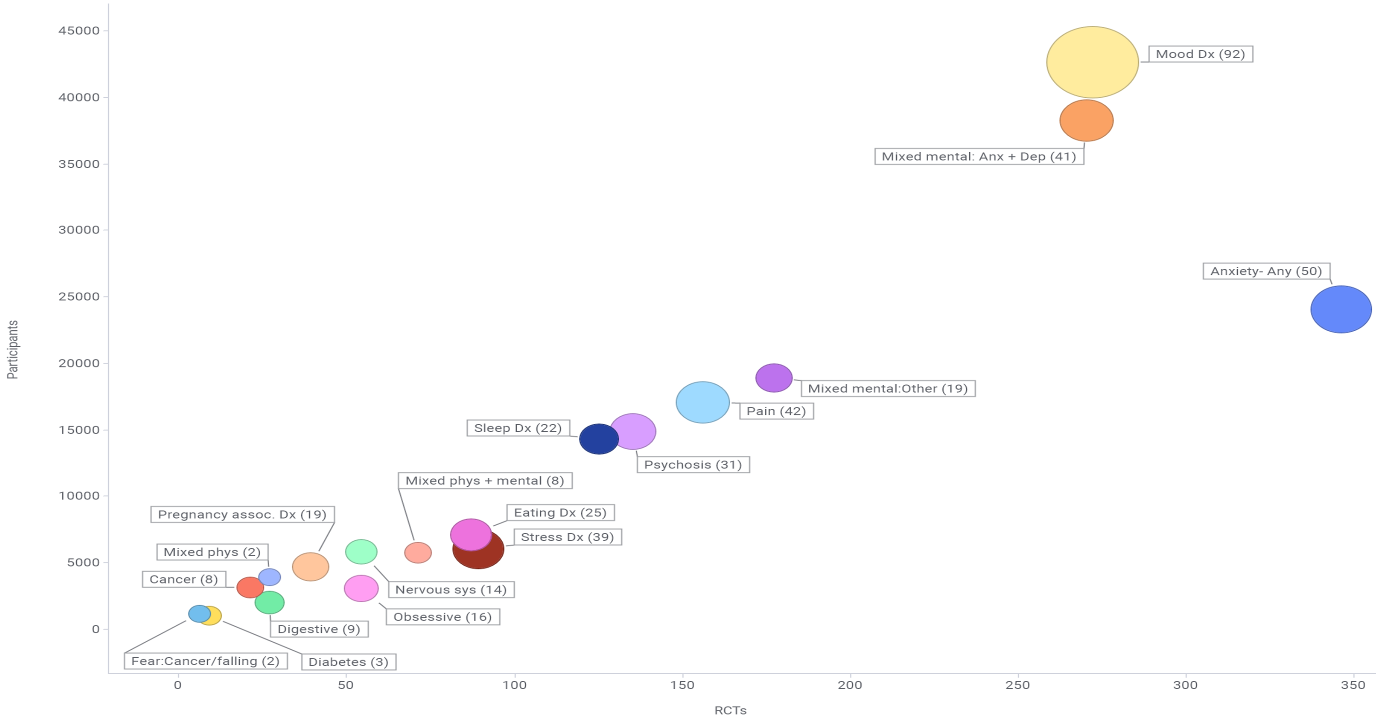

Key: 'Anx' – anxiety, 'Anxiety-any' – Reviews of RCTs conducted in patients with any anxiety disorder listed under ICD-11 Anxiety or fear related disorders. 'Dep' – depression, 'Dx' – diseases/disorders, 'Mixed phys/mixed phys + mental' – Reviews including trials conducted in different physical and or mental conditions, **The size of the bubble represents the number of reviews included in our map. This is also denoted by the number in the brackets following the health condition description label*

eFigure 3: Depression panoramic meta-analysis

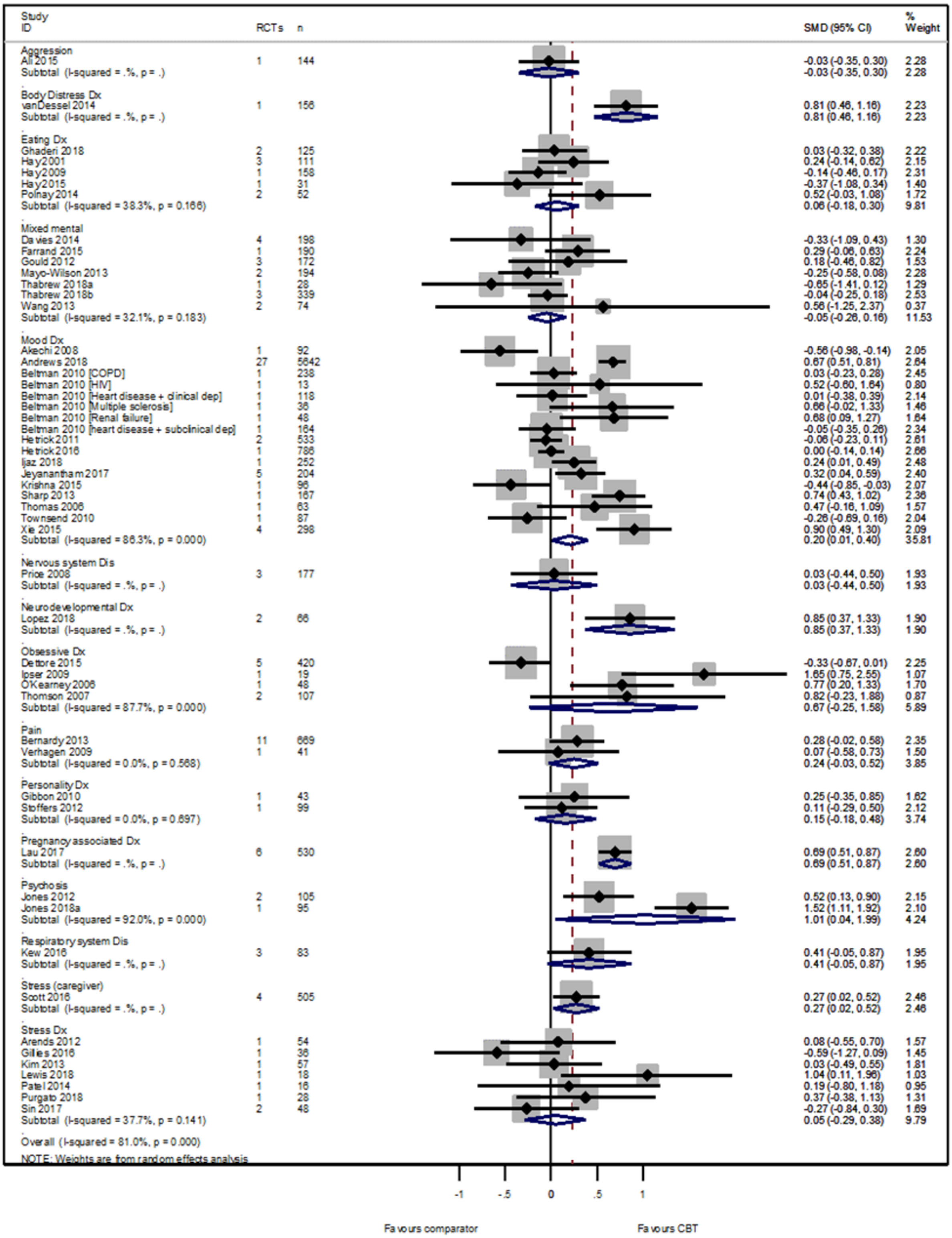

eTable 1: Populations characteristics of the mapped reviews

Reviews	Age				Sex				Ethnicity					Countries where the included RCTs were conducted		
	**<18 years old**	**18-65 years old**	**>65 years old**	***NR***	**<50% female sample**	**>50% female sample**	**Mixed**	***NR***	**<25% white sample**	**25-75% white sample**	**>75% white sample**	**Mixed**	***NR***	**Europe, North America, Australasia**	**Africa, Asia, South America**	***NR***
**n=494**	108<br>22%	378<br>77%	30<br>6%	*19*<br>*4%*	44<br>9%	167<br>34%	65<br>13%	*218*<br>*44%*	9<br>2%	6<br>1%	10<br>2%	11<br>2%	*458*<br>*93%*	231<br>47%	45<br>10%	*218*<br>*44%*

Key: 'Mixed' – Reviews which include trials which fit into each of our classification strata. 'NR' - Not Reported

Note: Some reviews (and RCTs) included participants across various sub-categories and therefore the total in each category may not always add up to 100% (n=494 reviews).

<u>eTable 2: Context characteristics of the mapped reviews</u>

**Reviews**	**Severity**					**Intensity**			**Delivered**				**Participants recruited**							**Follow-up**		
	**Sub-Clinical**	**Clinical**	**Chronic**	**Severe**	***Not reported***	**High**	**Low**	***Not reported***	**Preventative**	**Standard**	**Relapse**	***Not reported***	**Community**	**GP primary**	**Outpatients**	**Inpatients**	**School/Uni**	**Institution**	***Not reported***	**Short**	**Long**	***Not reported***
**n=494**	16<br>3%	216<br>44%	19<br>4%	10<br>2%	*247*<br>*50%*	397<br>80<br>%	139<br>28<br>%	*8*<br>*2%*	29<br>6%	463<br>94%	7<br>1%	*0*	92<br>19%	41<br>8%	114<br>23%	35<br>7%	36<br>7%	4<br>1%	*283*<br>*57%*	402<br>81%	130<br>26%	*7*<br>*1%*

<u>Key:</u> 'Uni' - University

Note: Some reviews (and RCTs) included participants across various sub-categories and therefore the total in each category may not always add up to 100% (n=494 reviews
